# Deep Sequencing of Maize Small RNAs Reveals a Diverse Set of MicroRNA in Dry and Imbibed Seeds

**DOI:** 10.1371/journal.pone.0055107

**Published:** 2013-01-24

**Authors:** Detao Li, Liwen Wang, Xu Liu, Dezhou Cui, Tingting Chen, Hua Zhang, Chuan Jiang, Chunyan Xu, Peng Li, Song Li, Li Zhao, Huabang Chen

**Affiliations:** 1 State Key Laboratory of Crop Biology, Shandong Agricultural University, Taian, Shandong, P. R. China; 2 State Key Laboratory of Plant Cell and Chromosome Engineering, Institute of Genetics and Developmental Biology, Chinese Academy of Sciences, Beijing, P. R. China; University of North Carolina at Charlotte, United States of America

## Abstract

Seed germination plays a pivotal role during the life cycle of plants. As dry seeds imbibe water, the resumption of energy metabolism and cellular repair occur and miRNA-mediated gene expression regulation is involved in the reactivation events. This research was aimed at understanding the role of miRNA in the molecular control during seed imbibition process. Small RNA libraries constructed from dry and imbibed maize seed embryos were sequenced using the Illumina platform. Twenty-four conserved miRNA families were identified in both libraries. Sixteen of them showed significant expression differences between dry and imbibed seeds. Twelve miRNA families, miR156, miR159, miR164, miR166, miR167, miR168, miR169, miR172, miR319, miR393, miR394 and miR397, were significantly down-regulated; while four families, miR398, miR408, miR528 and miR529, were significantly up-regulated in imbibed seeds compared to that in dry seeds. Furthermore, putative novel maize miRNAs and their target genes were predicted. Target gene GO analysis was performed for novel miRNAs that were sequenced more than 50 times in the normalized libraries. The result showed that carbohydrate catabolic related genes were specifically enriched in the dry seed, while in imbibed seed target gene enrichment covered a broad range of functional categories including genes in amino acid biosynthesis, isomerase activity, ligase activity and others. The sequencing results were partially validated by quantitative RT-PCR for both conserved and novel miRNAs and the predicted target genes. Our data suggested that diverse and complex miRNAs are involved in the seed imbibition process. That miRNA are involved in plant hormone regulation may play important roles during the dry-imbibed seed transition.

## Introduction

Plant gene expression is highly regulated to ensure proper development and function of tissues and adequate responses to abiotic and biotic stresses. Gene expression is often a multistep process and can be regulated at several levels. One of the most recently discovered regulatory mechanisms is post-transcriptional and involves 21–24 nt small RNA molecules [1]. The small RNA content of plant cells is surprisingly complex, suggesting an extensive regulatory role for these molecules [2]. The best-characterized class of plant small RNA is microRNA (miRNA) [3]. MiRNAs were first identified in *Caenorhabditis elegans* through genetic screens for aberrant development and were later found in almost all multicellular eukaryotes examined [4]. Mature miRNAs are single-stranded ∼21 nt small RNA which are generated from a single-stranded primary transcript by a series of enzymatic activities. Mature miRNAs down-regulate their target genes through the cleavage of mRNAs [5], translational repression [6], or transcriptional inhibition [7]. However, mRNA cleavage seems to be the predominant mechanism of miRNA-mediated regulation in plants [8]. Many miRNAs targets in plants are transcription factors [9], [10]. Transcription factors play key regulatory roles in plant development [10], however they are no longer needed after they function and may even be harmful for the next developmental stage. To maintain normal plant development, MiRNAs play crucial roles in the elimination of those unwanted factors [11].

As dry seeds imbibe water, the resumption of energy metabolism and cellular repair occur. Potential miRNA-mediated gene expression regulation has been suggested in seed development, dormancy and germination [12], [13]. For example, SPL13 (SQUAMOSA PROMOTER BINDING PROTEIN-LIKE 13) is the miR156 target gene and down-regulation of SPL13 by miR156 appears to be essential for the transition to the vegetative-leaf stages. Mutated SPL13 that is resistant to miR156 over-accumulates at the post germination stages and causes a delay in vegetative leaf development from the cotyledon-stage seedlings [14], [15]. Transcription factor ARF10 (AUXIN RESPONSE FACTOR10) is the target of miR160. The mutant expressing miR160 de-regulated ARF10 (mARF10) exhibited altered hormone sensitivity during seed germination and seedling growth in addition to a serrated leaf phenotype [16]. The balance between ABA and gibberellin (GA) is important for determining the dormancy status of seeds. ABA is abundant in dormant seeds and generally decreases during imbibition when seed dormancy is released [17] whereas GA increases during the transition to germination [18]. The miR159 was reported to targets MYB101 and MYB33 transcription factors, which are positive regulators of ABA signalling during *Arabidopsis* seed germination, suggesting that miR159 may play a role in seed germination [19].

In recent years, development of high-throughput pyrosequencing technology has revolutionized the discovery of miRNA species. High-throughput technologies has allowed the identification of several non-conserved or lowly expressed miRNAs through deep sequencing, e.g. in *Arabidopsis*, wheat and tomato [20]–[22]. The discovery of non-conserved miRNAs suggests that plant species/families with specific developmental features may contain non-conserved miRNAs that are involved in the regulation of gene expression specific to those features. Characterizing the stage-and tissue-specific expression of miRNAs is becoming more important for understanding the regulatory mechanisms of critical events during plant development.

Maize is one of the most important crops worldwide as well as a model plant for biological research. A number of miRNAs with specific function have been reported in maize. The expression of APETALA2 floral homeotic transcription factor, which is required for spikelet meristem determination, is regulated by miR172 [23]. MiR172 also regulates the APETALA2-like gene *glossy*15 to promote vegetative phase transition [24]. Teosinte glume architecture 1 (*tga*1), which plays an important role in maize domestication, has been identified as a target of miR156 [25]. The miR166 functions in acting on the asymmetry development of leaves in maize by regulating a class III homeodomain leucine zipper (HD-ZIPIII) protein [26]. However, the small RNA population in maize is highly complicated and only limited numbers of miRNAs and their target genes have been analysed in seed research to date.

Plants go through distinct phases during early stages of development. Dry seeds imbibe water and re-initiate active physiology [27]. The reactivation events such as the activation of genes encoding enzymes involved starch degradation and protein and DNA/RNA synthesis play important roles in the decision as to whether a seed will germinate or not. The shift from the seed development/maturation mode to the germination mode is a critical change in the developmental program of seeds. Regulation of transcription factors targeted by miRNAs is involved at this critical stage in plant development [28]. To investigate the roles of miRNAs in both dry and imbibed maize seeds and to identify potential seed-specific miRNAs, we sequenced small RNA populations of dry and imbibed maize seeds using the Illumina platform. Our results indicated that diverse miRNAs were involved in the imbibition process of maize seed.

## Results

### Deep Sequencing of Maize Small RNAs

**Figure 1:**
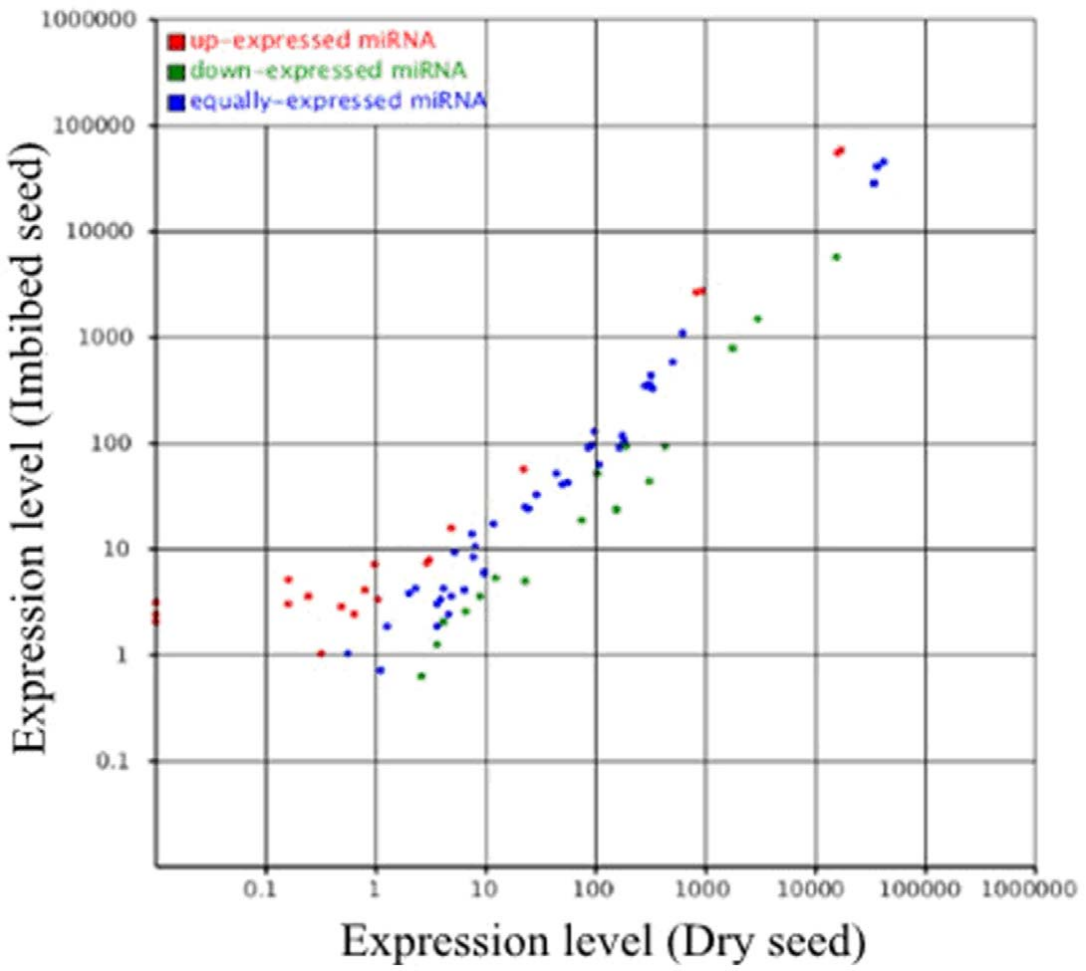
Small RNA expression profiles in dry and imbibed maize seeds.

**Figure 2:**
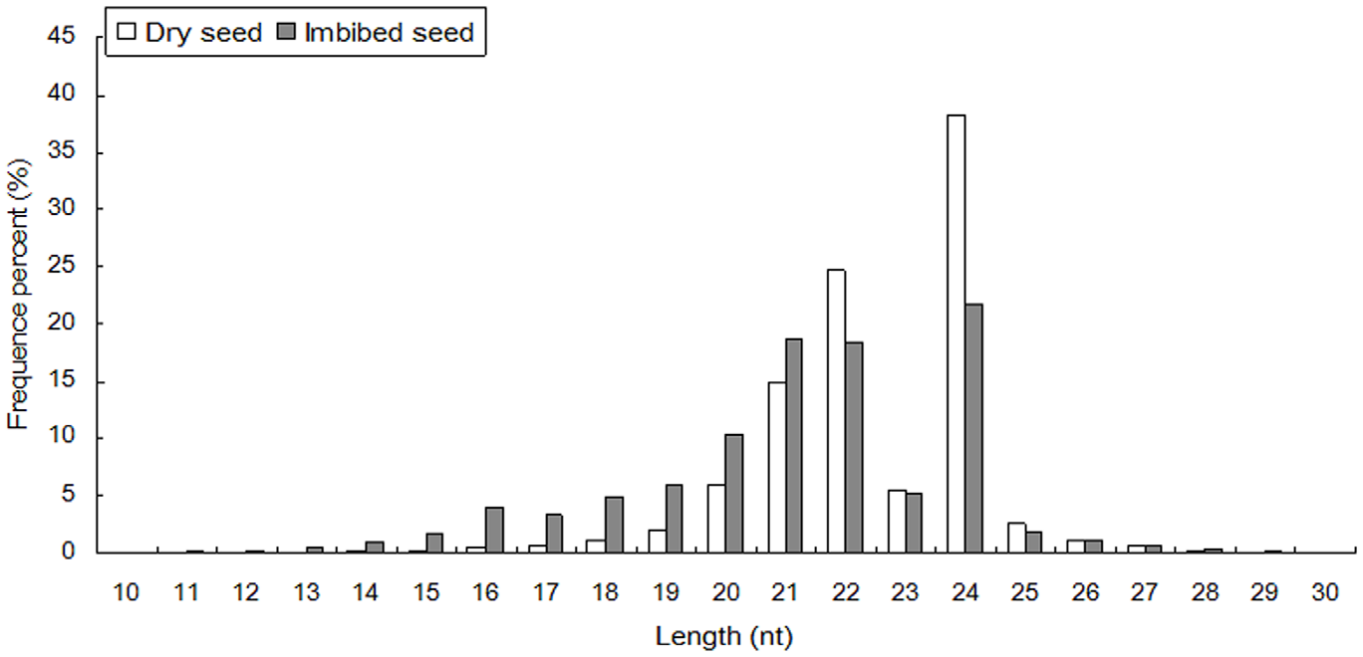
Length distribution and abundance of small RNAs from dry and imbibed maize seeds.

In order to identify the miRNAs involved in both dry and imbibed seeds, small RNA libraries from dry and imbibed seed embryos (imbibed 24 hours) were sequenced side by side using the Illumina platform. The statistics of the small RNA sequences from the two libraries were summarized in the tables (Table 1 and Table 2). A total of 16,991,216 and 11,321,391 raw reads were obtained from dry seeds and imbibed seeds, respectively. After removing the low quality sequences, adapter sequences, and sequences smaller than 18 nt, the remaining clean reads from the two libraries were aligned to the maize genome. A total of 12,341,357 and 9,735,651 clean reads were perfectly matched to the B73 genome (B73 RefGen_v2 (Release 5a.57 in May, 2010)). The two libraries generated 3,454,632 unique small RNA reads indicating a highly complex small RNA population in maize seed. For the total small RNAs, 75.89% were found in both libraries. For the unique small RNAs, however, only 14.33% were shared in both libraries, 18.55% were dry-seed-specific and 67.12% were imbibed-seed-specific, indicating dynamic small RNA population was expressed in the imbibed seeds (Table 3). Figure 1 showed that some small RNAs were up-regulated, some were down-regulated in imbibed seeds, and some were roughly equally expressed in both dry and imbibed seeds. The size of the small RNAs was not evenly distributed in both libraries, however, overall size distribution of the sequenced reads from the two sequencing efforts were very similar. The majority (>75%) of the small RNAs from both libraries was 20–24 nt, with the 24 nt being the most abundant, followed by 22 and 21 nt classes (Figure 2). This result was consistent with recent report in maize [29] and with that of *Medicago truncatula* [30], rice [31], peanut [32] and *Arabidopsis* [33].

**Table 1:** Summary of sRNA signatures that match various RNAs.

Type	DS count	Percent of total reads (%)	GS count	Percent of total reads (%)
total_reads	16991216		11321391	
high_quality	16423498	100	11083487	100
adaptor3_null	105794	0.64	6171	0.06
insert_null	4203	0.03	6474	0.06
adaptor5_contaminants	27371	0.17	170348	1.54
small_than_18nt	223194	1.36	1164672	10.51
polyA	805	0.00	171	0.00
clean reads	16062131	97.8	9735651	87.84

DS, Dry seed; GS, Imbibed seed.

**Table 2:** Summary of sRNA signatures that match various RNAs.

Locus class	DS Distinct signatures	DS Total signatures	GS Distinct signatures	GS Total signatures
snoRNA	1004 (0.09%)	21624 (0.18%)	1207 (0.04%)	9327 (0.10%)
snRNA	1010 (0.09%)	10892 (0.09%)	1459 (0.05%)	5249 (0.05%)
tRNA	6884 (0.61%)	945828 (7.66%)	10170 (0.36%)	490197 (5.04%)
rRNA	34927 (3.07%)	1634953 (13.25%)	93729 (3.33%)	2358514 (24.23%)
siRNA	10947 (0.96%)	163161 (1.32%)	45517 (1.62%)	267092 (2.74%)
exon_antisense	30209 (2.66%)	292579 (2.37%)	54497 (1.94%)	115300 (1.18%)
exon_sense	48908 (4.31%)	431167 (3.49%)	126466 (4.49%)	291957 (3.00%)
intron_antisense	19679 (1.73%)	125922 (1.02%)	96317 (3.42%)	242193 (2.49%)
intron_sense	37727 (3.32%)	343222 (2.78%)	225015 (8.00%)	680193 (6.99%)
Known miRNA	384 (0.03%)	1461286 (11.84%)	759 (0.03%)	1394428 (14.32%)
Novel miRNA	376 (0.03%)	14709 (0.72%)	584 (0.02%)	30939 (0.32%)
Other small RNAs	943828 (83.1%)	6896014 (55.88%)	2158063 (76.70%)	3850262 (39.55%)
Total	1135883	12341357	2813783	9735651

DS, Dry seed; GS, Imbibed seed.

**Table 3:** Comparison of small RNAs between dry and imbibed seeds.

	Unique sRNAs reads	Percent of unique sRNAs reads (%)	Totals RNAs reads	Percent of total RNAs reads (%)
Total sRNAs	3454632	100.00	22077008	100.00
DS & GS	495034	14.33	16754063	75.89
DS specific	640849	18.55	2370868	10.74
GS specific	2318749	67.12	2952077	13.37

DS, Dry seed; GS, Imbibed seed.

### Conserved miRNAs

**Figure 3:**
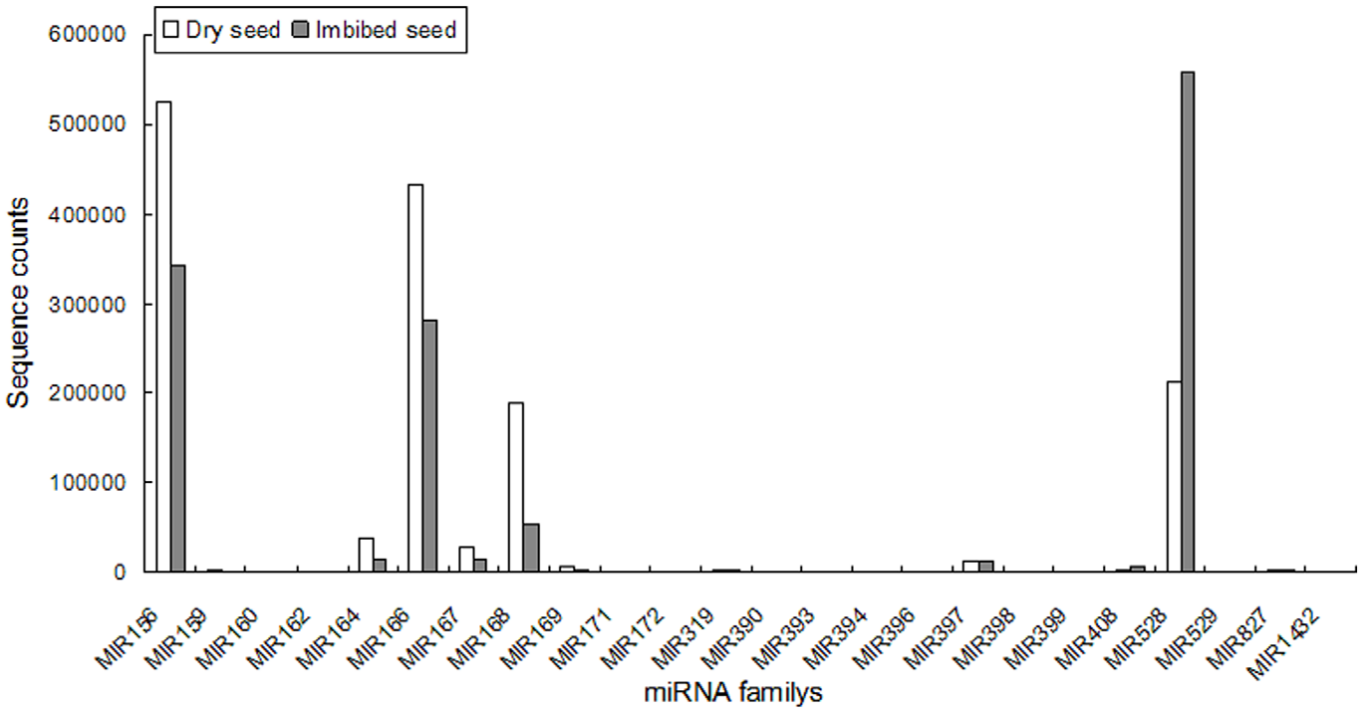
Abundance of conserved miRNAs in dry and imbibed maize seed small RNA libraries.

**Figure 4:**
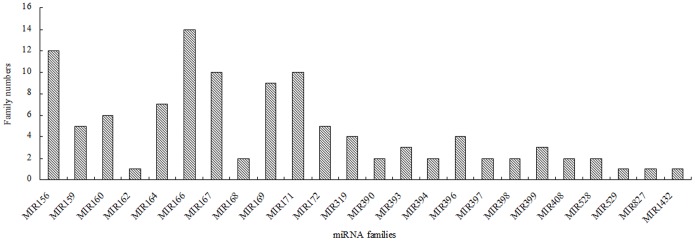
Number of detected family members per miRNA family. Candidate miRNA families were taken together and grouped by their miRBase numerical identifiers.

Conserved miRNA families were found in many plant species and had important functions in plant development. To identify conserved miRNAs in our dataset, all small RNA sequences were Blastn searched against the known mature miRNAs and their precursors in the miRNA database miRBase. There were currently 29 families containing 172 known miRNAs of maize in miRBase [34]. Aligning small RNA sequences to known miRNAs resulted in 1,461,286 and 1,394,428 matches for dry seed and imbibed seed, respectively. The statistics of the conserved miRNA families in dry and imbibed seeds was listed in the table (Table 4). Conserved miRNAs from dry and imbibed seeds had the following features in common. First, both libraries identified 24 conserved miRNA families consisting of 110 individual miRNAs (Additional File S1). The conserved miRNA families in both libraries showed similar abundance distribution. The most abundant families were miR156/miR157, miR166, miR168 and miR528, and the least abundant families were miR162 and miR399 (Figure 3). The conserved miRNA families in both libraries had identical family member distribution. The largest miRNA family size identified was miR166 that consisted of 14 members and miR156/157, miR167 and miR169 possessed 12, 10 and 9 members, respectively; whereas miR162, miR529, miR827 and miR1432 had only one member detected in the maize seeds (Figure 4). Second, the two libraries showed similar relative family member abundance pattern within a given miRNA family (Additional File S1). For instance, the sequencing frequencies for miR156d were 517,422 and 436,396, respectively, whereas the sequencing frequencies for miR156j were only 929 and 181, respectively. Third, 5 conserved miRNA families, miR395, miR444, miR482, miR2118 and miR2275 were not detected in both libraries. Fourth, the abundance of the originally annotated miRNA* of zma-miR397b was much higher (7736, 10727 times in two sequenced libraries) than its annotated miRNA (5240, 893 times in two sequenced libraries). This may suggest that a small fraction of miRNA* do not degrade as fast as others [29]. Although the identified conserved miRNA families in dry and imbibed seeds showed similarities in several aspects, significant differences existed between them. 16 of the 24 conserved miRNA families identified showed significant expression difference between dry and imbibed seeds at P = 0.01 level, with 12 significantly down-regulated and 4 significantly up-regulated in the imbibed seed compared to those of the dry seed. The 12 down-regulated miRNA families were miR156, miR159, miR164, miR166, miR167, miR168, miR169, miR172, miR319, miR393, miR394 and miR397. The 4 up-regulated families were miR398, miR408, miR528 and miR529. For both up- and down-regulated conserved miRNA families, their abundances differed greatly. For example, miR159, miR393 and miR394 were down-regulated by 7.7, 5.3 and 5.5 folds, respectively, in imbibed seeds compared to those in dry seeds. However, their basal expression level in dry seeds were only 2027, 296 and 33, respectively. Whereas miR156 and miR166, although they both were down-regulated in imbibed seed only by 1.5 folds, their basal frequencies in dry seeds were 525,736 and 432,679, respectively, much higher than those of miR159, miR393 and miR394. Even they were significantly down-regulated in the imbibed seeds; there still were 342,738 and 282021 miRNA transcripts of miR156 and miR166, respectively, in the imbibed seeds. The same was true for those up-regulated families. MiR528 and miR408 both were up-regulated by 2.6 folds, but the basal expression frequency of miR528 was 214,196 whereas the basal expression frequency of miR408 was only 2,571.

**Table 4:** Statistics of the expression abundance of the conserved miRNAs from dry and imbibed seeds.

miRNA Family	DS	GS	Ratio (DS/GS)	P value
MIR156	525736	342738	1.5	**
MIR159	2027	264	7.7	**
MIR160	87	97	1	
MIR162	4	8	0.5	
MIR164	38672	15272	2.5	**
MIR166	432679	282021	1.5	**
MIR167	30249	14717	2	**
MIR168	189938	54036	3.5	**
MIR169	5372	2600	2	**
MIR171	137	149	0.9	
MIR172	63	26	2.4	**
MIR319	3092	2430	1.3	**
MIR390	288	240	1.2	
MIR393	296	55	5.3	**
MIR394	33	6	5.5	**
MIR396	434	480	0.9	
MIR397	13076	11626	1.1	**
MIR398	377	652	0.6	**
MIR399	10	4	2.5	
MIR408	2571	7023	0.4	**
MIR528	214196	557872	0.4	**
MIR529	92	140	0.7	**
MIR827	1749	1765	1	
MIR1432	25	38	0.7	

** , Significant values (P< = 0.01); DS, Dry seed; GS, Imbibed seed.

### Novel Maize miRNAs

**Figure 5:**
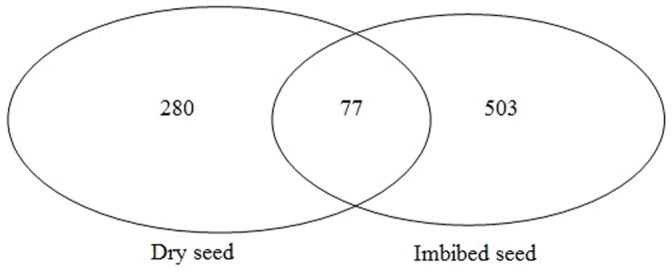
Comparison of novel miRNAs between dry and imbibed seeds.

It is always challenging to identify novel plant miRNAs due to their low level of expression and abundance. Some of the common features of miRNAs have been explored and different miRNA prediction programs have been developed to predict plant novel miRNAs. For example, one of the characteristics of miRNA precursors was their hairpin structures. However, hairpin structure is not unique to pre-miRNA, lots of other coding or non-coding RNAs, such as rRNA, tRNA and mRNA, also have the similar hairpin structures [35]. Several studies observed that miRNA precursors have low folding free energy, and considered that low free energy is one important characteristic of miRNAs [36]. We previously developed a very stringent miRNA prediction program to predict maize novel miRNAs [37]. Briefly, a small RNA is considered as a potential miRNA candidate only if it meets all the following strict criteria: 1) the sequence could fold into an appropriate stemloop hairpin secondary structure, 2) the small RNA sits in one arm of the hairpin structure, 3) no more than 6 mismatches between the predicted mature miRNA sequence and its opposite miRNA* sequence in the secondary structure, 4) no loop or break in the miRNA or miRNA* sequences, and 5) predicted secondary structure has higher minimal folding free energy index and negative minimal folding free energy. Based on the miRNA prediction criteria mentioned above, 357 and 580 novel miRNAs were predicted from dry and imbibed seeds, respectively (Additional File S2, S3, S4). They were mapped to all 10 chromosomes in maize. The lengths of the vast majority of the novel miRNAs were 20, 21 and 22 nt and none of them were 24 nt small RNAs. More than half of the novel miRNAs began with 5' uridine, which was a characteristic feature of miRNAs [35], [36]. The predicted hairpin structures of the miRNA precursors were in the range of 64–371 nt, which was similar to those observed in rice [38]. The average negative minimal folding free energy of these miRNA precursors was −61.15 kcal mol^−1^ according to Mfold3.2, which was similar to the computational values of *Arabidopsis thaliana* miRNA precursors (−57 kcal mol^−1^) and much lower than folding free energies of tRNA (−27.5 kcal mol^−1^) or rRNA (−33 kcal mol^−1^) [36]. Compared to conserved miRNA families, the reads of novel miRNAs were very low, and the majority of them were sequenced less than 50 times. The most abundant novel miRNAs was miRgs297 that was sequenced 3019 times in imbibed seed and novel miRds40 that was 381 times in dry seed. We also looked for sequenced miRNA* sequences, only 46 complementary sequences were found in our combined dry and imbibed seed data sets. Most miRNA* showed weak expression (sequencing frequency <10) and their expression levels were much lower than their corresponding miRNAs, consistent with the idea that miRNA* strands were degraded rapidly during the biogenesis of mature miRNAs [33]. Compared to conserved miRNAs, different set of novel miRNAs was expressed in dry and imbibed seeds. Of the novel miRNA identified, 280 were dry-seed-specific, 503 were imbibed-seed-specific, and only 77 were shared in both dry and imbibed seeds. Large numbers of novel miRNAs were specifically expressed in imbibed seeds (Figure 5).

### Target Prediction of Maize Novel miRNAs and GO Analysis

**Figure 6:**
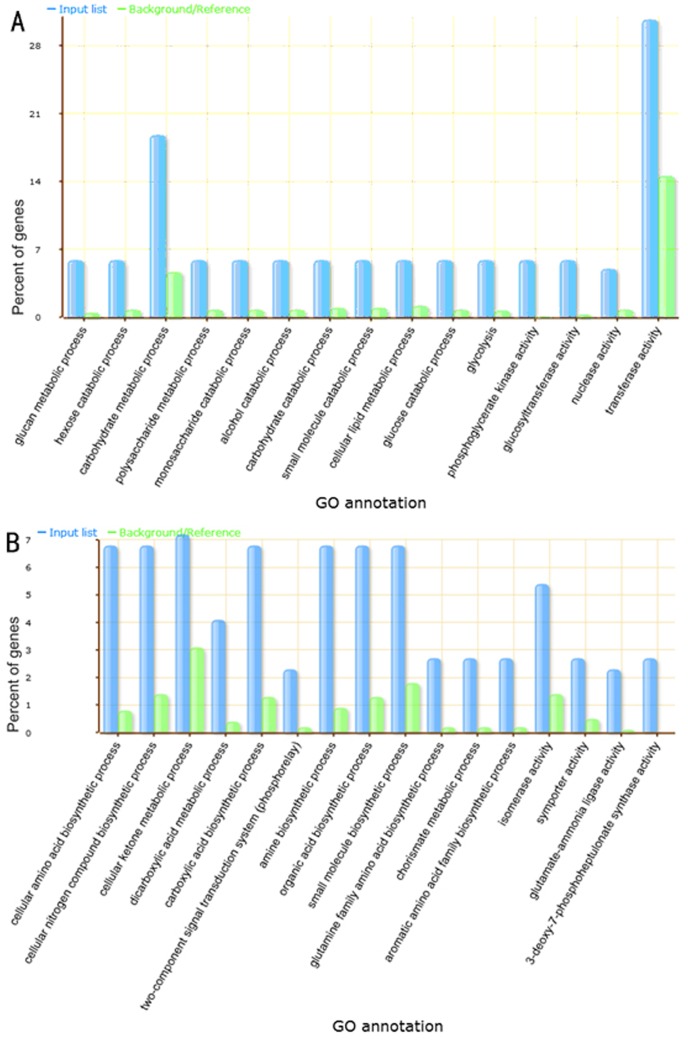
Gene ontology classification of novel miRNA targets in dry seed and imbibed seed. A represents in dry seed, B represents in imbibed seed. The X-axis is the categories of target genes. The Y-axis is the percentage of genes mapped by the categories, and represents the abundance of the GO categaries. AgriGO web-based tool was used to analyze GO categories of genes showing changes in transcription levels.

To assess the function of the novel miRNAs from dry and imbibed maize seeds, putative targets of novel miRNAs were predicted using a stringent bioinformatic program. Targets of 301 novel miRNAs were successfully predicted. No target genes were found for the rest of 559 novel miRNAs. To gain a better understanding of the functional roles of the predicted miRNA target genes in maize, we did Gene Ontology (GO) analysis on putative targets. The targets were annotated by using the GO annotations available from B73 RefGen_v2. GO terms are commonly used to describe the functions of genes and gene products and to facilitate queries among genes from different organisms. Since the expression levels of the majority of the predicted novel miRNAs were low, targets of miRNAs with sequencing frequency greater than 50 reads were GO analysed. There were 65 miRNA families consisting of 99 miRNAs whose reads were more than 50. Figure 6A showed target genes specifically enriched in the dry seed. Novel miRNA targets of carbohydrate catabolic related genes were specifically enriched in dry seed. Figure 6B showed target genes specifically enriched in the imbibed seed. Contrary to the dry seed, novel miRNA targets covered a broad range of functional categories in the imbibed seed. They included genes in amino acid biosynthesis, isomerase activity, ligase activity and others.

### Validation of miRNA and Target Genes

**Figure 7:**
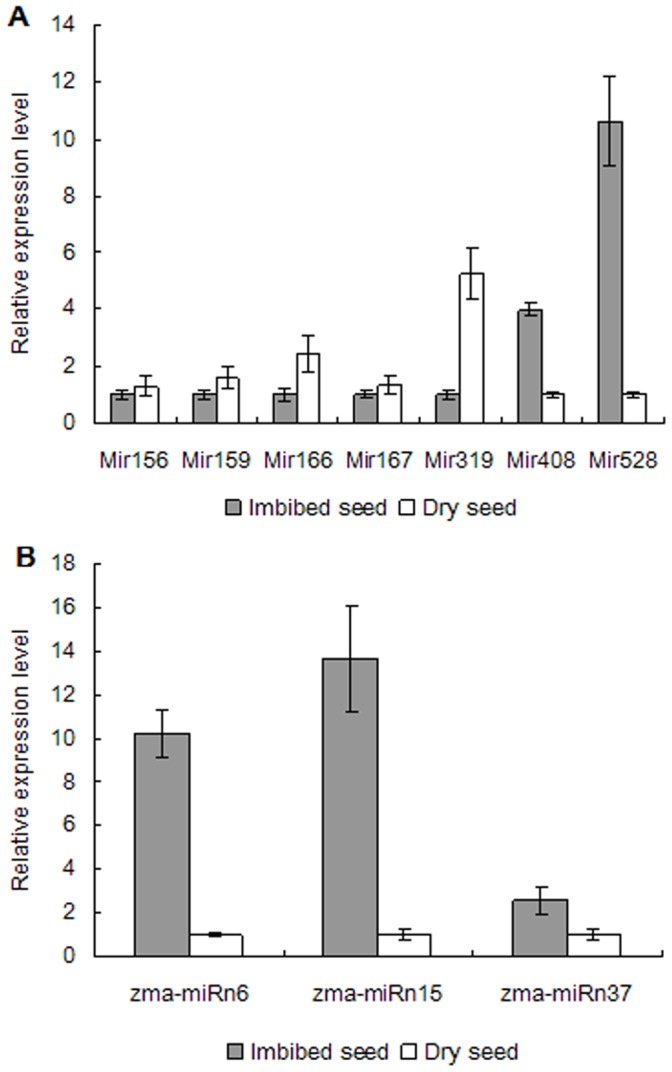
Quantitative RT-PCR validation of mature miRNAs in dry and imbibed seeds. These expression profiles of mature miRNAs in dry and imbibed seeds were similar to the results obtained by small RNA deep sequencing. The expression values presented were means of four technical replicates ± SD. U6 was used for each sample as an endogenous control. A and B represented the expression pattern of mature conserved and novel miRNAs, respectively.

**Figure 8:**
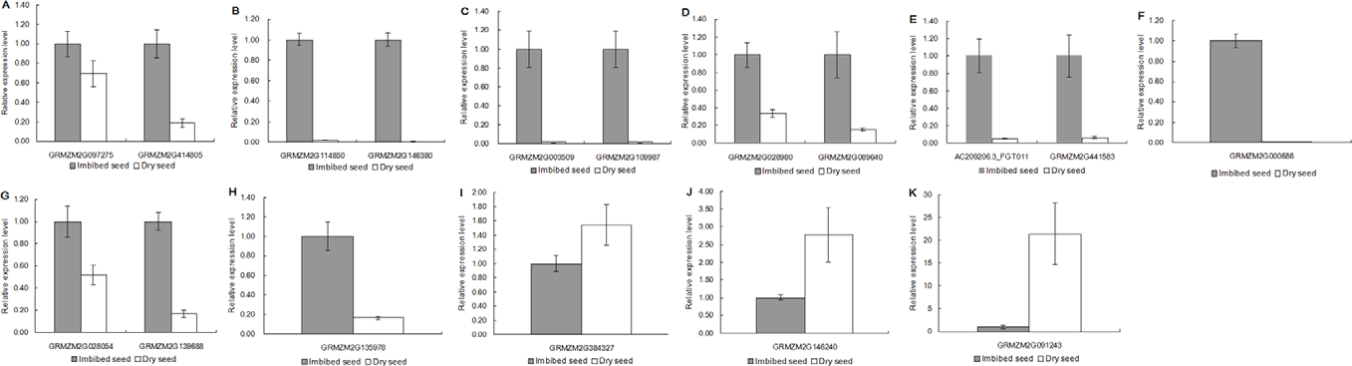
Quantitative RT-PCR analyses of the relative expression levels of various predicted target genes. The maize housekeeping gene actin was used as the internal control. The values presented were means of three technical replicates ± SD. A-K represented the expression profiles of some predicted target genes of miR156, miR164, miR166, miR167, miR168, miR169, miR319, miR393, miR408, miR528 and zma-miRn6 in dry and imbibed seeds, respectively.

**Figure 9:**
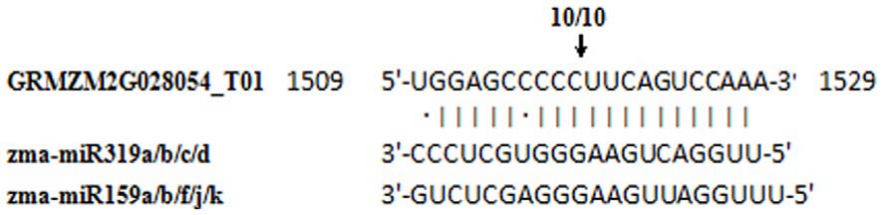
Zma-miR319/miR159 cleavage site in GRMZM2G028054_T01 mRNAs determined by 5'-RLM-RACE. The vertical lines represent the nucleotides that base-pair with GRMZM2G028054_T01 and dots show the mismatched nucleotide. Arrow indicates the 5'termini of mRNA fragments and the frequency of 5'-RACE clones corresponding to cleavage side.

To validate conserved miRNAs identified and novel ones predicted, quantitative RT-PCR (qRT-PCR) was performed on 10 randomly selected miRNAs, miR156, miR159, miR166, miR167, miR319, miR408, miR528, zma-miRn6, zma-miRn15 and zma-miRn37 in dry and imbibed seed. qRT-PCR was also performed on target genes of 17 randomly selected miRNA targets. These 17 targets were targets of 11 miRNA families (miR156, miR164, miR166, miR167, miR168, miR169, miR319, miR393, miR408, miR528 and zma-miRn6). Figure 7 showed that the 10 miRNA expression profiles by qRT-PCR were consistent with those by deep sequencing. Figure 8 showed that the 17 target gene expression profiles were opposite to those of their corresponding miRNA that cleave them. GRMZM2G028054, a member of MYB family, was target gene of both miR159 and miR319. The cleavage site was validated by 5'-RLM-RACE (Figure 9).

## Discussion

Small RNA libraries from maize dry and imbibed seed embryos were sequenced in this study. The two libraries generated a total of 25,797,782 clean reads and 85.58% of them were perfectly matched to the B73 genome, indicating the overall good quality of the sequence data. The total clean reads consisted of 3,454,632 unique small RNA reads. These sequencing data suggested a highly complex small RNA population in maize seed. The two libraries shared 75.89% of the total clean reads; they shared, however, only 14.33% of the unique small RNA reads. The above results indicated that the shared reads have less small RNA members with each member, on average, having high expression level; while the dry-seed-specific and imbibed-seed-specific small RNAs have more small RNA members with each member having low expression level. The low expression level of these specific unique small RNAs suggested the possibility that they acted upstream of the complex regulating pathway. As to the unique small RNA reads, 18.55% were dry-seed-specific and 67.12% were imbibed-seed-specific, indicated that more unique small RNAs were expressed in imbibed seed. The result reflected the fact that complicated physiological, biochemical and molecular changes took place and diverse small RNAs were needed to regulate gene expression in imbibed seed.

MicroRNAs are one of the major players of the total small RNA population in regulating gene expression. Twenty-four conserved miRNA families were identified and 860 putative novel miRNAs were predicted in this study. Sixteen conserved miRNA families showed significant expression differences between dry and imbibed seeds with 12 families down-regulated and 4 families up-regulated in imbibed seeds. Once seeds imbibe water, germination begins. Plant hormones, especially the balance between abscisic acid (ABA) and gibberellin acid (GA), play important roles in seed development, maturation, dormancy and germination. ABA is abundant in dormant seeds and generally decreases during imbibition when seed dormancy is released, whereas GA increases during the transition to germination [17]. The endogenous ABA contents in dry seeds rapidly decline upon imbibition during the early phase of germination (within 6–12 hours) [39]. Many ABA signal transduction proteins were involved in seed germination [40]–[43]. In *Arabidopsis*, miR159, targeting MYB33 and MYB101, two ABA positive regulators, plays a vital role in seed germination [19]. In this study, GRMZM2G423833 and GRMZM2G070523, the homologs of AtMYB33 and AtMYB101, were predicted as target genes of MiR159 and GRMZM2G070523 was validated by degradome sequencing (data not shown). MiR159 and miR319 showed high similarity in mature miRNA sequences, their target motifs were highly conserved in cereal and *Arabidopsis* GAMYB genes, and they often shared same target genes. RMZM2G139688 and GRMZM2G028054 were predicted as target genes of both miR159 and miR319 in this study. GRMZM2G139688 was identified in our degradome dataset (data not shown). GRMZM2G028054 was validated as target gene of miR159/319 by 5′ RACE cleavage assay (Figure 9). Their expression levels were about 6 and 2 fold changes in imbibed seed by qRT-PCR, respectively (Figure 8). These two target genes were members of the MYB transcription factor family and were homologous to AtMYB65 in *Arabidopsis*. AtMYB33, AtMYB65 and AtMYB101 were members of GAMYB-like family and were post-transcriptionally regulated by miR159 [44]. In the absence of miR159, the deregulation of MYB33 and MYB65 up-regulated genes that were GA induced during seed germination. These genes participated in aleurone vacuolation which was GA-mediated programmed cell death (PCD) required for seed germination [45]. Thus, GAMYB-like genes participated in GA-induced pathways via miR159-mediated regulation during seed germination. The predicted target gene of miR169 was nuclear factor YA. Nuclear factor Y (NF-Y) was a highly conserved transcription factor presented in all eukaryotic organisms, and was a heterotrimer consisting of three subunits (NF-YA, NF-YB and NF-YC) [46]. In our degredome dataset, NF-YA5 was the most abundance target gene of miR169. In *Arabidopsis*, the expression of NF-YA5 was regulated by miR169 and overexpression of NF-YA5 caused hypersensitivity to ABA during seed germination [47], [48]. In rice, a *cis*-acting ABA responsive element (ABRE) was found in the upstream region of miR169n suggesting that it may be ABA regulated [49]. Upon seed germination, ABA level decreases, so are the abundances of miR159, mi169 and miR319. In this report, MiR159, miR169 and miR319 were all down-regulated with miR159 being down-regulated by 7.7 folds, the most down-regulated miRNA family in imbibed seed. When ABA level decreased, the expression of miR159 would be decreased. Its targets, MYB33 and MYB101, the two ABA positive regulators, would be up-regulated, which, in turn, would increase the level of ABA in imbibed seed. This is somewhat counter-intuitive. However, it was thought that this pathway played a pivotal role in resetting ABA responses in order for seeds to sense a decrease in ABA level. Probably, cells need to degrade positive factors of ABA signalling to reset the developmental program. Thus, miR159 may function in allowing a fast recovery from high ABA levels by regulating the genes of positive factors continually when the signal disappears [19]. This negative feedback of ABA signalling by miR159 may be important for seed in the shift from dormancy to germination [11].

MiR164, miR167 and miR393 were also significantly decreased in maize imbibed seed. Their predicted target genes were mainly involved in auxin signal transduction pathway and downstream transcription factors. In *Arabidopsis*, miR393 has been shown function via the auxin pathway by post-transcriptional regulation of auxin receptorsTIR1, AFB2 and AFB3 [50]. In rice, OsmiR393 was recently found to play an important role in response to salt and drought stress by targeting two rice auxin receptor genes OsTIR1 and OsAFB2 [51]. AUXIN RESPONSE FACTOR (ARF)s are the executors of auxin-dependent transcription and form the pivotal point in translating auxin signals into transcriptional responses [52]. MiR167 regulate ARF6 and ARF8 as positive regulators of adventitious rooting in *Arabidopsis* [53]. In cultured rice cells, miR167 was shown to cleave auxin responsive factor 8 (ARF8) mRNA [54]. MiR164 has also been shown to play a role in plant development. In *Arabidopsis*, miR164 targets transcription factor NAC1 to down-regulate auxin signals for *Arabidopsis* lateral root development [55]. We proposed that the down-regulation of miR393, miR167 and miR164 in imbibed seed might play important roles in dry-to-germinating seed transition by regulating auxin perception, transduction and function.

**Figure 10:**
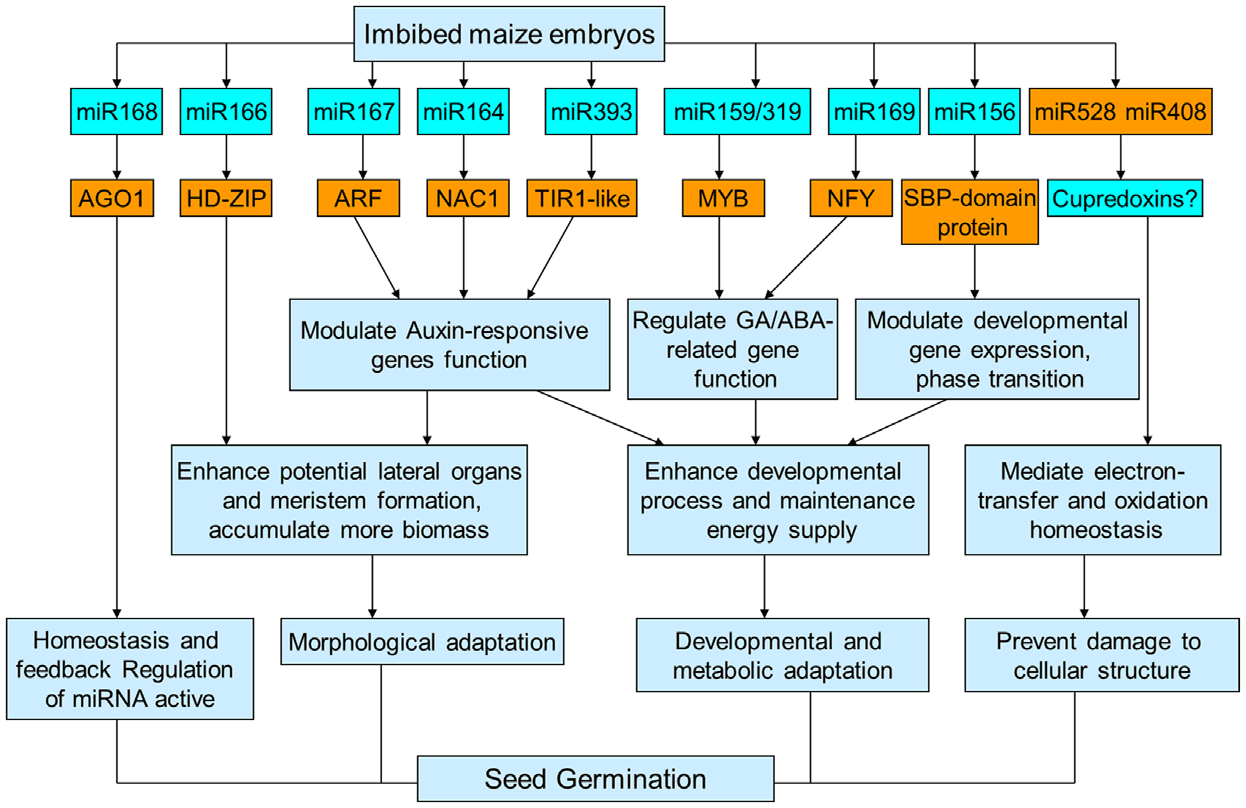
The potential regulatory network for miRNAs in maize dry and imbibed seed. The blue boxes represent down-regulated miRNA/genes and the orange boxes represent up-regulated miRNA/genes.

Plants undergo several developmental transitions, including the transition from an embryonic to post-embryonic mode of growth, the juvenile-to-adult vegetative transition, and the vegetative-to-reproductive transition. The juvenile phase is an important and critical step during plant development to ensure maximum growth and productivity. MiR156 plays crucial role in the control of juvenile-to-adult transition in plant by targeting the SQUAMOSA PROMOTER BINDING PROTEIN LIKE (SPL) plant-specific transcription factors [56]. SPLs affect diverse developmental processes such as leaf development, shoot maturation, phase change and flowering in plants. In *Arabidopsis thaliana*, there are 17 members of the SPL family of transcription factors, and 11 of them are the MiR156 targets [57]. The rice genome contains 19 SPL genes, with 11 of those SPLs containing the target sites of OsmiR156. Overexpression of miR156 in *Arabidopsis*, rice and maize, repressed the transcript abundance of related SPL genes and reduced apical dominance, delayed flowering time, caused dwarfism and increased total leaf numbers and biomass [58], [57], [23]. Teosinte glume architecture1 (*tga*1), which encodes an SBP-domain family protein as does *Arabidopsis* SPL13 and plays an important role in maize domestication, has been identified as a target of miR156 [25]. In maize, *Corngrass1* (*Cg1*) mutants overexpress two tandem miR156 genes [23], which target *teosinte glume architecture1* (*tga1*). The reduction of *tga*1 expression in *Cg*1 mutants affects the juvenile to adult transition, an important phase transition in plant development [25]. It was reported that 7 out of 8 switchgrass SPL genes exhibited higher transcript abundance in inflorescences than in other tissues at reproductive stage [59]. A decline in miR156 abundance provides a permissive environment for flowering and is paralleled by a rise in SPL levels [60]. MiR156 seems to play the same role in the dry seed (embryonic mode) to the imbibed seed (post-embryonic mode) transition as that in the juvenile to adult transition. MiR156 showed the highest abundance (525736) in dry seed, possibly keeping SPL factors low to prevent germination. After 24 hours of imbibition, the transcript was reduced to 342738, a 1.5 fold reduction. The balance between miR156 and SPL appears to be important to maintain proper plant development and phase transition. Previous studies showed that miRNA gene regulation cascades exist and the miR156 pathway acts upstream of the miR172 pathway. Moreover, targets of miR156 and miR172 exert positive feedback on the expression of miRNA genes that suppress themselves [61]. Compared to dry seed, miR172 also was significantly reduced in imbibed seed (by 2.4 fold). MiR166 is the second most abundant miRNA in the dry seed and showed similar reduction in imbibed seed (from 432679 to 282021, by 1.5 fold). Previous studies indicated that miR166 regulated plant leaf morphogenesis, vascular development as well as lateral organ polarity and meristem formation, by targeting class III HOMEODOMAIN-LEUCINE ZIPPER (HD-ZIP III) transcription factors [62], [63]. In dry seed, abundant miR166 also is needed to keep low activities of HD-ZIP III transcription factors. Not only the miRNA156 and miRNA166 families were abundant in dry and imbibed seeds, but also they had more family members than any other miRNA families, suggesting the importance of these two miRNA families in dry and imbibed seeds. MiR168 also was significantly decreased in imbibed seed. In *Arabidopsis*, exclusive target of miR168 is AGO1, which is the core component of the RNA-induced silencing complex (RISC) [64]. AGO1 also was predicted as the target of zma-miR 168a/b in maize root [65]. RISC associates with miRNA and inhibits target genes by mRNA cleavage or translational repression [66]–[68]. It is well documented that miRNAs play crucial roles in controlling a variety of developmental processes such as organ identity establishment, organ separation, hormone signalling, flowering time control and regulating plant stress response. ARGONAUTE (AGO) proteins are considered to be integral players in all known small RNA-targeted regulatory pathways [67]. The miR168-mediated feedback regulatory loop regulates ARGONAUTE1 (AGO1) homeostasis, which is crucial for gene expression modulation and plant development. MiR528 was up-regulated upon imbibition and also was the most abundant miRNAs in the imbibed seed. MiR528 was found to be repressed in response to drought stress in leaves of *Triticum dicoccoides* [69]. Rice miR528 had been shown to be down-regulated during the early submergence phase and induced after 24 h of submergence in maize roots [70]. There was a stable strong repression of miR528 in maize both roots and shoots under the low N condition [71]. Copper proteins, cupredoxin, multicopper oxidase and laccase genes have been predicted as targets of miR528, only Cu^2+^-binding domain-containing protein (CBP) was experimentally validated in sugarcane [72]. MiR528 was not found in *Arabidopsis* and believed to be monocot-specific. MiR408 also was up-regulated in imbibed seed. The *Arabidopsis* gene family of MiR408 has a single member, regulate a subset of the laccase gene family (AtLAC) and plantacyanin transcripts [73], [74]. Both LACs and plantacyanin are Cu-containing proteins. Plant laccases play a putative role in lignin biosynthesis [75], while plantacyanin functions in reproduction of *Arabidopsis* [76]. It is widely accepted that during seed germination, reactive oxygen species (ROS) such as superoxide radicals, hydroxyl radicals and hydrogen peroxide (H_2_O_2_) are produced in excess. ROS are efficiently scavenged by superoxide dismutase (SOD) [77] to minimize cell damage and to promote high-germination capacity and vigorous seedling development. MiR408 and miR528, whose potential target genes are predominantly involved in energy metabolism and scavenging of the oxidative species produced during stress. The distinct roles of miR528 and miR408 in seed germination need further investigation. Based on the information discussed above, we proposed the possible roles of miRNA playing in the very early stage of germination [Figure 10].

Novel miRNAs were predicted using a very stringent bioinformatic program and the majority of them showed low level of expression. We chose those that showed more than 50 reads in the normalized libraries for target GO analysis. It was very interesting to notice that carbohydrate catabolic related genes were specifically enriched in the dry seed library, indicating that dry-seed-specific miRNAs supress carbohydrate catabolic related genes to keep dry seed in the dry state. Or it may reflect the fact that dry seeds contain mRNAs stored during maturation (also called long-lived transcripts) to indicate they survived desiccation [78]. Over 10,000 different stored mRNAs have been identified in transcriptome analysis of *Arabidopsis* [79]–[82]. Similar numbers were found in barley and rice [83], [84]. During maize seed maturation, carbohydrates are accumulated for storage and it is not surprising to notice that carbohydrate catabolic related genes are specifically enriched in dry seed. However, target gene enrichment in imbibed seed was totally different from that in dry seed. They covered a broad range of functional categories including genes in amino acid biosynthesis, isomerase activity, ligase activity and others. This may suggest that low expression of novel imbibed seed specific miRNAs regulate transcription, amino acid biosynthesis and translation at the proper speed.

## Methods

### RNA Isolation and Cloning of Maize Small RNAs

Maize (*Zea mays*) inbred line 87-1 was used in this study. Dry seeds were soaked in distilled water, wrapped in paper towels and incubated at 25°C for 24 hours in dark. Embryos of imbibed seeds were used for RNA extraction. Embryos of dry seeds without soaking were used as control. Briefly, total RNAs were extracted from the two samples using Trizol kit (Invitrogen, USA). The small RNAs of 16–28 nt were gel-purified by poly-ethylene glycol precipitation, separated on 15% denaturing PAGE, and visualized by SYBR-gold staining. Small RNAs were ligated to a 5′adaptor and a 3′adaptor sequentially, reverse-transcription polymerase chain reaction (RT-PCR) amplified, and PCR products were subjected to sequencing [85]. The sequencing was performed on the Illumina platform (Beijing Genomics Institute, China).

### Identification of Conserved and Novel miRNAs

The raw sequences and vector removal were processed using PHRED and CROSS MATCH programs as previously reported [86], [87]. Trimmed sequences with >18 nt were used for further analyses. First, known non-coding RNAs including rRNA, tRNA, snRNA, snoRNA, and those containing the polyA tail, were removed from the small RNA sequences and the remaining sequences were mapped to the maize ncRNAs deposited in the NCBI GenBank database and Rfam database. Then, the unique small RNA sequences were analyzed by BLAST search against the miRNA database (miRBase 17.0). Only mature and precursor sequences of those small RNAs perfectly matched to known maize miRNAs, were regarded as conserved miRNAs.

To discover potential novel miRNA precursor sequences in our dataset, we used the identified mature miRNA sequences to do Blastn searches against maize genomic sequence. Sequences that met previously described criteria were then considered to be miRNA precursors [88]. A maximum of six unpaired nucleotides and the distance ranging from 5 to 240 nt between the miRNA and miRNA* were allowed. The selected sequences were then folded into a secondary structure using an RNA-folding program mFold3.2 [89] (see Additional File S5, S6, S7). Specifically, dominant, mature sequences residing in the stem region of the stem-loop structure and ranging between 20–22 nt with a maximum free-folding energy of −20 kcal mol^−1^ were considered as potential novel maize miRNA candidates.

### Target Gene Prediction and Analysis

The major steps and parameter settings for predicting target genes of miRNAs were performed as described in previous studies [90], [91], [58]. Briefly, the criteria were as follows: 1) No more than four mismatches between miRNA and target (G-U bases count as 0.5 mismatches), 2) No more than two adjacent mismatches in the miRNA/target duplex, 3) No adjacent mismatches in positions 2–12 of the miRNA/target duplex (5' of miRNA), 4) No mismatches in positions 10–11 of miRNA/target duplex, 5) No more than 2.5 mismatches in positions 1–12 of the miRNA/target duplex (5' of miRNA). MiRNA target genes were predicted follow the criteria for the identified novel miRNAs (see Additional File S8, S9, S10). These targets were grouped by the biological function of the proteins they encode for, as described by UniProt (http://www.uniprot.org/). The agriGO web service (http://bioinfo.cau.edu.cn/agriGO/analysis.php) was used for the gene ontology term enrichment test [92].

### Quantitative RT-PCR for Mature miRNAs

Small RNAs were isolated using the miRcute miRNA isolation kit (Tiangen, Beijing, China) from dry and imbibed maize seed embryos. Then a polyA adapter was ligated to the mature miRNAs 3' end by *E.coli* poly(A) polymerase, and the ligated products were used for the initiation of the reverse transcription according to the supplier’s manual of the miRcute miRNA first-strand cDNA synthesis kit (Tiangen, Beijing, China). The reverse transcription product was amplified using a miRNA-specific forward primer and a universal reverse primer.

The specific primers for quantitative RT-PCR (qRT-PCR) on the mature miRNAs were designed with the software primer premier 5.0 (PREMIER Biosoft Int., Palo Alto, CA, USA) (Additional File S11). For mature miRNAs, qRT-PCR with SYBR Green was performed on a CFX96™ Real-Time System (Bio-Rad, Hercules, California, USA). Briefly, 20 ul PCR reaction contained about 5 ng miRNA first-strand cDNA synthesis, 10 ul 2× miRcute miRNA premix, 200 nM each primer. The reactions were mixed gently and incubated at 94°C for 2 min, followed by 45 cycles of 94°C for 15 s, 61.5°C for 30 s. Finally, a final stage of 65°C to 95°C was performed to confirm the absence of multiple products and primer dimers. U6 was used for each sample as an endogenous control. All samples were performed at least three technical replicates. Data were analyzed using Bio-Rad cfx manager software (Bio-Rad, Hercules, California, USA).

### Validation of the miRNA Predicted Target Gene Expression Profiles by Quantitative RT-PCR

Total RNA was isolated from dry and imbibed seed embryos (24 hours imbibed) using Trizol kit respectively (Invitrogen, USA). The total RNA (about 2 ug) was reverse transcribed using RevertAid ™ First Strand cDNA Synthesis Kit (Fermentas, EU).

Potential target genes of conserved miRNA were identified in maize according to Zhang et al [93]. The predicted target genes were obtained from the Maize Sequence database. The specific primers for quantitative RT-PCR were designed with the software primer premier 5.0 (PREMIER Biosoft Int., Palo Alto, CA, USA) (Additional File S12). qRT-PCR with SYBR Green was performed on a CFX96™ Real-Time System (Bio-Rad, Hercules, California, USA). Briefly, 20 ul PCR reaction contained about 100 ng cDNA, 9 ul 2.5× RealMasterMix/20× SYBR solution, 200 nM each primer. The reactions were mixed gently and incubated at 94°C for 3 min, followed by 45 cycles of 94°C for 10 s, 57°C for 20 s and 68°C for 20 s. Finally, a final stage of 65°C to 95°C was performed to confirm the absence of multiple products and primer dimers. Actin (GRMZM2G126010_T01) was used for each sample as an endogenous control. All samples were performed at least three technical replicates. Data were analyzed using Bio-Rad cfx manager software (Bio-Rad, Hercules, California, USA).

### 5'-RLM-RACE Validation of miRNA Targets

RNA ligase-mediated 5'-rapid amplification of cDNA ends (RLM 5'-RACE) was performed using the FirstChoice RLM-RACE Kit (Ambion, USA). The total RNA was extracted from the embryos of imbibed seeds (87-1). An RNA adapter was directly ligated to 3 ug of total RNA using a T4 RNA ligase. Ligated mRNAs were reverse transcribed using RevertAid ™ First Strand cDNA Synthesis Kit (Fermentas, EU) according to the manufacturer’s instructions. Two gene-specific primers (GSP and NGSP) were designed by Primer Premier 5.0 software (Additional File S13). These two primers were used for two rounds of PCR. Initial PCR reactions were done with the 5'RACE outer primer and complementary GSP. The nested PCR was performed with the 5'RACE inner primer and the NGSP and the nested PCR products were analyzed on an agarose gel. Positive PCR products were cloned into pEASY-T1 (TransGen) vector by use of pEASY-T1 Cloning Kit (TransGen) and sequenced.

## Supporting Information

Additional File S1 **Conserved miRNAs in maize.** These miRNA are conserved in maize and have been reported in miRBase. DS, dry seed; GS: imbibed seed, ‘+’: present in our dataset; ‘−’, absent in our dataset; L, length; **, miRNA sequences of maize were identical to those in other species; *, maize miRNA sequences were conserved in other species but have variations in some nucleotide positions.
(XLS)

Additional File S2 **Novel miRNAs identified in common in this study.**
(XLS)

Additional File S3 **Novel miRNAs only identified in dry seed in this study.**
(XLS)

Additional File S4 **Novel miRNAs identified in imbibed seed in this study.**
(XLS)

Additional File S5 **Secondary structures of putative novel miRNA identified in dry and imbibed seeds in this study.**
(DOC)

Additional File S6 **Secondary structures of putative novel miRNA only identified in dry seed in this study.**
(DOC)

Additional File S7 **Secondary structures of putative novel miRNA only identified in imbibed seed in this study.**
(DOC)

Additional File S8 **Predicted targets for novel miRNAs identified in common in this study.**
(XLS)

Additional File S9 **Predicted targets for novel miRNAs only identified in dry seed in this study.**
(XLS)

Additional File S10 **Predicted targets for novel miRNAs only identified in imbibed seed in this study.**
(XLS)

Additional File S11 **Primers used for mature miRNAs qRT-PCR.**
(XLS)

Additional File S12 **Primers used for predicted miRNAs targets qRT-PCR.**
(XLS)

Additional File S13 **5'RACE validation of the miRNA targets nested PCR primers.**
(XLS)

## References

[1] Phillips JR, Dalmay T, Bartels D (2007) The role of small RNAs in abiotic stress. FEBS Letter 581: 3592–3597. 10.1016/j.febslet.2007.04.007 17451688

[2] Lu C, Tej SS, Luo S, Haudenschild CD, Meyers BC, et al (2005) Elucidation of the small RNA component of the transcriptome. Science 309: 1567–1569. 16141074 10.1126/science.1114112

[3] Jones-Rhoades MW, Bartel DP, Bartel B (2006) MicroRNAs and their regulatory roles in plants. Annual Review of Plant Biology 57: 19–53. 10.1146/annurev.arplant.57.032905.105218 16669754

[4] Lee RC, Feinbaum RL, Ambros V (1993) The C. elegans heterochronic gene lin-4 encodes small RNAs with antisense complementarity to lin-14. Cell 75: 843–854. 8252621 10.1016/0092-8674(93)90529-y

[5] Xie Z, Kasschau KD, Carrington JC (2003) Negative feedback regulation of Dicer-Like1 in Arabidopsis by microRNA-guided mRNA degradation. Current Biology 13: 784–789. 12725739 10.1016/s0960-9822(03)00281-1

[6] Brodersen P, Sakvarelidze-Achard L, Bruun-Rasmussen M, Dunoyer P, Yamamoto YY, et al (2008) Widespread translational inhibition by plant miRNAs and siRNAs. Science 320: 1185–1190. 18483398 10.1126/science.1159151

[7] Khraiwesh B, Arif MA, Seumel GI, Ossowski S, Weigel D, et al (2010) Transcriptional control of gene expression by microRNAs. Cell 140: 111–122. 20085706 10.1016/j.cell.2009.12.023

[8] Mallory AC, Vaucheret H (2006) Functions of microRNAs and related small RNAs in plants. Nature Genetics 38: S31–S36. 16736022 10.1038/ng1791

[9] Rhoades MW, Reinhart BJ, Lim LP, Burge CB, Bartel B, et al (2002) Prediction of plant microRNA targets. Cell 110: 513–520. 12202040 10.1016/s0092-8674(02)00863-2

[10] Mitsuda N, Ohme-Takagi M (2009) Functional Analysis of Transcription Factors in Arabidopsis. Plant and Cell Physiology 50: 1232–1248. 19478073 10.1093/pcp/pcp075 PMC2709548

[11] Nonogaki H (2010) MicroRNA gene regulation cascades during early stages of plant development. Plant Cell Physiology 51(11): 1840–1846. 20937608 10.1093/pcp/pcq154

[12] Martin RC, Liu PP, Nonogaki H (2005) Simple purification of small RNAs from seeds and efficient detection of multiple microRNAs expressed in Arabidopsis thaliana and tomato (Lycopersicon esculentum) seeds. Seed Science Research 15: pp319–328.

[13] Martin RC, Liu PP, Nonogaki H (2006) microRNAs in seeds: modified detection techniques and potential applications. Canadian Journal of Botany 84: 189–198.

[14] Martin RC, Asahina M, Liu PP, Kristof JR, Coppersmith JL, et al (2010) The regulation of post-germinative transition from the cotyledon to vegetative-leaf stages by microRNA-targeted SQUAMOSA PROMOTER-BINDING PROTEIN LIKE13 in Arabidopsis. Seed Science Research 20: 89–96.

[15] Martin RC, Asahina M, Liu PP, Kristof JR, Coppersmith JL, et al (2010) The microRNA156 and microRNA172 gene regulation cascades at post-germinative stages in Arabidopsis. Seed Science Research 20: 79–87.

[16] Liu PP, Montgomery TA, Fahlgren N, Kasschau KD, Nonogaki H, et al (2007) Repression of AUXIN RESPONSE FACTOR10 by microRNA160 is critical for seed germination and post-germination stages. The Plant Journal 52: 133–146. 17672844 10.1111/j.1365-313X.2007.03218.x

[17] Kushiro T, Okamoto M, Nakabayashi K, Yamagishi K, Kitamura S, et al (2004) The Arabidopsis cytochrome P450 CYP707A encodes ABA 8'-hydroxylases: key enzymes in ABA catabolism. EMBO Journal 23: 1647–1656. 15044947 10.1038/sj.emboj.7600121 PMC391058

[18] Yamaguchi S, Smith MW, Brown RGS, Kamiya Y, Sun T-P (1998) Phytochrome regulation and differential expression of gibberellin 3b-hydroxylase genes in germinating Arabidopsis seeds. The Plant Cell 10: 2115–2126. 9836749 10.1105/tpc.10.12.2115 PMC143973

[19] Reyes JL, Chua NH (2007) ABA induction of miR159 controls transcript levels of two MYB factors during Arabidopsis seed germination. The Plant Journal 49: 592–606. 17217461 10.1111/j.1365-313X.2006.02980.x

[20] Fahlgren N, Howell MD, Kasschau KD, Chapman EJ, Sullivan CM, et al (2007) High-throughput sequencing of Arabidopsis microRNAs: evidence for frequent birth and death of MIRNA genes. PLoS ONE 2(2): e219. 17299599 10.1371/journal.pone.0000219 PMC1790633

[21] Yao Y, Guo G, Ni Z, Sunkar R, Du J, et al (2007) Cloning and characterization of microRNAs from wheat (Triticum aestivum L.). Genome Biology 8(6): R96. 17543110 10.1186/gb-2007-8-6-r96 PMC2394755

[22] Moxon S, Jing R, Szittya G, Schwach F, Rusholme Pilcher RL, et al (2008) Deep sequencing of tomato short RNAs identifies microRNAs targeting genes involved in fruit ripening. Genome Res. 18(10): 1602–1609. 10.1101/gr.080127.108 PMC2556272 18653800

[23] Chuck G, Cigan AM, Saeteurn K, Hake S (2007) The heterochronic maize mutant Corngrass1 results from overexpression of a tandem microRNA. Nature Genetics 39: 544–549. 17369828 10.1038/ng2001

[24] Lauter N, Kampani A, Carlson S, Goebel M, Moose SP (2005) microRNA172 down-regulates glossy15 to promote vegetative phase change in maize. Proceedings of the National Academy of Sciences of the United States of America 102: 9412–9417. 15958531 10.1073/pnas.0503927102 PMC1166634

[25] Wang H, Nussbaum-Wagler T, Li BL, Zhao Q, Vigouroux Y, et al (2005) The origin of the naked grains of maize. Nature 436: 714–719. 16079849 10.1038/nature03863 PMC1464477

[26] Juarez MT, Kui JS, Thomas J, Heller BA, Timmermans MC (2004) microRNA-mediated repression of rolled leaf1 specifies maize leaf polarity. Nature 428: 84–88. 14999285 10.1038/nature02363

[27] Preston J, Tatematsu K, Kanno Y, Hobo T, Kimura M, et al (2009) Temporal expression patterns of hormone metabolism genes during imbibition of Arabidopsis thaliana seeds: a comparative study on dormant and non-dormant accessions. Plant Cell Physiol 50: 1786–1800. 19713425 10.1093/pcp/pcp121

[28] Martin RC, Liu PP, Goloviznina NA, Nonogaki H (2010) microRNA, seeds, and Darwin? : diverse function of miRNA in seed biology and plant responses to stress. Journal of Experimental Botany 61: 2229–2234. 20335408 10.1093/jxb/erq063

[29] Jiao YP, Song WB, Zhang M, Lai JS (2011) Identification of novel maize miRNAs by measuring the precision of precursor processing. BMC Plant Biol 11: 141. 22014170 10.1186/1471-2229-11-141 PMC3214924

[30] Szittya G, Moxon S, Santos DM, Jing R, Fevereiro MPS, et al (2008) High-throughput sequencing of Medicago truncatula short RNAs identifies eight new miRNA families. BMC Genomics 9: 593. 19068109 10.1186/1471-2164-9-593 PMC2621214

[31] Morin RD, Aksay G, Dolgosheina E, Ebhardt HA, Magrini V, et al (2008) Comparative analysis of the small RNA transcriptomes of Pinus contorta and Oryza sativa. Genome Research 18: 571–584. 18323537 10.1101/gr.6897308 PMC2279245

[32] Zhao CZ, Xiao H, Frazier TP, Yao YY, Bi YP, et al (2010) Deep sequencing identifies novel and conserved microRNAs in peanuts (Arachis hypogaea L.). BMC Plant Biology 10: 3. 20047695 10.1186/1471-2229-10-3 PMC2826338

[33] Rajagopalan R, Vaucheret H, Trejo J, Bartel DP (2006) A diverse and evolutionarily fluid set of microRNAs in Arabidopsis thaliana. Genes and Development 20: 3407–3425. 17182867 10.1101/gad.1476406 PMC1698448

[34] Kozomara A, Griffiths-Jones S (2011) miRBase: integrating microRNA annotation and deep-sequencing data. Nucleic Acids Research 39: D152–D157. 21037258 10.1093/nar/gkq1027 PMC3013655

[35] Zhang BH, Pan XP, Cox SB, Cobb GP, Anderson TA (2006) Evidence that miRNAs are different from other RNAs. Cellular and Molecular Life Sciences 63: 246–254. 16395542 10.1007/s00018-005-5467-7 PMC11136112

[36] Bonnet E, Wuyts J, Rouze P, Peer VY de (2004) Evidence that microRNA precursors, unlike other non-coding RNAs, have lower folding free energies than random sequences. Bioinformatics 20: 2911–2917. 15217813 10.1093/bioinformatics/bth374

[37] Wang LW, Liu HH, Li DT, Chen HB (2011) Identification and characterization of maize microRNAs involved in the very early stage of seed germination. BMC Genomics 12: 154. 21414237 10.1186/1471-2164-12-154 PMC3066126

[38] Zhu QH, Spriggs A, Matthew L, Fan LJ, Kennedy G, et al (2008) A diverse set of microRNAs and microRNA-like small RNAs in developing rice grains. Genome Research 18: 1456–1465. 18687877 10.1101/gr.075572.107 PMC2527712

[39] Chiwocha SDS, Cutler AJ, Abrams SR, Ambrose SJ, Yang J, et al (2005) The etr1–2 mutation in Arabidopsis thaliana affects the abscisic acid, auxin, cytokinin and gibberellin metabolic pathways during maintenance of seed dormancy, moist-chilling and germination. Plant J 42: 35–48. 15773852 10.1111/j.1365-313X.2005.02359.x

[40] Finkelstein R, Reeves W, Ariizumi T, Steber C (2008) Molecular aspects of seed dormancy. Annual Review of Plant Biology 59: 387–415. 10.1146/annurev.arplant.59.032607.092740 18257711

[41] Nakashima K, Fujita Y, Kanamori N, Katagiri T, Umezawa T, et al (2009) Three Arabidopsis SnRK2 protein kinases, SRK2D/SnRK2.2, SRK2E/SnRK2.6/OST1 and SRK2I/SnRK2.3, involved in ABA signaling are essential for the control of seed development and dormancy. Plant Cell Physiol 50: 1345–1363. 19541597 10.1093/pcp/pcp083

[42] Yamagishi K, Tatematsu K, Yano R, Preston J, Kitamura S, et al (2009) CHOTTO1, a double AP2 domain protein of Arabidopsis thaliana, regulates germination and seedling growth under excess supply of glucose and nitrate. Plant Cell Physiol 50: 330–340. 19109301 10.1093/pcp/pcn201

[43] Kinoshita N, Berr A, Belin C, Chappuis R, Nishizawa NK, et al (2010) Identification of growth insensitive to ABA3 (gia3), a recessive mutation affecting ABA signaling for the control of early post-germination growth in Arabidopsis thaliana. Plant Cell Physiol 51: 239–251. 20022976 10.1093/pcp/pcp183

[44] Palatnik JF, Allen E, Wu X, Schommer C, Schwab R, et al (2003) Control of leaf morphogenesis by microRNAs. Nature 425: 257–263. 12931144 10.1038/nature01958

[45] Alonso-Peral MM, Li JY, Li YJ, Allen RS, Schnippenkoetter W, et al (2010) The MicroRNA159-Regulated GAMYB-like GenesInhibit Growth and Promote Programmed Cell Deathin *Arabidopsis* . Plant Physiology 154(2): 757–771. 20699403 10.1104/pp.110.160630 PMC2949021

[46] Mantovani R (1999) The molecular biology of the CCAAT-binding factor NF-Y. Gene 239: 15–27. 10571030 10.1016/s0378-1119(99)00368-6

[47] Li WX, Oono Y, Zhu JH, He XJ, Wu JM, et al (2008) The Arabidopsis NF-YA5 Transcription Factor Is Regulated Transcriptionally and Post-trancriptionally to Promote Drought Resistance. The Plant Cell 20: 2238–2251. 18682547 10.1105/tpc.108.059444 PMC2553615

[48] Mu JY, Tan HL, Hong SL, Liang Y, Zuo JR (2012) Arabidopsis Transcription Factor Genes NF-YA1, 5, 6 and 9 Play Redundant roles in Male Gametogenesis, Embryogenesis, and Seed Development. Molecular Plant [Epub ahead of print]. 10.1093/mp/sss061 22933713

[49] Zhao B, Ge L, Liang R, Li W, Ruan K, et al (2009) Members of miR169 family are induced by high salinity and transiently inhibit the NF-YA transcription factor. BMC Molecular Biology 10: 29. 19351418 10.1186/1471-2199-10-29 PMC2670843

[50] Navarro L, Dunoyer P, Jay F, Arnold B, Dharmasiri N, et al (2006) A plant miRNA contributes to antibacterial resistance by repressing auxin signaling. Science 312: 436–439. 16627744 10.1126/science.1126088

[51] Xia K, Wang R, Ou X, Fang Z, Tian C, et al (2012) OsTIR1 and OsAFB2 Downregulation via OsmiR393 Overexpression Leads to More Tillers, Early Flowering and Less Tolerance to Salt and Drought in Rice. PLoS ONE 7: e30039. 22253868 10.1371/journal.pone.0030039 PMC3254625

[52] Abel S, Theologis A (1996) Early genes and auxin action. Plant Physiology 111: 9–17. 8685277 10.1104/pp.111.1.9 PMC157808

[53] Gutierrez L, Bussell JD, Păcurar DI, Schwambach J, Păcurar M, et al (2009) Phenotypic Plasticity of Adventitious Rooting in Arabidopsis Is Controlled by Complex Regulation of AUXIN RESPONSE FACTOR Transcripts and MicroRNA Abundance. The Plant Cell 21(10): 3119–3132. 19820192 10.1105/tpc.108.064758 PMC2782293

[54] Yang JH, Han SJ, Yoon EK, Lee WS (2006) Evidence of an auxin signal pathway, microRNA167-ARF8-GH3, and its response to exogenous auxin in cultured rice cells. Nucleic Acids Research 34: 1892–1899. 16598073 10.1093/nar/gkl118 PMC1447648

[55] Guo HS, Xie Q, Fei JF, Chuad NH (2005) MicroRNA Directs mRNA Cleavage of the Transcription Factor NAC1 to Downregulate Auxin Signals for Arabidopsis Lateral Root Development. The Plant Cell 17: 1376–1386. 15829603 10.1105/tpc.105.030841 PMC1091761

[56] Wu G, Poethig RS (2006) Temporal regulation of shoot development in Arabidopsis thaliana by miR156 and its target SPL3. Development 133: 3539–3547. 16914499 10.1242/dev.02521 PMC1610107

[57] Xie KB, Wu CQ, Xiong LZ (2006) Genomic organization, differential expression, and interaction of SQUAMOSA promoter-binding-like transcription factors and microRNA156 in rice. Plant physiology 142: 280–293. 16861571 10.1104/pp.106.084475 PMC1557610

[58] Schwab R, Palatnik JF, Riester M, Schommer C, Schmid M, et al (2005) Specific effects of microRNAs on the plant transcriptome. Developmental Cell 8: 517–527. 15809034 10.1016/j.devcel.2005.01.018

[59] Fu CX, Sunkar R, Zhou CN, Shen H, Zhang JY, et al.. (2012) Overexpression of miR156 in switchgrass (Panicum virgatum L.) results in various morphological alterations and leads to improved biomass production. Plant Biotechnology Journal pp: 1–10. 10.1111/j.1467-7652.2011.00677.x PMC3489066 22239253

[60] Wang JW, Czech B, Weigel D (2009) miR156-regulated SPL transcription factors define an endogenous flowering pathway in Arabidopsis thaliana. Cell 138: 738–749. 19703399 10.1016/j.cell.2009.06.014

[61] Wu G, Park MY, Conway SR, Wang JW, Weigel D, et al (2009) The sequential action of miR156 and miR172 regulates developmental timing in Arabidopsis. Cell 138: 750–759. 19703400 10.1016/j.cell.2009.06.031 PMC2732587

[62] Prigge MJ, Otsuga D, Alonso JM, Ecker JR, Drews GN, et al (2005) Class III homeodomain-leucine zipper gene family members have overlapping, antagonistic, and distinct roles in Arabidopsis development. Plant Cell 17: 61–76. 15598805 10.1105/tpc.104.026161 PMC544490

[63] Boualem A, Laporte P, Jovanovic M, Laffont C, Plet J, et al (2008) MicroRNA166 controls root and nodule development in Medicago truncatula. The Plant Journal 54: 876–887. 18298674 10.1111/j.1365-313X.2008.03448.x

[64] Vaucheret H, Mallory AC, Bartel DP (2006) AGO1 homeostasis entails coexpression of MIR168 and AGO1 and preferential stabilization of miR168 by AGO1. Mol Cell 22(1): 129–136. 16600876 10.1016/j.molcel.2006.03.011 PMC2323247

[65] Ding D, Zhang L, Wang H, Liu ZJ, Zhang ZX, et al (2009) Differential expression of miRNAs in response to salt stress in maize roots. Annals of Botany 103: 29–38. 18952624 10.1093/aob/mcn205 PMC2707283

[66] Vaucheret H, Vazquez F, Crété P, Bartel DP (2004) The action of ARGONAUTE1 in the miRNA pathway and its regulation by the miRNA pathway are crucial for plant development. Genes Development 18: 1187–1197. 15131082 10.1101/gad.1201404 PMC415643

[67] Vaucheret H (2008) Plant ARGONAUTES. Trends in Plant Science 13: 350–358. 18508405 10.1016/j.tplants.2008.04.007

[68] Voinnet O (2009) Origin, biogenesis, and activity of plant microRNAs. Cell 136: 669–687. 19239888 10.1016/j.cell.2009.01.046

[69] Kantar M, Lucas S, Budak H (2011) MiRNA expression patterns of Triticum dicoccoides in response to shock drought stress. Planta 233: 471–484. 21069383 10.1007/s00425-010-1309-4

[70] Zhang ZX, Wei LY, Zou XL, Tao YS, Liu ZJ, et al (2008) Submergence-responsive MicroRNAs are Potentially Involved in the Regulation of Morphological and Metabolic Adaptations in Maize Root Cells. Annals of Botany 102: 509–519. 18669574 10.1093/aob/mcn129 PMC2701776

[71] Xu ZH, Zhong SH, Li XH, Li WX, Rothstein SJ, et al (2011) Genome-Wide Identification of MicroRNAs in Response to Low Nitrate Availability in Maize Leaves and Roots. PLoS ONE 6(11): e28009. 22132192 10.1371/journal.pone.0028009 PMC3223196

[72] Zanca AS, Vicentini R, Ortiz-Morea FA, Bem1 LED, Silva1 MJ, et al (2010) Identification and expression analysis of microRNAs and targets in the biofuel crop sugarcane. BMC Plant Biology 10: 260. 21092324 10.1186/1471-2229-10-260 PMC3017846

[73] Jones-Rhoades MW, Bartel DP (2004) Computational identification of plant microRNAs and their targets, including a stress-induced miRNA. Molecular Cell 14: 787–799. 15200956 10.1016/j.molcel.2004.05.027

[74] Abdel-Ghany SE, Pilon M (2008) MicroRNA-mediated Systemic Down-regulation of Copper Protein Expression in Response to Low Copper Availability in Arabidopsis. The Journal of Biological Chemistry 283: 15932–15945. 18408011 10.1074/jbc.M801406200 PMC3259626

[75] Freudenberg K (1959) Biochemische Vorgange bei der Holzbildung. In: Biochemistry of Wood (Eds K. Kratzl & G. Billek). London: Pergamon Press. pp: 121–136.

[76] Dong J, Kim ST, Lord EM (2005) Plantacyanin Plays a Role in Reproduction in Arabidopsis. Plant Physiol 138: 778–789. 15908590 10.1104/pp.105.063388 PMC1150396

[77] Banu MN, Hoque MA, Watanabe-Sugimoto M, Matsuoka K, Nakamura Y, et al (2009) Proline and gly-cinebetaine induce antioxidant defense gene expression and suppress cell death in cultured tobacco cells under salt stress. Journal of Plant Physiology 166: 146–156. 18471929 10.1016/j.jplph.2008.03.002

[78] Rajjou L, Gallardo K, Debeaujon I, Vandekerckhove J, Job C, et al (2004) The Effect of {alpha}-Amanitin on the Arabidopsis Seed Proteome Highlights the Distinct Roles of Stored and Neosynthesized mRNAs during Germination. Plant Physiology 134: 1598–1613. 15047896 10.1104/pp.103.036293 PMC419834

[79] Nakabayashi K, Okamoto M, Koshiba T, Kamiya Y, Nambara E (2005) Genome-wide profiling of stored mRNA in Arabidopsis thaliana seed germination: epigenetic and genetic regulation of transcription in seed. Plant J 41: 697–709. 15703057 10.1111/j.1365-313X.2005.02337.x

[80] Kimura M, Nambara E (2010) Stored and neosynthesized mRNA in *Arabidopsis* seeds: effects of cycloheximide and controlled deterioration treatment on the resumption of transcription during imbibition. Plant Molecular Biology 73: 119–129. 20099072 10.1007/s11103-010-9603-x

[81] Okamoto M, Tatematsu K, Matsui A, Morosawa T, Ishida J, et al (2010) Genome-wide analysis of endogenous abscisic acid-mediated transcription in dry and imbibed seeds of Arabidopsis using tiling arrays. The Plant Journal 62: 39–51. 20088898 10.1111/j.1365-313X.2010.04135.x

[82] Weitbrecht K, Muller K, Leubner-Metzger G (2011) First off the mark: early seed germination. Journal of Experimental Botany 62: 3289–3309. 21430292 10.1093/jxb/err030

[83] Sreenivasulu N, Borisjuk L, Junker BH, Mock HP, Rolletschek H, et al (2010) Barley grain development toward an integrative view. Int Rev Cell Mol Biol 281: 49–89. 20460183 10.1016/S1937-6448(10)81002-0

[84] Howell KA, Narsai R, Carroll A, Ivanova A, Lohse M, et al (2009) Mapping metabolic and transcript temporal switches during germination in Oryza sativa highlights specific transcription factors and the role of RNA instability in the germination process. Plant Physiol 149: 961–980. 19074628 10.1104/pp.108.129874 PMC2633829

[85] Lau et al (2001) MicroRNA and siRNA Cloning Protocol. Science 294: 858–862.11679671

[86] Sunkar R, Zhu JK (2004) Novel and stress-regulated microRNAs and other small RNAs from Arabidopsis. Plant Cell 16: 2001–2019. 15258262 10.1105/tpc.104.022830 PMC519194

[87] Sunkar R, Girke T, Zhu JK (2005) Identification and characterization of endogenous small interfering RNAs from rice. Nucleic Acids Research 33: 4443–4454. 16077027 10.1093/nar/gki758 PMC1182700

[88] Meyers BC, Axtell MJ, Bartel B, Bartel DP, Baulcombe D, et al (2008) Criteria for Annotation of Plant MicroRNAs. The Plant Cell 20: 3186–3190. 19074682 10.1105/tpc.108.064311 PMC2630443

[89] Mathews DH, Sabina J, Zuker M, Turner DH (1999) Expanded sequence dependence of thermodynamic parameters improves prediction of RNA secondary structure. Journal of Molecular Biology 288: 911–940. 10329189 10.1006/jmbi.1999.2700

[90] Allen E, Xie ZX, Gustafson AM, Carrington JC (2005) MicroRNA-Directed Phasing during Trans-Acting siRNA Biogenesis in Plants. Cell 121: 207–221. 15851028 10.1016/j.cell.2005.04.004

[91] Qiu CX, Xie FL, Zhu YY, Guo K, Huang SQ, et al (2007) Computational identification of microRNAs and their targets in Gossypium hirsutum expressed sequence tags. Gene 395: 49–61. 17408884 10.1016/j.gene.2007.01.034

[92] Du Z, Zhou X, Ling Y, Zhang ZH, Su Z (2010) agriGO: a GO analysis toolkit for the agricultural community. Nucleic Acids Research 38: W64–W70. 20435677 10.1093/nar/gkq310 PMC2896167

[93] Zhang LF, Chia JM, Kumari S, Stein JC, Liu ZJ, et al (2009) A Genome-Wide Characterization of MicroRNA Genes in Maize. PLoS Genetics 5(11): e1000716. 19936050 10.1371/journal.pgen.1000716 PMC2773440

