# Supplementary material for: Deep Sequencing of Maize Small RNAs Reveals a Diverse Set of MicroRNA in Dry and Imbibed Seeds
Source: PLoS One. 2013 Jan 24;8(1):e55107. doi: 10.1371/journal.pone.0055107 (PMC3554676; doi:10.1371/journal.pone.0055107)
Supplement: Additional File S5 — Secondary structures of putative novel miRNA identified in dry and imbibed seeds in this study. (DOC) [file pone.0055107.s005.doc]

**Additional file S5:** S**econdary structures of putative novel miRNA identified in dry and imbibed seeds.**

**(**Red colored letter: mature miRNA sequence; blue colored letter: miRNA* sequence.)

**zma-miRn1 AGUGGAUUAGAGGGGCUAAAA**

**-| C a u a a c CC C A**

**CAAAACC GA g gg uu gagggg uaaaaU CCUUCU AUUCA A**

**GUUUUGG CU C CC AA CUCCCC AUUUUA GGAGGA UAAGU A**

**U^ C - U A C U AA A U**

**zma-miRn2 AUGGAGAGAACUGAGAAGGUAA**

**-| C aac aa a CC A AA**

**AG AAGUGUAauggagag ugag ggu aGAAU CUUA UAUUC \**

**UC UUCACAUUACCUCUC ACUC CCA UCUUA GAAU AUAAG A**

**C^ A CUA C- A UA A UU**

**zma-miRn3a UGCACACGAGAUGGACUGGCC**

**G CU ---- U GAU - G U ------| A A GGAGG AAA**

**GUCU C GCUAGUUC UCU UGUGCGCAC AU AGA GGGUCUAGCGG AUCCUC UGUA GGCUG GCUC \**

**CAGA G cggucagg aga acacguGUG UG UCU CCUAGAUUGUU UGGGAG AUAU CUGGU UGAG G**

**- AG UAUc u gc- C G - CUCUGG^ - - AA--- CGA**

**zma-miRn3b UGCACAUGAGAUGGACUGGCC**

**C A-- - au a g A GG G CG---- AG-| AUGG**

**CUGGUC UGugc acaugag gg cug ccU UG GG CGGAU UCGUGU GGG \**

**GACUAG ACACG UGUACUC CC GGC GGG AU CC GUCUA AGCACG CUC G**

**C AGG U -- A G G AA G UAAAUG AAA^ GAGA**

**zma-miRn4 UGGCCUGCAGAAUAGUCGUCA**

**CU C - g c gu .-aCAGACA -| C AU**

**GCA GU CGu gc ugcagaaua cguc CUGC CCUG AGC C**

**CGU CA GCA CG ACGUCUUGU GUAG GACG GGAC UCG C**

**AC - U G C UG \ ------- C^ C CU**

**zma-miRn5 UUUGGGGUGGAUACGUGGUCA**

**CC C U | GCAG**

**UGUUUGGAUG CCAUGUAUCCACCUCAA CCAUGU--GUGGAAU \**

**ACAAACCUac ggugcauaggugggguu GGUACA CACCUUA U**

**GA u u \ ^ AAUU**

**zma-miRn6 GAAAUGUUGUCUGGUUCAAGG**

**- --- Uga uu a UUUGCG --------| UC C**

**GCU UUUGAG aaug gucugguuc aggUCUUGC AUUUGAGGA UGA CAUG U**

**CGA GGACUC UUAC CGGACCAGG UCUAGAACG UAGGCUCCU GUU GUAC U**

**U UAA CCC UU C UA---- UAUUUUUU^ GU G**

**zma-miRn7 UCGGACCAGGCUUCAUUCCCCG**

**U| UCU A C C ACG A- U UG**

**CAUG GUCG GGGGAAUGA GUC GGUCCGA AGCC CGGC GC C**

**GUAC CAGU ccccuuacu cgg ccaggcu UCGG GCCG CG U**

**G^ U-- g u a --- GC C CG**

**zma-miRn8 AUGCAGAACAAUUUACAGACG**

**-| C a g cg A AUUUCUCA GCA**

**UAG AUUCUU ugca aacaauuuacaga GAAAUG UUUGUACU GGU A**

**AUC UAAGAA ACGU UUGUUGAAUGUCU CUUUAC AAACGUGG CCA A**

**C^ A C G AG C ACUCUUUA ACC**

**zma-miRn9 UUCUGGAGGGGAUUUGAGUUU**

**G| U U AG GC**

**GCUAGUUUGGAAACUCAAAUCCCUUCCGG AUUGGAGG GAUUGAGGGG AAU A**

**CGAUCAAACCuuugaguuuaggggagguc uAAUCUCC CUAACUCCUU UUA A**

**-^ u U CU AU**

**zma-miRn10 UGGAUUAUGGUGGAAGGAUA**

**-| A a a A C AGA U AAU UCC**

**UUAUAUAA Uuggauuauggugga gg ua GAGGGUAAAAUAU ACUUU AUC CAAAUAAGUUGA AAGCUA A**

**AAUAUAUU AAUCUAAUACCACUU CC AU CUCCCAUUUUAUA UGAAA UGG GUUUAUUCGAUU UUCGAU A**

**U^ C C C C A CCC U AUC CAU**

**zma-miRn11 GAUCGGCUGAUCGUUUGGCCUG**

**C| UCA g - u g UG**

**GUGAUU gaucg c ugaucguu g ccugGG \**

**CGCUGA UUAGC G ACUAGCAG C GGAUUU A**

**C^ --- G G - G UU**

**zma-miRn12a UUUCAAAGUUCGUGGACCUAA**

**AAGA C A ----- C .-A - GUAU G**

**AUG CACUUAGGUCCACGAACUUUGAAA UGUAUUUCUAGGUCCUUGAACUUGUUAAGUGA CACAC UC GUG GUCG GGUCCA A**

**UAC GUGaauccaggugcuugaaacuuu ACAUAAAGAUCCAGGAACUUGAACAAUUCACU GUGUG AG CAC UAGC CCAGGU A**

**CCC- A C AUAUG U \ - G AU-- A**

**zma-miRn12b UUUCAAAGUUCGUGGACCUAA**

**UGA U C- U A C G C .-UAUCCCUAAACU| AA**

**GUGUCACUUA GUCCAC ACUUUGAAAAC CAUUUC GGGUC CUGAACU GUUAAGUGGUG ACUGCA UGUA A**

**UACAGUGaau caggug ugaaacuuuUG GUAAAG UCCAG GAUUUGA CAAUUCACCAC UGGCGU ACGU U**

**UCC c cu U A A A C \ ------------^ GA**

**zma-miRn12c UUUCAAAGUUCGUGGACCUAA**

**UCAA UGCCACUU - U C C A .-ACACUUCAC AAA-| U**

**GGGU GGUUCAUGAACUUUGAAAACA AUUUCU GUC UUAAA UUGUUAAGUG UGUACC UAGACUUGUA CAG G**

**UCCA ccaggugcuugaaacuuuUGU UAAAGA CAG GAUUU AACAAUUCAC ACGUGG AUCUGGGCGU GUC C**

**UCAA CUaau--- G U U A C \ --------- CGCC^ A**

**zma-miRn13 UCUAACGGACCAGAAUGCUCA**

**AGC U UC .-AGAUAUAAAUGU| UG UAG UU**

**GUGCAAGUGAGCAUUCUG UUCGUUAGAUCAUGA CAACUGU GGUU U AGGUUU U**

**CACGUUCacucguaagac aggcaaucuAGUGCU GUUGGUA UCGA G UCUAAA U**

**AUA c GA \ ------------^ GU CA- UA**

**zma-miRn14a UUACGGUAGAGACAUUAGAAAA**

**AGUA AUAUUUG - AA .-AA A G A**

**GU UUUCUAAU UCUC UACUG AAUAU UCA UUU \**

**CG aaagauua agag auggc UUAUA AGU GAG U**

**G--- GUUUa-- c -- \ -- A G G**

**GAAAUUAAAAC AC A G AA--- U GAGGGA G GA GCAUAUA**

**GUG UA CUAUUGU AAUUC UGUAU G UCU CUCUU UGCUG C**

**CAC AU GAUAACA UUAAG ACAUA C AGA GAGAA GUGGC U**

**auuAU------ AC C G GGUAG - AAGCAA A -- ACACUUA**

**zma-miRn14b UUACGGUAGAGACAUUAGAAAA**

**AGUA AUAUUUG - AA .-AA C- UG A GAA-- GAA**

**GU UUUCUAAU UCUC UACUG AAUAUAU AGUUUA G GGU AUAUU A**

**CG aaagauua agag auggc UUAUGUA UUAAGU U UCA UGUAA U**

**G--- GUUUa-- c -- \ -- AC GU A AUCAG AAU**

**GGU-- AUCU CU GC UAUA CAC GUGAA GA**

**GGG GCU UGAU UGGCA CUAUC ACG GU A**

**CCC CGA AUUA ACCGU GGUAG UGC CA G**

**auuAU ACAU -- -- UAAG ACA AAG-- AA**

**zma-miRn15 UGGCUGUGAUGACAAAAAGGUUA**

**UGCUGC - gu ---| a UGGUU A UG**

**UGCGu ggcu gaug acaaa agguuaUU GAUU UGAUUCUUAA C**

**ACGUA CCGA CUGU UGUUU UCCAGUAG UUAA ACUAGGAAUU C**

**GAAGUA G GU GCG^ A UG--- - UA**

**zma-miRn16a AAAAGAAACGGAUGGAGUAUA**

**UCCGC| UCUG C G**

**UCAU GUACUCC UCCGUUUCUUUUUAUUUGUCGCUGGAUAGU U**

**AGUA uaugagg aggcaaagaaaaAUAAACAGCGACCUAUCA A**

**UUUCA^ Ua-- u C**

**zma-miRn16b AAAAGAAACGGAUGGAGUAUA**

**ACAA G A C G .-AAC| UCG U**

**CUAUU UA ACUCC UCCGUUUC UUUUA GUCG CUA U**

**GAUAG au ugagg aggcaaag aaaAU CGGU GAU U**

**UCAG A a u a \ ---^ UA- A**

**zma-miRn16c AAAAGAAACGGAUGGAGUAUA**

**A CU--| C UC C CAA**

**GC AGG AUGUACUU UCCGUUUCUUUUUAUUUGUCG UGGAUAGUG U**

**CG UUC Uauaugag aggcaaagaaaaAUAAACAGC ACCUAUCAC U**

**- CAAU^ - gu A AUU**

**zma-miRn17 UGUGAUCAUAGUUGGGAGUGCC**

**A UCUCAA U A C- .-CUCUAAUAU -| A**

**CCC GGUACUCC UAAC GU UCACA GGAA CUC U**

**GGG ccgugagg guug ua agugu CCUU GAG A**

**C UGUCAC - a cu \ --------- A^ A**

**zma-miRn18 UAACUCCUUCUGAAGCUGCUU**

**-| GCUCA u c-- ga uCUUU**

**UCU GC aacuc uucu agcugcu \**

**AGA CG UUGGG AAGA UCGAUGA U**

**C^ ACUG- - UAU AC CCGUC**

**zma-miRn19 AACAUUCUCUAUCCGUUAACA**

**UUU--- C GAAAC - AU .-G G**

**UUAACGGAUAGAGAAU UUCUCA UUC CCAU UUGAU UUUG A**

**aauugccuaucucuua aaGAGU AAG GGUA AACUG AAAC A**

**ACGAac C AGUCU A -- \ - G**

**zma-miRn20 UGCAAUGAUGAUACUGUUUUU**

**G UCAU-- U C UUC UU AGC -- .-UUU| UAUUUUUUC**

**UGGGCUG GAUA GUG UU CAUUGCA UG UCAUUCA AGAAUA UGGUAU \**

**ACUCGAC uugu cau ag guaacgu AC AGUAAGU UCUUAU AUCAUG U**

**- CCAuuu - - ua- UU --- UU \ ---^ UCGUGCCUU**

**zma-miRn21 AACGAUGAUGAAAAGCUCCGUGA**

**GA------ C -- C GC .-UUUUCAUCCCUA CAACUUU UUUU**

**GGGGUUUUUC GUC CGUUUC G CG GUGC GU \**

**ccucgaaaag uag gcaaAG C GC CGCG CA U**

**ACGCagug - ua - UA \ ------------ UCUACUU UGUA**

**zma-miRn22a GAUGCUUGGGCACGCCUCGCGG**

**G GC A gaug -- -| CCA**

**AG CA CU cuugggc acgccucgcg gU \**

**UC GU GA GGAUUCG UGCGGGGCGU CG A**

**A UU C GG-- UC A^ CAC**

**zma-miRn22b GAUGCUUGGGCACGCCUCGCGG**

**GA| CAG gaug -- - CCA**

**GGC UC cuugggc acgccucgcg gU \**

**CCG AG GGAUUCG UGCGGGGUGU CG A**

**UA^ --- GG-- UC A UAC**

**zma-miRn22c GAUGCUUGGGCACGCCUCGCGG**

**G GC A gaug -- -| CCA**

**AG CA CU cuugggc acgccucgcg gU \**

**UC GU GA GGAUUCG UGCGGGGCGU CG A**

**A UU C GG-- UC A^ CAC**

**zma-miRn22d GAUGCUUGGGCACGCCUCGCGG**

**GA| AGUUgaug -- - CCA**

**GGCU cuugggc acgccucgcg gU \**

**CCGA GGAUUCG UGCGGGGCGU CG A**

**UA^ GGG----- UC A UAC**

**zma-miRn23 AACAGCAAAACCGAGGGCACC**

**G| G C CU UU UUCUA**

**UUUUUAUGGUGGUGCCCUCGGUUUUGCUG UGU CUCAUUUUUA UUUAGUUGUGUUUUUU UCUCAGUUUUGUCAUUCUA \**

**AAAAAUACCAccacgggagccaaaacgac aCA GAGUAAAAAU GAAUCAACACAAAAAA AGAGUCAAAACAGUAAGGU U**

**-^ a U AG -- CACGA**

**zma-miRn24 UCUCCAUGGGCUGCGUCGCAU**

**A| GCCAGG UG .-GAAGCCAUGAGAUC AUC**

**CGAAGGUGGCAUGUGGCGCAGUCCAUGGAGAUGGGGGCGCCGGUU ACGACGCCG AAAUUGGCAGCA GG A**

**GCUUCCACCGuacgcugcgucggguaccucuACCCCUGCGGUCAA UGCUGCGGC UUUAACCGUCGU CC A**

**-^ ------ GU \ -------------- CAA**

**zma-miRn25 UAAUGGAUCAAAUGGUUUGCU**

**G A C A AC .-ACAGA| A UG UUAG U**

**CAA UGUGCAAGCAAAUCAU UGAUUCAUUA AUU GAUUCAACC CAC AUCA GUUUG GGUUUUU U**

**GUU AUACGUucguuuggua acuagguaau UAG CUAGGUUGG GUG UGGU CGAGU UUAGAAA A**

**- C a C CA \ -----^ G -- UA-- U**

**zma-miRn26 UUAGGCUCGGGGACUACGGUG**

**C A cu ga C UG .-CUAU| A A**

**GGCCU ACAuuagg cggg cuacggug AGCCAAGGA ACUUGCCGAU CGUCG UC \**

**UCGGA UGUAGUCC GUCC GGUGCCAC UCGGUUCUU UGAACGGCUA GCAGC AG A**

**U A UC UC A GU \ ----^ G C**

**zma-miRn27 AAGCAUGAAGGUGGGAGGACU**

**- Ca c a - AGG---| UGGG**

**ACCAAGACA ag aug aggugggaggacuUUUUUAAAAAUA UCU GAGAGG \**

**UGGUUCUGU UC UAC UCCACCUUCCUGGAAAAAUUUUUAU AGG UUUUCU A**

**U -- A C A AAAAAG^ UGGG**

**zma-miRn28a UUUGGGAGCAAGUGGAAUGGA**

**UA - A--| a UGAAGA GC AU-- A**

**UAUC UAU uuugggagc aguggaauggaGAGAAU G UAUAAUU UUGUUAUUCA A**

**AUGG GUG AAACCCUCG UCACUUUAUUUCUCUUA C AUAUUAG AACGAUAAGU A**

**GA C AAC^ C ------ UA GGAG U**

**zma-miRn28b UUUGGGAGCAAGUGGAAUGGA**

**CUA A- u - ---------------- -| C A**

**AGG UUGuuugggagcaag gg aauggaGG AGUC CCU GCUAUUUA A**

**UCU AACAAACCUUCGUUU CC UUAUCUCC UUAG GGA UGAUAAGU A**

**UAA CG - A CCUAACUCCCCCGAUC A^ - U**

**zma-miRn29a AAGUUACAGAUGUAUGGAGCA**

**CUUA A .-C| UC**

**UAAAAUUGCUCCAUACA CUGUAACUUUGGACUAAA UC C**

**AUUUUAacgagguaugu gacauugaaACCUGAUUU AG U**

**AAAC a \ -^ UU**

**zma-miRn29b AAGUUACAGAUGUAUGGAGCA**

**CUUA A | UC**

**UAAAAUUGCUCCAUACA CUGUAACUUUGGACUAAAC--UC C**

**GUUUUAacgagguaugu gacauugaaACCUGAUUUG AG U**

**AAAC a \ ^ UU**

**zma-miRn30 AUGGUGCAUUGACUUGGUCAA**

**UGCUACUA U AG A A**

**UG CCAAGUCAAUGCAUCAUUGCCCCU UUUUGCC UC \**

**ac gguucaguuacgugguaACGGGGA GAAGCGG AG A**

**CUAa---- u CG A A**

**zma-miRn31 AUGGAUCCUGGAGAUAAGGCU**

**UAG| UAU a - a u AA AUC**

**AUGGGG gg ucc uggagaua ggc GAA AU C**

**UAUUCC UC GGG ACCUCUGU CCG CUU UA U**

**GGG^ U-- - U A U AC CCC**

**zma-miRn32 UCUGUUUGAAUGCACUAGAGC**

**- CUU g - C .-U .-CAUCCAAACAA| UU**

**GUA AGGGucuguuugaaugcacua agcUAAUAGUUAGCU GCU AAA UAGCUG--GAGA CCAGCUAAUAG \**

**UAU UCCCGGACAAACUUACGUGAU UUGAUUAUCAAUCGA CGA UUU AUCGAC CUCU GGUCGAUUAUC C**

**A AAU G C U \ - \ \ -----------^ GA**

**zma-miRn33 CGGAGGGGAUUGGAGAGGCUA**

**- C AAAAC-- ga--| a C UC A**

**GU AC cg ggggauuggag ggcuaGAAU U UUCUUAUUCA A**

**CA UG GC UCUCUAACCUC CUGAUCUUA G AAGAAUAAGU A**

**A A GAAUAUC AAAC^ C C GA U**

**zma-miRn34a UUGGAUUUUGAUUGGAUGCAC**

**GCAUCCAAUCAAAAUCUAACA**

**CCG- .-aA C - G GG G**

**GUgcauccaaucaaaaucuaac UUA UG CUAUUUUA GU AA A**

**cacguagguuaguuuuagguuG AAU AC GAUAAAAU UA UU G**

**GUCA \ -- U A G AA G**

**zma-miRn34b UUGGAUUUUGAUUGGAUGCAC**

**C G U .-GAUGGUU| GG U**

**AAGU GUC GGUGCAUUCAAUCAAAAUCCAAC GUUAUUUUACGU AA G**

**UUCA UAG UcacguagguuaguuuuagguuG CAAUAAAAUGUA UU G**

**- A C \ -------^ AA G**

**zma-miRn34c UUGGAUUUUGAUUGGAUGCAC**

**GU AAGCUAUA CA | GG**

**GCAAUC UGGUGCAUCUAAUUAAAAUCCAA GUGACUGCUAU--UUUAGCU \**

**CGUUAG ACcacguagguuaguuuuagguu CACUGACGAUA AAAUUGG A**

**UU -------- AC \ ^ GA**

**zma-miRn34d UUGGAUUUUGAUUGGAUGCAC**

**UAGUACAACC .-GUUUUAUAAUUGACCUCUUUAUCUCUUCUAU| AAA**

**GUGCAUCCAAUCAAAAUCUAAC CUAA \**

**cacguagguuaguuuuagguuG GAUU A**

**UUUGCUAGAA \ -------------------------------^ CAA**

**zma-miRn34e UUGGAUUUUGAUUGGAUGCAC**

**CAU--- - AA U U .-AUAUGGAAGGGG A**

**UC AG CAUCUAAUC AGAUUCAAUGGU UC UUAC A**

**AG Uc guagguuag uuuagguuACCA AG AAUG A**

**UUCGCU A ac u C \ ------------ U**

**zma-miRn34f UUGGAUUUUGAUUGGAUGCAC**

**AAG U C g a- u- .-acCAGA| AAUCCAC CA AAC**

**G CA CGuug auuuug uugga gc UUGCUC AUUUGUUA UG \**

**C GU GCAAC UAGGAU GACCU CG AAUGAG UAAACGAU GC C**

**CAA - U G AG UC \ ------^ CAA---- AA CUA**

**zma-miRn34g UUGGAUUUUGAUUGGAUGCAC**

**A - G C ACA GG--| AA**

**AGUG AU UGGUGCAUCUAAUCAAAAUUCAA GGUGGC UAUUUUA UAG G**

**UUAC UA ACcacguagguuaguuuuagguu CCAUUG AUAAAAU AUU G**

**- U G A ACG AUAA^ GG**

**zma-miRn35 GGGCGUUUGGUCUAGUGGUAU**

**CAACCUGUA CU - CCACA AU UG .-GACC .-AAAUU| U C**

**UAUGCUAUU GCCGAA GCCU UG UU UUAG UGA GCA CA U**

**Guaugguga ugguuu cggg AC AA AAUC ACU CGU GU U**

**UCGCUCUUA uc g ACA-- AC GU \ ---- \ -----^ - G**

**zma-miRn36 UCGCUAGAUCGUUGAGGGAUU**

**A - C A .-A C--| AUCC**

**AUUCC CUCCAAUCCCUCA CGAUCUAGC ACCAUAACAGU GGCUACAU UAUG A**

**UGGGG GAGGuuagggagu gcuagaucg uGGUAUUGUCA CCGAUGUA AUAC U**

**- A u c \ - CGU^ CACA**

**zma-miRn37 UGAUAGAAGAGAGUGAGCAC**

**U| UCC A A C U CCGGC**

**CCAUA UG UGCUCACU UCU CU UCAG \**

**GGUAU AC acgaguga aga ga aguU U**

**-^ CGU c g a u UAACU**

**zma-miRn38a UGGACAAGAUUUGAUGUUAGC**

**G A U A C C A AAC .-A| CGAUGUG UCUAU**

**GU CUAACAUGCUAACAUCAAAUC UUGUCUAUCGGAUC AU AUCUG GGUUGA UGU CCC UCUGAGGA CCCUAU \**

**CA GAUUGUAcgauuguaguuuag aacagguAGCCUAG UG UAGAU CCGAUU ACG GGG GGAUUUCU GGGAUG G**

**- C - G A A C CUA \ -^ UA----- CAAAA**

**zma-miRn38b UGGACAAGAUUUGAUGUUAGC**

**UAUUA A U C C CCA C C .-C| AC**

**GGCGUGCUAACAU AAAUCUU UCCA UAGAUC ACCAUCUG CUG GUGUAAUC CA UAGGG G**

**UUGUAcgauugua uuuagaa aggu AUCUAG UGGUAGAU GAU CACGUUAG GU AUCCU A**

**UACGA g c U A ACA U A \ -^ AC**

**zma-miRn39 UUGAGCCGCGCCAAUAUCUCU**

**CAA| AA C A C A AA**

**UGGA GAG GAUAUUGGUG GGUUCAAUC GAUGUU GGUUUUACA \**

**ACCU UUc cuauaaccgc ccgaguuAG CUAUGA CUAAAAUGU A**

**UUA^ CC u g U - CG**

**zma-miRn40 UGAACGUGAGUGGGGGUGUUGA**

**U -| UC AUU C UUUC**

**UUGUGC CUUUA CAUUU UUCACGUUUG GCC \**

**AAUAUG GAagu guggg gagugcaagu CGG U**

**G U^ u- ggu - UCUC**

**zma-miRn41a UAUAUAAGUUGGAUUAUGGUA**

**- -| gu AAAAUAUAU U- AAAU G CC CUA A UGUA**

**CCUU UUAGAUuauauaa uggauuauggua GG AGUAAA UAUUUUG GA AUAAGUAAG AAAUAA CU U**

**GGAA GAUUUAAUAUAUU ACCUAAUACCAU CC UCAUUU GUGAAAU CU UAUUUAUUC UUUAUU GA U**

**A G^ UG --------- UC ---- A AA AAC C UGAC**

**zma-miRn41b UAUAUAAGUUGGAUUAUGGUA**

**C C --- U ---------------- GA CC -- AC UAAU--| CCC**

**CUC UAGAUuauauaaguuggauuauggua AAAA GC AGGAG UAAAAUAUCA UUUUA AUCACAAAUAA UGAAA CUA A**

**GAG AUUUAAUAUAUUCAAUCUAAUACCAU UUUU CG UCUUU AUUUUAUAGU AAAAU UGGUGUUUAUU AUUUU GAU A**

**A A UAU C AUCAUAUAUAAUACCA UC -- CA CA UUUUCC^ CAU**

**zma-miRn41c UAUAUAAGUUGGAUUAUGGUA**

**- C-- aAAC A A -| AC C ACA**

**UCUC UAGAUuauauaaguuggauuauggu GG UAGGAGGGUA AAUAUCACUUUG GA CACAAAUAGGUUGAAA AAGCUA U**

**AGGG AUCUAAUAUAUUCAAUCUAAUACUA CC AUCCUUCUAU UUAUGGUGAAAC CU GUGUUUAUUCGACUUU UUCGAU U**

**A AAU CCUU C C A^ A- A GGU**

**zma-miRn41d UAUAUAAGUUGGAUUAUGGUA**

**- - U guaC UUAAAAG-| UUA A AAUA**

**CC UCCUA AUuauauaaguuggauuaug AAG GGUAAAAUAUCACUUU GAUUAU AAUAAGCU \**

**GG AGGAU UAAUAUAUUCAACCUAAUAU UUC UCAUUUUAUAGUGAAA CUAGUG UUAUUCGA U**

**A A C GACC UUAUCUUA^ CA- G AGAU**

**zma-miRn41e UAUAUAAGUUGGAUUAUGGUA**

**G| U a g aAAAACG GA CAC ACCAC**

**CUCCUA AUuauaua guug auuauggu UAGGA GUAAAAUAU UUUAGU \**

**GAGGAU UAAUAUAU CAAC UAAUACCA AUCCU CAUUUUGUA AAGUCG A**

**A^ C A A CCUUCCA AC --- AAUAA**

**zma-miRn41f UAUAUAAGUUGGAUUAUGGUA**

**- aA A AUC--| U A U C CCC**

**AGCCU--UAUAUuauauaaguuggauuauggu AAGAGUAG AG AGUAUUA UUUGGGAUUACA AUAA CUGAAAUAAG UA A**

**UCGGA AUAUAAUAUAUUCAACCUAAUACCA UUUUCAUC UC UUAUAGU AAAUCCUGGUGU UAUU GAUUUUAUUC AU A**

**U \ CG C CCAUU^ U A C A AAU**

**zma-miRn41g UAUAUAAGUUGGAUUAUGGUA**

**C -- aAAAAAAAUAG C----| C CUC AG CC UC A CCC**

**CUCCUAGAU uauauaaguuggauuauggu GAGGGUA AAUA UA UG AUCA AAUAA UGAAAUA GCUA A**

**GAGGAUCUA AUAUAUUCAACCUAAUACCA CUCCCAU UUAU AU AC UGGU UUAUU ACUUUAU CGAU A**

**A AC ACUUCACAUA- UUUUU^ A AAA CU AU CA C CGU**

**zma-miRn41h UAUAUAAGUUGGAUUAUGGUA**

**- -| u a A A GA A AC A A GCA**

**CUUUC UAAAUuaua aaguuggauuauggu GAA GGUAG AG UAAAAUAUC CUUUAGA CAUAAAUAAGCU AAAUAA CUA A**

**GGGAG AUUUAAUAU UUUAAUCUAAUAUCA CUU UCGUC UC AUUUUAUGG GAAAUCU GUGUUUAUUCGA UUUAUU GAU U**

**A A^ U C A C UC A A- C C GAG**

**zma-miRn4i UAUAUAAGUUGGAUUAUGGUA**

**- C A u gu aAAAUG .-AAA| CGC**

**CCU CUA AUuaua aa uggauuauggu GUA--GGAGGU AUAC \**

**GGA GAU UAAUAU UU AUCUAAUACUA CAU CCUCUA UAUG U**

**G A C U UG CCUUCA \ \ ---^ AUU**

**zma-miRn4j UAUAUAAGUUGGAUUAUGGUA**

**A| C A GA U A U C C A GUA**

**CUC UGGAUuauauaaguuggauuauggua AAG UA AAGGGU AAAUAUCACUUUA AA UA AAAUAA CUGAAAUAAGCUA U**

**GAG AUUUAAUAUAUUCAAUCUAAUACCAU UUC AU UUCUUA UUUAUAGUGAAAU UU GU UUUAUU GACUUUAUUCGAU U**

**G^ U C UC U A C A A C GGU**

**zma-miRn42 UUUGAUGGCAUUGAAGGGAUU**

**UG A ugau-- aa-- .-U AAUUUCUUG-| UUUUUGGA**

**AUAUA Guuu ggcauug gggauuU GUCUACA UGUGGG \**

**UAUGU UAAA UCGUGGC UCCUAAA CAGGUGU GCAUUU A**

**GA A UAUCCU GCGA \ - AGUCUUUUAA^ UCUAAUUC**

**zma-miRn43a UCUUGGACACUAGAAAUGGUAC**

**A UC GU --- - U AC ---- .-UGAG UG UGAG --- A .-A| U AGA**

**GCCG GGG UGCCG CUGG GUUCAGGAG CUG CGGAG UCC CAGCCCC U GGCG AACC UGGGCC CGA GGAU A**

**UGGU CCC auggu gauc cagguucuC GAU GCUUU AGG GUCGGGG G CCGC UUGG GCCCGG GUU CCUG G**

**- CU Uc aaa a - AU GUAA \ ---- GU CAG- AGA - \ -^ - CGA**

**zma-miRn43b UCUUGGACACUAGAAAUGGUAC**

**-| a U U C A A G AACAGC C A A .-AGAAAAAAAA**

**UGGUUUUAGCucuuggacacu gaaaugguacUAUU AUUAG AA AU CUGAGAAA GUUG UUAUUAUC ACACCG AGC AU GAAAUUUCCAUUUU U**

**ACCGAAAUCGAGAACCUGUGA CUUUACCAUGAUAA UAGUC UU UA GAUUCUUU UAAC AGUAAUAG UGUGGC UCG UA UUUUAAAGGUAAAA A**

**C^ C C C A C G A ACA--- U C A \ ----------**

**zma-miRn43c UCUUGGACACUAGAAAUGGUAC**

**- ag A A A CA G -| C A AUAC- CA G AAAU**

**UGGCUUUAGCucuuggacacu aaaugguacUAUUUAUCAG AACACACU AG AA UUG UUAUUAUC AG GGCACA CGCAGCG AAAUUUC UUUUAG AA C**

**ACCGAAAUCGAGAACCUGUGA UUUACUAUGAUAAAUAGUC UUGUGUGA UC UU AAC AGUAAUAG UC CCGUGU GUGUUGC UUUAAAG AAGGUC UU G**

**C CU C C C AC A A^ - G GAAUU UA G CU--**

**zma-miRn44a AGCGGUGGAAGGGGCAUGCAGA**

**UA C cg u g G CAC C .-UGU| GAA**

**GUAGCAG ag guggaaggggca gca aGGA GAG GAG GAGG GGCUG \**

**CAUCGUC UC UACCUUCUCCGU CGU UCCU CUC CUC CUCC CCGAC G**

**CG U CU C G - --- A \ ---^ GAA**

**zma-miRn44b AGCGGUGGAAGGGGCAUGCAGA**

**CU| C cg u g CACG U GG**

**GUAGCAG ag guggaaggggca gca aGGAG AACGAGG GU \**

**CAUCGUC UC UACCUUCUCCGU CGU UCCUC UUGCUCC CG U**

**CG^ U CU C G UCGA C GU**

**zma-miRn45a UGGAGGGGAUUGAGGGGCAUA**

**-| A a A C U U**

**GCAUGGGGA uggaggggauugaggggc uaUAAUCCC UG UG UCA U**

**CGUACCCUU ACCUCUCCUAACUCCCCG AUAUUAGGG AC AU AGU U**

**A^ A G A A C U**

zma-miRn45b **UGGAGGGGAUUGAGGGGCAUA**

**AAU C -| C UU CUC - CC U C**

**GGGAUU UA GCCCCUC AAU CUUCCGAUUUU GC AC AAAU AGUC U**

**CUCUAA au cggggag uua ggagguUAAGG CG UG UUUA UCGG A**

**UCU U a^ - gg AUA C A- U A**

**zma-miRn45c UGGAGGGGAUUGAGGGGCAUA**

**AACAA--| GA A GUC**

**GA UuggaggggauugaggggcauaUAAUUC UUGCUA A**

**CU AAUCUCCUCUAAUUCCCCGUAUAUUAAG AACGAU U**

**AGGUACC^ G- G AAG**
