# Supplementary material for: Deep Sequencing of Maize Small RNAs Reveals a Diverse Set of MicroRNA in Dry and Imbibed Seeds
Source: PLoS One. 2013 Jan 24;8(1):e55107. doi: 10.1371/journal.pone.0055107 (PMC3554676; doi:10.1371/journal.pone.0055107)
Supplement: Additional File S6 — Secondary structures of putative novel miRNA only identified in dry seed in this study. (DOC) [file pone.0055107.s006.doc]

**Additional file S6:** S**econdary structures of putative novel miRNA identified in dry seed**

**(**Red colored letter: mature miRNA sequence; blue colored letter: miRNA* sequence.)

**zma-miRds1 AGUCUAUAAAUGCAACUGGUCG**

**UUUU .-Aagucuauaaau g-- .-gU| AA**

**UCCAC gcaacu guc GUUCA A**

**AGGUG CGUUGA UAG UAAGU U**

**UAGC \ ------------ AUA \ --^ CG**

**UUAUUUA------- .-UA AG - .-GA G AG**

**UGAUUGUAU GAG GUG GUUGUUGAU GAUU GGGUGA A**

**AUUAACAUA CUC UAC CGACGGCUG UUAA CUUACU A**

**AAAGUCGUAGUCUA \ -- AU U \ -- A GU**

**zma-miRds2a AAACCCGGUUUUGGACGGUUU**

**C C A C CCU C - -- .-CCCCCGAACU C-- C--| A**

**GGCCCAU CAAAACC UCCAAAACCGGGUUUGCUCAAA UGGAUG ACGUGGCGUGCUA GUUGGAGU UACU UGAA UGGCAUG UGUCA UUGG U**

**CCGGGUA GUuuugg agguuuuggcccaaaCGAGUUU GCCUAC UGCGCCGCACGGU CAACCUCG AUGA ACUU ACCGUGU ACGGU AGCC C**

**- C c A AAC A U UU \ ---------- CCU UCA^ C**

**zma-miRds2b AAACCCGGUUUUGGACGGUUU**

**UAA UGG C- G A C A A - CUAAUA .-A -| UGACACAA**

**CC UCGG CCGU CAAAACC GGUUUGCUCAAACCGGAUGCCGA GUGGCGU CCAC UUGGAG CACU AGUUC GGGUCC CAAG \**

**GG AGUu ggca guuuugg ccaaaCGAGUUUGGCCUACGGCU CACUGUA GGUG AACCUC GUGA UCAGG CCUAGG GUUU C**

**CCA UA- uu g c A C C A AC---- \ - A^ GAAUCGUC**

**zma-miRds2c AAACCCGGUUUUGGACGGUUU**

**UGC A A - ACC U UG C AC .-CAC UC ACU G**

**AAAACCGUC AAAACCGG UUU CUUCAA AG UGU A GUG GUGUCAUGUUGGAGU UUGGG CCCGA UGACAU \**

**Uuuuggcag uuuuggcc aaa GAGGUU UC ACA U CAC CACGGUGCAACCUCG AGCUC GGGUU ACUGUG C**

**A-- g c C GA- U GU A CA \ --- CU C-- U**

**zma-miRds2d AAACCCGGUUUUGGACGGUUU**

**UGC A A - ACC U UG C AC .-CAC UC ACU G**

**AAAACCGUC AAAACCGG UUU CUUCAA AG UGU A GUG GUGUCAUGUUGGAGU UUGGG CCCGA UGACAU \**

**Uuuuggcag uuuuggcc aaa GAGGUU UC ACA U CAC CACGGUGCAACCUCG AGCUC GGGUU ACUGUG C**

**A-- g c C GA- U GU A CA \ --- CU C-- U**

**zma-miRds2e AAACCCGGUUUUGGACGGUUU**

**U A U---- GC A A AUG .-AAAGUAAC -|U CA G**

**GG UCC AAACCGUCCAAAACCGGGUUU UCAAACC GAUGUC AC GCGUGUCACGUU UUGG G CUC AAGUGGCAU \**

**CC GGG uuuggcagguuuuggcccaaa AGUUUGG CUACGG UG CGCACGGUGCAA AGCC C GAG UUCACCGUA C**

**- - CACGU GA C C AAA \ -------- U^U -- U**

**zma-miRds3 UAGGCAAUAAAGAGACAGACAA**

**- .-AGU g u-- g ga- .-AAAA| UC**

**UGGCC UCua gcaa aaaga aca caaCU AC U**

**ACUGG AGGU UGUU UUUCU UGU GUUGA UG A**

**U \ --- A UGU A AAG \ ----^ UU**

**zma-miRds4 CCUGGAGGAUGAGGUCGAGGAC**

**C CAG G GG| .-CCCUCC UC**

**GUCGUUAUG CC UGACU UCAUCCUCU UGGG \**

**UAGUAGUAU gg gcugg aguaggagg ACCU C**

**C ca- a --^ \ ------ CC**

**zma-miRds5a AAAACUCUAGGUGGAGCAGCU**

**AU A u- ug .-u UC A U--| UU**

**CU CUCCAaaaacuc agg gagcagc CUGC CAG GUU AGA \**

**GA GAGGUUUUUGAG UUU UUUGUUG GACG GUC CAA UCU G**

**AC C UU UU \ - GU - CAC^ UU**

**zma-miRds5b AAAACUCUAGGUGGAGCAGCU**

**- CACC u .-CUGCU| GAU**

**GCUCCA aaaacucuagg ggagcagcu CCA U**

**CGAGGU UUUUGAGAUUC UUUCGUUGG GGU U**

**A ---- - \ -----^ AAA**

**zma-miRds5c AAAACUCUAGGUGGAGCAGCU**

**- ACUC gu .-GUUUAGAU -| A G**

**GCUCU Caaaacucuag ggagcagcu--CUGCUCCAAA UGUUU CUCGU ACUG U**

**UGAGA GUUUUGAGGUU CCUCGUCGA GACGAGGUUU AUAAA GGGCG UGAC G**

**C ACUC -- \ \ -------- U^ A U**

**zma-miRds5d AAAACUCUAGGUGGAGCAGCU**

**AAUACUACUC| U A C C A CU AUAC**

**AGUUGCUCUAC CUA GGG UU UUUGGC UGA UUGCU \**

**ucgacgaggug gau cuc aa aaACUG AUU GACGA U**

**AAAUCUCGUC^ - - - - - UC GAUC**

**zma-miRds5e AAAACUCUAGGUGGAGCAGCU**

**- CUA - gu u C .-AAUAGUUUA| UU**

**GCU CUCCa aaacucuag ggagcagc CUG UC GAUUGU \**

**CGA GAGGU UUUGAGGUU UUUCGUUG GAC AG CUAAUA C**

**A C-- C -- - C \ ---------^ CU**

**zma-miRds6 CACAGCACCGUUGGAUGUGGA**

**CACAUCCAACGGUGCUGCGUC**

**A C U c cA- A-| UCUUU A**

**GA CACACC UCcacauccaacggugcug gu GGGGGUUUU UAAAAUGU GCA C**

**CU GUGUGG agguguagguugccacgac cA CCCCCAAAA AUUUUACA UGU U**

**- A U a AUC AC^ ----- C**

**zma-miRds7 UAGGCUGUCUUCAAAUCCUGGG**

**U G-- cugu aauc----------- gg - .-AC| CCA**

**AAUUUC CCuagg cuuca cug UCC GCC UGGU \**

**UUAAAG GGGUCC GAAGU GAC GGG UGG ACCA U**

**C ACA U--- GUUAAAACCUCAAAU UU U \ --^ AAC**

**zma-miRds8 UCCGGGAGGUAGGAUCGACGUC**

**A --- U C .-UG -- A .-CACCCAGACCUA G - -| ACC**

**GGAGGAGC ACG UCG UCCUGCUUUCC GCUAG AGG GGGA UGUU UCGUC CC UCUC \**

**CUUUCUCG ugc agc aggauggaggg CGGUC UCC CCCU ACAA AGCAG GG AGAG A**

**- CAc - u \ -- GA A \ ------------ - A C^ AAG**

**zma-miRds9 AAGCAGAAAGCAGUGCUCUGAG**

**- GA aa- g u ga- CA- A---| C----- CAUAGUA U GC .-AUAG U UU**

**UUUGCA CAaagcag agca ugc cu gag UUUG GAUGG UUCAG UCAGAUAUUU GUG UAC UAG GU \**

**AAACGU GUUUUGUC UCGU AUG GA CUC AAGC UUACC GAGUC AGUCUAUAAA UAC GUG AUC CG U**

**A UC AAC A C ACC AGA AGUG^ UUCUUA ------- C UA \ ---- C GU**

**zma-miRds10a UCUAGAUCUAACGGACCAAA**

**UAU A - - .-aaAGAC .-CU| C**

**GGUU GAucuagauc uaacgg acca CUG UGCA G**

**UUAA CUAGAUUUGG AUUGUC UGGU GAU ACGU C**

**GAG C A U \ ------ \ --^ U**

**zma-miRds10b UCUAGAUCUAACGGACCAAA**

**C -| C C C CAU GAUCG- UUG UUU AAA AC**

**AAG CAG UCU UUGGUUCGUUAGAUCUAGAUC AGCCG GUAU UGGU UUAGG UUUUUUUAU GCUAC \**

**UUC GUC AGA aaccaggcaaucuagaucuAG UUGGC UAUG AUCG AAUUU AGGAGGGUG UGGUG U**

**- A^ - a A AU- AUAUAA UA- U-- A-- GA**

**zma-miRds10c UCUAGAUCUAACGGACCAAA**

**C| AGC C A C GU GUAAUUG UUG GG UAUAAA AU**

**AAGC UCU UUGGUCUGUUAGAUCUA GAUC A CGUAUGU UGGU UUAGG CUUUUU GCUAC \**

**UUCG AGA aaccaggcaaucuagau cuAG U GCAUACG AUCG AAUUU GGAGGG UGGUG G**

**-^ ACC a - A UG GUAUAA- UA- A- UGG--- GA**

**zma-miRds10d UCUAGAUCUAACGGACCAAA**

**----- A .-AC U- UCAUAUUU AACU**

**UGGU CGUUAGAUCUAGAUCCA CG GUG AG U**

**acca gcaaucuagaucuAGGU GC CAC UC A**

**AUAaa g \ -- UC UACCACCU CCAA**

**zma-miRds10e UCUAGAUCUAACGGACCAAA**

**C GC C A A C - .-AUUAUGG| AGG U**

**AAGCA UCU UUGGUCC UUGGAUCUA AUC AACCGUAUG UGUA UUUGUU AGUUUU U**

**UUCGU AGA aaccagg aaucuagau uAG UUGGCAUAU AUAU GAGCAA UCAAAA A**

**- AC a c c A G \ -------^ A-- U**

**zma-miRds10f UCUAGAUCUAACGGACCAAA**

**C CUC --| A C GUAAUCAUGGUCUG GC AAAU AC**

**AAGUAG UUUUGGUCCG GGAUU AGAUC AAUCGUAUGU UUAGA UUUUUUAU CUAC \**

**UUCAUC Aaaaccaggc ucuag ucuAG UUGGCAUACG AAUUU GGAGGGUG GGUG G**

**- UAU aa^ a A AUAUAAAUUGUA-- A- GU-- GA**

**zma-miRds10g UCUAGAUCUAACGGACCAAA**

**C CUC --| A C GUAAUCAUGGUCUG GC AAAU AC**

**AAGUAG UUUUGGUCCG GGAUU AGAUC AAUCGUAUGU UUAGA UUUUUUAU CUAC \**

**UUCAUC Aaaaccaggc ucuag ucuAG UUGGCAUACG AAUUU GGAGGGUG GGUG G**

**- UAU aa^ a A AUAUAAAUUGUA-- A- GU-- GA**

**zma-miRds11 AUGGAGUGGUUUGAGGAGGCU**

**- -| u ug a c A AG A**

**GAG AAGGGGAa ggag guuugagg gg uA AAUCUCUC UAUUCA A**

**CUC UUCCUCUU CCUC CAAACUCC CC AU UUAGGGAG AUAAGU A**

**U G^ U CU C A C CG U**

**zma-miRds12 CAAAGAGUAGAAACAGUGGCC**

**AAAU U .-AG| U**

**GGGC UGGGCCA AUGGCCCUAAA \**

**CUCG AUccggu UGCUGGGGUUU U**

**AAAU - \ --^ U**

**G---- G AGCC**

**UC UGCU \**

**ag auga c**

**gacaa - gaaa**

**zma-miRds13 UUGCUGUGCUCGACAAUUGGAU**

**A GU- uu g-- ug uC- UC-- - -| UUUG C GA**

**AUA UUGU gcugu cucgacaau ga UGU CAG GC AAUUG UUC CAUUA \**

**UAU AACG UGACA GAGUUGUUA UU ACA GUU CG UUGAC AAG GUAGU U**

**A AAU UU AAA GU CAA UAAU G U^ UA-- A GU**

**zma-miRds14 GGUCGAGUCGUUGCAAUAGUA**

**U -| C C C AG C UUUG AC AUC AGCUC G**

**GAC GAUUUC UAC AU GCAACGACUC UCGAUUUUGUGUUUA GUUGAUUUUGGAUGGU ACUAAA GAUU AUAGAAGCGGGCUGAAA GGCGU U**

**CUG CUAAAG AUg ua cguugcugag ggUUAAAACACAAAU CAACUAAAACCUACCA UGGUUU CUAA UAUCUUCGCCCGAUUUU UCGCA A**

**- U^ - a a cu A UAAA AA GAA CUUAC A**

**zma-miRds15 AUCGAUGUUGUGUGAAGGAGGAU**

**GUA AC G .-A AG .-AUAGUA ------ AG**

**GUC CCU UUAUACAGUAU UUCC AAGU GGUAACUAGGG GUAUGAG \**

**uag gga aguguguugua AAGG UUCG CCAUUGAUCCC CGUAUUU U**

**A-- ga - \ - G- \ ------ AGAGCA CA**

**ACAC- AG CCA**

**GUGUU GGU A**

**UACAA CCA C**

**gcuaC G- AAA**

**zma-miRds16 CUAUUAUUUAUCGUUUAGGAC**

**- UA C a .-CAAC .-AGUCUCU| CAU**

**CUU AUGU cuauuauuuaucguuu gga GAC AAAAUAUAG \**

**GAG UACA GAUAAUAAAUAGCAAA UCU CUG UUUUGUAUC U**

**G GC U A \ ---- \ -------^ UAC**

**zma-miRds17 UUCAGCAGUGACUCUGAUGAGA**

**A C UG U .-UUU AUUCCC- .-AAUU| A**

**CUUUUUG AGU CGUCAGA UUAUU GGCUUU UUGACCG UGGUC A**

**GGAGGAC UUa guagucu aguga cCGAAA AACUGGC ACCAG C**

**A - ga c \ --- CCCUUCA \ ----^ A**

**zma-miRds18 UAAAGUUGGUUUUCGGAUUUUU**

**A G CCUCAG- UG .-GU| A UG G**

**GGC CGGCCU CG GGCCGACU GCCU GGAUU GGCCGU U**

**UUG GUUGGA gc uugguuga UGGG CCUGG UUGGCA C**

**A - uuuuuag uu \ --^ A GU A**

**zma-miRds19 AGCGCUCGGCUGUUUGCAUG**

**CA - c u uu .-GCUAUAAAA .-ACCACCA| AAUC**

**GACG CAGCagcg ucggc gu gcaugG UGG CCUC G**

**CUGC GUUGUCGU AGUCG CG CGUACU ACC GGAG A**

**CA U - U U- \ --------- \ -------^ GGAU**

**zma-miRds20 UUGCCGACCCGUGCAAAAUCG**

**UA G c c C .-GC UG .-AU| C - AG**

**CUU AACUuug cgacc gugcaaaaucgUCCAAAACCGGAUUUGCU AAACUGGAUG GACA GCA GC ACGU UGG C**

**GAA UUGAAAC GCUGG CACGUUUUAGUAGGUUUUGGCCUAAAUGA UUUGAUCUAC CUGU UGU CG UGCA ACC C**

**AG G A C A \ -- GU \ --^ - C CU**

**zma-miRds21a UUUAAGGACCUAAAAAUGCAU**

**A A CA A .-CACACCAC - G -| G**

**AACUUUGAAAAU UAUUU AGGUCCUUGAACUU UUAAGUGA AAUGGU CG UAUG GUUUA A**

**UUGAAAUUUUua guaaa uccaggaauuuGAA AAUUCACU UUACCA GU GUGU CAGGU A**

**- c aa C \ -------- C A A^ A**

**zma-miRds21b UUUAAGGACCUAAAAAUGCAU**

**G C C | C GC**

**AACUUUGAAAAUGUAUUU UA GUCCUUAAACUUGUUAAGUGA--CG ACC \**

**UUGAAACUUUuacguaaa au caggaauuuGAAUAAUUCACU GC UGG A**

**- a c \ ^ - UG**

**zma-miRds21c UUUAAGGACCUAAAAAUGCAU**

**G A C G AU .-C -| AA G**

**AACUUU AAGAUGCAUUU UAGGUUCUUAAACUUG UAAGUG GUACCGCA AUGC CU ACUU U**

**UUGGAA UUUuacguaaa auccaggaauuUGAAC AUUCAC UAUGGUGU UACG GA UGAA A**

**- C a A CG \ - U^ -- A**

**zma-miRds21d UUUAAGGACCUAAAAAUGCAU**

**G C U .-GC CC | U G**

**AACUUUGGAAAUGCAUUU UA GUCCUUGAACUUGUUAAG CUUGUUCGUUUGUG GGAU--UAG GGGUCG \**

**UUGAAACUUUuacguaaa au caggaauuuGAACAAUUC GAACAAGCAAACAU CCUA AUC CUUAGC A**

**- a c \ -- AA \ ^ - A**

**zma-miRds22a UUUGGUUUGAAGAAUGAGCUA**

**10**

**UUAUA a gagc G .-UCUCAC| UCACUU**

**GGGUGuuugguuuga gaau ua UCCAUCU ACC U**

**CCCACAAACCAAACU CUUA AU AGGUAGG UGG U**

**AUUCC C ---- G \ ------^ UUUAUU**

**zma-miRds22b UUUGGUUUGAAGAAUGAGCUA**

**GGCUCG .-GU A G .-AAUGAAA GGACU G C---| ACCA**

**GGGCUAGU GC CGC UUGA CCCGUGUCGU CGUG CC GCAC \**

**CCUGaucg CG GCG AGCU GGGCAUAGUA GCGC GG CGUG A**

**CUACUA \ -- A - \ ------- AAGUU A UCAC^ AGUG**

**CGC---- AUU-- .-AGGAU AC G AC**

**UCAAA CGCC GGCUUUGC GCAC--GCAC CAC \**

**aguuu GUGG CCGAAACG CGUG CGUG GUG C**

**aguaaga gguuu \ ----- C- \ A AA**

**zma-miRds22c UUUGGUUUGAAGAAUGAGCUA**

**UUAU - a ga .-aGUCCAUCAUCUUCUCUCCUUUUUUUUGUUUAGUC.-A -| C CC**

**GGGG UGuuugguuuga gaau gcu CGUGGA UAG AAUGAG UGAU \**

**CCCC ACAAACCAAACU CUUA UGA GUACCU AUC UUACUC ACUA A**

**GAUU A C G- \ -----------------------------------\ - A^ C UU**

**zma-miRds22d UUUGGUUUGAAGAAUGAGCUA**

**UGA u a - G .-UCAUC .-CUCCUC| UUU**

**GGAGGU--Guuugg uuga gaau gagcua UCUA UUCUCA ACUA U**

**CCUCCA CAAACC AACU CUUA UUUGGU AGGU AAGAGU UGAU U**

**UUC \ U C G - \ ----- \ ------^ UUG**

**zma-miRds23 UUUUCUGGCGCAUCGAAUUUG**

**A| CUU cg c CUAA GU**

**CUUUGA uuuucugg cau gaauuugCUAUGUAU AUAUAC \**

**GAAACU AAAAGACC GUA CUUAAAUGAUACAUA UAUAUG U**

**C^ U-- AU A ---- UA**

**zma-miRds24a UUGGUGACCAGGGAAAUGGAG**

**C ------ - G UU - .-G| U U**

**CAUGGAUCCCCUCCAUUUCCCUGGUCACCAAA CCA GUAGGGU UG UGGUU UCA GAAUGAGG GG C**

**GUACCUAGGGgagguaaagggaccagugguuU GGU UAUCUUA AC AUCGA AGU CUUACUCU CU C**

**- AGUCGG G G UC C \ -^ U A**

**zma-miRds24b UUGGUGACCAGGGAAAUGGAG**

**C .-CCCCAC A -| UGUCAA**

**CAUGGAUCCCCUCCAUUUCCCUGGUCACCAAAUCAG AUAG AUCUGAGUA GC \**

**GUACCUAGGGgagguaaagggaccagugguuUGGUC UGUU UAGAUUUAU CG U**

**- \ ------ A A^ UUACCG**

**zma-miRds24c UUGGUGACCAGGGAAAUGGAG**

**G| A G CUCC**

**AGGGCUG UuuggugaccagggaaauggagGGG AUUC \**

**UCCCGAU AAACUACUGGUCCUUUUACCUCCCC UAAG C**

**G^ C - UACC**

**zma-miRds24d UUGGUGACCAGGGAAAUGGAG**

**G A a - .-A| AA**

**AG GUUGGUuuggug cc agggaaauggagG GGAAUCUCUUUCCUAUUCA \**

**UC CAACCAAACCAC GG UCCCUUUACCUCC CCUUAGGGGAAGGAUAAGU A**

**G C - A \ -^ AU**

**zma-miRds25a UUCUAUAAAGGAAGAAGUCUAG**

**A GA c a C .-UUCAA UGC .-G GA -| UU**

**ACAUA UGuu uau aaggaagaagucuagAUUUAAC AUUGAAC AGAUAUAUUU GGU GAGA GUA GUUU \**

**UGUGU ACAA AUA UUCCUUCUUCAGAUCUAAAUUG UAACUUG UCUGUAUGAA CCA UUUU CAU CAAG A**

**G A- - C A \ ----- --- \ - AA A^ AA**

**zma-miRds25b UUCUAUAAAGGAAGAAGUCUAG**

**A GA c a C .-UUCAAAGAUAUAU| AGAGAGUAG AUA UU AAC**

**ACAUA UGuu uau aaggaagaagucuagAUUUAAC AUUGAAC UUUGGGGUGG UUUUUAAAGAAC CAAUU UUUG \**

**UGUGU ACAA AUA UUCCUUCUUCAGAUCUAAAUUG UAACUUG AAACUUCACC AAAAGUUUUUUG GUUAG GGGC A**

**G A- - C A \ -------------^ AACAAAAAA AA- U- AGU**

**zma-miRds25c UUCUAUAAAGGAAGAAGUCUAG**

**A GA c a C .-UUCAA U .-GGU -- -| UU**

**ACAUA UGuu uau aaggaagaagucuagAUUUAAC AUUGAAC AGAUAUA UUUGG GGAGAGA GUA GUUU \**

**UGUGU ACAA AUA UUCCUUCUUCAGAUCUAAAUUG UAACUUG UCUGUAU GAACC UUUUUUU CAU CAAG A**

**G A- - C A \ ----- - \ --- AA A^ AA**

**zma-miRds26 AAAAGAUUAAGCUGAUGGGAGA**

**G GG U G AC UC CUU AG---- .-CU| GCA**

**UG CGA CUUUUU CAU CGG UAGUUUUUU UUUGAA GGUAC GGAA \**

**AC GUU GAagag gua guc auuagaaaa AAACUU CCAUG CCUU A**

**A AG - g -- ga CU- AACUAA \ --^ AAU**

**zma-miRds27 GUCUUUAUGCUGUAGGAGAGGGU**

**G CGCAG -| AC UA U**

**GGUCAGG UC CUCC GCGGC GGAGACGUCGCA G**

**UUAGUCC gg gagg ugucg uuucugUAGCGU G**

**- CCAug a^ a- ua G**

**zma-miRds28 UACAUAUGUUGUCUGCCUCACU**

**-| c CA A --- AG**

**UCGAAUGUUCuacauauguugucug cucacuUG GCA CACU UUCUCA \**

**AGCUUACAAGAUGUAUACAACAGAC GAGUGAAC UGU GUGA AAGAGU C**

**A^ A -- - CUU CU**

**zma-miRds29 AGCUGGCUGUGGAAAAAAGCUG**

**GG| UGUUU U G G UUU G**

**GCC GGU CGGCUUUUUUCU AC AGCU CU A**

**CGG UCG gucgaaaaaagg ug ucgg ga A**

**GG^ UG--- - - - uc- a**

**zma-miRds30 AUGGAGUGGAUUGAGUGGGCU**

**AUCAAA| u ug A C A**

**GGUGauggag ggauugag ggcuAGAAUCC UCACUA UCA U**

**CCAUUACCUC CCUAACUC CCGAUUUUAGG AGUGAU AGU U**

**UAUUUC^ C CU G A U**

**zma-miRds31a UUUGUUAAUGUUUGGAGUAGC**

**-- GGA- C ACACACC CC - U- AA--| A**

**UCU UAAUUuuguuaauguuuggaguagc CCAUCAAAG AGUUC GU GU UCC AGCC C**

**GGA AUUAAAACAAUUACAAACCUCAUCG GGUAGUUUC UUAAG CG CA AGG UCGG C**

**CG AACA C CAAAU-- A- U UU ACCA^ A**

**zma-miRds31b UUUGUUAAUGUUUGGAGUAGC**

**C U-| G UU AAUUCUG AA .-GU U**

**UUUG UAAUuuuguuaauguuuggaguagc CCAUCAAAGG UA CAGU UCCUG AGCC G**

**AGAC AUUAAAACAAUUACAAACCUCAUCG GGUAGUUUCU GU GUCA AGGGC UCGG G**

**A CU^ G GU G------ -- \ -- U**

**zma-miRds31c UUUGUUAAUGUUUGGAGUAGC**

**- GGA- C A .-AC| CAG**

**UCU UAAUuuuguuaauguuuggaguagc CC UCAAAG ACAC U**

**GGA AUUAAAACAAUUACAAACCUCAUCG GG AGUUUC UGUG U**

**G AAUA C C \ --^ CCC**

**zma-miRds31d UUUGUUAAUGUUUGGAGUAGC**

**CUUUAU| G UUUAAAAUC G - .-GU U**

**UAAUuuuguuaauguuuggaguagc CCGUCAAAGG UGCA UAGU CCUG AGCC G**

**AUUAAAACAAUUACAAACCUCAUCG GGUAGUUUCU GUGU GUCA GGGC UCGG G**

**AGACCU^ G --------- G A \ -- U**

**zma-miRds31e UUUGUUAAUGUUUGGAGUAGC**

**C U-| G UUUAAAUUC G - .-GU U**

**UUUG UAAUuuuguuaauguuuggaguagc CCGUCAAAGG UACA UAGU CCUG AGCC G**

**AGAC AUUAAAACAAUUACAAACCUCAUCG GGUAGUUUCU GUGU GUCA GGGC UCGG G**

**A CU^ G --------- G A \ -- U**

**zma-miRds31f UUUGUUAAUGUUUGGAGUAGC**

**-- GGA- C A ACACACC CCGU U- AA--| A**

**UCU UAAUuuuguuaauguuuggaguagc CC UCAAAG AGUUC GU UCC AGCC C**

**GGA AUUAAAACAAUUACAAACCUCAUCG GG AGUUUC UUAAG CA AGG UCGG C**

**CG AACA C C CAAAU-- AUGU UC ACCA^ A**

**zma-miRds31g UUUGUUAAUGUUUGGAGUAGC**

**C U-| G UUUAAAUUU G - .-GU U**

**UUUG UAAUuuuguuaauguuuggaguagc CCGUCAAAGG UGCA UAGU CCUG AGCC G**

**AGAC AUUAAAACAAUUACAAACCUCAUCG GGUAGUUUCU GUGU GUCA GGGC UCGG G**

**A CU^ G --------- G A \ -- U**

**zma-miRds31h UUUGUUAAUGUUUGGAGUAGC**

**-- GGA- C A .-AC| CAG**

**UCU UAAUuuuguuaauguuuggaguagc CC UCAAAG ACAC U**

**GGA AUUAAAACAAUUACAAACCUCAUCG GG AGUUUC UGUG U**

**CG AACA C C \ --^ CCC**

**zma-miRds32a AACGGACCUGUAGAUAUGGUA**

**CUUU -- G AA GC----- A CG .-C| UC**

**ACUA UACUAUGU UACA UCCG ACUU AAG GUA UCCC C**

**UGAU augguaua augu aggc UGAG UUC CAU AGGG C**

**CGUU CU g cc aaCCUCU A AU \ -^ AC**

**zma-miRds32b AACGGACCUGUAGAUAUGGUA**

**CUUU -- G AA GC----- A CG .-C| UC**

**ACUA UACUAUGU UACA UCCG ACUU AAG GUA UCCC C**

**UGAU augguaua augu aggc UGAG UUC CAU AGGG C**

**CGUU CU g cc aaCCUCU A AU \ -^ AC**

**zma-miRds32c AACGGACCUGUAGAUAUGGUA**

**CUUU -- G AA GC----- A CG .-C| UC**

**ACUA UACUAUGU UACA UCCG ACUU AAG GUA UCCC C**

**UGAU augguaua augu aggc UGAG UUC CAU AGGG C**

**CGUU CU g cc aaCCUCU A AU \ -^ AC**

**zma-miRds32d AACGGACCUGUAGAUAUGGUA**

**CUUU -- G AA GC----- A CG .-C| UC**

**ACUA UACUAUGU UACA UCCG ACUU AAG GUA UCCC C**

**UGAU augguaua augu aggc UGAG UUC CAU AGGG C**

**CGUU CU g cc AACCUCU A AU \ -^ AC**

**zma-miRds33 UUCAAUGUAGAAGAAUUUUCAU**

**G CCA UU - U .-C AAAAA .-AAAAAUCAAUUCUUAAAAAA C-| AU UA**

**GAU CUAGGAUGGG UUC UUUAU UUGA ACCU UGC AUUG AAGA UGCUU U**

**CUA GAUUUuacuu aag agaug aacu uGGG ACG UAAC UUCU ACGAA A**

**G A-- uu a u \ - GG--- \ -------------------- UA^ C- UA**

**zma-miRds34a UUUGAAAGGGAAGUAGGGUUUA**

**G C C C - G .-CA AG- C-| C CUAU UG**

**GGC AGU AGUG AUUUU CUU CUUUUUCA CU GAUGCUUUG UUGA AAGGG CGU \**

**CCG UCA UCau uggga gaa gggaaagu GA CUAUGAAAC AACU UUCUC GCG C**

**A U - u u - \ -- GCA UU^ - CUU- AA**

**zma-miRds34b UUUGAAAGGGAAGUAGGGUUUA**

**G C C C - G .-C C-| C CUAU UG**

**GGC AGU AGUG AUUUU CUU CUUUUUCA ACUAG--GAUGCUUUG UUGA AAGGG CGU \**

**CCG UCA UCau uggga gaa gggaaagu UGAUC CUAUGAAAC AACU UUCUC GCG C**

**A U - u u - \ - \ UU^ - CUU- AA**

**zma-miRds34c UUUGAAAGGGAAGUAGGGUUU**

**G C C C - G .-C C-| C CUAU UG**

**GGC AGU AGUG AUUUU CUU CUUUUUCA ACUAG--GAUGCUUUG UUGA AAGGG CGU \**

**CCG UCA UCau uggga gaa gggaaagu UGAUC CUAUGAAAC AACU UUCUC GCG C**

**- U - u u - \ - \ UU^ - CUU- AA**

**zma-miRds34d UUUGAAAGGGAAGUAGGGUUU**

**G C C C - G .-C C-| C CUAU UG**

**GGC AGU AGUG AUUUU CUU CUUUUUCA ACUAG--GAUGCUUUG UUGA AAGGG CGU \**

**CCG UCA UCau uggga gaa gggaaagu UGAUC CUAUGAAAC AACU UUCUC GCG C**

**- U - u u - \ - \ UU^ - CUU- AA**

**zma-miRds34e UUUGAAAGGGAAGUAGGGUUU**

**G C C C - G .-C C-| C CUAU UG**

**GGC AGU AGUG AUUUU CUU CUUUUUCA ACUAG--GAUGCUUUG UUGA AAGGG CGU \**

**CCG UCA UCau uggga gaa gggaaagu UGAUC CUAUGAAAC AACU UUCUC GCG C**

**A U - u u - \ - \ UU^ - CUU- AA**

**zma-miRds34f UUUGAAAGGGAAGUAGGGUUUA**

**G C C C - G .-C C-| C CUAU UG**

**GGC AGU AGUG AUUUU CUU CUUUUUCA ACUAG--GAUGCUUUG UUGA AAGGG CGU \**

**CCG UCA UCau uggga gaa gggaaagu UGAUC CUAUGAAAC AACU UUCUC GCG C**

**A U - u u - \ - \ UU^ - CUU- AA**

**zma-miRds35 UCACAAUCGAUUGGACUAAAA**

**U GA GC U C .-AUGU| U U UC**

**UCU UGUCAUUUUAGUCCAAUCG UGUGAACA GAC UGG GGCU UGCCA CGUG \**

**AGG ACAGUaaaaucagguuagc acacuUGU CUG ACC CCGG ACGGU GCAC G**

**- UC ua C A \ ----^ U - CU**

**zma-miRds36 UAAAGUGUCGACUAUUUUGGG**

**GACCCAUA aa u g - .-CAC| AU AGAAA**

**GCu agug c acuauuu ugggA GGGGAGU GC C**

**CGA UCGC G UGAUAGA ACCUU CCCCUUA UG C**

**AUGUAAAA G- U - U \ ---^ CU ACAUU**

**zma-miRds37a UAUCUGGAGGAAGAAUCGCUA**

**A UCAUGCC - G UUA AG-------- .-GAA| UA**

**GGACUGG UAGC AUUCU UCUUCUA AUAGGA GAAGG UGA \**

**UUUGACC aucg uaaga aggaggu uauCCU CUUUC ACU G**

**- CUU---- c - c-- AAAGUACUAA \ ---^ CA**

**zma-miRds37b UAUCUGGAGGAAGAAUCGCUA**

**A UCAUGCC - G UUA .-AGGAA| GAAG**

**GGACUGG UAGC AUUCU UCUUCUA AUAGGA GGG \**

**UUUGACC aucg uaaga aggaggu uauCCU CUC A**

**- CUU---- c - c-- \ -----^ AGAU**

**zma-miRds37c UAUCUGGAGGAAGAAUCGCUA**

**A UCAUGCC - G UUA .-AGGAA| GAAG**

**GGACUGG UAGC AUUCU UCUUCUA AUAGGA GGG \**

**UUUGACC aucg uaaga aggaggu uauCCU CUC A**

**- CUU---- c - c-- \ -----^ AGAU**

**zma-miRds38 GUAUCUAGAAAAGCUAAAACGA**

**AUAUUC -| C U GAAC A G**

**UAGGUUG UUUGGUUUUUCUAGAUAC UAG UUUUGUUAU CUAG UAUA \**

**AUCUagc aaaucgaaaagaucuaug GUU AAAAUAAUA GAUC GUAU G**

**UUAAAU a^ A C AAUA C U**

**zma-miRds39 AAAGCUAAAAUCAAGUCGGUG**

**UUGGG C U C- ------------- CU GA CU .-AU .-CUGAAAUCAUUAUG| UUU**

**UGU CCACUGG CUU UUUUAGU GU UAGU AAAGU AUC GACCCUUG GGUGAGA A**

**GCG Gguggcu gaa aaaaucg CA AUCA UUUCG UGG UUGGGAAU CCAUUCU U**

**GAAUA C - cu aaaAUACUAAAGA AC AG -- \ -- \ --------------^ CAC**

**zma-miRds40a AUGUUAUGAUGCAUGCUUCGUU**

**AGCUCA U - - A CGC .-GAA| C**

**AGGA AUG GGU UGCAUCG GACAU UCAUU GGA A**

**UCCU ugc ucg acguagu uugua AGUAA CCU U**

**CGCUUA u u u a --- \ ---^ A**

**zma-miRds40b AUGUUAUGAUGCAUGCUUCGUU**

**CAGAAGCUCA U - - A C- AU---- .-CACAUCUAGA .-AUUG CU-| C C UC GU CU**

**AGAA AUG GGC UGCGUCG GACA CACUC UGAAAGA CUCU CAG GCU CAC UGCG GGAGGU GCGA G**

**UCUU ugc ucg acguagu uugu GUGAG AUUUUCU GAGG GUC CGA GUG ACGU CCUCCG UGUU C**

**CGCUUA---- u u u a aA AAAACU \ ---------- \ ---- UAC^ A C CA UC AU**

**zma-miRds40c AUGUUAUGAUGCAUGCUUCGUU**

**U C CUCAA UAU - A C- .-AUUGAA A .-UAAACUCU UG CU- C C -| C U ACU**

**GC AGUAG GAA GGGU UGCGUCA GACA CACUC GGACAC UC GC CAG GCU CAC UGU GU GGAGGUG GCG C**

**CG UUAUC CUu uucg acguagu uugu GUGAG CCUGUG AG CG GUC CGA GUG ACG CA CCUCCGU CGU C**

**- C ----- ugc u a aA \ ------ G \ -------- UA UAC A C U^ - C CAU**

**zma-miRds41 AUGACUAGUAGAGUGGCACUUCA**

**.-UGGAUCACACa g ag| -- - - C GAAACAACACAUCAU .-C GCAUAGCA AU AAUA- UG AAA**

**ugacua uag uggcacu uc aC UUUGCAA ACACU GCAUUUG CCUGU UCUUUUUUA CUU CA UUACU G**

**AUUGAU AUU AUCGUGA AG UG AGAUGUU UGUGA UGUAAAU GGACA AGAGAGGGU GAG GU GAUGA U**

**\ ----------- G G-^ AU A U - --------------- \ - AGAG---- GG GAGCA GU AAA**

**zma-miRds42a UGGAUUAUAUAAUCUGGGUAG**

**AAAA| A U A A AC**

**UAUAAUCUGCCCA AUUAUAU AUCCAA AAUUUUU AACU U**

**AUAUUGgaugggu uaauaua uagguU UUAAAAA UUGA A**

**UCUA^ c u G A CC**

**zma-miRds42b UGGAUUAUAUAAUCUGGGUAG**

**A| A A G C**

**GAUUAUAAU UAUCCA AUUAUAUAAUC AAUAAUUUUUGAACUAA \**

**CUAAUAUUA augggu uaauauauuag UUAUUAAAAACUUGAUU A**

**C^ G c g C**

**zma-miRds42c UGGAUUAUAUAAUCUGGGUAG**

**G| C C C**

**GGAUUAUAAUCUACCUAGAUUAUAUAAUCCAA AA UUUUGAACUAA \**

**CUUAAUAUUAgaugggucuaauauauuagguU UU AAAACUUGAUU U**

**-^ A A C**

**zma-miRds42d UGGAUUAUAUAAUCUGGGUAG**

**G| C C C**

**GGAUUAUAAUCUACCCAGAUUAUAUAAUCCAA AA UUUUGAACUAA \**

**CCUAAUAUUAgaugggucuaauauauuagguU UU AAAACUUGAUU U**

**-^ A A C**

**zma-miRds42e UGGAUUAUAUAAUCUGGGUAG**

**UGU------- CCAU UG UUUUGG**

**UUACCC GGAUUAUAUAAUC GAUUAU A**

**gauggg ucuaauauauuag CUAAUA G**

**UGUUUGUCCU ---- gu UAUUAG**

**zma-miRds42f UGGAUUAUAUAAUCUGGGUAG**

**G| C C G**

**GGAUUAUAAUCUACCCAGAUUAUAUAAUCCAA AA UUUUGAACUAA \**

**CCUAAUAUUAgaugggucuaauauauuagguU UU AAAACUUGAUU A**

**-^ A A G**

**zma-miRds42g UGGAUUAUAUAAUCUGGGUAG**

**G| A U G AC**

**AGAUUAUAAUCUGCCCA AUUAUAU AUCCAAUAAUUUUU AACU U**

**UCUAAUAUUAgaugggu uaauaua uagguUGUUAAAAA UUGA A**

**-^ c u A CC**

**zma-miRds43 UAUCGUAAUGUUGCAAACAGAA**

**AC---| GAA A**

**--UUGCUGAGGUGcgggucgcugagacacugcuGCAACGGACUUCGAAUGUUCUACAUAUGUUGUCUGUCUCACUUG ACACU UUCUCAG \**

**AACGACUCCACGUCCAGCGACUCUGUGACGACUGUUGCCUGAAGCUUACAAGAUGUAUACAACAGACGGAGUGAAC UGUGA AAGAGUU G**

**\ GUCGU^ --- C**

**zma-miRds44 UUGUAUUCAUUGAUCUCAGAAG**

**GAAUG A GG AGCG A .-UAGUCCAUUCUAA| U C CAA**

**GCC UCUU UUGA GGUGAGU GCAG AAAGA AAU UGA A**

**CGG Agaa gacu uuacuua uguu UUUCU UUA ACU A**

**ACAAA A -- cuag - \ -------------^ C - ACA**

**zma-miRds45 AAGCUUUGGGCAGCUCGGACA**

**- A-| CC aAC GUG G G**

**CGG GC CAaagcuuugggcagcucggac CU GAA CCGGA U**

**GCC CG GUUUCGAAACUCGUCGAGCCUG GA CUU GGUCU C**

**G GA^ A- C-- AG- G C**

**zma-miRds46 UACUCUAUAAGGACUAGUUUG**

**AG a ------------| C U AUGA**

**GGGAAACAuacucu uaaggacuaguuugGGAACCUCAU UUUUCCAAGGGAUUU UAUUUUCUCA GGGAAA \**

**CCCUUUGUAUGAGA AUUCCUGAUCAAACCCUUGGAGUA AAAGGGUUCCCUAAA GUAAAAGGGU CUCUUU A**

**GA G AAAGGGUUAAUA^ A U AGUC**

**zma-miRds47 UCAACACAUGUGGAUUGUGGUC**

**A| U CC G A G**

**CUCUA UAUUGAUCACAAUCUACAUGUGUUGAGGUGGA GA GUGUAA UUA \**

**GAGAU AUAGcugguguuagguguacacaacuCUACCU CU CACAUU AAU U**

**-^ C AA G G U**

**zma-miRds48 UAAGGGUCUGUUUGGUUUUU**

**--- A- GUua uuG CUA UA -------------| CG GA**

**CU UUCGU agggucuguuugguuu UACCUAA UGUCA CUUUGUCUA AGGUUAGU UUC \**

**GA GAGUA UUCCGGAUAAACCAAA AUGGAUU ACGGU GAAACGGAU UCCAAUCA AAG A**

**CAU GG AA-- CG- AA- GC UGAAAAGAUAGAU^ AG UU**

**zma-miRds49 AAGUGGACAGGCUAAAACACAU**

**A C C A U C --| C G**

**AAUAA CUAU UGUGUUUUAGCC GUCCACUUGAAGGUGAU GAUUGCUCUAA ACU UUGG UGUUAAA A**

**UUAUU GAUA acacaaaaucgg caggugaaCUUCCGCUA CUAACGAGAUU UGA AAUU AUAGUUU U**

**- U u a U C UU^ - U**

**zma-miRds50 UUAGAUUAUAGUAGAAGAGUA**

**-| A GAAGA C CA UA**

**AUUAUAUAAGuu gauuauaguagaagaguaG UAAAAUAUCA UUUGGUA ACAAAUAAGC \**

**UAAUAUAUUCAA CUAAUAUUAUCUUCUCAUU AUUUUAUAGU AAACCAU UGUUUAUUCG G**

**C^ C AUACA A AG AC**

**zma-miRds51a CAAACAUGCUGUGGAUAGGUU**

**A A A--| GUGA UG A A - UUC CAC G**

**GA UGA AGCCUA U CA GCA GUUUG GAAUUU AGG GGG A**

**CU ACU uuggau g gu cgu caaac UUUGAA UCU UCC G**

**A G AGG^ a--- gu - a A --- UUA C**

**zma-miRds51b CAAACAUGCUGUGGAUAGGUU**

**A A A--| GUGA UG A A - UUC CAC G**

**GA UGA AGCCUA U CA GCA GUUUG GAAUUU AGG GGG A**

**CU ACU uuggau g gu cgu caaac UUUGAA UCU UCC G**

**A G AGG^ a--- gu - a A --- UUA C**

**zma-miRds51a UAUGCAACAAGUAGAUUCCUGA**

**C CA GCCCUAG AC A - .-A .-GCCUUAU G | CGA**

**GGU AU UAG AUC ACUUGUU CAUAA CUCCA CUAC CUGCU--UUACC \**

**CCA UA guc uag ugaacaa guauU GAGGU GAUG GGCGG AGUGG C**

**- AG AAUa--- cu a c \ - \ ------- A \ ^ ACC**

**zma-miRds51b UAUGCAACAAGUAGAUUCCUGA**

**C CA GCCCUAG AC A - .-AA .-GCCUUAU G | CGA**

**GGU AU UAG AUC ACUUGUU CAUA CUCCA CUAC CUGCU--UUACC \**

**CCA UA guc uag ugaacaa guaU GAGGU GAUG GACGG AGUGG C**

**- AG AAUa--- cu a c \ -- \ ------- A \ ^ ACC**

**zma-miRds53 AGAUGAUGGACUAGCUUAUUCA**

**AG AGAA- u a .-caCUAAACCAAACACCCUAUUAGACUAUCUCCAACAAUAUCCUCUAUAUUUAUA CAACUU .-AUA| AA**

**GUGUG ga gauggacu gcuuauu CCCUCAU GUAU GGCCG GGGGC G**

**CACAC CU CUACCUGA CGGAUAA GGGAGUA CGUG CUGGU CCCCG A**

**AA GUGUG - C \ -------------------------------------------------UAA-- CCCC-- \ ---^ CC**

**zma-miRds54 AUAAUUUGUGUGGUUGUUUGGUU**

**UAUUC A CCU C UG GGA .-AAGUAAUAAUAUCAUU|UG**

**CAG UCAAUCA GC GC CAUAAGU UGC CCG U**

**GUC AGuuggu ug ug guguuua AUG GGU G**

**UGCAU - u-- u gu AUA \ ----------------^UU**

**zma-miRds55 CGAAUACUGGAUGUAGGGACGUU**

**U U CCACU| AAC UA- G - ---------- A**

**UUC GGU UGU CCCUAC C AGUAUU GGU UAGCA U**

**AAG CCA gca gggaug g ucauaa cCG GUUGU G**

**C C UUuu-^ --- uag - g CUGAACGAUG G**

**zma-miRds56a AUCUGAUGGAGUGAUGGCGAU**

**CAUCUC-- CCU GA| G CG CUC**

**ACA CCAU AC UCCA UCGGGUCGU \**

**UGu ggua ug aggu agucuaGUA C**

**UCUUCAUA agc g-^ - -- CCA**

**zma-miRds56b AUCUGAUGGAGUGAUGGCGAU**

**A A .-AGCCCCC -| UCC UAAG**

**AGAAGCAUGCAUCGCCAUCACUCCAUCAGAUCGUGGUGGAGAC ACCC GAGG GUG AAG \**

**UCUUCGUACGuagcgguagugagguagucuaGUACCACCUCUG UGGG CUCC CAC UUC C**

**- C \ ------- A^ CC- CUCU**

**zma-miRds56c AUCUGAUGGAGUGAUGGCGAU**

**A A .-GA| AA**

**AGAAGCAUGCAUCGCCAUCACUCCAUCAGAUC UGGUGGAGACGACCC CAUG C**

**UCUUCGUACGuagcgguagugagguagucuaG ACCACCUCUGUUGGG GUAC A**

**- C \ --^ CU**

**zma-miRds57 UAUCAUGACUGAUUGGGUUCG**

**- C u - C CCA .-UGUAUC| A U UAUU**

**UGAAUGG UGuaucaugacugau ggg uucgU GGCCCUGU CAAGU GUG ACGA GCC G**

**ACUUAUC ACAUAGUACUGACUA CCU AAGUA CCGGGACA GUUUA CAC UGUU CGG U**

**U A U A - A-- \ ------^ C C UUGU**

**zma-miRds58a ACAUUUUAAAUCUGGGACAUC**

**- A a C C C C G GAUU ------------| AC**

**GCUCA GUGCacauuuuaaaucugggac uc AUCU ACAUC AGCGG AGCA UUAAUUU AAGAACC CCUCCC \**

**CGAGU CACGUGUAAAAUUUAGAUCUUG AG UAGG UGUAG UUGUC UCGU GGUUGAA UUCUUGG GGAGGG C**

**A A C U U U U - AUAU AAUAAAGUGGAA^ AA**

**zma-miRds58b ACAUUUUAAAUCUGGGACAUC**

**- c CAG AGC CACC-| G CC CCU**

**GCUCACGUGCacauuuuaaaucugggacau CAUC CAUCUAAC AG AAUU UAUAAAGAACUCCUUCCA UC C**

**CGAGUGCACGUGUAAAAUUUAGACUCUGUA GUAG GUAGGUUG UC UUAA AUGUUUUUUGAGGAGGGU AG C**

**A A AAA CCA CAAGA^ A -- UAC**

**zma-miRds58c ACAUUUUAAAUCUGGGACAUC**

**A ------- CA .-CGACAGCACCA| A**

**CUCACG UGCacauuuuaaaucugggacaucCAUCU CAUCUAA GUUUU U**

**GAGUGU ACGUGUAAAAUUUAGAUCCUGUAGGUAGA GUAGGUU CAAGA A**

**C ACGUUUG AC \ -----------^ A**

**zma-miRds59a ACUUAGGAACGGAGGGAGUAC**

**UAGAAAAUAA U .-AAGCAAAUU .-AAAAAUCU| GU**

**GUACUCCCUCCGUUCCUAAGUCUCCA CGUAU GGA--UUUAUUC CCAUC A**

**caugagggaggcaaggauucaGAGGU GCAUA CCU AAAUGAG GGUGG U**

**UAGAACACUA C \ --------- \ \ --------^ AU**

**zma-miRds59b ACUUAGGAACGGAGGGAGUAC**

**AAUCUU U G U .-C G CA ACCA .-A| CAA CC**

**GUGA GUACUCCCUCCGUUCCUAAGUCUCCA CGUAU AGU AUCU CA GUG AGG GCGG AGGAGCG G**

**UAUU caugagggaggcaaggauucaGAGGU GCAUA UCG UAGA GU CAU UCC CGCU UUUUCGU A**

**AUCUUU - A U \ - G AG AA-- \ -^ CA- AU**

**zma-miRds60a UUAUGCAACAAGUAGAUUCCUG**

**AAUGCCCUAG A A - .-ACUCCAGCC| AC UUU**

**UAG AAUC ACUUGUU CAUAA UUAUCU GUUG \**

**guc uuag ugaacaa guauu AGUGGA CAGC U**

**CAAGUAAAUA c a c \ ---------^ CC CCA**

**zma-miRds60b UUAUGCAACAAGUAGAUUCCUG**

**AAUGCCCUAG AC A - .-A .-GCCUUAU G | CGA**

**UAG AUC ACUUGUU CAUAA CUCCA CUAC CUGCU--UUACC \**

**guc uag ugaacaa guauu GAGGU GAUG GGCGG AGUGG C**

**CAAGUAAAUA cu a c \ - \ ------- A \ ^ ACC**

**zma-miRds61a UGGUUGACAUAUGGACCCCAC**

**GGGGUCCAUAUGUCAACCACG**

**- C AC --| CA UG CGA**

**AAAUGGGUCGugguugacauauggaccccacAUGUUAG CUGUGUAUC UUUUCUC UCUUUUC CG UC U**

**UUUGCUCAgcaccaacuguauaccuggggUGUAUAGUC GACACAUAG AAAAGAG AGAAAAG GC AG G**

**G A AU AC^ A- CA AGA**

**zma-miRds61b UGGUUGACAUAUGGACCCCAC**

**GGGGUCCAUAUGUCAACCACG**

**AAACGA| AUC UA UG - - UCU**

**GUCGugguugacauauggaccccacAU AGUCUGUGUAUC UUUUCUC UCUUUUCU CG GUUC \**

**CAgcaccaacuguauaccuggggUGUA UCGGACACAUAG AAAAGAG AGAAAAGG GC CAGG C**

**UUUACC^ CAA UG -- U A CUA**

**zma-miRds61c UGGUUGACAUAUGGACCCCAC**

**C A c G CCCUCUGU AA C .-A| UCUC**

**AAUGAGUC ugguugacauaugga cccacAUGUCAU CA UCU UUUCU UCUCUUUU UCGGU U**

**UUGCUCAG ACCAACUGUAUACCU GGGUGUAUAGUA GU AGA AAGGA AGAGAAAA GGCCA C**

**U C U - ACAUAU-- GA A \ -^ CUCU**

**zma-miRds6d UGGUUGACAUAUGGACCCCAC**

**AAACA| CUUUUCUAUCUCUUUUCC UUCUCU**

**GGUCGugguugacauauggaccccacAUGUCAGCAUGUAUCU CGG C**

**CCAGUACCAACUGUAUACCUGGGGUGUACAGUCGUACAUAGG GCC U**

**UUUAC^ UUU--------------- CUCCAC**

**zma-miRds61e UGGUUGACAUAUGGACCCCAC**

**GGGGUCCAUAUGUCAACCACG**

**- A | A**

**AAAUGGGUC ugguugacauauggaccccacAUGUCAGCAUGUAU--CC A**

**UUUGUCCAg accaacuguauaccuggggUGUACAGUCGUACAUA GG A**

**G c \ ^ C**

**zma-miRds61f UGGUUGACAUAUGGACCCCAC**

**-| A u c G - U CUCUCUCUUU**

**AAAC GGUCGugg ugacauauggaccc acAUGUCAG C UGUGUAUCUCUUUUCUCUC CC \**

**UUUG CCAGCACC ACUGUAUACCUGGG UGUAUAGUC G ACACAUAGAGAAAAGAGAG GG U**

**G^ C U U - U U CCUGUACAUC**

**zma-miRds62 UUGCAAUGACAGGACGAGACG**

**AAAUAUUC UA A C UAA .-A| C - AA AAA**

**CU UC UC UUCUGUCAU UGCAACUAAA UAAA AAGGAC AAA AUA \**

**GA ag ag aggacagua acguuGGUUU AUUU UUCUUG UUU UAU U**

**CACUAGCA gc - c --- \ -^ - A AG AAU**

**zma-miRds63 UGAUCUUGCUUGUAGAAGAUGU**

**G - .-u c c g .-gu - UU ---------------| AUCCUU**

**CC UUCUUCC gau uug uugua aagau UGUGG CA AUCAUCA CAAGUGGAC \**

**GG AAGAAGG CUA AAU AAUAU UUCUA ACACC GU UGGUAGU GUUCACUUG A**

**A A \ - A - G \ -- U C- CUUGAUGUUACCUUC^ AUAAAC**

**zma-miRds64 AUUUGGCAAAAUGGGUCGCUC**

**GAGG----- A UU UU**

**AGGGCGACCCAUUUUG CAAAU CUC \**

**Ucucgcuggguaaaac guuua GAG C**

**GUCGUUGAA g GG CC**

**zma-miRds65 UCUGAUCCAUUAUAUUACGAC**

**- - u c .-ACAGAUACAAAGAAGG|UG AG UU UAA**

**AAGCAAACUAucugau ccauua auuacga CCAACC UU UUA GGUUU UA \**

**UUCGUUUGGUAGACUA GGUAAU UAAUGCU GGUUGG AG GGU UCGAG AU A**

**G G C A \ ----------------^GU GG UU UAG**

**zma-miRds66 UAUAAGUUGGAUUAUGAUGGA**

**- .-CUAUAU ga AUUA - - A - AAA--- U C--| CU**

**CCUG uauaaguuggauuau uggaAG GG AA GUAAAAUAU AC CU AUAAGU AUC AAUA A**

**GGAC AUAUUUAAUCUAAUA AUCUUC CC UU CAUUUUAUA UG GA UAUUUA UAG UUAU G**

**A \ ------ AC CUA- A C G A CUCGGG U ACU^ UU**

**zma-miRds67a UUCACAAGUUUAGGGACCUGGA**

**C| G CG C A U CU UC GUA AA**

**AAAGUGC CAUCCAGGUCCCUAAACUUGUGAAGUUGUAUCAUCU GUCC UAAACU UCAA GUGCACAUUU GUC UAUACUU UGGUUGUGUCAUCUG U**

**UUUCACG GUagguccagggauuugaacacuuCAAUAUAGUAGG CAGG AUUUGA AGUU UAUGUGUAAG CAG AUAUGGA ACCAAUACAGUAGAC A**

**-^ A AU A G U AC GA GAA CC**

**zma-miRds67b UUCACAAGUUUAGGGACCUGGA**

**C U A CUA C AAUA -| G C CUCUU A CA**

**AAAGUGC CAUCCAGGUCCCUAAACUUGUGAAGUU UAUCAUC GUCCUUAAACU UCAA CACA UUCUG UC UUAUAC UGGUU UGUCAUCUGGGUC U**

**UUUCACG GUagguccagggauuugaacacuuCAA AUAGUAG CAGGGAUUUGA AGUU GUGU AAGAC AG GAUAUG ACCAA ACAGUAGACUUAG A**

**- C C AGC U ACAC A^ - A AACAU C AG**

**zma-miRds67c UUCACAAGUUUAGGGACCUGGA**

**C U UGC A C C AAUA -| G C CUCUU A CA**

**AAAGUGC CAUCCA CCCUAAACUUGUGAAGUU UAUCAUC CGGUCCUUAAACU UCAA CACA UUCUG UC UUAUAC UGGUU UGUCAUCUGGGUC U**

**UUUCACG GUaggu gggauuugaacacuuCAA AUAGUAG GCCAGGGAUUUGA AGUU GUGU AAGAC AG GAUAUG ACCAA ACAGUAGACUCAG A**

**- C cca C A U ACAC A^ - A AAUAU C AG**

**zma-miRds68 UACGACUGAAGGGUAAAUUUGU**

**A A A -| C G UU UGUUU UUG**

**AUAUACAAAGAUAAAUUUACUCUUCAGUCGUA UU UGAUUUUG UC CUCUUU AAAA AAC GUAA U**

**UAUAUGUUUCuguuuaaaugggaagucagcau AA ACUAAAAC AG GAGGAA UUUU UUG CAUU G**

**- C C G^ U A -- UCUC- UUA**

**zma-miRds69 UUUGGAGUAGAGACCGAGCGA**

**AGUAGAG A CU U-------- UU**

**ACCG GCGACU GUAAA AGCGU \**

**uggc cgcuga cguuu UCGUA C**

**GGag--- - -- CAAUCCCUC CG**

**zma-miRds70 AGAGGACAACAAUCGCUUUUGA**

**GCGUU C G - UA - .-UUACUUAUAUAAGAUGUCUA -| UA**

**GAUA GU GAAA UGGUU UGUCCU UGGA GAUUAACA AUU A**

**CUGU Ca uuuu gcuaa acagga aUCU UUGAUUGU UGA C**

**GUCUC - g c ca g \ -------------------- C^ UA**

**zma-miRds71 AGGGCGAUGGCUUGCUAGACGA**

**GUCUAACAAGCCAUCGCCCUGC**

**C A a .-GAUCA| A UUAA U**

**AGCCUG CUUUCgucua caagccaucgcccugcCGCCCAGGGUGAUGCAUGUAUG UCAUUAUCUAGAAAGGAACAACCAU GG GUCU U**

**UCGGAC GAAagcagau guucgguagcgggACGGCGGGUCCCACUACGUACAUAC AGUAAUAGAUCUUUCCUUGUUGGUA CC UAGA A**

**- C c \ -----^ - UAG- A**

**zma-miRds72 UUGUUCAUAUGGGAGUGAUGUC**

**A C-------- g --- ucCU.-UCAUAUACCUUCUUCUUAAUCUCCU-| GAUA**

**GUUCAU GGuuguucauaug ga gugaug CA UUG CCAU \**

**CGAGUA UUAACAGGUAUAC UU UACUAC GU AGC GGUG C**

**A AAGUAACGA G UUC UACU\ -------------------------U^ UGCA**

**zma-miRds73a AGAUAGAGUGUUGUUUUCUGA**

**- .-AAAA uag - -|ug U**

**UGC UAUaga agug uuguuuu c aA U**

**ACG GUAUCU UCGU AACAAAA G UU A**

**C \ ---- UA- G U^GU A**

**zma-miRds73b AGAUAGAGUGUUGUUUUCUGA**

**CACUAUAAAAA U U UUAUG | G**

**UAGAGG CA AUAUUUUAUCUAU UUGU--UCUUAU G**

**gucuuu gu ugugagauagaUA AACG AGAAUA A**

**GGUUUAAUUAa u - UAA-- \ ^ C**

**zma-miRds73c AGAUAGAGUGUUGUUUUCUGA**

**UGCAAAAUA a-- uuc .-AUUAAUUUGAUGAAACCGAUGCUAU UU -| AAAACAAAUGCCAUUUC**

**Uagau gaguguuguu uga UCUACGAA GC UUG \**

**AUCUG CUUACAAUAG ACU AGGUGUUU CG AAC C**

**CCAUAUAGA GGG UC- \ ------------------------- UU U^ CAAACUCUUCUGCAAAU**

**zma-miRds74a UUGGAGCAAGGGAAUUGGAGG**

**U U -| A AG UUAGC**

**AG GCUAGUuu ggagcaagggaauuggaggGGAUUGAGGAGGCUAAAA UCC GGAUU \**

**UC CGGUCAAA CCUCGUUCCCUUAACCUCCCCUAACUCCUCCGAUUUU AGG CCUAA C**

**U C C^ - GA CUCCU**

**zma-miRds74b UUGGAGCAAGGGAAUUGGAGG**

**UUA A -| c - u UGCUA**

**GGC UGUuu ggag aag ggaau ggagg A**

**CCG ACAAA CCUC UUC CCUUG CCUCC A**

**UUC A C^ - U - CCCUA**

**zma-miRds75 UCGGUGUUUGGACUAGCGGUC**

**UUCACGAAUAUC| GAA C A UUU CCU- AUA**

**CUGCUAGUCUAAAU ACCG CA AAU UGU UGUA A**

**ggcgaucagguuug uggc GU UUA ACG AUAU A**

**CCAACCAAUCcu^ --- u G CU- UUCC AUG**

**zma-miRds76 UCGGAUUUUGUCGGCACGGCC**

**- .-U uu .-ccC| ACCACCC UUUU A**

**GUACUGGG Cucggauu gucggcacgg UGCUC UGUGC CUGC G**

**CGUGAUCC GAGUUUAA UAGCCGUGCC ACGGG ACAUG GACG G**

**A \ - U- \ ---^ GUAUCA- U--- U**

**zma-miRds77 UGCCUUGGUCGCACGGUUGCA**

**UU A A U .-AGAUG| AAAAA**

**CCUAUUGGUGCAACCGUGC ACUAAG CACAAGGAU GAGGUG ACAUUUUU U**

**GGAUAACCacguuggcacg ugguuc guGUUUCUA UUUUAC UGUAGGGG A**

**AU c c U \ -----^ GAUUU**

**zma-miRds78 CCGGAGGGGUUGAAGGUGCUA**

**GCUCCUCCAAUCUCCUCCGGUA**

**- CACAAA - a u -| A**

**AGC Accggagg gguug agg gcuaAAAUCCUC UUUUAUUCA U**

**UCG uggccucc cuaac ucc cgAUUUUAGGGG AAAAUAAGU U**

**C AUUUUa u c u A^ U**

**zma-miRds79 UUGGCAAGUAUGAGAGAGGAC**

**A UG AA - a-- a - ac UC- --- ---- .-UUCAACUACUA-| AA**

**UG UUG uug gca gu ug agagagg UCUC UGUCUC UCC GAGU AG UGAAGUG G**

**AC GAC AAC CGU CG AC UCUCUCC AGAG ACAGAG AGG CUCG UC ACUUCAC U**

**A GU CG A GCC G A -- UUU CUG GACA \ -----------G^ GA**

**zma-miRds80 UUCAAUCCUCCCGUGAUCUCG**

**A| CAAA A A GA**

**UUUGGUGA GAGAUCACG GGGGAUUGAAG GGAUUGAGGGGGAAAU A**

**AAACCACU cucuagugc cuccuaacuuC CCUAACUCCCUCUUUA C**

**-^ AGg- c C AU**

**zma-miRds81a UUGAAGUAAACAUGUAUGAAGUC**

**- ---| aa g U**

**GGC CUAAAUUuugaagu acauguau aagucCCCAUCAUA U**

**UCG GAUUUAAAACUUCA UGUACAUG UUCAGGGGUAGUAU G**

**U UAU^ -- - C**

**zma-miRds81b UUGAAGUAAACAUGUAUGAAGUC**

**- ---| aa g U**

**GGC CUAAAUUuugaagu acauguau aagucCCCAUCAUA U**

**UCG GAUUUAAAACUUCA UGUACAUG UUCAGGGGUAGUAU G**

**U UAU^ -- - C**

**zma-miRds82a UUUAGUAUCUAGAGGACAACA**

**CAACAUC uau c -| A**

**UCCuuuag cuagagga aA CAGAA C**

**AGGAGAUC GAUUUCCU UU GUUUU U**

**AGACGAC UAC A A^ U**

**zma-miRds82b UUUAGUAUCUAGAGGACAACA**

**A A C uau - a .-UCGCUUUCUGAC UA-| CCC**

**UGU GU CCuuuag cuagag gac acaG UGUCCUCUG GU \**

**ACG CA GGAAAUC GAUUUC CUG UGUC ACAGGAGAU CG U**

**G A - UAC U A \ ------------ CUA^ AUU**

**zma-miRds82c UUUAGUAUCUAGAGGACAACA**

**UU GC- ag u c C .-U| G A UG A**

**GACU ACuuu uauc agagga aaca CUGUG GCUGCA UGUGU G U \**

**CUGA UGAGA GUAG UCUCCU UUGU GAUAC CGACGU ACGCG U G G**

**CC GAA -- C A - \ -^ G A GU U**

**zma-miRds82d UUUAGUAUCUAGAGGACAACA**

**ACAGU UG CA .-C| G AGC**

**GCUU CUG GUUCUUUAGA UA AAGAC G**

**CGAA Gac caggagaucu gu uuCUG C**

**UUUUU -- aa \ -^ g GUA**

**zma-miRds83 UUAUCUUAGAAGGGUCGUCUG**

**- CCC---| a uu c AGG- UC C UC**

**GGUCUC Cuu uc agaagggu gucug GU GC GCGGU \**

**CCAGGG GAA AG UCUUCCCA CGGAU CA CG UGUCG A**

**A CGCCUC^ - CC U CACA UU - UU**

**zma-miRds84a UGGGCUUCUGAAAAUAGGUAUC**

**AGCCAAA AAUA A GCC C CCA - C-- -- .-AAAAA CACC A A CU - .-AC A-| U**

**CG ACUUGU UUUAGGA GCCCAG UCUU GAU CCU UUUCAA GGCUC CUACAA AAUU AGA AG CUG UAAGCAU UUAG UG G**

**GC uggaua aagucuu cggguC AGAA CUA GGA AAGGUU UCGAG GGUGUU UUGA UCU UC GAC GUUCGUA GAUC AC A**

**GCCACAA Ccua a --- - CCA U ACC UU \ ----- ---- - A UC U \ -- CA^ A**

**zma-miRds84b UGGGCUUCUGAAAAUAGGUAUC**

**AAAGC .-G G U-| A C**

**CGC GGAGGCU AGUUUU UGA AC G**

**GUG ucuucgg uCAGAA ACU UG U**

**----- \ - g CC^ A G**

**zma-miRds85 AAUCUAUUAGGAAAAUAAGG**

**C UAA AUGU CC CG A C G UAA A U A G | AU**

**GAAGAU GGCCC UUAUUUUCCU UAGAUUA UAAGUU GAUUA GAUGAAAA GUAG GGUAAAAUAUCACUUUG GA UAC AAUAAGCU AAAUAAGC--CAAC U**

**CUUUUG UCGgg aauaaaagga aucuaAU AUUCAA CUAAU CUACUUUU CAUC CUAUUUUAUAGUGAAAU CU AUG UUAUUUGA UUUAUUCG GUUG U**

**C --- ---- uu AU C A A CUC C U - A \ ^ AA**

**zma-miRds86 UCGGAGAAGGGCAAUCACUCGU**

**GCAGUAA a-| u uc CU**

**GGGucggag agggcaa cac guU \**

**CUUAGUUUU UUCCGUU GUG CAA U**

**AACAACA CG^ - U- AA**

**zma-miRds87a AUAUACUAUUAUGACCUCUAA**

**AA-- GGUU AUCC- UCUUACU GUU A C**

**GU GGGU UAGUGUGUGUGU GCA GUGG CAA A**

**CA ucca aucauauaCACA CGU UACC GUU U**

**AAAG aauc guauu UUUAU-- GU- G U**

**zma-miRds87b AUAUACUAUUAUGACCUCUAA**

**- C C .-AUAUAAAAAAUA .-ACACACGCA| C**

**CU UUUACGCauauacuauuaugaccucuaaACGA ACAGUA GUUACUGAUAGUAAC GG C**

**GA AAAUGUGUAUAUGAUAAUACUGGAGAUUUGCU UGUCAU CGAUGACUAUCGUUG CC A**

**C U U \ ------------ \ ---------^ G**

**zma-miRds88 CUUGCAUGCCGGCUGUAGGGGGC**

**ACCCCCUCU u- ccg--- -| ACG**

**Cc ugcaug gcuguagggggcACU CU G**

**GG GCGUAU CGACAUCCCCCGUGG GA C**

**UCUUUCCCC CU CCCCCG C^ CGC**

**zma-miRds89a UCGCGUCUUUCGCGGUCGGGC**

**A .-CC| GC**

**CACACGCGUCGCCCGACCGCGAAAGACGCGAGCUAGAGGUUUCUGC AGCAU U**

**GUGUGCGUAGcgggcuggcgcuuucugcgcuCGGUCUCCAGAGACG UCGUA A**

**- \ --^ GG**

**zma-miRds89b UCGCGUCUUUCGCGGUCGGGC**

**A A C C A .-CGACAC| UC**

**CACACGC UCGCCCGACCGCGAAAGACGCGAGC AGAGGU UCUGCU GGU CACCA \**

**GUGUGCG AGcgggcuggcgcuuucugcgcuCG UCUCCA AGACGG UCG GUGGU U**

**- C A A G \ ------^ UG**

**zma-miRds90a AGCUUUUGCAGCCGACUGGUCA**

**G-- UC--- A C U- U A**

**GC UGGCCAGUC GC GCAGG CUG CCGC--GCAG U**

**CG acuggucag cg cguuu gaC GGCG CGUU U**

**ACG UCUCC c a uc - \ U**

**zma-miRds90b AGCUUUUGCAGCCGACUGGUCA**

**G-- UC--- U - U- U .-A UUUUG**

**GC UGACCGGUC GGC GUAGG CUG CCGCGC GAU C**

**CG acuggucag ccg cguuu gaC GGCGCG CUG A**

**ACG UCUCC - a uc - \ - UGCCG**

**zma-miRds91 AAUCUUAGAUUGAAGUGGAUUGA**

**UUGGU AGC U GAAG A G- UC---- G UUUGA GAU .-CUG| CC AUUC**

**CCAC CA UCCAC CGAUC AAGA GCCC GCC UGAAG GGGGUC UGG CC AGGG \**

**GGUG gu aggug guuag uucu UGGG CGG ACUUC CCUCAG ACC GG UCCC G**

**AAUAU Ga- u aa-- a aa UUUGUU G CC--- AU- \ ---^ UA ACAU**

**zma-miRds92 UCAAUUGUAGGUAUAGAUGGAC**

**- C AU UA U AC---| G AA**

**AUUUAU CC CAAUUG GG AUAGAUGG AAU C \**

**uagaua gg guuaac CC UAUUUACC UUA G A**

**g u au uA C CAUAC^ G UA**

**zma-miRds93 CACCAUCUACGGCUGAGUGC**

**- C c c c U U --| U U AAGAACCA C**

**CCAU GAAUCca cau uacgg ugagugcAAUC CACC UCUCU CUUAGAC AU UUUUA UGAA A**

**GGUA CUUAGGU GUA AUGUC ACUCACGUUAG GUGG GGAGA GAAUCUG UG GAGAU ACUU U**

**G A A A A U U AG^ C C AAGAC--- U**

**zma-miRds94a CAAGCAAACCAUAUGAUCCAU**

**-| C c a c CG C U ACACUUAAACCCUUCCACCACCAGCUCAA**

**UGCAAGC UGcaagcaaa cau ugau cauUAGAU UGAUCCAACCGUAGGU ACA UUA U**

**ACGUUCG ACGUUCGUUU GUA ACUA GUAAUCUA ACUAGGUUGGUGUCUA UGU AGU A**

**C^ U A G U AU - U ACCAAACAAUCCCCAAAAAAAUAUUUCUA**

**zma-miRds94b CAAGCAAACCAUAUGAUCCAU**

**UACAAA c a c CG .-AGUCAC| U ACUUAAAUCC**

**CGUGcaagcaaac au ugau cauUAGAU UGAUUUAACCGCA AUU AGC \**

**GCACGUUCGUUUG UA ACUA GUAAUCUA GCUAGGUUGGUGU UAA UCG U**

**ACAUUC A A A AU \ ------^ C ACCACCACCU**

**zma-miRds94c CAAGCAAACCAUAUGAUCCAU**

**-| a CG C UUUAACACUUAAACCCUUCCACC ACUCAAUAAUCUUUAUA**

**UGUAAGCGUGcaagcaaaccau ugauccauUAGAU UGAUCCAACCGUAGGU ACA ACCA A**

**ACGUUCGCACGUUCGUUUGGUA GCUAGGUAAUCUA GCUAGGUUGGUGUCUA UGU UGGU A**

**C^ G AU - ----------------------- ACCAAACAAUCCCCAAA**

**zma-miRds95a UCAAUACACGUGGAUUGAGGUG**

**A| A A A A A G**

**CUCUC UAUUCA CUCGAUCCAUGUGUAUUGAG UGGAUUG AGUGUAA UUA \**

**GAGGG AUAAgu gaguuaggugcacauaacuC ACCUAAC UCGUAUU AAU U**

**-^ C g C C G U**

**zma-miRds95b UCAAUACACGUGGAUUGAGGUG**

**CCUCAAUCCAUGUGUAUUGAGG**

**A| G C A**

**CUCUCGUAUUCAccucaauccauguguauugaggUGGAUUG GGUGUAA UUA \**

**GAGAGCAUAAguggaguuaggugcacauaacuCCACCUAAU CCACAUU AAU A**

**-^ A U C**

**zma-miRds96 CGGUAGUGGAAUGUGGUGGCGC**

**UC - A g aa g - .-UAA ---- .-AUGACAAG| GUAGA**

**GCG GG GUc guagugg uguggu gc gc GCA GUGCGCCUG GAGC \**

**CGC CC CAG CGUCGCC ACGCCG CG CG CGU CACGCGGGC CUUG A**

**GA U A A -- A U \ --- CCUC \ --------^ AGGUU**

**zma-miRds97 UAAACUGUUUUAGUCUUUAGUC**

**AAACAA G ac uu ---| CA**

**GAA uaa ug uuagucu uuagucACU \**

**CUU AUU AC AAUUAGG AAUCAGUGG A**

**UUCCAA A AU CU AAA^ UA**

**zma-miRds98 UCUGAAAUACAUGUCGCCGGUU**

**CAA GA AA A GU .-AUUUUUUUAUUGCC UU-| UAAAU U**

**UUGAA UGAUCGG GAUA GUGUUU GAU ACUUUUUG CC CAA C**

**GAUUU Auuggcc cugu cauaaa cuG UGAAAGAC GG GUU A**

**UUA G- g- a gu \ -------------- CUU^ UGUC- U**

**zma-miRds99 UCUGAAUAAAUAGAAUACGUAA**

**- - U- --- au ua- a .-AUAC| ACU**

**CUG UGCGAU uc ugaauaa agaa cgu aUUUG UCAAC C**

**GAU AUGCUA AG ACUUAUU UCUU GCA UAAAC AGUUG U**

**U G UC GUU CC CCA A \ ----^ AUG**

**zma-miRds100 AGCCAGAUUCUCGGAAAAGCU**

**G GUU CU .-C GCU----| UCAGACCG**

**UGUUU UUAGCUUUUUCGAG UCUGGC ACCAGGA GCU G**

**ACAAA GGucgaaaaggcuc agaccg UGGUCCU CGA U**

**- GUU uu \ - AUUUUUU^ UUCACAAA**

**zma-miRds101a UCCCCUUCGGGAUUGGAGAGG**

**-| C cg a AU GA**

**GAA CUCAAAuccccuu ggauuggag ggAUUGGG GGAAAU A**

**CUU GAGUUUAGGGGAA CCUAACUUC CCUAACCU CCUUUA C**

**C^ U AU C CU AU**

**zma-miRds101b UCCCCUUCGGGAUUGGAGAGG**

**-| AC c c g- UAA AA**

**GA CUCAAAucc cuu gg auuggagaggAUUGAGG AAAU A**

**CU GAGUUUAGG GAA UC UAACUUCUCCUAACUUC UUUA C**

**C^ AU A - GA UCC AU**

**zma-miRds101c UCCCCUUCGGGAUUGGAGAGG**

**- C UA-| cc a - U AUGA**

**GAA CUCA uc cuucgggauuggag ggAUU GAGG GAAA \**

**CUU GAGU AG GAAGCCCUAACUUC CCUAA CUCU CUUU A**

**A U UUA^ A- C U C AAUC**

**zma-miRds102 CCCGUUAGACAUAGAGAUGGCAA**

**A U AA C U- U ---| CA**

**GGG UGCUUAU CCGUC UUAUGUUU CGGG GCC GACU \**

**CCU AUGAGUa gguag gauacaga gccc CGG UUGA U**

**G - ac a uu - UCA^ UA**

**zma-miRds103 UGAAGUGGAUUAGAGGGGCUA**

**-| A a u a c G CC A**

**GC AGGGGAAug ag ggauu gagggg uaAAAUC CCUU UAUUCA U**

**CG UUCCCUUAC UC CUUAA CUCCCC AUUUUAG GGAA AUAAGU U**

**U^ G C C C A G CA U**

**zma-miRds104 UAUUUAUGAUAUGUUACUCUA**

**U| C A A A CC**

**GGAA GGUAG AGGGUAA AUAUCAUAAAUAA CUGAAAUAAGCUA U**

**UUUU UCAUC ucucauu uauaguauuuauU GACUUUAUUCGAU A**

**-^ C a g A AA**

**zma-miRds105 ACCUAGUUUUUAAGAAUUUAG**

**- ACGa - a CCAU .-UUUAUAA| A UGUU AC**

**UUUGGAA ccu aguuuuuaagaauuu gUUU AAACUGG AAAAUUGGUU AUGG UGGA C**

**AAACCUU GGA UCAAAAAUUCUUAGA CAAA UUUGACU UUUUGAUCAA UACC AUCU A**

**C CG-- G C UACU \ -------^ A UUU- CC**

**zma-miRds106 UAGGAUUAGCAUAAGAAUUGGU**

**CAGUGUCUUGUCUGAAUCGAG**

**C ----- a a c - .-AA .-CCC| CU C**

**GGAAU UUCUu gg uuag auaaga auuggu AAAAAGC GAU UC A**

**CCUUA AAgag cu aguc uguucu ugacUA UUUUUCG CUA AG U**

**A ACGAU - a - g \ -- \ ---^ AG U**

**zma-miRds107 AGAGAGGAUUGAAGGGGUUAC**

**-| a U CAA**

**CAAGGAUACCagagaggauug agggguuacAAUCUCUUU CUAUU A**

**GUUCUUAUGGUCUCUCCUAAC UCCCCGAUGUUAGGGGAA GAUAA A**

**C^ C C AUC**

**zma-miRds108 GCCUCCGUAGCAUAGUGGUAGUG**

**---- GGU GA .-U .-GAC GA**

**ACCA UGUUG GAGGU GACG GACG U**

**uggu acgau cuccg CUGC CUGC C**

**guga gau gc \ - \ --- AC**

**zma-miRds109 UGAACCGUUGCCUGCUAUCCAG**

**UG .-A .-c ug -| a**

**GGAACU Cugaa cgu ccu gcu u**

**CCUUGA GACUU GCA GGA Cga c**

**GG \ - \ - UA U^ c**

**AGAU - GG C- .-GAGAAG UG A GG**

**CCG UUC CCAC GUCCAU A AUGGAA UGU A**

**GGU AAG GGUG UAGGUA U UACUUU ACG A**

**U--- G A- UA \ ------ GU A AA**

**zma-miRds110 GAGAGAGCUGUAAGAGCGGUA**

**A A g - g a- --| aAAA A**

**CAAGAG GA aga ga cugua gagc ggu GA A**

**GUUUUC CU UUU UU GACAU CUUG CCG CU G**

**A - A A G CA GC^ GUGG C**

**zma-miRds111 AAGUAGGGGUUGAUAUUUUGAU**

**UACCU U g --- auu u .-AAACA| U**

**UUAU aaguag ggu ugau uuga GUGG AG G**

**AAUA UUCAUC CCA ACUA AACU CACU UC U**

**AUUAC C A AAU AC- - \ -----^ G**

**zma-miRds112a GGGUGGUUACAGAUCGUAGGAGA**

**CGAAGGGGAAAAA - G- C AAC--| U**

**CC ACGG UG UGACCACC AAUA C**

**gg ugcu ac auuggugg UUAU A**

**AGUAACAUUUaga a ag - GCUUU^ U**

**zma-miRds112b GGGUGGUUACAGAUCGUAGGAGA**

**- - -| a a C C- GU AA**

**GUAGA CC ggaug auagagaa cug ACC GCAGA UAU A**

**CAUCU GG CCUAC UAUCUCUU GAU UGG UGUUU GUG G**

**U C C^ G C - UU -- AU**

**zma-miRds113 UCCUGCAGAAACGAACGGGCC**

**AAGGCCUA G - C .-GAAGACC| U**

**AGGGCCCGUUCG UUC GCA GGAAUG GGAA \**

**UCccgggcaagc aag cgu ccuUAC CCUU A**

**UUUAAUAA a a - \ -------^ G**

**zma-miRds114a ACGCACGCAGGGUCACCGGCC**

**AGA A- c g g- - .-GGU .-A| A**

**UAG GGCacg ac cagg ucaccgg cc GUGCGGAGG CCGG \**

**AUC UCGUGU UG GUUC GGUGGUC GG CAUGCUUCC GGCC G**

**GUA AG U G AA U \ --- \ -^ U**

**zma-miRds114b ACGCACGCAGGGUCACCGGCC**

**AGAGAGAG ac- - ac c ---- GGA GGA .-GAGGG| G A**

**GC gcacgcagg guc cgg cGGUGU GC GGAUC AGC CGC AG C**

**CG CGUGUGUCC CAG GCC GCCGCG CG CCUAG UCG GCG UC G**

**UAUCAGCG AUC G CC - AGUC AGC AG- \ -----^ G A**

**zma-miRds115 AGCGACUACAGAGGACCGUCAA**

**UA .-CUAA| a ac c aAAA**

**GAUG agcg cu agaggac guca \**

**CUAC UCGU GA UCUCCUG UAGU G**

**UA \ ----^ A -- - CAGC**

**AAA-------- ACUAC --- AA**

**GAG AGAGGACA GUC A**

**CUC UCUCCUGU CAG A**

**CUUAUUCAGAA GUAGA UGU CG**

**zma-miRds116 GUUUGAUUUAAGGUUAAAGUA**

**UUUAUGA u g ua u AUA U C--| UCGA**

**UGUguuugauu aa gu aag aGG AGA GGGGGC--GACAUUGU CCU \**

**ACACAAACUAA UU CA UUC UCC UCU UCCCUG CUGUAACA GGA U**

**UAUUCCC C A UA C CUC - \ AUA^ UUUU**

**zma-miRds117 AGAGGAUCUAUGGUGGAGGAA**

**-| A a u A A**

**AG GAUCACGag ggaucuaugg ggaggaaUCCCCUU CUAUUUA U**

**UC CUAGUGCUC CCUAGGUACC CCUCCUUAGGGGAA GAUAAGU U**

**G^ - C C C U**

**zma-miRds118a UCUAAAGGUCCAGGAGGCUCG**

**AGCAUCCUAGACCUUUAGAUC**

**A C a a cG C .-CACA| C**

**AG GUGCAAGUGagc uccu gaccuuuagau UGAUCCAA CGUAGGU UUUAA \**

**UC CACGUUCgcucg agga cuggaaaucuA ACUAGGUU GUAUCUA AAAUU A**

**A - g c AU A \ ----^ C**

**zma-miRds118b UCUAAAGGUCCAGGAGGCUCG**

**GCCUCCUAGACCUUUAGAUCG**

**AGGU ----- a cg C .-CCACA| C**

**GUAAGC GUGCAAGUGAgccuccu gaccuuuagau UGAUCCAA CGUAGG UUUAA \**

**CGUUCG CACGUUCgcucggagga cuggaaaucuA ACUAGGUU GUAUCU AAAUU A**

**UU-- GAAUC c AU A \ -----^ C**

**zma-miRds119 AAGUGAGGAUGGGAUUAUCUGC**

**AUAAAAGA .-CUA GC-| GC**

**GAGCAGAUAAUCCUAUCCUCACUUGAGGAUG UGUGGA AU A**

**CUcgucuauuaggguaggagugaaCUCCUAC ACACCU UA C**

**AUCUUCCA \ --- AAC^ AA**

**zma-miRds120 AGGAGGUGUUUGUUUGGAAUU**

**A| UUag a AC A A C**

**UGCUAAC gagguguuuguuugg auuAUAAUCU UCGGAUUAUA AAUC AACAAAUUUUGAACUAA \**

**AUGAUUG CUCCACAAACAAACC UAAUAUUAGA GGUCUAAUAU UUAG UUGUUUAAAACUUGAUU A**

**A^ ---- C CA A G C**

**zma-miRds121 UGGCGGCGGCAACUCCCUUCUC**

**- U U u- - a-- .-uCU CCG-| UC**

**GCC CA CCG ggcgg cggca cucccuuc CGCC GUC C**

**CGG GU GGC CCGCU GCUGU GAGGGAGG GCGG CAG C**

**G C C CC G GAA \ --- ACGA^ CA**

**zma-miRds122 AUACUUAAACUUAGGUAUGU**

**UGAA| a c g A AU**

**GCCGAC uacuuaaa uua guauguC GCCU G**

**UGGCUG AUGAAUUU AAU CAUGCAG CGGA C**

**AUCC^ C C A C CU**

**zma-miRds123 UCUAAACAAAAGUAGAAGUUC**

**A U ---- -- GCA GAAAA**

**UUAG GUUU CUUUUAUUUUU UUGGAA AGG \**

**AAUC CAAA gaagaugaaaa aaucuU UCC A**

**U - Acuu ca AAA GUAAU**

**zma-miRds124a UACAUUUUAAAUCUGAGACAUC**

**- c g ----- ---| C C C G GAUU- CA C AAAG**

**UGCUCAAGUAua auuuuaaaucu aga caucCA UCU ACGUC AGCGA AACA UUAAUUU AAAAAC CU UCACC G**

**ACGAGUUUAUGU UAAAAUUUAGA UCU GUAGGU AGA UGUAG UUGUU UUGU GAUUAAA UUUUUG GA AGUGG A**

**C A - UGCAA UGU^ U A U - AUGUU AA A AAGG**

**zma-miRds124**b **UACAUUUUAAAUCUGAGACAUC**

**UGC - a C C ---| C ACC AAA ACCUCCUUCCCA G**

**UCACGUGuacauuuuaaaucu gag cauccaucU AUAU UAAC AG AGC AAUUUUAUAAA AACUCUUCU UGGC A**

**AGUGUACGUGUAAAAUUUAGA CUC GUAGGUAGA UGUA GUUG UC UCG UUAAAAUGUUU UUGGGGAGA ACCG G**

**AUA U - A A CCA^ C A-- G-- ------------ A**

**zma-miRds125a UCACGUCGUCUCUACAUCCGU**

**AC----------- U GUA A AU .-UUAUGAAAAACUA AC**

**GAU UAGAGGC GACG GGG UCGCUG GUGG \**

**cua aucucug cugc cuC AGUGAC CACC A**

**CUCUUCCCAUugc c --- a AC \ ------------- UG**

**zma-miRds125b UCACGUCGUCUCUACAUCCGU**

**C UA a --- c c .-AC -| A U G- UC**

**CAGUGA Cuc cgu cgucucua au cguU CCUG UGGC G CCGC GGCGAGU U**

**GUCGCU GGG GCG GCGGAGGU UA GCAG GGAC GCCG C GGCG UCGCUCG G**

**U UA A GAU U - \ -- G^ - - GG GC**

**zma-miRds126a AGCGGACUCUUGUCGGUGGGU**

**U C C--- .-C CGUCCCACCC - U U**

**CGGACGCGCGACCCACCGACAAGAGUCCGCUCCGCCGCCACGCGCUGCGCGC CUGG GCUCGU GCGG ACGC GA GCCG G**

**GCCUGCGCGCuggguggcuguucucaggcgaGGCGGCGGUGCGCGACGCGCG GACC CGAGCA CGCC UGCG CU CGGC C**

**- U AGGA \ - ---------- A C U**

**zma-miRds126b AGCGGACUCUUGUCGGUGGGU**

**U .-AC GGAGGA .-GUGA GU .-AG| AC**

**CGGACGCGCGACCCACCGACAAGAGUCCGCUCCGCCGCCACGCGCUGCGCGC UGGUCCUGCUCGU GCGCGUCC GGG CGG CGCGGCGG G**

**GCCUGCGCGCuggguggcuguucucaggcgaGGCGGCGGUGCGCGACGCGCG ACCGGGGCGAGCA CGCGUAGG CCC GUC GCGCCGCC C**

**- \ -- GGG--- \ ---- UG \ --^ GC**

**zma-miRds127 UUGAGAGUAAGUGGAAUGGAG**

**- U G a u - AG GU A-| UA**

**GAGG CUU Uuug gaguaag gg aauggagGGAUU GG CU GGAUCCCUUAUUAUUU A**

**UUCC GAA AAAC CUCGUUC CC UUACCUCCCUAG UC GG UUUAGGGAGUGAUAAG A**

**A C G C - A GA UG GA^ UU**

**zma-miRds128 UGAAACUGUCACAGCAUGAUCUA**

**-| CA A U CA GCG G A**

**AAACCAA CAAGAAUGGAG UG GAGGAUCUUUUUAAAAAUAUC GU AGGU GA G**

**UUUGGUU GUUCUUAccuc ac cuccuggaaAAAUUUUUAUAG UA UUUA UU G**

**G^ AG c c UA AGG G G**

**zma-miRds129 UCAUGUAGUUCAGAUUAGAAG**

**UGCAACCA C .-CAAAUA GCGG - .-GAGGGG G**

**UGUAAACUUCUAAUCUGAAC ACAUGAUGUUGAUC GACGU AGGAGUG AGA AUUU A**

**ACGUUUgaagauuagacuug uguacuACAACUAG CUGCG UUCUCGC UCU UAAA A**

**AGCC---- a \ ------ ---- A \ ------ A**

**zma-miRds130 UUAACGGGUAGAGAAUCUUCU**

**---- AAG CUA C c GAAA C AU GU-- G .-AAAGA| AU UUAUC C**

**GUC UGCU UG AUUGuuaacggguagagaau uucuCA AUUC CCAU UUGAU UC GAAGCA UGUGG GAU AGGC A**

**CGG ACGG AC UAACAAUUGCCUAUCUCUUA AAGAGU UAAG GGUA AACUG AG CUUUGU ACACC CUG UUUG A**

**GCUU AG- UUA - C AGUC A -- ACGU G \ -----^ AU UC--- A**

**zma-miRds131 AUGCGGAGAGGCUCUCGAGAGA**

**GUUUCUG c -- c ------ - - A C-| AACG A**

**UAGaug ggaga ggcu ucgagaga UUGACAG AAGAG AGUGAGCAC CGGCG GACG ACAU \**

**AUCUGC CCUCU UCGG AGCUCUCU GACUGUC UUCUC UCACUCGUG GCCGC CUGC UGUA G**

**GCCUAG- - AU U UUCUCC U U C UC^ CGUA C**

**zma-miRds132 GGAUGUCGAUAUUGGAGGGC**

**U U A c gg CU-- UU .-AU AA GC-| UG**

**GC CC UUUggaugu gauauugga gcAUGGAA GAAUUGG UCA UACA UCA CA A**

**UG GG AAACCUACA CUAUAACCU CGUGCCUU CUUAACC AGU AUGU AGU GU U**

**G - C U -- AAUC UU \ -- AG AAA^ UA**

**zma-miRds133 UUCACCUCCCGGACAGGUAUG**

**C ----| c c uau UU**

**UUCCUCUGCu ucac uc cggacagg gAU \**

**GAGGAGGCGA AGUG AG GUUUGUUU UUG U**

**A GGGA^ A A UGU AU**

**zma-miRds134 UGCAACUGUGCUCAUAUACAAC**

**.-UCCUGUCUU a - u ua- .-c| GGUUUAAAUUC UU A**

**Uugc ac ugugc caua caa UAGUUAUU ACUG GC A**

**AACG UG ACGCG GUAU GUU AUUAAUGA UGAC UG C**

**\ --------- G U - UCG \ -^ ----------- CC U**

**zma-miRds135 GAUGUUGAUCUAAGGGACACA**

**- C - aca---| C AUAAUUUUACACUUAACCACCCACUG**

**UGGGC ACAUgauguugaucu aagggac GGGG GUGG \**

**ACUUG UGUACUACAACUAGG UUCCCUG CCCC CACC C**

**G A U CACGCC^ U CUCCCCCUAAACUUUUUCACACUAAU**

**zma-miRds136 CAAGGCUGAGGAAGGAUUUCGU**

**G U - U- A GA A .-AUA AACUACUUUAG- .-UGA| GA A**

**UUGUG GCA UAU AGUC CU UU UAGCUUUGA GGACA UAACUCU CUAUUGCUU AAC A**

**AACAC CGU Aug uuag ga ga gucggaacU CCUGU AUUGGGA GGUAACGAA UUG G**

**- - C cu - ag - \ --- GAACGACGUAAG \ ---^ G- C**

**zma-miRds137 UUUGUCACAGAAGUUGGGAUG**

**-| GGUG UG UU A**

**GUA UACuuugucacagaaguugggaugCAAACU UU GU G**

**UAU AUGAAACAGUGUUUUCAACCUUACGUUUGA AG UA A**

**G^ AUAA GU UU G**

**zma-miRds138 UAAGGAUUAAAGGAAAACCGGA**

**- CCAAACA - ga c- a - --------| ACU A UCUU U**

**GCU uaagga uuaaag aaa cgg GAG CUAGUA GCA AGCA CU GACA C**

**CGG GUUCCU GGUUUC UUU GUC CUC GAUUAU CGU UCGU GA CUGU U**

**A UUACCCA C AG CA - G UUCUCUUA^ CU- C UUU- U**

**zma-miRds139a CCAGGUUUCUUGUAGAAGCGGU**

**CAA AA c uuu -| guAGC - CC**

**ACUG Cc agg cuuguaga agcg UCC GCC C**

**UGAU GG UCC GAACAUCU UUGC AGG CGG U**

**CCC GC U CUU A^ ----- A UC**

**zma-miRds139b CCAGGUUUCUUGUAGAAGCGGU**

**CAA AA c uuu -| guAGC - CC**

**ACUG Cc agg cuuguaga agcg UCC GCC C**

**UGAU GG UCC GAACAUCU UUGC AGG CGG U**

**CCC GC U CUU A^ ----- A UC**

**zma-miRds140 UGUAGAAUUGAUAUAUAGGUGU**

**.-ACAA| UC u - a agg uA**

**CAA Auguagaa ug au uau ug C**

**GUU UACAUCUU AC UA AUG AC A**

**\ ----^ U- U A A GUA UA**

**zma-miRds141 AAGAGAAGGUUGACACAUGUU**

**AUU A -| AA GAGA**

**UAU UGAGACAUGU UCGACCU UCU G**

**GUG GCUuuguaca aguugga aga C**

**AAU A c^ ag AGCA**

**zma-miRds142 UAGAAAAUAUUUAGGACAUCUG**

**.-UA CUGAA U G .-ACCACUAA A**

**UUUGUUUAUUUU UGUG GGGUGU UCAA GUA \**

**Agauaaauaaaa acgu uccACG AGUU CAU U**

**\ -- ----- u A \ -------- C**

**zma-miRds143 AUUGUAAAGAAAUAUUUAGAU**

**- UA Aa a a a A A C C .-A U UA- .-A A---| AACA**

**GCU AAUA uugu aagaa uauuu gauCGU AUC AUUA UAC CCUAG GGUCC AGAGG GCCGA GCUU AGGUC \**

**UGG UUAU AACA UUCUU GUAAA CUAGUA UAG UAAU GUG GGAUC CCAGG UUUCU UGGUU CGAA UCUAG U**

**C GA AA C A C C A A A \ - - UAA \ - AGCA^ AAAG**

**zma-miRds144 ACGGAUGAGGUGUAAAUCAAG**

**A AU g | G**

**GUA GGGAacggau agguguaaaucaagCACUUGGUGCAAACC--GUGCA--AUCA U**

**CAU CCCUUGCCUA UUCACGUUUGGUUUGUGAAUCACGUUUGG CACGU UAGU C**

**A -- G \ \ ^ A**

**zma-miRds145a GCAAGCAAGAUUGGUCGGAU**

**- c u uc .-CACUUUUCC| UA**

**UUUCCAUU--GUgcaag aaga ugg ggauUAGCC GUGGGU \**

**AGAGGUAG CAUGUUU UUUU AUU UUUAAUUGG CACUUA U**

**G \ U U GA \ ---------^ UA**

**zma-miRds145b GCAAGCAAGAUUGGUCGGAU**

**UUGGACAUCUUGUUUGGGC**

**GUA --- G | UU**

**UGGGCU U GAU--GUUC A**

**ACCCGA g cug CAGG U**

**UUC Uua g \ ^ UU**

**a-- G UACA .-UUU ----- --- G GG**

**ucuuguuug gc GGA AUGU UCUUAAUU UCCUG CGU \**

**agaacgaac UG CCU UACG AGAGUUAA AGGAC GUA A**

**guu g UUA- \ --- AUUUC UUA G AU**

**zma-miRds146 CAAUACACAUGGGUUGAGGAG**

**- a -| A**

**GUAUUGACCCcaauacacauggguugagg gGAUUG GGUGUAAAUUA \**

**CAUAACUGGGGUUAUGUGUACUCAACUCC CCUAAC CUACAUUUAAU A**

**U A U^ C**

**zma-miRds147 GUCUUCAUGCUGUAGGAGAGGA**

**GCA UG U u- u a - .-a| CUG**

**U CGG guc uca gcugu ggag agg UACCC A**

**A GCC CAG AGU CGGCA CCUU UCC AUGGG G**

**ACC GU - UC - C A \ -^ UUU**

**zma-miRds148 GGUCCCCGGCAACGGCGCCA**

**GGGCC AGAA A GUCA AC G- - A GAGAU C- --- AA**

**ACGCUGU GGC GCCGU GCUG GGGC GCG GUGCCG UC CCUG GG GCC \**

**UGCGGCG ccg cggca cggc ccug CGC UACGGC AG GGAC CC CGG G**

**CU--- Ga-- - a--- c- gA A - ACCAC CA UGA CG**

**zma-miRds149 UUGGAUGUCGAUAUUGAGGGCA**

**U c a .-AA| GUUC**

**AUGCUCCGUuuggaugu gauauug gggcaUGGAAUUG UUGG \**

**UAUGGGGCAAACCUACA CUAUAAC CUCGUACCUUAAC AACC A**

**U U - \ --^ AUAA**

**zma-miRds150a UACAGAAUGGUAAAGAGGGCUC**

**UGGGU UA -| C A A C A A**

**UGGA GGCCC CU UACCAU UCU UA AAAU GA U**

**AUCU ucggg ga auggua aga au UUUA CU A**

**CUAGC Uc a^ a - c A C G**

**zma-miRds150b UACAGAAUGGUAAAGAGGGCUC**

**UGGGU UA -| C A A C A A**

**UGGA GGCCC CU UACCAU UCU UA AAAU GA U**

**AUCU ucggg ga auggua aga au UUUA CU A**

**CUAGC Uc a^ a - c A C G**

**zma-miRds151 UCAGAAAAUAUGAACUUGAGA**

**CUUAAU| c ug CAUA G AUU**

**UACCu agaaaauaugaacu agaUUCAACUUUUA GAGAAAUAAAAGA ACAAAUCUU A**

**GUGGA UCUUUUAUACUUGA UUUAAGUUGAAAAU CUCUUUGUUUUUU UGUUUAGAG G**

**GUGUAC^ A GU ACGC A AGA**

**zma-miRds152 AGAUACUGAACGGAUGAGUUG**

**U U C .-AA U--| GU**

**AAAAUACAUCUAACUCAUCCGUUC GUAUC AAC UGU AAUGU U**

**UUUUAUGUAGguugaguaggcaag cauag UUG ACG UUACA A**

**- u a \ -- UUU^ AU**

**zma-miRds153 UCUGGACCAGUUGAACGGGCC**

**CU A - - aacg .-cGA .-CCA -| CA**

**AGCCUC G ucugg accaguug ggc GCUGG GACACGG GGUCG A**

**UUGGAG C AGACC UGGUUAAC CCG CGAUC UUGUGUC CCGGC A**

**GC C G A AA-- \ --- \ --- U^ AC**

**zma-miRds154 UGAAUAGAGAAACUGCACCCGC**

**- - -| a a c c- GU AA**

**GUAGA CC GGAug auagagaa cug acc gcAGA UAU A**

**CAUCU GG CCUAC UAUCUCUU GAU UGG UGUUU GUG G**

**U C C^ G C - UU -- AU**

**zma-miRds155 UUGAUGUAGUUAGUACUCAGG**

**UGC a- guac--| UAUUCGACGCUC**

**AAAUUGUuug uguaguua ucaggU \**

**UUUAGCAAGC AUAUCAGU AGUCCA A**

**UAA GA GAGCAC^ UUAAUCAAUAGA**

**zma-miRds156 GAGCGUGGCGCUAGAGUCGGUG**

**UU G c c a u -- .-A| G UU AACU GG**

**--CGGAG GG gag gugg gcu gag c ggugG GACUU GGG GGGGUAG GCUC A**

**GCUUC CC CUC UACC CGG UUC G CCACC UUGAG CUC CCUCAUC UGAG G**

**\ -- G C - A U UU \ -^ A -- CGU- AA**

**zma-miRds157 AUAUUUUAAAUUUGGGACAUU**

**-| a C UC CGA AA--- AA - CG UCCC**

**GCUCACGUGCauauuuuaaauuugggac uuUAUCUUG AU AAC AGCAGC UUUUAU AGAACCC CUU ACC U**

**CGAGUGCACGUGUAAAAUUUAAACCCUG AAGUAGAAC UA UUG UCGUCG AAAAUA UUUUGGG GAG UGG C**

**A^ C U GA --- CGGUG C- A AG UACU**

**zma-miRds158 UAAGACACAACUUAAGACACA**

**UAA GUCA A ACA .-CCA| CAU ACCA**

**UACAGUG UGUCU AA UGUGUCUUA UAUU UGU A**

**AUGUCAC acaga uu acacagaau AUAA ACG U**

**UAC ac-- a ca- \ ---^ CUU AGAC**

**zma-miRds159 GACGCUACGGACUUGAUUGUAU**

**UUUAUGAA A AG GG CAAAUA GAUGAG .-GACUA| AA**

**UAAUAUAAU AAG CG GUGUCU GA UUGCUA CGUUGAAU \**

**GUuauguua uuc gc cgcagA CU AGCGGU GUAACUUA U**

**GGUUCCGA g ag au UGG--- AA---- \ -----^ AG**

**zma-miRds160 UGAAAGGCGGAUGGCUCUCUA**

**UGUACC CC GG - CA U | AU**

**GAUC GAGAGCCAU GCCUUUCACUUGUGUGUCUA GACC CUUGC AAUCAC--CAC C**

**CUAG cucucggua cggaaaguGAAUACACAGGU CUGG GAACG UUAGUG GUG G**

**AUAUUA au gg C AG U \ ^ CU**

**zma-miRds161 AAGAAUGGAGAUGUGAGGAUC**

**-| CA a u CA GCG G A**

**AAACCAA Caagaauggag ug gaggaucUUUUUAAAAAUAUC GU AGGU GA G**

**UUUGGUU GUUCUUACCUC AC CUCCUGGAAAAAUUUUUAUAG UA UUUA UU G**

**G^ AG C C UA AGG G G**

**zma-miRds162 UUUGACACGAAGCACGAUGAGUU**

**UCCGAAUCC acac a a - | GU**

**Guuug ga gc cgauga guu--UGGGCC G**

**CAAAC CU CG GCUACU CAG ACCCGG C**

**ACGCACUAA CC-- C - G \ ^ GU**

**zma-miRds163 UAUGUGGCAGUAGGUCGUCGUC**

**-| G u u AU C C GA C UCCUUCAUUGACACAC**

**AGCUG CGCCuauguggcag aggucg cgucGUGGU UUGG GCUGACCUC UUUCCUA AACGG UGCUCGGCCUCA \**

**UCGAC GCGGAUGCACCGUC UCCAGC GCAGCACCA AACC CGGCUGGAG AAAGGAU UUGCC GCGAGCCGGAGU A**

**C^ G C C GU A A UC A UACUCACCACACACAC**

**zma-miRds164 AGAUCAGAUGAUUUGCUUGCA**

**CAAGCAAACCAUAUGAUCCAU**

**- c a c AC C .-ACAGAUACAA G UU AGG-| CU**

**UGCAAGCGUGcaagcaaa cau ugauc auUAGAUU GAUC AACC CCAU AU GUU GGUUUU U**

**ACGUUCGCacguucguuu gua acuag UAAUCUAG CUAG UUGG GGUG UG CGA UUAGAA A**

**C a g a CA A \ ---------- G GU GUUA^ AU**

**zma-miRds165 UCCUGCAGAAACGAACAGGCC**

**G A U C GUUGUUAU .-GAGUACU C**

**GCC ACU AGGGCC GUUCG GGAAUG GGAA \**

**UGG UGA UCccgg caagc ccuUGC CCUU C**

**- G U a aaagacgu \ ------- G**

**zma-miRds166a CGGGUGCAUGGAAGCUCGGCAC**

**G A-- A AGCAGG GA .-G| CAACAA CA**

**UGUC CGGUG UGAGC GUGCAC CGA GCCGGA UGU \**

**ACAG GCcac gcucg uacgug gcU CGGCCU ACG U**

**G ACA g aagg-- g- \ -^ CGGGG- GG**

**zma-miRds166b CGGGUGCAUGGAAGCUCGGCAC**

**G A-- A AGCAGG GA .-GG| G A**

**UGUC CGGUG UGAGC GUGCAC CGA CG GACA C**

**ACAG GCcac gcucg uacgug gcU GC CUGU A**

**G ACA g aagg-- g- \ --^ A A**

**zma-miRds167 AACAAGAAAACGGAUAGACGCA**

**AA A ----- - - aacg a .-aGC .-AC| U**

**CCUG CC Caa c aagaa gauag cgc GCA GGCU G**

**GGAC GG GUU G UUCUU CUAUC GCG CGU CCGA A**

**GC C UCCAA A U CAA- - \ --- \ --^ U**

**zma-miRds168 AAGUUAGAUCUAAGGGCCAAA**

**A AC U G -| AG**

**UACUCCUUUGUUUGGCCCUUAGAUCUGAC GGUA GCG GUCA GC \**

**AUGGGGAAACaaaccgggaaucuagauug CCGU CGU CGGU CG C**

**- aa U A A^ AC**

**zma-miRds169 AACUAUGGACCUAAAGAAAAGAC**

**U G --| A A A UC UG**

**AAGUCAUUUUG CUUUUCU AGGUUCAUAGUUUUU CU UGU UCUAG AU \**

**UUCAGUAAAAc gaaaaga uccagguaucaaAAA GA ACG AGAUC UA U**

**- a aa^ C C A UA UG**

**zma-miRds170 AUCAACUUAAACUUUUGGGUUG**

**U U- UA- CU .-A --| ACGCAAC**

**CC CC UCAGCUUAAG UUUGGGUUGA UUGGU UGGUGC \**

**GG GG Aguuggguuu aaauucaacu gaccg acuaUG U**

**- UU UUA uc \ - aa^ GUAUAAU**

**zma-miRds171 CCUUGCUUCGGCGGUGGACGCA**

**CC C - -- u c .-a ACC--- A-| AUAA U**

**GGGUUC Cccuug cuuc ggcgg gga gc CCG CUG GUUGG GUGGU A**

**CUUAAG GGGAAC GAGG CCGUC CCU CG GGC GAC CGGCC CGCCA C**

**CA U A UA U A \ - AACAAC CG^ A--- A**

**zma-miRds172 CAGCUGAGCAAUUUUCUGGAC**

**CC| AUCA a caauuuu g GA**

**GC GCc gcugag cug aC \**

**CG CGG CGACUC GAC UG U**

**AC^ C--- A UAAGUUC G AA**

**zma-miRds173 UCAUGGAUUGUCAUAAUCUAG**

**AU| c C A**

**UGACCUGCu auggauugucauaaucuagGUAUCAUAAUCUAG UU A**

**ACUGGACGA UACCUAAUAGUAUUAGAUCCAUAGUAUUAGAUC AA A**

**AU^ A U U**

**zma-miRds174 GUGUGGCGUCACACGAGCUUG**

**.-GAACCA - - c-- - CGUU .-UGC A U-| C**

**UG GUgugug gcguca ac gagcuugC GCACCC GUGC CCUU--GCU CUG G**

**AC CGCGCGC CGCGGU UG CUCGGGCG CGUGGG CACG GGAA CGA GAC A**

**\ ------ G U UCC U ---- \ --- - \ UC^ A**

**zma-miRds175 UGCUGUGAGAUGCAAGAUGAA**

**CAUCUUGAAUCUAACAGCAGC**

**A| U a a C C AAUC CC A G**

**AUUUGGGAC UUcaucuug aucu acagcagcA CA UUUUAUGAA CCCU CACC UG G**

**UAAACCCUG aaguagaac uaga ugucguCGU GU AAAAUAUUU GGGA GUGG GC A**

**C^ U g g C U CUUU AA A G**

**zma-miRds176 AAGAGACUACAUGAUACAACU**

**ACU - u a caa A--- A-| GAU - GU**

**AAGAG AUaagagac ac ugaua cuCUA GCUC UA CAUA AAUAUU \**

**UUUUC UGUUUUCUG UG AUUGU GAGAU UGAG AU GUAU UUAUAA U**

**AUU A C G ACA GUGC AG^ AU- A AA**

**zma-miRds177a ACGCGCAGCGCGUCGGCAGG**

**- U- - -- a - c CCC .-CAUC| UCU**

**UGU UCG UGC acgcgc gcgcgu cgg aggGU GACGA CG A**

**ACA GGC ACG UGCGCG CGUGCA GUC UCCCA CUGCU GC C**

**C CU U CU - C C U-- \ ----^ CCC**

**zma-miRds177b ACGCGCAGCGCGUCGGCAGG**

**- U- - --| a - c CCC CAUC-- UCUACCCC CU**

**UGU UCG UGC acgcgc gcgcgu cgg aggGU GACGA CG CGU \**

**ACA GGC ACG UGCGCG CGUGCA GUC UCCCA CUGCU GC GCG A**

**U CU U CU^ - C C U-- CCAUCU CCCCAUCU CU**

**zma-miRds178 UUUGGAUCUAGAAUCAAAGGC**

**C A C .-A| G A GCACCA**

**UUAACA CCuuuggaucuagaaucaaaggcUAAUUUUAAUC CACCU ACC CC CC G**

**AAUUGU GGAAACCUAGGUCUUAGUUUCCGAUUAGAAUUAG GUGGA UGG GG GG A**

**A C A \ -^ G A ACCACU**

**zma-miRds179 AAGUGGAUUGAGGUGUAUUGAGA**

**AG| a ga ugu a UAUUU- AU**

**UGGGAUUG agug uugagg auug gaGAG AAUCU \**

**ACCCUAAU UCAC AACUUC UAAC CUCUC UUAGG U**

**AG^ C AA CC- - CCACAU GG**

**zma-miRds180a UUCGUAUUUUAGUAGAGGAGG**

**UCAU CAGAU G .-UUG A UG--|CU U U GUG**

**GCG GAACCUU UCUGCU G AGUACCUU CC GGAG GGAGGAU GUCCC A**

**CGC CUUggag agauga C UUAUGGGA GG CCUC UUUCCUG UAGGG U**

**GUUC ----- g \ --- - UGAA^CU - - AGU**

**zma-miRds180b UUCGUAUUUUAGUAGAGGAGG**

**UCAU CAGAU G .-UUG A UG--|CU U U GUG**

**GCG GAACCUU UCUGCU G AGUACCUU CC GGAG GGAGGAU GUCCC A**

**CGC CUUggag agauga c uuAUGGGA GG CCUC UUUCCUG UAGGG U**

**GUUC ----- g \ --- - UGAA^CU - - AGU**

**zma-miRds181 UUGAUGACCAGAGAUCACGAG**

**-| a a a c AG AAAUAG AU G**

**AAGGGCUAGUuug ug ccag gau acgag GAUCCAU CA GG A**

**UUCCCGAUUAAAC AC GGUC CUA UGCUC CUAGGUA GU CC U**

**A^ C C C C CU CA---- CU U**

**zma-miRds182a UGAGCUGCCAAGAAGAAUUCAA**

**AGA UCU--- -- - - uu .-aA|G A**

**AAGG ugagc ugc c aagaagaa ca GC UAGGGUGC \**

**UUCC GCUUG ACG G UUCUUCUU GU CG GUCCCACG C**

**CGA UGUAUU UC C U UC \ --^- U**

**zma-miRds182b UGAGCUGCCAAGAAGAAUUCAA**

**AGA UCU--- -- - - uu .-aA| G A**

**AAGG ugagc ugc c aagaagaa ca GC UAGGGUGC \**

**UUCC GCUUG ACG G UUCUUCUU GU CG GUCCCACG C**

**CGA UGUAUU UC C U UC \ --^ - U**

**zma-miRds182c UGAGCUGCCAAGAAGAAUUCAA**

**AGA UCU--- -- - - uu .-aA| G A**

**AAGG ugagc ugc c aagaagaa ca GC UAGGGUGC \**

**UUCC GCUUG ACG G UUCUUCUU GU CG GUCCCACG C**

**CGA UGUAUU UC C U UC \ --^ - U**

**zma-miRds185 UAUUUUUGUAGGUUAUGAGGA**

**UCAUCCCUCUUCAAAUGCA ---- U UAUGUU .-CA CCCA**

**UUCCUCA CUACGAGA UGAA UUCC GCCU \**

**Aaggagu gauguuuu auUU AAGG CGGA C**

**CGACUAUCC---------- auug u UCUUC- \ -- UGAC**

**zma-miRds186 UGGAUUGUAGGGGCUAGAAU**

**AUU UU .-UGCU| UUA**

**GA CGGG AGA U**

**CU GCCC UCU C**

**AUU U- \ ----^ CAG**

**- - .-UUGAAUU AUG**

**UUCU GUCUCUAUAAUC UCUAG \**

**aaga cggggauguuag AGGUC C**

**u u \ ------- AUA**

**- .-GUAA UA-- UG U**

**ACUC CCA AGCCU--UAGAA GAUGUG G**

**uGAG GGU UCGGG AUCUU UUGUAC C**

**g \ ---- UUGA \ UU C**

**zma-miRds187 UCCGGCAGAAACGAACAAGCC**

**C - U C AC .-CAACCAGAAUC| CCAC CAAAGU**

**CG CUU AGG CUUGUUCGUUU GUCGGAUUG GUU CUAAU U**

**GC GAA UCc gaacaagcaaa cggccuAAU CGA GAUUA U**

**A A U c ga \ -----------^ AGA- AAUAUA**
