# Supplementary material for: Deep Sequencing of Maize Small RNAs Reveals a Diverse Set of MicroRNA in Dry and Imbibed Seeds
Source: PLoS One. 2013 Jan 24;8(1):e55107. doi: 10.1371/journal.pone.0055107 (PMC3554676; doi:10.1371/journal.pone.0055107)
Supplement: Additional File S7 — Secondary structures of putative novel miRNA only identified in imbibed seed in this study. (DOC) [file pone.0055107.s007.doc]

**Additional file S7:** S**econdary structures of putative novel miRNA identified in imbibed seed**

**(**Red colored letter: mature miRNA sequence; blue colored letter: miRNA* sequence.)

**zma-miRgs1 GUGAGCUAUUCGUUGGAUCUU**

**CC UC AAC -- - .-AAA --| A ACAGGAUA**

**CCCA GCCUGGGGUC CGGAUA UCAC CAU GUUGU GC GUGGA \**

**GGGU UGGAuucuag gcuuau agug GUA CAGCA CG UAUCU A**

**AC -- guu cg A \ --- AU^ A AAGGAGCU**

**zma-miRgs2 GAACCAAAAUUGAGCCGGCGGAC**

**U UACCGUCA .-A C-| A**

**GGGCC GGAC CUAUAUG AUGU U**

**CCCGG CCUG GGUAUGC UAUA A**

**- -------- \ - UU^ U**

**CCUAAAAUAACCCUAUUACUUCUCUCUA A -- GUAGCUCC A**

**UCC GUUGGU GAUUU ACAC U**

**agg cggccg uuaaa UGUG A**

**UC-------------------------- - ag accaagCU A**

**zma-miRgs3 AGGACUUUCCUAUCUGUCUGC**

**- --- Aa- - -- u g | UU**

**CUGGG GCUA ggacuu uc cua cu ucugcC--CCUC \**

**GACCC CGGU UUUGAA AG GAU GA AGACGG GGAG C**

**G UCU AAA U UC U - \ ^ UU**

**zma-miRgs4 GCUGCGGGCUGUAUGGCGCGGA**

**A C C - uau --| a A**

**CG CGUU Ugcugcg ggcug ggcg cgg GU G**

**GC GCAG AUGACGC CCGAC CCGC GUC CG U**

**A - C G UC- AA^ G G**

**zma-miRgs5 UCAGACUCUUGUGAUCGGUGU**

**CA A--- G U - .-CA CG-- CUU- AC --| C**

**UCGGGU GCU GGUCACAA AG CUG UGGA UGCU UUCG GCAAGGCA UCUG A**

**GGUUCA ugg cuaguguu uc gac ACCU GUGA GGGC CGUUCCGU AGAU A**

**AA CAUG - c a \ -- CUAA UAGU C- UU^ C**

**zma-miRgs6a GUUCGGGGACGCAAGUGACAUA**

**U A - C G AA----- G AG A UA A GA AG-| UAUGAGCAU G**

**UUGGCAUG UGUGUCACUUG GUUCCUGAACUC UAAAAUCAG AAAAC GUACU GAACUU CCG CCCAUU AA UU GUUUG CAUCCGA UCAC U**

**AACCGUAC auacagugaac caggggcuugAG GUUUUGGUC UUUUG CAUGA UUUGAA GGC GGGUAG UU GG CAAAC GUAGGUU GGUG C**

**G G g A A CCCAGCC A CU C GC A UC CGG^ UAU------ A**

**zma-miRgs6b GUUCGGGGACGCAAGUGACAUA**

**U A - C G AA G AG A UA A A A AU---------| G**

**UUGGCAUG UGUGUCACUUG GUUCCUGAACUC UAAAAUCA GAAAAC GUACU GAACUU CCG CCCAUU AA UCG GUUUG GC UCAC U**

**AACCGUAC auacagugaac caggggcuugAG GUUUUGGU CUUUUG CAUGA UUUGAA GGC GGGUAG UU GGU CAAAC CG GGUG C**

**G G g A A CC A CU C GC A C - GGUAGGUUUAU^ A**

**zma-miRgs7a UGGAGAUGUGCUACAACUUGAC**

**UA U- UGC CAA - - | CUC**

**UUUA GG UCAAGUUGUA GCA CUCC CC--GGAC \**

**GAGU CC aguucaacau ugu gagg GG CCUG A**

**CA UU c-- cg- a u \ ^ AAC**

**zma-miRgs7b UGGAGAUGUGCUACAACUUGAC**

**---- CAA - - .-GAACCUCA U**

**UCAAGUUGUA GCA CUCC CC CAAG C**

**aguucaacau ugu gagg GG GUUU C**

**UCCc cg- a u \ -------- A**

**zma-miRgs7c UGGAGAUGUGCUACAACUUGAC**

**U AUUUAU UGC CAA - - | CUC**

**GUUU GG UCAAGUUGUA GCA CUCC CC--GGAC \**

**CAGA CC aguucaacau ugu gagg GG CCUG A**

**- GUUU-- c-- cg- a u \ ^ AAC**

**zma-miRgs7d UGGAGAUGUGCUACAACUUGAC**

**U AUUUAU UUC ACA U - | CUC**

**GUUU GG UCAAGUUGU AGCAC UC CC--GGAC \**

**CAGA CC aguucaaca ucgug ag gg CCUG A**

**- GUUU-- c-- --- u a \ ^ AAC**

**zma-miRgs7e UGGAGAUGUGCUACAACUUGAC**

**U AUUUAU UGC CAA - .-CC| CUC**

**GUUU GG UCAAGUUGUA GCA CUCC GGAC \**

**CAGA CC aguucaacau ugu gagg CCUG A**

**- GUUU-- c-- cg- a \ --^ AAC**

**zma-miRgs7f UGGAGAUGUGCUACAACUUGAC**

**AUAUUUGU UGC - AG-- U .-C| CUC**

**GG UCAAGUUGUA CA UCC CCU GGAC \**

**CC aguucaacau gu agg GGA CCUG A**

**CAGAGUUU c-- c guag u \ -^ AAC**

**zma-miRgs8a GGAGGAGAUUGGAGGGGCUAA**

**- C g a ACC---| C A**

**CCA AAAAUCg agg gauuggaggggcuaaAAU CU AUUCA U**

**GGU UUUUGGC UCC CUAAUCUCCCCGAUUUUA GA UAAGU U**

**A - A C AGAAAA^ A U**

**zma-miRgs8b GGAGGAGAUUGGAGGGGCUA**

**UCGUUUCACA AGCG- | A AU**

**UAGCCCUUC UCUUCCGAA--GGGU CC \**

**aucggggag aggaggUUU CCUA GG U**

**UUACCCUUAG guuag \ ^ C AC**

**zma-miRgs8c GGAGGAGAUUGGAGGGGCUAA**

**UC| U - a C- U A**

**CAAA ACCgga gg gauuggaggggcuaaAAUC CUUCUUAU CA A**

**GUUU UGGCUU UC CUAAUCUCCCCGAUUUUAG GAGGAAUA GU A**

**GU^ - G - AU U U**

**zma-miRgs8d GGAGGAGAUUGGAGGGGCUAA**

**-| UAAU a u C A**

**CAAGGG ggagg gauuggaggggc aaAAUUC CUUGCUAUUCA A**

**GUUCCC UCUCC CUAACCUCCCCG UUUUAGG GAACGAUAAGU U**

**C^ UU-- C U A U**

**zma-miRgs8e GGAGGAGAUUGGAGGGGCUAA**

**- AA a - --| A**

**CCC GAACCggagg gauuggaggggcuaaAAUC CC CUUAUUCA A**

**GGG UUUGGCUUCC CUAACCUCCCCGAUUUUAG GG GAAUAAGU A**

**C CC C A AA^ C**

**zma-miRgs9a UGAGUUGGAGAUUAUGAGGAUG**

**GUGGUGGAAAA GGACG G AAAUUACA UG .-ACUAA AU**

**GCGUAU GUAA CUCUAACUUAUG UGGU AC AUUG A**

**UGCgua uauu gagguugaguAC GCCA UG UGAC A**

**----------- ggag- a GAGCAA-- GU \ ----- GU**

**zma-miRgs9b UGAGUUGGAGAUUAUGAGGAUG**

**CG C -| C G U - AAC**

**UCUUC CGUA CCUCGUG UCU CC GAC CG G**

**AGGAG GCgu ggaguau aga gg uug gu A**

**AG U a^ u - - a ACG**

**zma-miRgs10 GACAAUUUACUCGUCGGUGCA**

**A AA AGG U U U A GC .-GC | GACG**

**GG GGUCC GC C CG ACGAG AGGUUG CGC GGGAGAACGG--GCC \**

**CC CCAGG cg g gc ugcuc uuuaac gCG CCCUCUUGUC CGG C**

**- CC Ga- u - - a a- \ -- \ ^ AAUA**

**zma-miRgs11 UGACGGUUUAUAUGGCAAUGGC**

**G CAGA GC U G - .-ACC .-A| A CUCCCCU C**

**GAAAUU GCUG UGCC UGU AGAUUG CA UUUCCUUGC GAU UCUGGGU UGU U**

**CUUUAG cggu acgg aua uuuggc gu AGAGGGACG CUA GGACCUA ACA U**

**- CUAA a- u - a \ --- \ -^ A UUAAAU- U**

**zma-miRgs12a GAUGUUGAUCCAAGGGACACA**

**- aca | C A UUU**

**UGGGCCACAUgauguugauccaaggg caUG--GG GUGG UAA U**

**ACUUGGUGUACUACAACUAGGUUCCC GUAC CC CACC AUU A**

**G CCC \ ^ - A CAC**

**zma-miRgs12b GAUGUUGAUCCAAGGGACACA**

**- - aca .-C| A UUA**

**UGAACCAUAUgauguugauccaa gggac UGGA GUGGAUA UU C**

**ACUUGGUGUACUACAACUAGGUU CCCUG ACCU CGCCUGU AA A**

**G C CAC \ -^ C UUC**

**zma-miRgs13a UGAACCUUUGGACUAAAGUGCA**

**-| AAAG A uuug - aAAAACCA**

**GCA UG ugaacc gacu aaagugc A**

**UGU AC ACUUGG CUGA UUUUAUG G**

**G^ AUAA - UUUA A AGUAAACC**

**zma-miRgs13b UGAACCUUUGGACUAAAGUGCA**

**-| AAAG A uuug - AAAAACCA**

**GCA UG ugaacc gacu aaaGUGC A**

**UGU AC ACUUGG CUGA UUUUAUG G**

**G^ AUAA - UUUA A AGUAAACC**

**zma-miRgs13c UGAACCUUUGGACUAAAGUGCA**

**-| AAAG A uuug - aAAAACCA**

**GCA UG ugaacc gacu aaagugc A**

**UGU AC ACUUGG CUGA UUUUAUG G**

**G^ AUAA - UUUA A AGUAAACC**

**zma-miRgs13d UGAACCUUUGGACUAAAGUGCA**

**-| AAAG A uuug - aAAAACCA**

**GCA UG ugaacc gacu aaagugc A**

**UGU AC ACUUGG CUGA UUUUAUG G**

**G^ AUAA - UUUA A AGUAAACC**

**zma-miRgs13e UGAACCUUUGGACUAAAGUGCA**

**- AG--------| A uuug - aAAAACCA**

**GCAAA UG ugaacc gacu aaagugc A**

**CGUUU AC ACUUGG CUGA UUUUAUG G**

**A AGGGAUUAAA^ - UAUA A AAUAAACC**

**zma-miRgs14a AAUCCGACGCAAACGAACAAGG**

**A ACAAACC A .-GU| UCGA**

**CCGA CU AGGCCUUGUUCGUUUGUGUCGGAUUG GGG A**

**GGCU GA UCUGgaacaagcaaacgcagccuaaC CCU A**

**- ------- A \ --^ UAAC**

**zma-miRgs14b AAUCCGACGCAAACGAACAAGG**

**ACCCCUAAU A AUG-- UGUC-| UCUAU**

**GCCUUGUUCGUUUGUGUC GAUUG GAGAUUGUU UAAUU A**

**Cggaacaagcaaacgcag cuaaC CUUUAGCAA AUUAG A**

**CGACCGAAU c GUGGG UGUCA^ UUUCA**

**zma-miRgs14c AAUCCGACGCAAACGAACAAGG**

**- GG G--| a g U**

**UUCCG U Caauccg cgcaaacgaacaag AC A**

**AAGGC G GUUAGGC GUGUUUGUUUGUUC UG A**

**C UU GAG^ C - A**

**zma-miRgs15a AGAGGAUGAGAUGACUACCUGG**

**AAG GACAA CC A - .-CCCCUA GCCGA A - GC -| CUG**

**GGGC GGGU UCAUC UCG CCUC GC CAC UUCU UCGU GCGG CAUC \**

**CUCG ucca aguag agu ggag CG GUG GAGA AGUA CGUC GUAG A**

**ACA AAAgg uc - a \ ------ AAUCA A G AA U^ UCA**

**zma-miRgs15b AGAGGAUGAGAUGACUACCUGG**

**AAG GACAA CC A - .-CCC - GCCGA A - --| C CUG**

**GGGC GGGU UCAUC UCG CCUC CUG GC CAC UUCU UCA UGCA GGCAUC \**

**CUCG ucca aguag agu ggag GAC CG GUG GAGA AGU ACGU CUGUAG A**

**ACA AAAgg uc - a \ --- A AAUCA A G AA^ - UCA**

**zma-miRgs15c AGAGGAUGAGAUGACUACCUGG**

**AAG GACGA CC A - .-CCC - GCCGA A - GC -| CCUG**

**GGGC GGGU UCAUC UCG CCUC CUG GC CAC UUCU UCGU GCGG CAU \**

**CUCG ucca aguag agu ggag GAC CG GUG GAGA AGUA CGUC GUA A**

**ACA AAAgg uc - a \ --- A AAUCA A G AA U^ AUCA**

**zma-miRgs15d AGAGGAUGAGAUGACUACCUGG**

**AAG GACGA CC A - .-CCC - GCCGA A - GC -| CUG**

**GGGC GGGU UCAUC UCG CCUC CUG GC CAC UUCU UCAU GCGG CAUC \**

**CUCG ucca aguag agu ggag GAC CG GUG GAGA AGUA CGUC GUAG A**

**ACA AAAgg uc - a \ --- A AAUCA A G AA U^ UCA**

**zma-miRgs16a CAAUUUAGGGACUAAAAUAAA**

**U U A AAU-- CAAA U-| A**

**UG AGAG CU UUUUAGUCUUUAAAUUGCUAAACA GACUAAAAUA GA U**

**AC UCUC GA aaaaucagggauuuaacGAUUUGU CUGAUUUUAU CU U**

**- - A Gaaau AUAC UU^ A**

**zma-miRgs16b CAAUUUAGGGACUAAAAUAAA**

**U| CC A U UA**

**UUUAUUUUAUU AUUUUAGUCCUUAAAUU CUAAAUACGGAA CUAAA G**

**AGGUAAAAUAa uaaaaucagggauuuaa GAUUUAUGCCUU GAUUU A**

**-^ aa c U UG**

**zma-miRgs16c CAAUUUAGGGACUAAAAUAAA**

**CUAAA| CC AC CU**

**UUUAUU AUUUUAGUUCUUAAAUU UAAAUACGGAAACUAAAA \**

**AAAUAa uaaaaucagggauuuaa GUUUAUGCCUUUGAUUUU C**

**AAGUA^ aa cA AU**

**zma-miRgs16d CAAUUUAGGGACUAAAAUAAA**

**C| C ACG AG CU**

**UGCAUUUUAUUU AUUUUAGUUCCUAAAUU AAAUAU AAACUAAAA \**

**AUGUAAAAUAaa uaaaaucagggauuuaa UUUAUG UUUGAUUUU U**

**-^ a cAA CU AU**

**zma-miRgs16e CAAUUUAGGGACUAAAAUAAA**

**UAUA| A C AC UAUAUAGC CA**

**UUUUAUU UAU UUAGUCCCUAAAUU UAAAUA AACUAAAU \**

**GAAAUAa aua aaucagggauuuaa GUUUAU UUGAUUUA G**

**CUGA^ a a cA UUUU---- CA**

**zma-miRgs16f CAAUUUAGGGACUAAAAUAAA**

**UUUA| C GCA UC AG CU**

**UUUUAUUU AUUUUAGU UAAAUUG AAAUAU AAACUAAAA \**

**GAAAUAaa uaaaauca auuuaac UUUAUA UUUGAUUUU C**

**CAGA^ a ggg GA CU AU**

**zma-miRgs16g CAAUUUAGGGACUAAAAUAAA**

**UC| C A C G A CU**

**UAUUUUAUU UAUUUUAGUC UUAAAUUGC AAAUA GAAAACU AAA \**

**AUAAAAUAa auaaaaucag gauuuaacG UUUAU CUUUUGA UUU U**

**AA^ a g A A A AU**

**zma-miRgs17a AGAGGGGAUUGGAGGGGCUAA**

**-| AUa g - A**

**CAAGGGAA gaggggauuggag ggcuaaAAUC CCU G**

**GUUCCCUU CUCCCCUAACCUC CCGAUUUUAG GGA C**

**G^ AC- G U A**

**zma-miRgs17b AGAGGGGAUUGGAGGGGCUAA**

**U A - g CU - --| A**

**CUA AAA CUagaggggauugga gggcuaaAAU CC UC AUUCA A**

**GGU UUU GGUUUCCUCUAACCU CCCGAUUUUA GG AG UAAGU A**

**C A A G AG A AA^ U**

**zma-miRgs17c AGAGGGGAUUGGAGGGGCUAA**

**- - Ua -| c CA**

**GUCAC AAAC gaggggauugga gggg uaaAAUCCCUUCCUUAUU A**

**CGGUG UUUG CUCCCCUAACCU CCCC AUUUUAGGGGAGGAAUAA A**

**U U GC A^ - AA**

**zma-miRgs17d AGAGGGGAUUGGAGGGGCUAA**

**U AAU-| a C C U**

**CCA ACC gaggggauuggaggggcuaaAAUCC CUCCUUAUU AA A**

**GGU UGG CUCCCCUAAUCUCCCCGAUUUUAGG GAGGAAUAA UU U**

**C GUUU^ C A A U**

**zma-miRgs18a AGGAGUCGCUCGAGCUUUCGUC**

**U U C--- U- -- G GUC**

**UGG GA GGCGA GUU GGCGGC UCCUC \**

**ACC CU cugcu cga ucgcug aggaG G**

**C - AAGA uu gc - GCU**

**zma-miRgs18b AGGAGUCGCUCGAGCUUUCGUC**

**UU GA U- -- G AUC**

**GGU CGGCGA GUU GGCGGC UCCUC \**

**CUA Gcugcu cga ucgcug aggaG G**

**-- AG uu gc - GCU**

**zma-miRgs18c AGGAGUCGCUCGAGCUUUCGUC**

**UU-- GA U- -- G GUC**

**GGU CGGCGA GUU GGCGGC UCCUC \**

**CUA Gcugcu cga ucgcug aggaG G**

**UACC AG uu gc - GCU**

**zma-miRgs18d AGGAGUCGCUCGAGCUUUCGUC**

**UU-- GA U- -- G GUU**

**GGU CGGCGA GUU GGCGGC UCCUC \**

**CUA Gcugcu cga ucgcug aggaG G**

**UACC AG uu gc - GCU**

**zma-miRgs18e AGGAGUCGCUCGAGCUUUCGUC**

**UU GA U- -- G GUC**

**GGU CGGCGA GUU GGCGGC UCCUC \**

**CUA Gcugcu cga ucgcug aggaG G**

**-- AG uu gc - GCU**

**zma-miRgs19a UGGGACGGAUGUAGAUAGGAGU**

**UUCUACUGCAAUUGCCACACU**

**U U U - a a a uCUUA-- AUAA GCU AU-- A U .-AAC| UU**

**GCA GUU Gug gg cgg uguag uaggag GCAGG GCA GAGUG AGGG AUG UGGGAG CAG \**

**CGU CAG cac cc guu acguc aucuuC CGUCC CGU UUUAC UUCC UAC ACCUUU GUC G**

**U - u a - a - UAUUCCC A--- AGU GAUU - - \ ---^ UU**

**zma-miRgs19b UGGGACGGAUGUAGAUAGGAGU**

**UUCUACUGCAAUUGCCACACU**

**U U U - a a a uCUUA-- .-A | GCU U GGAAU UG G C**

**GCA GUU Gug gg cgg uguag uaggag GC GGAU--AAGCG GAG GGUAG GU GGA AA C**

**CGU CAG cac cc guu acguc aucuuC CG CCUA UUCGC UUU CCAUC UA CUU UU A**

**U - u a - a - UAUUCCC \ - \ ^ AGU - AU--- GU G G**

**zma-miRgs19c UGGGACGGAUGUAGAUAGGAGU**

**U UCCU - - G G A .-UC| U**

**UUCUGUUG UUCCU UUUGCA CUGUU CCA AA--GU UUCUUGU UGAUUU \**

**AGGACGAU gagga agaugu ggcag ggu UU CA AAGGACG AUUAAG G**

**- UCu- u a - G \ A \ --^ A**

**zma-miRgs20a UCAAUACACAUGGAUUAAGGU**

**UCAA| C AC A A C A C C A**

**GUAUU AC UAAUUCAUGUG AUU AG UA AUUGAGG GUAA UUA \**

**CAUAA ug auuagguacac uaa uC AU UAAUUCC CAUU AAU A**

**AGAA^ A ga a c U A A U U**

**zma-miRgs20b UCAAUACACAUGGAUUAAGGU**

**UC| A C CA A A UGUUGU AG**

**UCGU UUCACCU AAUUCA UGUAUUGA GUGGA UG AAAUU U**

**AGCA AAGugga uuaggu acauaacu CACCU AC UUUGA U**

**AU^ C a ac C A CU---- CU**

**zma-miRgs21a UUUGACCAAGUUUGUAGAAAA**

**- U c A UA ----| A AUUU**

**CUAUGC AAUuuugac aaguuuguagaaaaG AUUAACAUGUA CAUC AUG UACGU \**

**GAUACG UUAAAACUG UUUAAAUAUCUUUUC UAAUUGUACAU GUAG UAC AUGUA U**

**U U C A GG CUUC^ - AUAC**

**zma-miRgs21b UUUGACCAAGUUUGUAGAAAA**

**- A -| UUGUC C AA U ACUAU A-- U**

**CUAUUUUA Guuugaccaaguuugua gaaaaGU GA AUUUAUG ACC AAUAAGUAU GA AAUAUG \**

**GAUAGAAU CAAACUGGUUCAAAUAU UUUUUCA CU UAAAUAU UGG UUAUUCAUA CU UUGUAC U**

**U A G^ UGA-- A AA U GUAAU AAG A**

**zma-miRgs21c UUUGACCAAGUUUGUAGAAAA**

**-| Cu A UUUCCCAUAUA UAA C GC- GUUG**

**UUUCUCUAA uugaccaaguuuguagaaaaA UGU ACCAAU AUACA UAUCAAG AUUUCAU G**

**GAAGAGAUU GACUGGUUCAAAUAUCUUUUU ACA UGGUUG UAUGU GUAGUUU UAAAGUA A**

**U^ U- - ----------- UA- U AAU GUUC**

**zma-miRgs21d UUUGACCAAGUUUGUAGAAAA**

**AA UU---| c AGA C GUACCUAA GUAUUA AG CA**

**ACUU ACuuugac aaguuuguagaaaa UAUUAAUAUCU CA UAAAU UCA AGAUUU U**

**UGAA UGAAAUUG UUCAAAUAUCUUUU AUAGUUAUAGA GU GUUUA AGU UCUAGG G**

**UC UAGAU^ U CAC U AGCA---- AUCAA- AA UG**

**zma-miRgs21e UUUGACCAAGUUUGUAGAAAA**

**- Cu a | UU**

**UUUCUCUAA uugacca guuuguagaaaaA--AUAU U**

**GAAGAGAUU GACUGGU CAAAUAUCUUUUU UAUA C**

**U U- C \ ^ CC**

**zma-miRgs21f UUUGACCAAGUUUGUAGAAAA**

**UUCUAUGAAUUUGGUCAAACA**

**- AC ug CC - .-GAAAUUG| C AC**

**CUUCUCUA uuugaccaaguu uagaaaaAUAUAUUU AUCUAAGAUA CAAA CACUAU AAG \**

**GAAGAGAU aaacugguuuaa aucuuUUUAUGUGGA UAGAUUUUGU GUUU GUGGUA UUC A**

**U ac gu UU A \ -------^ C AU**

**zma-miRgs21g UUUGACCAAGUUUGUAGAAAA**

**CUUAA Cu gac g .-CAC| CC CA**

**CUAA uu caaguuu uagaaaaAAG GG GU \**

**GAUU AA GUUCAAA AUCUUUUUUU CC CA A**

**UUGAA AU GUA A \ ---^ A- AC**

**zma-miRgs21h UUUGACCAAGUUUGUAGAAAA**

**CUUC C u aG C | AAAUAAAUUGCA A CA**

**UCUAA uuugaccaaguu guagaaa UACAUUAG AUCUA--UGAAAC CUAUCA GA U**

**AGAUU AAACUGGUUUAA UAUCUUU AUGUGGUU UGGAU ACUUUG GGUGGU CU A**

**GUAA C U CU U \ ^ GUACUGAUAUA- A UU**

**zma-miRgs22 UGAGAUUGAUAUUGAUGAGCA**

**UGGCCA - a g .-gca| UC**

**CUG Cugagauug uauu auga AUCCA \**

**GGC GACUUUAAC AUAG UAUU UAGGU A**

**AGUUAA U - - \ ---^ UC**

**zma-miRgs23 AAGAUCUAUGGCACCGACCCC**

**GUU---- ag ua c--- ---| ACUU CA**

**CGGCGUCa auc uggcaccgac ccUA GG GC C**

**GCUGCGGU UGG ACCGUGGUUG GGAU CC CG A**

**GCGUGUC CA UA AAAA GUA^ GUU- CU**

**zma-miRgs24a AGGGAGUGUUAGAUGGUCAGUU**

**A| U g aga - GUAUUUA- A**

**UAGCA UUAag gaguguu uggu caguu AUUA U**

**AUCGU AAUUC UUCACAG AUCA GUCGA UAAU U**

**A^ U G --- A AGCAAUAG U**

**zma-miRgs24b AGGGAGUGUUAGAUGGUCAGUU**

**CU U U C U AA .-U| U CUAC**

**UUAGA UACAACU GCU UCUG CGCUC UUA GUAAAA GGG \**

**GAUUU AUGuuga ugg agau gugag aAU CAUUUU UCU A**

**UU - c u u gg \ -^ - AUCA**

**zma-miRgs25 GGAUUGGAUGCGUAUGUAGGU**

**G U AAg g - .-gu| AU**

**GU GAUU gauug aug cguauguag UUG \**

**CA CUAG CUAGU UGC GUAUACAUC AGC G**

**A C AAA G U \ --^ UA**

**zma-miRgs26a GGAGGGAAUUGGAGGGGCUAA**

**-| UC a g CC A**

**CC AAACCUggaggg auu gaggggcuaaAAUCCCCU UUAUUCA U**

**GG UUUGGGCUUCCC UAA UUCCCCGAUUUUAGGGGA AAUAAGU U**

**A^ U- C A AA U**

**zma-miRgs26b GGAGGGAAUUGGAGGGGCUAA**

**UC U-| C a g A U U A**

**CAAA AC ggaggg auuggaggg cuaaA UCCCUUCC UAU CA A**

**GUUU UG CCUCUC UAACCUCCC GAUUU AGGGGAGG AUA GU A**

**CU UU^ A C A A U U U**

**zma-miRgs26c GGAGGGAAUUGGAGGGGCUAA**

**C| AC g C A**

**UAAAAU Uggagggaauuggaggg cuaaAAUCCCCUUCUUA UCA A**

**GUUUUA GCCUCCCUUAACCUCCC GAUUUUAGGGGAAGAAU AGU A**

**U^ -- A A U**

**zma-miRgs26d GGAGGGAAUUGGAGGGGCUAA**

**CC -| a g AA A**

**UCAAAAA Uggaggg auuggaggg cuaaAAU CCUCCUUAUUCA A**

**GGUUUUU GUCUCCC UAACCUCCC GAUUUUA GGAGGAAUAAGU A**

**CA G^ C G GG U**

**zma-miRgs26e GGAGGGAAUUGGAGGGGCUAA**

**UC| C c - C A**

**CAAAACC ggagggaauuggagggg uaaAAUCC CCUC UUAUUCA U**

**GUUUUGG UCUCCCUUAACUUCCCC AUUUUAGG GGAG AAUAAGU U**

**GU^ - U U - U**

**zma-miRgs27a UGAUCUGUAAAGGCAUCCCUGU**

**G AGAACAG CAC CG .-UU C ACCA .-AA --| G**

**GAC GCAGGGA CCUU C GAU GGC GACG GAC CG C**

**CUG ugucccu ggaa g UUA CCG UUGC CUG GC G**

**- ACAUUAA ac- au \ -- C GCC- \ -- AA^ A**

**zma-miRgs27b UGAUCUGUAAAGGCAUCCCUGU**

**G AGAACAG CAC CG UU - .-CCAGACGAAGACCGGC| A AUCCC**

**GAC GCAGGGA CCUU C GAUC GGCA GACGA GUC A**

**CUG ugucccu ggaa g cuag CCGU CUGCU CAG C**

**- ACAUUAA ac- au u- u \ ----------------^ C CGUGA**

**zma-miRgs27c UGAUCUGUAAAGGCAUCCCUGU**

**G AGAACAG CAC CG UU - A A- GAAGAC G .-AAGUCAUCC| A C GUCC**

**GAC GCAGGGA CCUU C GAUC GGC CC GAC CGGC ACG CAC GUG GGCCUC A**

**CUG ugucccu ggaa g cuag CCG GG UUG GCCG UGC GUG CAC CCGGAG A**

**- ACAUUAA ac- au u- u - CA AGCGCC G \ ---------^ - - ACUC**

**zma-miRgs27d UGAUCUGUAAAGGCAUCCCUGU**

**G GGAACAG CAC CG UU - A A- GAAGAC G .-AAGUCAUCC| A C GUCC**

**GAC GCGGGGA CCUU C GAUC GGC CC GAC CGGC ACG CAC GUG GGCCUC A**

**CUG ugucccu ggaa g cuag CCG GG UUG GCCG UGC GUG CAC CCGGAG G**

**- ACAUUAA ac- au u- u - CA AGCGCC G \ ---------^ - - ACUC**

**zma-miRgs27e UGAUCUGUAAAGGCAUCCCUGU**

**G AGAACAG CAC CG UU - A A- GAAGAC ACGAA .-AUCCAACA| C GUCU**

**GAC GCAGGGA CCUU C GAUC GGC CC GAC CGGCG GCC GUG GGCCUC G**

**CUG ugucccu ggaa g cuag CCG GG UUG GCCGC CGG CAC CUGGAG G**

**- ACAUUAA ac- au u- u - CA AGC--- ----- \ --------^ - ACUC**

**zma-miRgs28a UUCUCCAGGAGUUGAUGGACAA**

**AAAUA G A U- - .-UGGA| C GA - UG U --- .-G .-GAAG C**

**GUU AU CAGCUCCUGGAGG UC CUGGG UC CU UGCA G GCUG AAAG GAC CUGGCCC GCCA U**

**cag ua guugaggaccucu AG GGCCC AG GA ACGU C CGAC UUUC CUG GACUGGG CGGU G**

**Uaa-- g - uC A \ ----^ U G- U GU C UCU \ - \ ---- A**

**zma-miRgs28b UUCUCCAGGAGUUGAUGGACAA**

**GUCUUCGACUUGUGCGCGCUGU**

**- GGA uucuc- g u a .-GGGGCA U**

**UCCC GAC ca gaguuga ggac aUG ACCUGG G**

**GGGG Uug gu uucagcu ucug UAC UGGACC U**

**U AUG ucgcgc g - - \ ------ A**

**zma-miRgs29 UUCUUUGUAGAUUGUACCCGCA**

**AU u- aga gu--- g | UAGA AG**

**UCUACUUUuucu ugu uu accc caUA--GGU GUUGG A**

**AGAUGAGAAGGA ACG AG UGGG GUAU CCA CAACC A**

**AC UU CG- AUUAU - \ ^ CCCC AG**

**zma-miRgs30 UCGCUAGUUUGGUAUUAUG**

**C u ---| UA**

**AGCUGAGCUucgcuag uugguauuaugGAGCACACACCCGCGCCAGUU GAU \**

**UCGACUCGAAGCGGUC AACCAUAAUACCUUGUGUGUGGGCGCGGUCAA CUG A**

**A C ACC^ AU**

**zma-miRgs31a AAGAGGUCUAUGUGAAGAAGCU**

**- C U ag- c ga a .-CCA .-CUU| U G UUC**

**GA GGG CCGa aggu uaugu ag agcu GUGGU GA GUA UCAUCACA \**

**CU CUC GGCU UCUA GUACG UU UCGA CACCG CU CGU AGUAGUGU U**

**U C C AGA C AA A \ --- \ ---^ U G CGU**

**zma-miRgs31b AAGAGGUCUAUGUGAAGAAGCU**

**AAC U ag- c ga a .-GUCUUGAUGUUG| UUC**

**GGG CCGa aggu uaugu ag agcu--CCAGUG UCAUCACA \**

**UCC GGCU UCUA GUACG UU UCGA GGUUAC AGUAGUGU U**

**UCC - AGA C AA A \ \ ------------^ CGU**

**zma-miRgs32a UUAGCAAGGGAGUAACGACU**

**\**

**CAAG A A-- A .-AGAUGAAGAAGAC| CAACGA AUACGCA UGG**

**AGAGC AGUUGUUG UCUUG UGAUGU GUUC CCGCA CCA \**

**UCUCG ucagcaau ggaac auuAUA CAAG GGCGU GGU C**

**UAGA C gag g \ -------------^ AACACC GUAGA-- UAA**

**zma-miRgs32b UUAGCAAGGGAGUAACGACU**

**CAAG CA A-- A .-A .-AAGAAGAC .-AACAGC| UA AC**

**AGAG AGUUGUUG UCUUG UGAUGU GAUG GUUCC CGCUA CGC C**

**UCUC ucagcaau ggaac auuAUA CUGC CAAGG GCGGU GCG A**

**UAGA AC gag g \ - \ -------- \ ------^ UA GU**

**zma-miRgs32c UUAGCAAGGGAGUAACGACU**

**CAAG A A-- A .-AGA| .-GAAGAC AAC AUACGCA UGG**

**AGAGC AGUUGUUG UCUUG UGAUGU UGAA GUUCC GGCCGCA CCA \**

**UCUCG ucagcaau ggaac auuAUA GCUU CAAGG CCGGCGU GGU C**

**UAGA C gag g \ ---^ \ ------ AUA GUAGA-- UAG**

**zma-miRgs32d UUAGCAAGGGAGUAACGACU**

**CAAG A A-- A .-A .-AAGAAGAC .-CAACGA| CAAUA C G AA**

**AGAGC AGUUGUUG UCUUG UGAUGU GACG GUUC CUG CGCAC AU UC \**

**UCUCG ucagcaau ggaac auuAUA CUGC CGAG GAC GCGUG UA AG U**

**UAGA C gag g \ - \ -------- \ ------^ ACCG- - G GU**

**zma-miRgs32e UUAGCAAGGGAGUAACGACU**

**CAAG A A-- A .-AGAUGAA .-AC| U**

**AGAGC AGUUGUUG UCUUG UGAUGU GGAG GU C**

**UCUCG ucagcaau ggaac auuAUA CCUC CA C**

**UAGA C gag g \ ------- \ --^ A**

**zma-miRgs33a AUGCACUAGAGCUAAUGGUUA**

**- - --| A**

**GGGUGUUUGAaugcacuagagcuaaugguuaG UGGCUAAAA UUGC C**

**CCCACAAACUUACGUGAUCUCGAUUAUCAAUC AUCGAUUUU AACG U**

**C A UA^ A**

**zma-miRgs33b AUGCACUAGAGCUAAUGGUUA**

**.-G ---- -| g U GUUA AAA**

**GGU GUUUGAau gcacuaga cuaaugguuaGC AGCUAAUAA CUAAC \**

**UCG UAGAUUUA CGUGGUUU GAUUAUCAAUUG UCGAUUAUU GGUUG A**

**\ - AUGU U^ - - AAUC AUA**

**zma-miRgs33c AUGCACUAGAGCUAAUGGUUA**

**UCA g - AGACAUCCAAAC --| U**

**UGUUUGAaugcacuagagcuaaug uuaGUUG CUAAA--AUUAGUUAA AGU AUAGCUAA A**

**ACAAACUUACGUGAUUUCGAUUAU AAUUAAC GAUUU UAAUCGAUU UCA UAUCGAUU G**

**CUG A U \ AAACUACUUUCA AU^ U**

**zma-miRgs33d AUGCACUAGAGCUAAUGGUUA**

**U A - -| CCUAACUA**

**GUUAGCUA CUAACUAUUAGCUCUA UGCAUUCAAA CAC \**

**UAAUCGAU Gauugguaaucgagau acguaAGUUU GUG U**

**- C c C^ AUCUUUAU**

**zma-miRgs34a UCGGCUAGCCCAUGUCGGUAUG**

**AAAAU--------- A - G**

**CCGACA GGGUUGGCU AUG C**

**ggcugu cccgaucgg uGC A**

**GGUUUAAGGAguau a c U**

**zma-miRgs34b UCGGCUAGCCCAUGUCGGUAUG**

**AAAAU--------- A - G**

**CCGACA GGGUUGGUU AUG C**

**ggcugu cccgaucgg uGC A**

**GGUUUAAGGAguau a c U**

**zma-miRgs35a UGCGUGAUGCGGAGGACUUGGCA**

**GG-------- G G U-- U UUU AA UG**

**UGC AAGUCCU CC UCGCGC UAA GCGG CCG \**

**acg uucagga gg agugcg GUU CGCC GGC A**

**GCUCGGCGUG g - cgu U UC- -- CC**

zma-miRgs35b **UGCGUGAUGCGGAGGACUUGGCA**

**U U - GGGGU UG C G - - U- - GAAG CCC C | AC**

**CG GG CGUAC C GG CCUU GCG CACG GC GGGC GCUGGGU UAUG UCG CAGAU--GCCUUUUC \**

**GC UC GCGUG g uc ggag cgu gugc uG UCCG CGGCCCA GUGC AGU GUUUA UGGGAAGG G**

**- - G ac--- gu a g a g UU C ---- CAA - \ ^ AG**

**zma-miRgs35c UGCGUGAUGCGGAGGACUUGGCA**

**UCUGGCCCUUGGCGCACGGCUG**

**U U - GGG| u c g - .-gcu - G AGUG**

**CG GG CGUAC Guc gg ccuu gcg cacg gGGCA CUGG CGA \**

**GC UC GCGUG cgg uc cgag cgu gugc UCCGU GACC GCU U**

**- - G A--^ u a g a \ --- A - CCCG**

**zma-miRgs36a GUGGAUUGAGUGGGAUUAGGU**

**- AUC ag a .-u| UUU**

**CUCA CAUguggauug ugggauu gg GGG \**

**GGGU GUGUACCUAAC ACCCUAA CC CCC A**

**A AAU CU - \ -^ UAA**

**zma-miRgs36b GUGGAUUGAGUGGGAUUAGGU**

**AAGC A G - UU G G .-CUAGA| UC AUAGA U**

**AAAC UGA UU GUCCC G UCAAU CAC GUG UGG UG G**

**UUUG ACu ga uaggg u aguua gug CAC ACC AC U**

**UAAA - g u -- g g \ -----^ UA AACCG G**

**zma-miRgs36c GUGGAUUGAGUGGGAUUAGGU**

**- UC ag - .-agg| UUU**

**CUUAA CGUguggauug ug ggauu uGGG \**

**GGAUU GUACGCCUAAC AC CCUAA ACUC A**

**A GU CU U \ ---^ UAA**

**zma-miRgs37 CUAUCUGCUGUAGUGGUGAAC**

**G - c - - --- GAA .-GUUUGUCUAA| U**

**UUUUCAGGG cuaucug ugua g uggugaacG UAUA ACGU CCUGU U**

**GGAAGUUCU GAUAGAU GCAU C ACCAUUUGU AUGU UGCG GGACG U**

**A G U A G UUA AUA \ ----------^ U**

**zma-miRgs38 UUUGAUGCUUACAUGUAGAUGG**

**UUUAUU - g ga .-AUAAAAGGAUCAAG AUAG GCUG .-AU -| GU**

**UAUA uuugau cuuacaugua ugg GUU UUUGCGUU UUUCAU AUG UUAUU C**

**GUGU AGAUUG GAAUGUACGU ACC CAG GAACGUAA AAAGUA UGC GGUAG A**

**GGGGUU A G A- \ -------------- GAG- AAUA \ -- U^ UA**

**zma-miRgs39 UGGUUGGCGAUGGUGUGCAC**

**- GAUG - aug c .-UG G UUGUCCC A --- - .-CU --- -| UG**

**AGUGG Uug guuggcg gugugca CUAUUGC GUU UGA UGUCGG GUC AGA CUACAUCGGC GACCCA AC UUCUGC G**

**UCGCC AAC CAGCCGC UACAUGU GGUAACG CAA ACU ACAGUC CAG UCU GAUGUGGUCG UUGGGU UG GGGGCG A**

**G ACA- U AG- A \ -- - UUUUAU- - GUU C \ -- UCC U^ UC**

**zma-miRgs40 AAUCCCCUCCGGUUUUAUGAC**

**- GAUG - AUG C .-UG G UUGUCCC A --- - .-CU --- -| UG**

**AGUGG UUG GUUGGCG GUGUGCA CUAUUGC GUU UGA UGUCGG GUC AGA CUACAUCGGC GACCCA AC UUCUGC G**

**UCGCC AAC cagccgc uacaugu GGUAACG CAA ACU ACAGUC CAG UCU GAUGUGGUCG UUGGGU UG GGGGCG A**

**G ACA- U ag- A \ -- - UUUUAU- - GUU C \ -- UCC U^ UC**

**zma-miRgs41a GUUUUGUACUGCUACAGUAGCU**

**CAACAG AG AAG A UC ACA--- AG .-A | UC**

**CAC GC GC GUGGUAGUAUAG CA AAUGG C GAUGG--UGGGG A**

**GUG cg ug caucgucauguu gU UUGCC G CUACC ACUCU U**

**CAAUAG Cu a-- a uu GGAAGG CU \ - \ ^ UG**

**zma-miRgs41b GUUUUGUACUGCUACAGUAGCU**

**------ GA g u ua- .-uC| ACUAUUUU**

**GUUG AGGU uuuug acugc caguagc GUGGACA \**

**CAAU UUCG GAAAC UGAUG GUCAUCG UACCUGU A**

**ACAACU UC - C UAA \ --^ CCGUUUCG**

**zma-miRgs42 UGCACACGAGAUGGACUGGCC**

**A GAA--- u- .-GA -| C**

**GAU gguccguuu cugg GCC UGG U**

**CUG CCGGGCAAG GACC CGG GCU G**

**A AUCAAG UU \ -- U^ G**

**zma-miRgs43 UGGAUGUGAGAGGUUGCAAAG**

**AAA- AG U - C CU AC- C --- C UCAAA -| AACUUA**

**GCAC CUUUGUAG CC CUC UGUCCACUAU UG GCC AC GAUAA AGA AC UUAC A**

**UGUG gaaacguu gg gag guagguGGUG AC CGG UG UUAUU UCU UG AAUG C**

**UUUG AA - a u U- GUA A UAU U UUUUG U^ GUUUUU**

**zma-miRgs44 UCAGGUGCGGCUUCGACGAUU**

**U------- GGG AGAA- UUG**

**GUCGGAGCCGUA GGAGG AU C**

**cagcuucggcgu cuUCU UA U**

**AGAUuuag gga GAAGG UUA**

**zma-miRgs45a UUGGAGGGGAUUGAGGGGGCUA**

**- -| U A g AAUAGGA**

**GA CAA GG Auuggaggggauugaggg gcuaG \**

**CU GUU CC UAACUUCCCCUAACUCCC CGAUC A**

**C C^ C C A CUAGGGG**

**zma-miRgs45b UUGGAGGGGAUUGAGGGGGCUA**

**-| gg AU G AAUU**

**AGCACGGGGAuuggaggggauuga gggcuaAA AUCCCCUUU CUA \**

**UCGUGCCCCUAACCUCCCCUAACU CCCGAUUU UAGGGGAAA GAU U**

**C^ AA -- - AAGU**

**zma-miRgs45c UUGGAGGGGAUUGAGGGGGCUA**

**-| AU G AAUU**

**AGCACGGGGAuuggaggggauugagggggcuaAA AUCCCCUUU CUA \**

**UCGUGCCCCUAACCUCCCCUAACUCCCCCGAUUU UAGGGGAAA GAU U**

**C^ -- - AAGU**

**zma-miRgs45d UUGGAGGGGAUUGAGGGGGCUA**

**- g a -| AA**

**AGCAAGGGGAuug agggg uuga gggggcuaGAGU \**

**UCGUUUCCCUAAC UCCCC AACU CCCCCGAUCUUA G**

**C A C C^ GG**

**zma-miRgs46 AGGUGUUUCCUGUAGUGAUA**

**A-- A A CG U U UU----- .-UUUUC UU**

**CACUACAGGAAACGC UAAA UUUCGUGGGCC GA AUUC CG GCCUA GUCGAA U**

**gugauguccuuugug aUUU AAAGCACCCGG UU UGGG GC CGGAU CGGCUU A**

**aua g A UU - U UUUUAUU \ ----- UU**

**zma-miRgs47 GUGUCGCACUCGGCAAAUAG**

**GGUAC G GA AU C A**

**UUUGC GAGU GUGA CACU GAUAAA \**

**aaacg cuca cgcu gugA CUAUUU C**

**AGgau g -- -- U A**

**zma-miRgs48a AUAGGGGAUCUGCUGGAGAUG**

**U AGAUGGUUU U UG G-- AAA .-GAGGGAC| G U GA**

**CAUUUA AGGC GUCUUCGG GAUC UGU AUC GU GGAC GUA \**

**GUGAAU UCCg uagagguc cuag aua UAG CA UUUG CAU U**

**C --------- - gu ggg GAG \ -------^ G U AG**

**zma-miRgs48b AUAGGGGAUCUGCUGGAGAUG**

**CU CUA A UG A .-AA| GGAAU**

**GG AG CUGUCUCCAG GAUC CCUA AUAG \**

**CC UC Gguagagguc cuag ggau UGUC G**

**UG AA- C gu g \ --^ AAGGC**

**zma-miRgs49 GCAGGGACUGGGAAAGGGGGAU**

**AUUCUU----- U UU CUCU**

**UCCCUUUUUCC C UCUCU U**

**agggggaaagg g aggga A**

**GAAAACCCCAu - uc cgCC**

**zma-miRgs50a UCGCGGGGGGACGGUGCUCGCC**

**UA|GU C UC CAGG G U UG CG UCCA**

**G ACGAGGGCGA G CUG CCCUCCGCGA C CGC G UCG C**

**C UGCUUccgcu c ggc ggggggcgcu G GCG U GGC U**

**AG^UC - gu a--- - U GU AG UCGU**

**zma-miRgs50b UCGCGGGGGGACGGUGCUCGCC**

**CA|GU C UC CAAG G UG CG UCCA**

**G ACGAGGGCGA G CUG CUCUCCGCGA CGCGC G UCG C**

**C UGCUUccgcu c ggc ggggggcgcu GUGCG U GGC U**

**AA^UC - gu a--- - GU AG UCGU**

**zma-miRgs50c UCGCGGGGGGACGGUGCUCGCC**

**UA|GU C UC CAGG G UG CG UCCA**

**G ACGAGGGCGA G CUG CCCUCCGCGA CGCGC G UCG C**

**C UGCUUccgcu c ggc ggggggcgcu GUGCG U AGC U**

**AA^UC - gu a--- - GU AG UCGU**

**zma-miRgs50d UCGCGGGGGGACGGUGCUCGCC**

**GA UG G - -| g cu ccU - A**

**U GCGU ucg cgggggg acg ug cg UCG UCUCG A**

**G CGCG GGC GCCUUCC UGC GC GC AGC GGAGC C**

**GG GU G U G^ G CU --- U G**

**zma-miRgs51 GAUCUAGACGGAGUAUGUAGCAU**

**UCCGGGU .-AUAG UG-| AG**

**UUUGAUCU AAUCUU GAC G**

**AAAUUAGA UUAGAA UUG A**

**C------ \ ---- UCG^ CU**

**0**

**--- UG - C- CC- A .-UU AU .-UAAAAAGAAAAAUU UG**

**GCUAU ACUU GUUUG UUUGUAC UUA GCGCC GAU GUUUCUAUAGA CC U**

**cgaug ugag cagau agACGUG AAU CGUGG CUG UAAAGAUGUUU GG U**

**Aua ua g cu UAA C \ -- -- \ -------------- UU**

**zma-miRgs52a AGGUCAAAUGCUUGAACUAGGG**

**-| U A aa a-- gUAGA**

**UGGC AGU Uagguca ugcuuga cuagg \**

**AUCG UCA AUUCAGU AUGGACU GAUCU A**

**A^ U - A- CAA GAAGA**

**zma-miRgs52b AGGUCAAAUGCUUGAACUAGGG**

**-| U AG aa a-- AG**

**UGGC AGU agguca ugcuuga cuagggU A**

**AUCG UCA UUCAGU AUGGACU GAUCUCA A**

**A^ U A- A- CAA AG**

**zma-miRgs52c AGGUCAAAUGCUUGAACUAGGG**

**UA A-- C -- GA- AU ------ AG .-AG .-GU| CA G UU**

**UCU UGCUCU GGUU GGCGU ACCUG CUGGU CUUA AACCC AUGGCGA UGACGUAUG AG G \**

**AGA AUggga ucaa ucgua uggaU GAUCG GAAU UUGGG UGCCGCU GCUGUAUAC UC U A**

**CA AAG - gu aac AU GUUAAA AG \ -- \ --^ A- G GA**

**zma-miRgs52d AGGUCAAAUGCUUGAACUAGGG**

**-| U A aa a-- AGA**

**UGGC AGU Uagguca ugcuuga cuagggU \**

**AUCG UCA AUUCAGU AUGGACU GAUCUCA A**

**A^ U - A- CAA AGA**

**zma-miRgs52e AGGUCAAAUGCUUGAACUAGGG**

**UA G-- C -- GA- AU ------ AG .-C GC- .-U| CA G UU**

**UCU UGCUCU GGUU GGCGU ACCUG CUAGU CUUA AACCC UAUG GAG UGACAUAUG AG G \**

**AGA AUggga ucaa ucgua uggaU GAUCG GAAU UUGGG GUAC CUC ACUGUAUAC UC U A**

**CA AAG - gu aac AU GUUAAA AG \ - AGA \ -^ A- G GA**

**zma-miRgs53a UUUGGAUGCACUAGAGCUAAU**

**A .-GG c g U G ACU U AA- .-AACAAA| CUA**

**CAGGG UGuuuggaugca ua agcuaauAGUUA GU GCUAAAA GCUAG GG UUAGAUGGUU UAGCUAG \**

**GUCUU ACAAACUUACGU GU UCGAUUAUCAAU CG CGAUUUU CGAUU CU GGUUUGCCAG AUCGAUU A**

**A \ -- A A - A AAU U GUA \ ------^ AUC**

**zma-miRgs53b UUUGGAUGCACUAGAGCUAAU**

**AA G CU- .-UUA| CAUCCAAACAGUC UU**

**GGGGGGUGuuuggaugcacuagagcuaauA UUAG GCUAAAA GCUGAAGG UAGCUAAUAG \**

**UCCCCCACAAACUUACGUGAUCUCGAUUAU AAUC CGAUUUU CGAUUUUC AUCGAUUAUC C**

**AA A AAC \ ---^ ------------- AA**

**zma-miRgs53c UUUGGAUGCACUAGAGCUAAU**

**UU C A | AGUU CAA AC**

**AGGCC UGuuuggaugcacuagagcuaauAGUU GCUG--GCUGAAA GCUAGUA UUAGCUGGCUA \**

**UCCGG ACAAACUUAUGUGAUCUCGAUUAUCAA CGAU CGACUUU UGAUUAU AAUCGAUCGAU A**

**AU U C \ ^ AAU- C-- AA**

**zma-miRgs54 UAUCAGCUGAGAAUUAGGCGUC**

**.-UGAUGGGGGU u- c- .-UGAG .-UAAU| CCCGA**

**uaucagcugagaa uagg gucU GGUACCCC UAUGGUC C**

**AUAGUUGAUUUUU AUCC CAGA UCAUGGGG AUAUUAG A**

**\ ---------- UU AA \ ---- \ ----^ UCAUG**

**zma-miRgs55 ACGAUCUAAUGGACCAGAUGG**

**C AC C .-ACA ACAA G UU AAA-| U**

**UGC AGCAAACCA CUGGUCCAUUAGAUU GAUC AACC GAAU CCAU AU GCU GGUUUUU U**

**ACG UCGUUUggu gaccagguaaucuag UUAG UUGG UUUG GGUG UG CGA UUAGAAA A**

**- U a ca A \ --- GGAA G GU GUUA^ U**

**zma-miRgs56 UGGCAGUACUUGCUGAAACGGU**

**CU AG .-aa ucaugug GG .-A| AUG**

**G GGugg ugucguag GAGCUU UCUUCGUC GGCGC A**

**C CCACC ACAGCGUC UUCGAA AGAAGUAG CCGCG G**

**UG CA \ -- ------- -- \ -^ CUC**

**zma-miRgs57 UUGAUUGGAGCUUCUUCGGAC**

**G-- A---| C U GA U- CAC-- AUA .-CUCU UUUCCU**

**GACA UCCG AA GAAGU CCGGUU GGC AUUC GCAUGCC UC \**

**UUGU aggc uu cuucg gguuag UUG UGAG UGUACGG AG U**

**UUG GCUc^ - - a- uu UUCUC --- \ ---- UUUGUU**

**zma-miRgs58 AGGUUGAUUGGUUCGAUUGAU**

**A| g CAU C A**

**UACCAAUCCAGAaggggauuggag ggauu UCC CUCCUAGUCA A**

**GUGGUUAGGUCUUCCCCUAACCUC CCUAA AGG GAGGAUCAGU A**

**G^ G AUU A A**

**zma-miRgs59a AGAAGGGGAUUGGAGGGGAUU**

**A| g CAU C A**

**UACCAAUCCagaaggggauuggag ggauu UCC CUCCUAGUCA A**

**GUGGUUAGGUCUUCCCCUAACCUC CCUAA AGG GAGGAUCAGU A**

**G^ G AUU A A**

**zma-miRgs59b AGAAGGGGAUUGGAGGGGAUU**

**U A - | A**

**AGGAGAUUUAAUCC CCU CAAUCCU--UAUG A**

**UCCUCUAAAuuagg gga guuaggg gUAC G**

**C - g \ ^ A**

**zma-miRgs59c AGAAGGGGAUUGGAGGGGAUU**

**-| a AAUA**

**ACACCAAUCCagaaggggauuggagggg uuAAAUCC \**

**UGUGGUUAGGUCUUCCCCUAACCUCCCC AAUUUAGG U**

**A^ A AGAA**

**zma-miRgs59d AGAAGGGGAUUGGAGGGGAUU**

**AC| G A**

**ACCAAUCCagaaggggauuggaggggauuUAAUCCCCU CUAGUCA A**

**UGGUUAGGUCUUCCCCUAAUCUCCCCUAAGUUAGGGGA GAUCAGU A**

**UA^ G A**

**zma-miRgs60a UUGAGCCGCGUCAAUAUCUCC**

**G U C--| C AG A G**

**ACAA GC GGGAGGUAUUGGCGUG CUCAAUCCGA GCGUGGCUG CUG \**

**UGUU CG Cccucuauaacugcgc gaguuAGGUU UGUACCGAC GGC C**

**- - UUC^ c CU - G**

**zma-miRgs60b UUGAGCCGCGUCAAUAUCUCC**

**GGAGGUAUUGGCGCGCCUCAAUC**

**C| A G Cg c AG AUAGAU**

**UAGG CAA GG gagguauuggcgcg cucaaucCGA GCGUGGCUG U**

**GUCC GUU Cc cucuauaacugcgc gaguuAGGUU UGUACCGAC G**

**U^ - G -- c CU GGCGCG**

**zma-miRgs61a AGGAAUCCCUUGGGAAAAUG**

**U| CC C C UC UC A UUAG**

**UUGGGAAC CGUUU CCCA GGGAUUU AUUUU CA GGGAAA \**

**AACCCUUG guaaa gggu cccuaag UAAAA GU CCCUUU U**

**-^ AU a u ga GA A UACU**

**zma-miRgs61b AGGAAUCCCUUGGGAAAAUG**

**U| CC U U UU UUAG**

**UUGGGAAC CAUUUUCCCAAG GAUUUCUA UUUCCC AGGAAA U**

**AACCUUUG guaaaaggguuc cuaaggaU AAAGGG UCCUUU U**

**-^ AU c U U- UUAC**

**zma-miRgs62 UGUAAGUGUGAUUGUGCACCAA**

**- CAC - u uu ac .-ACUAC -| A**

**UAG UGUAug uaagug ga gugc caaC ACU AGA C**

**AUC GCGUAC GUUCAU CU CAUG GUUG UGG UCU U**

**A CA- U C -- AU \ ----- U^ C**

**zma-miRgs63 GGUCUGUUUGGUUGGGCUAU**

**AUUAAUUU c g a- .-AAAAAAG UU - -- - AAA-- G GUU .-CUAUG| GUU**

**AGggu uguuugguu ggcu uGGCUUUG UUG GUGG GCUGUGA GC UAUGU AA UU GUGGU AGCU \**

**UCCCA ACAAACCAA CCGA ACCGAAAC AGC UACC UGACACU UG AUAUA UU AA UACCG UCGA A**

**GAACCUAU C A AA \ ------- C- U UU U GCUUC G AAU \ -----^ AAA**

**zma-miRgs64a AGAAUCAGGGAUGGAACGGUU**

**CCGCUCUGUUCCAGUUCACU**

**CAA c a- .-ac| CAA**

**AGGAUGGAGccg ucuguucc guuc uUUG C**

**UCUUGCCuuggc agguaggg uaag aAAC C**

**UGA a ac \ --^ AAA**

**zma-miRgs64b AGAAUCAGGGAUGGAACGGUU**

**- CC aa au c - C- CUU .-CUCAACCAAACAC| AAGAGAAU**

**CUCCGUU Uag ucaggg ggaa ggu uC GUUAUA GGCUC UACCU C**

**GAGGUAA GUC AGUUCC UCUU CCG AG CAAUGU UUGAG AUGGA C**

**G AU G- CC - C AA UCU \ -------------^ AAUGUACC**

**zma-miRgs64c AGAAUCAGGGAUGGAACGGUU**

**- CC aa au c - C- CUU .-CUCAACCAAACAC| AAGAGAAU**

**CUCCGUU Uag ucaggg ggaa ggu uC GUUAUA GGCUC UACCU C**

**GAGGUAA GUC AGUUCC UCUU CCG AG CAAUGU UUGAG AUGGA C**

**G AU G- CC - C AA UCU \ -------------^ AAUGUACC**

**zma-miRgs65 AGCAAGAGGAUUGGAGGGGCU**

**CA| a U**

**UGUUUGGGagcaag ggauuggaggggcuAAAAUCACCUUGCUAU C**

**ACAAACCCUCGUUU CCUAACCUCCCCGAUUUUAGUGGAACGAUA A**

**GA^ C A**

**zma-miRgs66a UAGGGUUUUGUGGUCGAGUGAU**

**A- CUUCC C G CUAGA-- UG - UC**

**GCU AAUCACUC GA CGC UGGUCGG C C \**

**CGA Uuagugag cu gug auUAGCC G G A**

**UG ----- - g uuuuggg GU A AC**

**zma-miRgs66b UAGGGUUUUGUGGUCGAGUGAU**

**C A A- GGAU UC - - CA GAGGU- A-| GAG UCCCGUUCC G - U AG**

**CCGACGU GAUCG CGAU CGG CUCUAG UGGCAC CUG GGGG UUGC GGGC GCUCGGA UGAU GGGAGA UGGUGA CUG \**

**GGUUGCG Uuagu gcug guu gggauC GCUGUG GAC CUCC AAUG UCCG CGAGCCU ACUG CCUUCU ACCGCU GAU A**

**C A ga gu-- uu G A A- AUGGUU GA^ --- C-------- G C - AC**

**zma-miRgs67 UACGGGGAGGCUACGCUAGCGA**

**ACCAAGAACAC GC A G - A .-AUGUUUCAGGUCC| A CUC**

**CGC GGUGUG CCUC CCGUGG CC AC GUUC UGUC U**

**gcg ucgcau ggag ggcauU GG UG CAAG ACGG G**

**UAAAACAAAAa a- c g A A \ -------------^ - UGU**

**zma-miRgs66c UAGGGUUUUGUGGUCGAGUGAU**

**A G G G CU U- A .-CAAG G .-U -| G**

**UCGGUG U GAUCA CUC CCAU GGC CCUA GGA GCUCAGA CCCGU UCCC A**

**AGUUAC A Uuagu gag ggug uug ggau UCU CGAGUCU GGGUA AGGG U**

**C G - - cu uu - \ ---- G \ - G^ G**

**zma-miRgs68 GACAUGAAUCGGUGGAUCCCU**

**UUC - - G - a c UG--- CGC-- .-G| GA**

**GA CU A Cgac auga ucgguggaucc uCGA GUGUA AAGUGGU GCG A**

**CU GA U GCUG UGCU AGUUACUUAGG AGUU CACAU UUCACUA CGC A**

**ACU A G G U C C UCUCA AGAUA \ -^ GA**

**zma-miRgs69a GUCGCCCCUGGACGGUCGCGGA**

**GUU .-A cc-- -| ga CG**

**CUAGGG gucgc cuggacggu cgcg CCAUC \**

**GAUCUC CGGUG GGCCUGCCA GCGC GGUGG C**

**GC- \ - UCCA G^ G- AC**

**zma-miRgs69b GUCGCCCCUGGACGGUCGCGGA**

**- UG A ccc ga - a-| G**

**GU UAGAG gucg cug cg gucgcgg CC U**

**CG AUCUC CGGC GAU GC CGGUGCC GG U**

**G CG C CU- AG G CA^ C**

**zma-miRgs70a UUCUUAGCACUUGAAGCACGCC**

**C C U - - cuu .-gccAUUUGAU| AUU**

**CGC G UAUuucu uag ca gaagcac GUGGA U**

**GUG C AUAAAGA GUC GU CUUCGUG CGCCU U**

**U U - U U CCU \ ----------^ AAA**

**zma-miRgs70b UUCUUAGCACUUGAAGCACGCC**

**C C U - - cuu .-gccAUUUGAU| AUU**

**CGC G UAUuucu uag ca gaagcac GUGGA U**

**GUG C AUAAAGA GUC GU CUUCGUG CGCCU U**

**U U - U U CCU \ ----------^ AAA**

**zma-miRgs70c UUCUUAGCACUUGAAGCACGCC**

**C C U - - cuu .-gccAUUUGAU| AUU**

**CGC G UAUuucu uag ca gaagcac GUGGA U**

**GUG C AUAAAGA GUC GU CUUCGUG CGCCU U**

**U U - U U CCU \ ----------^ AAA**

**zma-miRgs71 UGAAGGAAGUGGGAUCCUCAAAU**

**UAUGA C UGGG - - - .-AA| C**

**UG CU GAGGAU UCAC UCC UCA UGA A**

**AC GA cuccua ggug agg agu ACU A**

**UAAAA A uaaa g a a \ --^ G**

**zma-miRgs72a CUGGUGAGCGCGGACCGUCCGG**

**- C UC-| uga g c ggA U**

**GAC GUCC cugg gc cggac gucc CC \**

**CUG CAGG GAUC CG GCCUG CAGG GG A**

**C A UGU^ CCC - A AGC U**

**zma-miRgs72b CUGGUGAGCGCGGACCGUCCGG**

**- C UC-| uga g c ggA U**

**GAC GUCC cugg gc cggac gucc CC \**

**CUG CAGG GAUC CG GCCUG CAGG GG A**

**C A UGU^ CCC - A AGC U**

**zma-miRgs73a UGGCUAUGUGUUUACUGCGGC**

**A A-- - G C .-AUUGA| UA G**

**UCGAG GGCU UGGUAAA AUAU GGCUA UCUU GU U**

**AGCUU Ucgg gucauuu ugua ucggu AGAA UA U**

**C GUA c g - \ -----^ UG A**

**zma-miRgs73b UGGCUAUGUGUUUACUGCGGC**

**UCAUCCA - G C AUUGUUC .-AU| GCAA**

**AUGGCU UGGUAAA AUAU GGCUA UUUGGUGUUA AUAU \**

**UAUcgg gucauuu ugua ucggu AAGCCAUAGU UAUA U**

**CAACUUG c g - ------- \ --^ GCUC**

**zma-miRgs74a AAUAAAAAGAAACGGAUGGAG**

**CAAAUAU| U CAA**

**GUACUUC UCCGUUUCUUUUUAUUUGUCGCUGGAUAGUG U**

**UAUgagg aggcaaagaaaaauaaACAGCGACCUAUCAC U**

**GACGACC^ u AUU**

**zma-miRgs74b AAUAAAAAGAAACGGAUGGAG**

**AAAAAAAU| C A**

**UACUCC UCCGUUUCUUUUUAUUUGUCGCUGGAUAGUGUA A**

**AUgagg aggcaaagaaaaauaaACAGCGACCUAUCACGU A**

**UUUUAUUC^ u U**

**zma-miRgs74c AAUAAAAAGAAACGGAUGGAG**

**U| GUCAA C C AA A CGU ACAA**

**UUG UACUCC UCCGUU CUU UAU UGUCGUC UAGU A**

**AAC AUgagg aggcaa gaa aua ACAGCAG AUCA U**

**-^ AAUAC u a aa a CUC AAUU**

**zma-miRgs74d AAUAAAAAGAAACGGAUGGAG**

**G| CUCCAAACUGA C CAA**

**CUGCAUG UACUCC UCCGUUUCUUUUUAUUUGUCGCUGGAUAGUG A**

**GACGUAC AUgagg aggcaaagaaaaauaaACAGCGACCUAUCAC A**

**A^ ----------- u AUU**

**zma-miRgs74e AAUAAAAAGAAACGGAUGGAG**

**UGGUA -| C A**

**UG UACUCC UCCGUUUCUUUUUAUUUGUCGCUGGAUAGUGUA A**

**AC AUgagg aggcaaagaaaaauaaACAGCGACCUAUCACGU A**

**AUAAA G^ u U**

**zma-miRgs74f AAUAAAAAGAAACGGAUGGAG**

**UAUAUAUA| C UAA**

**UACUCC UCCGUUUCUUUUUAUUUGUCGCUGGAUAGUG A**

**AUgagg aggcaaagaaaaauaaACAGCGACCUAUCAU A**

**AUCCUACC^ u GUU**

**zma-miRgs75 UACUGGGUGACAAAUGCAGUCU**

**U| G UGAA GGG G A- - UUAC A**

**GA GUUCA AAAGGC UGCGUUUGUCA UCCG ACG GCA UGUG A**

**CU UAGGU UUucug acguaaacagu gggu uGC CGU AUAC U**

**-^ G ---- --- - ca U U--- U**

**zma-miRgs76 GCGACUUCGCCGGUCUACUA**

**AC A ACU C .-UU GU AU -| C**

**UGGCG AGUGG GGCUGGUG GGUCGUUUGGCG G GG UCGGCU UGCGC G**

**AUCGC UCauc cuggccgc ucagcgAGCUGC C CU AGUCGG GCGCG U**

**AC C au- u \ -- UG CC U^ U**

**zma-miRgs77 UAAGACUAGCAUGGCCUGGCUG**

**UCUG U AA A- A CA UU G - C-- AAA AU UUGA**

**UCU UAG AGG GUG UGGUU UUAGC UUAAG GUGUA CUAGA GUC CUCU UUAG \**

**AGG guc ucc uac aucag aauCG AAUUC UACGU GAUCU CGG GAGG AAUC U**

**CUA- U gg gg g -- CU G A UAU AG- -- UUUU**

**zma-miRgs78a UAAAGGGAUUCUCUCAAGUUGA**

**A| U C ga c gaAG**

**GACU GU Guaaagg uucucu aaguu \**

**UUGG CA CGUUUCC GAGAGG UUUAG G**

**G^ C - A- U AUUC**

**zma-miRgs78b UAAAGGGAUUCUCUCAAGUUGA**

**A| U U ga c gaAG**

**GACU GU Guaaagg uucucu aaguu \**

**UUGG CA CGUUUCC GAGAGG UUUAG G**

**G^ C - A- U AUUC**

**zma-miRgs78c UAAAGGGAUUCUCUCAAGUUGA**

**A| U U ga c gaAG**

**GACU GU Guaaagg uucucu aaguu \**

**UUGG CA CGUUUCC GAGAGG UUUAG G**

**G^ U - A- U AUUC**

**zma-miRgs78d UAAAGGGAUUCUCUCAAGUUGA**

**A| U U ga c gaAG**

**GACU GU Guaaagg uucucu aaguu \**

**UUGG CA CGUUUCC GAGAGG UUUAG G**

**G^ U - A- U AUUC**

**zma-miRgs79a AAGGGUCAAGUUGUAAGACAUC**

**- C .-Aaa - - - u .-CCUAAAACUC| UC**

**GUUAGU UC gg g ucaaguugua agaca cUCCC UGCA A**

**CAAUUA AG CC C AGUUCAACAU UUUGU GAGGG ACGU C**

**A - \ --- A G G - \ ----------^ UU**

**zma-miRgs79b AAGGGUCAAGUUGUAAGACAUC**

**- C .-Aaa - - - u .-CCUAAAACUG| UC**

**GUUAGU UC gg g ucaaguugua agaca cUCCC UGCA A**

**CAAUUA AG CC C AGUUCAACAU UUUGU GAGGG ACGU C**

**A - \ --- A G G - \ ----------^ UU**

**zma-miRgs79c AAGGGUCAAGUUGUAAGACAUC**

**- C .-Aaa - - - u .-CCUAAAACUG| UC**

**GUUAGU UC gg g ucaaguugua agaca cUCCC UGCA A**

**CAAUUA AG CC C AGUUCAACAU UUUGU GAGGG ACGU C**

**A - \ --- A G G - \ ----------^ UU**

**zma-miRgs80 AUGCAUUCUUGACGUCGAGCUG**

**C G Aaug u a .-gGUGAACA| A GAC**

**CGU GAGG ca ucuug cgucgagcu GGCC UU \**

**GCG CUUU GU AGGGC GCAGUUCGG CCGG AG G**

**A G GAAA C A \ --------^ G AGG**

**zma-miRgs81a AAACACAAGGGCAUAUAGAGAC**

**- UGAU--| ggg u A A**

**GUUA UCaaacacaa caua agagacUUCAAC AAUCC \**

**CAAU AGUUUGUGUU GUGU UCUUUGGAGUUG UUAGG C**

**G CAGAGU^ ACA - A U**

**zma-miRgs81b AAACACAAGGGCAUAUAGAGAC**

**- GAC-----| ggg u A A**

**GUUAU UCaaacacaa caua agagacUUCAAC AGUCC \**

**CGGUA AGUUUGUGUU GUGU UCUUUGAAGUUG UUAGG G**

**U AUCAGAGU^ ACA - A U**

**zma-miRgs81c AAACACAAGGGCAUAUAGAGAC**

**GAUAUGAC| ggg u A U**

**UCaaacacaa caua agagacUUUAAC AGUCC \**

**AGUUUGUGUU GUGU UCUUUGAAGUUG UCAGG C**

**AUCAGAGU^ ACA - A C**

**zma-miRgs81d AAACACAAGGGCAUAUAGAGAC**

**GAUAUGAC| ggg u U**

**UCaaacacaa caua agagacUUUAACAAGUCC \**

**AGUUUGUGUU GUGU UCUUUGAAGUUGUUCAGG C**

**AUCAGAGU^ ACA - A**

**zma-miRgs81e AAACACAAGGGCAUAUAGAGAC**

**GAUAUGAC| ggg u U**

**UCaaacacaa caua agagacUUUAACAAGUCC \**

**AGUUUGUGUU GUGU UCUUUGAAGUUGUUCAGG C**

**AUCAGAGU^ GCA - A**

**zma-miRgs81f AAACACAAGGGCAUAUAGAGAC**

**- UGAC--| ggg u U U**

**GUUA UCaaacacaa caua agagacUUCAAC AGUCC \**

**CAAU AGUUUGUGUU GUGU UCUUUGAAGUUG UCAGG C**

**G CAAAGC^ GCA - U A**

**zma-miRgs81g AAACACAAGGGCAUAUAGAGAC**

**UUCUUGUGACAUUGUGUUUGA**

**-| A AC ggg u C A A**

**GUU UG UCaaacacaa caua agagacUU GAC AAUCC \**

**CAG GC aguuuguguu gugu ucuuUGAA UUG UUAGG C**

**U^ A -- aca - U A U**

**zma-miRgs82 UGGGGCUCUUCGGAUCUGGC**

**C A - u au .-ACU| G C GA**

**UUGCGCUG ugg ggc cuucgg cuggcGU UCUC AA AGCU \**

**GAUGCGGU GUC CCG GAAGCC GACCGCA AGAG UU UCGA U**

**U A A - C- \ ---^ G C GA**

**zma-miRgs83a UGAAGACUAAACGACGAGACA**

**AUUC .-c cg .-aAAU| A**

**CAUAGAugaagacuaaa ga agac UCAUUA G**

**GUGUCUAUUUCUGAUUU CU UCUG AGUAAU C**

**UAAU \ - AA \ ----^ C**

**zma-miRgs83b UGAAGACUAAACGACGAGACA**

**AUA| a cg AAA C UUAAA CG AAGAA UACUU**

**ACAUAGAuga gacuaaacga agaC UUUAUUAAG CUAA UCCA AUU AUGUG \**

**UGUGUCUAUU CUGAUUUGCU UCUG AGAUAAUUC GAUU GGGU UAA UACAC U**

**UAA^ C AU CUC A C---- AA GCGAG CACGA**

**zma-miRgs83c UGAAGACUAAACGACGAGACA**

**AA a c a UC C .-UAA| AUA**

**UACAUAGAug aga uaaacgacgag caAA UAUUAAG CUAAC UGC \**

**AUGUGUCUAU UCU AUUUGUUGUUC GUUU AUAAUUU GAUUG ACG A**

**UA A A A UA C \ ---^ AUU**

**zma-miRgs83d UGAAGACUAAACGACGAGACA**

**AC U a ga - C--- A AACA-----| GUG**

**UAUAUA Auga gacuaaacgac gaca AAUUUA UAAGUCUAAUUA UCCAUGAUU AAUGC U**

**AUGUGU UGCU CUGAUUUGCUG UUGU UUAGAU AUUCGGAUUAAU AGGUACUAA UUACG U**

**UA C G -- A AAUU C ACGGCUAUA^ AUG**

**zma-miRgs83e UGAAGACUAAACGACGAGACA**

**- A a ac caAA AUC -----| A**

**AUUACAU AAuga gacuaaacg gaga UUUAUUAAGCCUAAUUA CAUGAUU AAC A**

**UAAUGUG UUAUU CUGAUUUGC UUUU AGAUAAUUUGGAUUAAU GUACUAA UUG A**

**U G C CA ACUC CAA GCGAU^ U**

**zma-miRgs84a GGGGAUUGAAGAGGAUUAAA**

**- ----------| a A A**

**UCGGUUGCUA ggggauugaag ggauuaaaUCCCC CCUAUUCA U**

**AGCCAAUGAU CCCCUAACUUC CCUAAUUUAGGGG GGAUAAGU U**

**A CACCAAACUC^ C A U**

**zma-miRgs84b GGGGAUUGAAGAGGAUUAAA**

**-| a CG**

**CAAUCCAGAGggggauugaag ggauuaaa G**

**GUUAGGUCUCCCCCUAACUUC CCUAAUUU G**

**G^ C AG**

**zma-miRgs84c GGGGAUUGAAGAGGAUUAAA**

**- -| A GA g AA**

**GG GG UUG ggggauugaaga gauuaaaUCCCCUCCUAUUC \**

**CC CC AAC UCCCUAACUUCU CUAAUUUAGGGGAGGAUAAG A**

**A A^ A UC A UA**

**zma-miRgs84d GGGGAUUGAAGAGGAUUAAA**

**GGGGAUUGGA| a U A**

**ggggauugaag ggauuaaaUCCCCUCCUAU CA U**

**CCCCUAACUUC CCUAAUUUAGGGGAGGAUA GU U**

**C---------^ C C U**

**zma-miRgs84e GGGGAUUGAAGAGGAUUAAA**

**- .-GAUUG| ag a CCCUG U A**

**GGC GAggggauugaag g uuaaaUC CUA UCA A**

**CCG CUCCCCUAAUUUC C AAUUUAG GAU AGU U**

**U \ -----^ CU A AGAAA C A**

**zma-miRgs84f GGGGAUUGAAGAGGAUUAAA**

**CUA -----------| aga UCA GUCAA**

**GGUUGU Aggggauuga ggauuaaaUCCC CUA A**

**CCAACG UUCCCUAGCU CCUAAUUUAGGG GAU A**

**AAA CAGACCUAGCU^ CCC UAA AAAUU**

**zma-miRgs84g GGGGAUUGAAGAGGAUUAAA**

**C| GCA g - ga C U A**

**GGAU AG gggauugaa gag uuaaaUCCC UUCUA UCA A**

**CCUA UC CUCUAACUU CUC AAUUUAGGG AAGAU AGU U**

**A^ AC- A U A- A C U**

**zma-miRgs84h GGGGAUUGAAGAGGAUUAAA**

**GGGGAUUGGA| a C A**

**ggggauugaag ggauuaaaUCCC UCCUAUUCA U**

**CCCCUAACUUC CCUAAUUUAGGG AGGAUAAGU U**

**C---------^ C A U**

**zma-miRgs84i GGGGAUUGAAGAGGAUUAAA**

**UC| g a aga AU A**

**GGUUGCUAg gg uuga ggauuaaaUC CUUCUAUUCA U**

**CCAAUGAUC CC AACU CCUAAUUUAG GAGGAUAAGU U**

**AA^ A A CCC GG U**

**zma-miRgs84j GGGGAUUGAAGAGGAUUAAA**

**- ----------| a CC A**

**UCGGUUGCU Aggggauugaag ggauuaaaUC CUCCUAUUCA U**

**AGCCAAUGA UCCCCUAACUUC CCUAAUUUAG GAGGAUAAGU U**

**A UCACCAAACU^ C AA U**

**zma-miRgs85 AUAGCUAUCUGUUGGGCACU**

**GGAC - UC AC G CUUC -- ----- C------ .-CAAAACUU| CU AU C GUGAA UU CC U**

**AGUC CGGUGC CCAG CAGA GGC GGUUCC CA CUUUGCU CUUUGUUGAAUC GAU UUUU UGG UGAGU CCU UACA UG**

**UCGG gucacg gguu gucu ucg CCGAGG GU GAAACGG GAAAUAACUUGG UUA AAAA ACC ACUUA GGG GUGU AU**

**UGAU U -- -- a aua- UU AUAUA AAACACA \ --------^ UU CC A AC--- UU UU U**

**zma-miRgs86 AAGUUUGUGGACCUAAGUGGC**

**GU U A C CA---- A GCAC .-GUGGUC ------| C**

**CAA GGAUGCCACUUAGGUC ACAAACU UGAAAAUGUAUUUCUAGAUCCU AACUUGUUA GUG ACCACA GGUGUA GUCCA A**

**GUU CUUAcggugaauccag uguuuga AUUUUUACAUAAAGAUCUAGGA UUGAACAAU CAC UGGUGU CCACGU CAGGU A**

**CC C g a ACGAAC C AACA \ ------ AGUGUC^ U**

**zma-miRgs87 UUUGUAUUCAUUGAUCUCAGAA**

**AAUG A GG AGCG A .-UAGUCCAUUCUAA| U C CAA**

**GCC UCUU UUGA GGUGAGU GCAG AAAGA AAU UGA A**

**CGG AGaa gacu uuacuua uguu UUUCU UUA ACU A**

**CAAA A -- cuag - \ -------------^ C - ACA**

**zma-miRgs88 AUCUCACGAGAUUGGAACAGGG**

**UCAAUU C AC .-UUCCU C UAUAUGA**

**AGGCCC GUUUCAAUCUC GGGAUAAACUUUAGC GCUAAA UUUAGC \**

**UCCggg caagguuagag cucuaUUUGAAAUCG CGAUUU AAAUCG A**

**AU---- a ca \ ----- A UGAAGUU**

**zma-miRgs89 UCAGGUUAGAAACAGAGCAUG**

**C A---- A -| a a gca CCA U AGA AAGCCA G A AA--- U GA**

**AGC CC GCu cagguu ga acaga ugUUU CUG GG GCA CUGG UG UGC UG CC A**

**UCG GG CGA GUCCGA CU UGUCU ACGAA GAC CC CGU GACU AC ACG AC GG A**

**C CCAAC A G^ - C AAG AC- - --- AACCCC G G ACCCG - AA**

**zma-miRgs90 UCUGAUCCGAGACAGAGUUUC**

**- UU- A a GCAG CCCCGC --- -| G**

**CC CU UACucug uccgagacagaguuucG CACCU UGUUCUCCGGAU ACAC GUCCU G**

**GG GA AUGAGGC AGGCUCUGUCUCAAAGC GUGGA ACAAGAGGCCUG UGUG UAGGG C**

**A UAC G C AACA AGUACA UCA A^ A**

**zma-miRgs91 UCUUUGUGAUAGACUUCGGGUU**

**CUAC ---------- - ug u g uUUU-| A UGUU U UC**

**GCGGA Gucu uug a agacuucg gu UCGC CU GUGC AG \**

**CGCUU CAGA AGC U UCUGAGGC CA AGCG GA CACG UC C**

**UUCA UUUUAGGCUA U GU U G UCUUC^ - UUCC - GG**

**zma-miRgs92a GAUGAAGGGGGCAUGUAGACAU**

**.-AUAUGAGAU - UC AAC ---- UCA**

**AUGUCU UGUGCCCU UCAU GAA AUUGGG \**

**uacaga guacgggg agua CUU UAACUU C**

**\ --------- u ga gUA UUAC CAC**

**zma-miRgs92b GAUGAAGGGGGCAUGUAGACAU**

**AUAU C - A AUC U UGUCACC**

**GAG GAUGUCU UGU CCCUUUUCAU GAAA UG \**

**UUC Cuacaga gua gggggaagua CUUU AC A**

**---- C u c gUA U UAACUUC**

**zma-miRgs92c GAUGAAGGGGGCAUGUAGACAU**

**UAUAU A -| AUC U UGUCACC**

**GAG GAUGUCU UGUGCCUUUUUCAU GAAA UG \**

**UUC Cuacaga guacgggggaagua CUUU AC A**

**GAGAG C u^ gUA U UAACUUC**

**zma-miRgs93 AUUGAUUGUAAAAGGACCAG**

**.-UUUUCUUUU| uuguaaaa- - .-GCCA U**

**Uauuga ggac cag GUA--GGGG U**

**GUGACU CCUG GUC CAU CCCC U**

**\ ---------^ CUUGUAUUG U \ ---- \ C**

**zma-miRgs94 AAUUCAUAUGGAUUGGGGUA**

**U AU- UU GA .-UACGGAU| AAAAA**

**UGUUCGGUU U CAAUCCAUAUGGAUUG GGGGGUUGA UGAG \**

**ACAAGCCAA g guuagguauacuuaaC CCCCUAACU AUUC U**

**- Gau gg UC \ -------^ AGUUG**

**zma-miRgs95 UUUUGAGAUGAAACGAACAAGG**

**C --- - UG U .-G| AU**

**CUAC GGGCC UGUUCGUUU UUUCAAA CCAUGUGGAUUGAGGGG GGA \**

**GGUG UCCgg acaagcaaa agaguuu GGUACACUUAACUCUCC CCU A**

**- UCA a gu u \ -^ AA**

**zma-miRgs96 AGACUUUAGUGAGAUGGCUGU**

**GACA A ac- - a AAAG - UUG----| A**

**GAGG Uag uuua gugag uggcugu CAAU UC GC A**

**CUUU GUC AGGU CAUUC ACUGACG GUUA AG CG C**

**AGAA G AGU C - AGUA U UUAAAGA^ U**

**zma-miRgs97 GCGGCGCUGACCAUCUGAGUGG**

**A A -- U -| AG A CCUUCUUC UC**

**GUAG CUG GCC CU AGAUG CAGUGC UGC UCG \**

**CAUC GGC Ugg ga ucuac gucgcg gcg AGC U**

**- - GU u g^ ca - CCAGCAUC UC**

**zma-miRgs98a UAUCGGACCUAGAUGCGGUAAG**

**- A- U--- g- .-c| aa A UAAA- AAUCA**

**UG AACA GAuauc gaccuagaug ggu gAU CAA CUACC \**

**AC UUGU CUGUAG CUGGAUCUAC CCA UUA GUU GAUGG U**

**U GG UUAC AG \ -^ G- - UGAGA CAAGA**

**zma-miRgs98b UAUCGGACCUAGAUGCGGUAAG**

**CUA U--- g- c .-uaagAUA UAAA-| AAUCA**

**AACA GAuauc gaccuagaug gg CAA CUACC \**

**UUGU CUGUAG CUGGAUCUAC CC GUU GAUGG U**

**CGG UUAC AG U \ ------- UGAGA^ CAAGA**

**zma-miRgs98c UAUCGGACCUAGAUGCGGUAAG**

**CUA U--- g- c .-uaagAUA UAAA-| AAUCA**

**AACA GAuauc gaccuagaug gg CAA CUACC \**

**UUGU CUGUAG CUGGAUCUAC CC GUU GAUGG A**

**CGG UUAC AG U \ ------- UGAGA^ CAAGG**

**zma-miRgs99a UUUGAAUGCACUAGAGCUGAU**

**UAGCUCUAGUGCAUUCAAACA**

**UAA - - G A- ----- | G**

**GAGG G UGuuugaaugcacuagagcugauA UUAGU GCUAAA AUUAGU--UGGA A**

**UUCC C acaaacuuacgugaucucgauUAU AAUCG CGAUUU UGAUCA ACCU C**

**UGA A A A AC UUUAA \ ^ A**

**zma-miRgs99b UUUGAAUGCACUAGAGCUGAU**

**UAAGC a c G .-GGCUAAAAAAUU .-AAAUUA| AC**

**GGGUGuuugaaugcacu gag ugauA UUAGUU GCUAGUA GCUAGCUA \**

**CCCACAAACUUACGUGA CUC AUUAU AAUCGA CGAUUAU CGAUCGAU A**

**GAAUU C C G \ ------------ \ ------^ AA**

**zma-miRgs99c UUUGAAUGCACUAGAGCUGAU**

**UAAAGA c GUUG .-AA .-AAUUA| AC**

**GGUGuuugaaugcacuagag ugauAGUUA GCUAAAA UAGCUAGUAA GCUGGCUA \**

**CCACAAAUUUACGUGAUCUC AUUAUCAAU CGAUUUU AUCGAUUAUU CGAUCGAU A**

**AAUCCC U AGA- \ -- \ -----^ AA**

**zma-miRgs99d UUUGAAUGCACUAGAGCUGAU**

**A AC-- AUA -| UA**

**GCUC UAUUAGCUCUA CAUUCAAACACUUC CU A**

**CGGG Auagucgagau guaaguuuGUGAGG GA U**

**A GAUC cac U^ UU**

**zma-miRgs100 GAGGACCCUCGGAAGCCGAA**

**- A UUU a - a g- .-CCAA| GUU**

**UC UG UUgagg ccc ucgg agcc aaGGCCC CA \**

**AG AC GGCUCC GGG AGUC UUGG UUUCGGG GU G**

**A - UGU - U A GA \ ----^ AAC**

**zma-miRgs101 UCUGGGCAGAGGCAGUCAACAC**

**AAGAACACA AGAG UAA GA .-GGA AAC U C CCUAUAGC AA**

**GGUGUUGA UUCU GC AGAAG UCA AUGAGU UU GGUUC CCACAACA A**

**Ccacaacu gaga cg ucuUC AGU UAUUCA AA CCAAG GGUGUUGU U**

**CCG------ gacg --- gg \ --- --- C A AAAGAGA- GG**

**zma-miRgs102 UGAAGGGGAUUGAGGGGGCUA**

**AUUGAU - A C G AU UA-- U--- --- UUU- .-U| C**

**AUUCU GCUCCC UC AU UCCUUCGU--UAAUAA GC ACA GAUUCAUU GCA GCCA GCAGCAU \**

**UAAGa cggggg ag ua gggaaguA AUUGUU CG UGU CUAAGUAA UGU CGGU CGUCGUA A**

**AAUCCC u - u g \ AC UUAG UUCU UUC UUAU \ -^ U**

**zma-miRgs103a AUGAAGACACGACAAUGUUCAU**

**A A GAA G GA AC AA | C**

**AGAGUCUU G GUGAG AUUG GUG UCU CAUCGC--UAGGCUUUU \**

**UUUCAGAA C uacuu uaac cac aga guaGCG AUCCGAGAG A**

**C - --- g ag -- a- \ ^ U**

**zma-miRgs103b AUGAAGACACGACAAUGUUCAU**

**A C GGAA G GA UAC AAG .-U| AUUC**

**AGAGUU UA GUGAG AUUG GU UCU GUCAC AGGCU \**

**UCUCAA AU uacuu uaac ca aga uaGUG UCCGA A**

**C A ---- g ag c-- ag- \ -^ GAGU**

**zma-miRgs104a UGGUGACCGUCUGCUUGGGUGU**

**- U - u ga -- uu .-GCCUUGUC .-AAUCUU| GU UUGC - GU UU UGU**

**UCU UGC UGA ggu cc gucugc ggguguU GAGGGGGAC GGUCCC CG UCUGC AGCU GCU GUCA \**

**GGA ACG ACU CUA GG CAGGCG UCCACGG CUUUCUCUG CCGGGG GC AGACG UCGG UGG CGGU U**

**U C U U G- UC U- \ -------- \ ------^ -- UU-- G GU -- UUC**

**zma-miRgs104b UGGUGACCGUCUGCUUGGGUGU**

**UCUUC - u ga -- uu .-G U .-AACAGUCUU| GU UUGC - GU UU UGU**

**GC UGA ggu cc gucugc ggguguU CCUGG CGAGGGG GGUCCC CG UCUGC AGCU GCU GUCA \**

**CG ACU CUA GG CAGGCG UCCGCGG GGGCC GUUUCUU CCAGGG GC AGACG UCGG UGG CGGU U**

**GGACA U U G- UC U- \ - U \ ---------^ -- UC-- G GU -- UUC**

**zma-miRgs104c UGGUGACCGUCUGCUUGGGUGU**

**UCUUC - u ga -- uu .-CCUUGUCA .-AGUUUU| GU UUGC - GU UU U UGU**

**GC UGA ggu cc gucugc ggguguCA AGGGAGAC GGUUCC CG UCUGC AGCU GCU G CA U**

**CG ACU CUA GG CAGGCG UCCGCGGU UCUCUCUG CCAGGG GC AGAUG UCGG UGG C GU C**

**GGACA U U G- UC U- \ -------- \ ------^ -- UU-- G GU -- - UCU**

**zma-miRgs105 AGGACAUGAGCGCGGAUAGCA**

**GA GACACA- A A CAG .-CAGCUACAUCAAUAAUAC - .-GC C--- .-AG| A CG**

**GG GC GUC GCGCUCAUG UCCGUU CCG UGAUUGGUUG ACGCU ACCAA CCA GCU C**

**CC cg uag cgcgaguac aGGCGG GGC ACUAACCAAC UGUGA UGGUU GGU CGG G**

**AA AAAAGAA a g aGG \ ------------------ G \ -- AAAA \ --^ C CC**

**zma-miRgs106 AGCCUAUGAGUGUCUGACAUG**

**GA| GUUUA - CU G GUG**

**UCGA UCAUGUCGGAU CUCA GGC UC \**

**AGUU Gguacagucug gagu ccg aG G**

**GA^ GUG-- u au - ACU**

**zma-miRgs107a UGUGGAUUAGGUGGGAUUGGA**

**- A a U-----------| C U CA**

**UCUUA UUCAuguggauu ggugggauugga GGAUUU AA CU A**

**AGGAU GGGUACACCUAA CUACCCUAACCU UCUAAA UU GA A**

**A A C UUUUUAAUACUU^ A - AC**

**zma-miRgs107b UGUGGAUUAGGUGGGAUUGGA**

**- CAA a | UUU**

**UCU UCCAuguggauu ggugggauugga--CGGG \**

**AGG AGGUACACCUAA CUAUCCUAACUU GCCC A**

**A AC- C \ ^ UAA**

**zma-miRgs107c UGUGGAUUAGGUGGGAUUGGA**

**- AAUU-- ag .-C| UUU**

**UCUU CAuguggauu gugggauugga GAG \**

**AGAA GUGCACCUAA UAUCUUAAUCU CUC A**

**C CAAGGC CU \ -^ CAA**

**zma-miRgs107d UGUGGAUUAGGUGGGAUUGGA**

**- CAA a | UUU**

**UCU UCCAuguggauu ggugggauugga--UGGG \**

**AGG GGGUACACCUAA CUACCCUAACCU ACCC C**

**A AUA C \ ^ UAA**

**zma-miRgs107e UGUGGAUUAGGUGGGAUUGGA**

**- CAA a U-| UCAAUCCUAAAUAA**

**UCU UCCAuguggauu ggugggauugga GAGUU \**

**AGG GGGUGCACCUAA CUACCCUAACCU UUUAA G**

**A AUA C UU^ UACUUUCUAAAACU**

**zma-miRgs108 AGACUUAGGAACGGAGGGAGU**

**GAAAAUAA U .-AAGCAAAUU .-AAAAAUCU| GU**

**GUACUCCCUCCGUUCCUAAGUCUCCA CGUAU GGA--UUUAUUC CCAUC A**

**CAugagggaggcaaggauucagaGGU GCAUA CCU AAAUGAG GGUGG U**

**GAACACUA C \ --------- \ \ --------^ AU**

**zma-miRgs109 ACUUUUAAGGUCAGGGAUGCGA**

**U A A A-- AA A- .-C| A**

**G GU GUAGG UGUCCU UGACC GAGAGU UUCC U**

**C CG CAUCC guaggg acugg uuuuca AAGG C**

**A - G agc -- aa \ -^ U**

**zma-miRgs110a UUUGAAUGCACUAGAACUAAUU**

**UUAUCUCUAGCGCAUUCAAACA**

**UU A a ac u UA AAAU .-AG| C AACAAAU**

**AG GGGUGuuugaaugc cuaga uaau GUUAGU GCUAAA GUUAGU AAUUAG UAGCC \**

**UC CCCacaaacuuacg gaucu auuA CAAUCA CGAUUU UAAUCA UUAAUU AUCGG A**

**AU C c cu U C- ---- \ --^ - CCAAUCA**

**zma-miRgs110b UUUGAAUGCACUAGAACUAAUU**

**UAA ac u .-UA| AA**

**GGG--GGUGuuugaaugcacuaga uaau GUUAGU GCUAA A**

**CCC CCACAAACUUACGUGAUCU AUUA CAAUCA UGAUU U**

**CAA \ CA U \ --^ GU**

**zma-miRgs111a UGGCUGCAGGACCAAUGUUGCA**

**GUCUUUGGC| UA AG - U UG U**

**GUGCAGCGUU UCU GC GCC ACG GAGC G**

**Cacguuguaa agg cg cgg uGU CUCG C**

**CUUGCAUUA^ cc a- u - CG U**

**zma-miRgs111b UGGCUGCAGGACCAAUGUUGCA**

**AU| UGGC UA AG C UG U**

**AUGUCUU GUGUAGCGUU UCU GCA CUACG GAGC G**

**UGCAGGA Cacguuguaa agg cgu gguGU CUCG C**

**AU^ ---- cc a- c CG U**

**zma-miRgs112 UCAAAAUCGGCUGAGUAAGGAA**

**- ca | aagg**

**UCGAUCGGUUu aaaucg--gcugagu \**

**AGUUGGCCAGG UUUGGC CGGCUUA a**

**A AC \ ^ AAAa**

**-- UG .-AGAAAAAUA GC U CU**

**UCAAC GUUUU UCUGCU GACUU UCUGACAG C**

**AGUUG CAAAG AGACGA CUGGA AGACUGUC U**

**CA GU \ --------- GA C AG**

**zma-miRgs113a ACAGAAAGAGAAGUGAGCACGC**

**GAGAGA--------| a a ACA - - C GGCCUA AG A U-- U**

**GCUGacagaa gaga gugagcacgcA GC AG CUGCG CUA GGCU GC UCCA CGA C**

**CGACUGUCUU CUCU CACUCGUGCGU CG UC GACGU GAU CCGA CG GGGU GCU C**

**ACGAUCUACCACAC^ - - AGC G U A AUAUG- -- - UUC G**

**zma-miRgs113b ACAGAAAGAGAAGUGAGCACGC**

**.-AA GC a a A---| CA**

**AGAG UGacag aagag agugagcacgcGGCG GGACGGCAUA \**

**UCUC ACUGUC UUCUC UCACUCGUGUGCCGC CUUGCUGUAU U**

**\ -- UA - - GCUG^ CG**

**zma-miRgs113c ACAGAAAGAGAAGUGAGCACGC**

**- G A--- a a .-ACA| A GC AUCUA**

**GA GG GCUGacag aagag agugagcacgcA GC GCUGC CUUGGC U**

**CU CC CGACUGUC UUCUC UCACUCGUGCGU UG CGACG GGACCG C**

**G G ACAC - - \ ---^ C A- CUUAC**

**zma-miRgs114 UGAAACCCAAUAGAAAAUCGUU**

**UGAGA CA A UG .-AA GG UU A - UGUGA**

**GAUU UUUGUUGG UUU UCU UUCUU CU CA UU UCUU A**

**cuaa agauaacc aaa AGA AAGAA GA GU AA AGAG A**

**GGuug a- c gu \ -- AA GG A C UGUAA**

**zma-miRgs115 UAGCCAAGGAUGAGCUGCCUG**

**CUCGU UCGAUC - - GU - - .-C - AA .-GA - - --| AU**

**GGCC ACAGGCAG UC UCCUUGGCUA CCG GGCGG GC CCU UAUCUG ACC UCCCU GGUU CAU UCAU A**

**CCGG Uguccguc ag aggaaccgau GGC UCGUC CG GGA GUGGAC UGG AGGGA CCAG GUG AGUG U**

**GUCCU ------ g u -- U U \ - C G- \ -- G C CG^ GC**

**zma-miRgs116 GUAAGUAGUGGAAUGAGGCUGG**

**AUGA UG A- A .-CUU .-AAAC GUCC**

**C GCC AUUC CAUUAUUU UUCCUUA UAGCACAUC \**

**g cgg uaag gugaugaa AAGGAAU AUCGUGUAG A**

**CCAA gu ag - \ --- \ ---- GUGU**

**zma-miRgs117a AAGGAACCGCCAGUGGAAAUGCU**

**- GC -| a c au uC UU CUCU**

**CGGUUG UUaag ga ccgc aguggaa gc UUCCACUG GUGGCU U**

**GCCGAC AAUUC CU GGUG UCACCUU CG AAGGUGAC CGCCGA A**

**C UC U^ C A CU UA -- CUCA**

**zma-miRgs117b AAGGAACCGCCAGUGGAAAUGCU**

**- GC -| a c au uC UU CUCU**

**CGGUUG UUaag ga ccgc aguggaa gc UUCCACUG GUGGCU U**

**GCCGAC AAUUC CU GGUG UCACCUU CG AAGGUGAC CGCCGA A**

**C UC U^ C A CU UA -- CUCA**

**zma-miRgs117c AAGGAACCGCCAGUGGAAAUGCU**

**C U -- ---| a g AU U CUCUCU**

**GG UGG CUUa agga ccgccagug aaaugcu UUUCACU GCGG U**

**CC ACC GAGU UCCU GGCGGUCAU UUUACGA AAAGUGA CGCC A**

**A - AA AUA^ - G -- C AACACA**

**zma-miRgs118a UGUGGAUUGAAUGAGAUUGGA**

**-| UAACA aa a .-aAAAAAAUAUGAAA UG CU**

**UUCUA uguggauug ug gauugg GAUUU A \**

**AAGAU ACACCUAAC AC CUAACU UUAAG U U**

**U^ ----- CC C \ -------------- GU UG**

**zma-miRgs118b UGUGGAUUGAAUGAGAUUGGA**

**- AUAAC-| aa AAAAUUAUGAAA UG CU**

**UUCU Auguggauug ugagauugga GAUUU A \**

**GAGA UACACCUAAC ACUCUAACUU UUAAG U U**

**C CUUAGA^ CC AUUCAAAG---- GU UG**

**zma-miRgs118c UGUGGAUUGAAUGAGAUUGGA**

**C--------- UC AC- - UAA C A U**

**UCUA CU UCAAUUCACAU AGAU GAG UAACCGAACAA CCC \**

**aggu ga aguuagguguA UCUA CUU AUUGGCUUGUU GGG U**

**AUAAAAAAAA ua gua C UC- - C A**

**zma-miRgs118d UGUGGAUUGAAUGAGAUUGGA**

**- AU- a .-a| AG**

**AUCU UUCAuguggauug augagauugg AAAAAAU \**

**UAGA AGGUACACCUAAC UACUCUAACU UUUUUUA G**

**U GUU C \ -^ AA**

**zma-miRgs118e UGUGGAUUGAAUGAGAUUGGA**

**-| AUCC a u UUUAAAAUCCCAAAAAAUCAA**

**UCUCA Auguggauug augagau ggaUGAG A**

**AGAGU UGUACCUAGC UACUUUG CCUACUU A**

**A^ AAUA C - UGACCUUUUUUUAAUCUUAUU**

**zma-miRgs118f UGUGGAUUGAAUGAGAUUGGA**

**- UU AUU a .-UGA| AAA**

**U CA CAuguggauugaaug gauugga GUUU \**

**A GU GUGUACCUAACUUAC CUAACUU CAAA U**

**A GG CAU A \ ---^ CCC**

**zma-miRgs119a AGACGUUAGAUCUGAGAUGGA**

**A| A A GU A U CCU AACCACAACACUUCACCA**

**AUUAUCGCCGUUCAUCUCA AUCUAAC UCUCUGGUUGCUU UAUAUUUU CAAA AACA UC C**

**UAGUAGUGGCagguagagu uagauug agaGAUCAACGAG AUAUAAAA GUUU UUGU AG U**

**-^ c c AC C C AGU ACUUAAGACUUCACCACU**

**zma-miRgs119b AGACGUUAGAUCUGAGAUGGA**

**UAA U ACUCU A U A UAUA A G ----| CA**

**AUCA CAUC UCUCAGAUCUAGC UCUCUG UUG UC UAUUUUGC AA AACA CCAUC A**

**UAGU GUag agagucuagauug agaGAC AAC AG AUAAAAUG UU UUGU GGUGG A**

**UAA U gu--- c U G CCAC A A AGAA^ CG**

**zma-miRgs119c AGACGUUAGAUCUGAGAUGGA**

**AUUAUC A A C ----| C U CA U CACC UACAC**

**GUCAUCUAUCUCAGAUCUAAC UCU UA UUGU UAUA UUUACAAA AA CC UC AC \**

**UAGUagguagagucuagauug aga AU AACG AUAU AAAUGUUU UU GG AG UG U**

**UAUCAA c G U AUAC^ A C AC U ACUU CUAUC**

**zma-miRgs120a GGAGGGAUUGGAGGGGCUAA**

**U AAU- a--| g UC A**

**CCA AUCggaggg uuggaggg cuaaAAUCUCAU UUAUUUA A**

**GGU UGGUCUCCC AACCUCCC GAUUUUAGAGUA AAUAAGU A**

**C GUUU CUA^ - GA U**

**zma-miRgs120b GGAGGGAUUGGAGGGGCUAA**

**-| A AA c AUCCCAUUC**

**CC CAA CCggagggauuggagggg uaaA \**

**GG GUU GGCCUCCCUAAUCUCCCC AUUU U**

**C^ - CC - AAAACUUAU**

**zma-miRgs120c GGAGGGAUUGGAGGGGCUAA**

**AGCCCCUUCAUUCCCCUCCGG**

**U AAU- au-| A**

**CCA ACCggaggg uggaggggcuaaAAUCCCCUCCUUAUUCA A**

**GGU Uggccuccc acuuccccgaUUUUAGGGGAGGAAUAAGU A**

**C AUUU cuu^ U**

**zma-miRgs120d GGAGGGAUUGGAGGGGCUAA**

**--| C A - C**

**CCA AA ACCggaggg auuggaggggcuaaAAU C**

**GGU UU UGGCCUCCC UAACCUCCCCGAUUUUA U**

**CA^ - A C U**

**zma-miRgs121 UUGGCUUCUGUGCUGACGUCAU**

**AUG UUU - G - - UG .-AUCAUAUAUGU| UGGUC**

**GC GUUUGA GA GU GGCG AGAAGC AGGA CUAGGA \**

**CG CGGACu cu ca ucgu ucuucg uCUU GGUCUU U**

**AUG --- a g g g gu \ -----------^ UGAGU**

**zma-miRgs122 AAGCUCUCGGCGAAAUGUGAC**

**GU Caa ugu CA U .-A| ACCAGG**

**CGAGUGU gcucucggcgaaa gac UCGGU AAUU UGUCGAGU \**

**GCUCACA UGAGAGCCGUUUU CUG AGCCA UUGA ACGGCUCA A**

**CU A-- UU- -- C \ -^ CAAUUG**

**zma-miRgs123 UUAACUGAUCUGGCCACUUUUC**

**ACCA ac- cu- u CCU .-GAU| AU**

**CUUUGGuua ugau ggccacu uuc GGGUU UGAAG \**

**GAAAUCGGU AUUG CCGGUGG AAG UUCAA ACUUC U**

**UUCG GUU AAU U UU- \ ---^ AA**

**zma-miRgs124 AUAAACGUUCGUAGGUAUGG**

**AC CCC acg- .-CUCUACCCAUACCUGUACCCGCC| A**

**UCGUG auaa uucguagguauggAU GGGUAUCUGCU U**

**GGCGC UAUU AAGCGUCCAUACCUA CCUAUAGACGG C**

**UA C-- AUAA \ -----------------------^ C**

**zma-miRgs125 AGGCGGAGGUCAAUCUGGCGCG**

**- U C g- agg- aa gGG | A**

**CAGGC GCG ag cgg uc ucuggcgc CGGAG--GGCA U**

**GUCCG CGC UC GCC AG AGGCCGUG GCUUC CCGU C**

**G U U AG CGAG CC AA- \ ^ U**

**zma-miRgs126a ACGGGCUGUCCGGUGCACACC**

**C U CA U - G CAAA----- CU -| GGUA - AAUGUA GAAUU A A C**

**CGGAC GUCCGGUG CACCGGAC GUCCG GU UGC UUUG CA AACU UGGCUUC AACUCUGG GA UC UA GCUUU C**

**GCCUG CAGGccac guggccug cgggc CG ACG AAAC GU UUGA AUCGAGG UUGAGGCU CU AG AU UGAAA A**

**- C ac u a A AACAGCAUA CG A^ AA-- C AAAA-- ACU-- A A A**

**zma-miRgs126b ACGGGCUGUCCGGUGCACACC**

**G - CA U G .-GCCAAA CU -| GGUA - GAUGUA A CAUAA C**

**GGAU GUCCGGUG CACCGGAC GUCC GUGU UUUG CA AACU UGGCUUC AACUCUGG GAU AUUU GCUUU C**

**CCUG CAGGccac guggccug cggg caCG AAAC GU UUGA AUCGAGG UUGAGGCU CUA UAGA UGAAA A**

**G C ac u - \ ------ CG A^ AA-- C AAAG-- C ACA-- A**

**zma-miRgs126c ACGGGCUGUCCGGUGCACACC**

**G - CA U - G CAAA----- AU -| GGUA - GAUGUA GA CAUAA C**

**GGAU GUCUGGUG CACCGGAC GUCCG GU UGC UUUG CA AACU UGGCUUC AACUCUGA GA AUUU GCUUU C**

**CCUG CAGGccac guggccug cgggc CG GCG AAAU GU UUGA AUCGAGG UUGAGGCU CU UAGA UGAAA A**

**G C ac u a A AACAACAUA CG A^ AA-- C AAAG-- AC ACA-- A**

**zma-miRgs127 UUUGAGUCCCUCCGGCAUUCUG**

**-| ACG u agu c u-- AU**

**GCCA UUC uug cccu cggcau cug G**

**UGGU AAG AGU GGGA GCCGUG GAC A**

**G^ CA- U GU- A CAU GA**

**zma-miRgs128 GUCUUAAACACUCGGCAAAG**

**GCCGA UCC UUCA | UCCGA**

**GUGUUUU UUGCCGAGUGUUU ACACUCGGUAAAG--UCUUUGCCGAGUG \**

**CGCAAga aacggcucacaaa ugUGAGCCGUUUC AGAAACGGCUCAU A**

**CUUAG --- uuc- \ ^ GAAAA**

**zma-miRgs129a AAGCUAACGAGCUGGCUCGACU**

**- A Ua - ug - | A**

**CU GCUCGU agcu aacgagc gcucga cuC--GGUUCGUUA \**

**GA CGAGCG UCGA UUGCUCG CGAGCU GAG CCGAGCGAU G**

**A C C- U GU C \ ^ U**

**zma-miRgs129b AAGCUAACGAGCUGGCUCGACU**

**GAUA| U UA ACUC CAUU AU**

**GGGC AGA UCGAGCCAGUUCG GCUU CGA U**

**CUCG UCu agcucggucgagc cgaa GCU G**

**AUUA^ - c- aau- UU-- CG**

**zma-miRgs129c AAGCUAACGAGCUGGCUCGACU**

**AGUA| A UA C GCUC CGUC CA**

**GGGCU GA UCGAGCCA CUCG GGCU CAAGUU A**

**CUCGG Cu agcucggu gagc ucga GUUCAG C**

**AUUG^ - c- c aa-- aUU- UC**

**zma-miRgs129d AAGCUAACGAGCUGGCUCGACU**

**AUA---------| A GUUC CGUC CG**

**UCGA CUAGCUCG AGCU CGAGCU A**

**agcu ggucgagc ucga GCUCGG G**

**AUCAAAUUGUuc^ c aa-- aUU- UA**

**zma-miRgs130a UUGGAUUGGUUUAGAGUGGUU**

**UAUAAA| g u ag UU GCA**

**ACAGuu gauuggu uag ugguuCAAC GC \**

**UGUCAA CUGACCA GUC GCCAAGUUG CG U**

**UUUAUG^ G C CG U- AUG**

**zma-miRgs130b UUGGAUUGGUUUAGAGUGGUU**

**UAUAAA| g u ag UU GCA**

**ACAGuu gauuggu uag ugguuCAAC GC \**

**UGUCAA CUGACCA GUC GCCAAGUUG CG C**

**UUUAUG^ G C CG U- AUG**

**zma-miRgs130c UUGGAUUGGUUUAGAGUGGUU**

**UAUAAA| g u ag UU GCA**

**ACAGuu gauuggu uag ugguuCAAC GC \**

**UGUCAA CUGACCA GUC GCCAAGUUG CG C**

**UUUAUG^ G C CG U- AUG**

**zma-miRgs130d UUGGAUUGGUUUAGAGUGGUU**

**UAUAAA| g u ag UU GCA**

**ACAGuu gauuggu uag ugguuCAAC GC \**

**UGUCAA CUGACCA GUC GCCAAGUUG CG C**

**UUUAUG^ G C CG U- AUG**

**zma-miRgs131 UCUGAUGCAGAAUGAGAAAGUC**

**UA | a**

**GGGUG--UAUucug \**

**CCCAC guaagac u**

**UA \ ^ g**

**agaaagucA AUCAC A**

**UUGCUUAUGC GCUU C**

**GACGAGUACG CGAA U**

**CCUAUUAA- C---- C**

**zma-miRgs132 AUAUUAUUAUGAUUGGAUGGCU**

**UAUAGAG| u a a C- U UGGG**

**UCAa auuauuaug uugg uggcuG GUG CCA \**

**AGUU UAAUAGUAU AACC ACCGAU CGC GGU C**

**GUACGCG^ - A G CU U UAGA**

**zma-miRgs133 CGGUAGUGGAAUGUGGUGGC**

**UC - A g aa g - .-UAA ---- .-AUGACAAG| GUAGA**

**GCG GG GUc guagugg uguggu gc GC GCA GUGCGCCUG GAGC \**

**CGC CC CAG CGUCGCC ACGCCG CG CG CGU CACGCGGGC CUUG A**

**GA U A A -- A U \ --- CCUC \ --------^ AGGUU**

**zma-miRgs134 UCUAGUGCUUGAAGAACCGCA**

**GGACAUCUCUA ACA C - GGUUUU UAC A .-AAA --| GAGAG U**

**GC GUUCU UAAGU CUAGAUAC AGCUA AACCC CAU UGAGU UGUAUCUU GUUC A**

**cg caaga guucg gaucuAUG UCGAU UUGGG GUA AUUCA ACAUAGAA UAAG A**

**UUAUGAUACAa c-- a u ------ UCU - \ --- GA^ ----- U**

**zma-miRgs135a UGAGGACGAAUAGGAUGAGCA**

**- AAAUU- ug --- g -| UG AU**

**GCA UG agg acgaaua gaug agca C \**

**CGU AC UUC UGUUUGU CUAC UUGU G C**

**U AAAACC GU CAU G G^ UA UC**

**zma-miRgs135b UGAGGACGAAUAGGAUGAGCA**

**- AAAUU- ug --- g -| UG AU**

**GCA UG agg acgaaua gaug agca C \**

**CGU AC UUC UGUUUGU CUAC UUGU G C**

**U AAAACC GU CAU G G^ UA UC**

**zma-miRgs135c UGAGGACGAAUAGGAUGAGCA**

**- AAAUU- ug --- g -| UG AU**

**GCA UG agg acgaaua gaug agca C \**

**CGU AC UUC UGUUUGU CUAC UUGU G C**

**U AAAACC GU CAU G G^ UA UC**

**zma-miRgs136 AUGAGCACAAUGUCGUCGACGU**

**CC AGA A .-AC U .-G A CCC .-G| UU**

**GCUCC AUGUCGAUGGCGU GUG GC GGUGCA GAAG GA UGGGGCC GCG \**

**UGAGG ugcagcugcugua cac CG UCGCGU CUUC CU ACCCCGG UGU C**

**GA AGA a \ -- - \ - - AA- \ -^ GU**

**zma-miRgs137a UGAAGGGGAUUGGAGGGGAUU**

**UGGGAGGGA| u ga A CA G A**

**Uugaagggga ug ggggauuAA UCCCUU UA UCA A**

**AACUUUCCCU AC CCCCUAAUU AGGGGA AU AGU A**

**---------^ U UC A UG A U**

**zma-miRgs137b UGAAGGGGAUUGGAGGGGAUU**

**AGGAA| Uugaa g - --- CC**

**GGGA ggggauugga gggauuAAAUCCC UUC UCAG A**

**UCCU CCCCUAACUU CCCUAAUUUAGGG AAG AGUU A**

**A----^ ----- A G AUA AA**

**zma-miRgs138 UGUUUGGAAGCAAGGGGAAUG**

**A| CAU a c - A A C U - U U A**

**AGG GUUuguuugg ag aaggggaau gA GGGAUUGGAG GG UAA GUC CC UGCUAU CA U**

**UCC CGAACAGACU UC UUCCCCUUA CU CCCUAACCUC CC AUU UAG GG AUGAUA GU U**

**A^ --- A - U C C U U A U C U**

**zma-miRgs139a ACUAAAUGGGACUAAAAUGG**

**U C--| CCG AUU**

**UUAGUC CUGUUUUAGUCCCA UUUGGUA \**

**AAUCAG gguaaaaucagggu aaaucaU U**

**U AGA^ --- GGA**

**zma-miRgs139b ACUAAAUGGGACUAAAAUGG**

**GUUUUAGUCCUACUGUUUGGUAA**

**U --| acu aaUU**

**UUAGUCCCU guuuuaguccu guuuggu \**

**AAUCAGGGA uaaaaucaggg uaaauca U**

**U gg^ --- AUGA**

**zma-miRgs140a AAUUCAUAUGGAUUGAGGUA**

**U AU-| U - UAU AUU GA- GACU**

**UGUUCGGUU UUUCAAUCCAUAUGGAUU GAG GGGAUUGA GG GGGA GAUUUU \**

**ACAAGCUAA ggaguuagguauacuuaa CUC CCCUAAUU UC CCCU UUAGGA U**

**- Gau^ - U --- --- AAA AUGA**

**zma-miRgs140b AAUUCAUAUGGAUUGAGGUA**

**U C AU- -| A UAU GAAGA A AC**

**GUU GGUU UUUUAAUUCAUAUGGAUU AG GGGGAUUGA GGAUU GA UUUUG U**

**CAA CCAA ggaguuagguauacuuaa UC CCCCUAACU CCUAA UU GAGAU U**

**A A Gau C^ - CC- AG--- A CA**

**zma-miRgs140c AAUUCAUAUGGAUUGAGGUA**

**U G AU- C- AGAUUAAA - .-AA| AG**

**UGUUC GUU UUUUAAUCUAUAUGA UGA GG GGAUUGA CAGAUUG \**

**ACAAG CAA ggaguuagguauacu aCU CC CCUAACU GUUUAAU A**

**- G Gau ua -------- U \ --^ AG**

**zma-miRgs141 GGAGAUAGGUUAAUGCCGUCG**

**ACGGUUUGAACCUUAUGUCCGA**

**G g -| aau C UA**

**GUUAAUACCGUCgga au agguu gccgucgGA AU \**

**CGGUUGUGGCagccu ua uccaa uggcaGCUU UA C**

**- g u^ guu U UC**

**zma-miRgs142 AACGGUUGCGCAUUUUGUCU**

**CCAGG--| GA - U ------ GAUCA- C**

**UC GGACGAGGU CGUAGC CGU GGUA GAC U**

**AG ucuguuuua gcguug gca CCGU CUG U**

**AGUAAUG^ G- c - aUCCUU AUAAAC G**

**zma-miRgs143 UAGACUGGCACUGUCACCUUCA**

**G| GC UG C A - U GG AG**

**GUC CU GU GAAG UGAUGG GCU GUC ACC U**

**CAG GA Ca cuuc acuguc cgg cag uGG C**

**-^ AC GU - c a u a- AU**

**zma-miRgs144 AAUGGACGUGCCGGGCCAGCC**

**CUGGGCACGGCACGAUAUUG**

**UA U A ga| g - CG A**

**CAUG CC aaug cgugccg gcc agcc GC C**

**GUGC Gg uuau gcacggc cgg ucGG CG G**

**UC - - a-^ a g CA A**

**zma-miRgs145 AUCGAAUGAGAUUGGGGGGAU**

**- C uga -| UC U A**

**UUGUG CUGGaucgaa gauugg ggggauUAAAUCU UUCUA UCA U**

**AAUGC GACCUAGCUU CUAACC CCUCUAAUUUAGA AGGAU AGU U**

**C A CCC U^ GA C U**

**zma-miRgs146 CAAGCAAACUCAUCAACCUCAA**

**UG| CAA U U GG GCAAA U UGA**

**UGGAUGAC UGAGGUUG UGA GU GU GGUGG GGG C**

**ACCUACUG acuccaac acu ca cg UCACC UCU A**

**GA^ a-- u - aa aac-- - UGA**

**zma-miRgs147a UGGAUACGGAUACGGGUAGCAC**

**A GCC AA- GA AU .-UUUAGCAACG| A U**

**CGGG UAAGUGU UCGUA CUG UGUCUA GUGGC CC C**

**GCCU GUUcacg ggcau ggc auaggu CACCG GG A**

**- AUA aug a- -- \ ----------^ - C**

zma-miRgs147b **UGGAUACGGAUACGGGUAGCAC**

**.-UUUGUACCC -- a c-- .-gcacUU| C**

**Auggaua cgg ua gggua GAUAUC G**

**UACCUAU GCC AU CCCAU UUAUAG U**

**\ --------- AA C AAA \ ------^ G**

**zma-miRgs148 UGCGAGUUGGGCAUAACCUUUU**

**C GAACCC AA A .-CCAUCAU| CAU A**

**ACUUG AAGG GU GUUCAACUCGC UGA CUCAA C**

**UGAAC uucc ua cggguugagcg ACU GAGUU U**

**C ACCGuu aa - \ -------^ ACU U**

**zma-miRgs149 AAGACUGAUUUGAUGACAAG**

**UUAUAC A a-| a UA GA- GAGU U A- A**

**CUU aag cugauuug ugacaagGAAAUUG AGG UCCAU GAGGAAAUC CCU CUAUUCA A**

**GAA UUC GACUAAAC ACUGUUCUUUUAAC UCC AGGUA CUCUUUUAG GGA GAUAAGU A**

**AAGGU- A CC^ C C- GCC ACU- - AC U**

**zma-miRgs150 AUACAUGGAUUGAAUGAGAUU**

**UA UUa c .-AA| AA**

**UUCUCA ua auggauugaaugagauuA AAAUUU G**

**AAGAGU AU UAUCUAACUUACUCUAAU UUUGAA A**

**UC UAG A \ --^ AA**

**zma-miRgs151 UAGUGAUAGUGACAGGAGCGAC**

**UACAAG| G AA - G UC UG A UC**

**UGU GU GC CC GUCACU AU CUA AUG \**

**ACA ca cg gg caguga ua gau UAU C**

**GGAAGA^ A g- a a -- gu C UA**

**zma-miRgs152 AUCGGAGAGGAUUGGAGGGGC**

**-| UC AAU- a A C A**

**GAG CA aucggag ggauuggaggggcUAAAAUCC CUC UUAUUCA U**

**CUC GU UGGCCUC CCUAACCUCCUCGAUUUUAGG GAG AAUAAGU U**

**C^ U- GCUU C G A U**

**zma-miRgs153 AUCUUGCGAGAUUGGAACAGG**

**U AA- C A .-UUCGU| UAUAUGA**

**AUUCU GCCC GUUUCAAUCUC CGGGAUAAACUUUAGC GCUAAACUUUAGC \**

**UAAGA CGgg caagguuagag guucuaUUUGAAAUCG CGAUUUGAAAUCG A**

**- AUC a c \ -----^ UGAAGUU**

**zma-miRgs154 UACGAUGGUACAUGAACGUCAU**

**UGU Aua u ga g-- - .-AACAU| UC A CAC**

**GUGGCU cga gguacau ac uc auGUGU UGG CUGC GC A**

**UACCGG GUU CCGUGUA UG AG UAUACA ACC GACG CG A**

**CUU --- C AA GAA U \ -----^ UC A AGC**

**zma-miRgs155a AGUUGUUUUGGUGAUUAAGUG**

**CUAA - AUC CA .-A - .-ACAAUGGUGUUGU UGUG C .-UUUGUU| U UUG**

**GUU GUCA AAUUACCAAAAC AAC AGGUC CUU GUUUUUGGAU UUUUUGCU UUGA GUG UC A**

**CAA CAgu uuagugguuuug uug UCCAG GAG CGGGAAUCUA AAGGACGA AACU CAC AG U**

**CACA C gaa -- \ - A \ ------------- ---- A \ ------^ U UGU**

**zma-miRgs155**b **AGUUGUUUUGGUGAUUAAGUG**

**- UUG ug uu- --- CUCAACA .-ACA GACU .-A --- .-AUA A-| AG**

**GGCCAU Uagu uu gg ugauuaagug AACC AUG AACU UCUUUAU AUGAGUUGGAGC GGUUC UUCAA A**

**UUGGUA AUCG GA UC ACUGAUUCAC UUGG UAC UUGG AGAGAUG UAUUCAAUCUCG CUAAG GAGUU A**

**U UCA UU UUU UUA CAUC--- \ --- ACAU \ - UUU \ --- AG^ AA**

**zma-miRgs156 AGGGUCCGGACUGUCCGCGCCA**

**CU .-GCCag -| ccaG GC AC**

**GUUCU gguc cggacuguccgcg GGG CGG \**

**CAAGA UCAG GCCUGGCAGGCGU CCC GUC U**

**CC \ ----- C^ ACCA AC UG**

**zma-miRgs157 GAUUUUGAUGAGUUGGAAUGAA**

**AAUUU a au u u .-C .-UUAUUC ACAAC--- A-| CC UAG CGCU U AC**

**AUAUAg uuuug gag uggaa gaaU CUAGC CGGU CGA CAGG CU GUGGC UGCAG CUAC G**

**UAUGUC AGGAC UUU ACCUU CUUA GAUUG GCCG GCU GUCC GA CACCG AUGUC GGUG C**

**AGCCC - CG - - \ - \ ------ CUUUUCCA AC^ UU --- UAU- - AC**

**zma-miRgs158a GUAAAUGAUGAUCUUACUCGGA**

**- UG .-UUC g uua .-ga .-C| U**

**UU AGC guaaau augauc cucg UUGC CC U**

**AA UUG UAUUUG UACUAG GAGC AACG GG C**

**A GU \ --- - --- \ -- \ -^ A**

**zma-miRgs158b GUAAAUGAUGAUCUUACUCGGA**

**- UG .-UUC g uua .-ga .-C| U**

**UU AGC guaaau augauc cucg UUGC CC U**

**AA UUG UAUUUG UACUAG GAGC AACG GG C**

**A GU \ --- - --- \ -- \ -^ A**

**zma-miRgs158c GUAAAUGAUGAUCUUACUCGGA**

**- UG .-UUC g uua .-ga .-C| U**

**UU AGC guaaau augauc cucg UUGC CC U**

**AA UUG UAUUUG UACUAG GAGC AACG GG C**

**A GU \ --- - --- \ -- \ -^ A**

**zma-miRgs159 UAUAUUUGUGUGCAUCUAGGCU**

**0**

**G - .-AC| u ug u uC**

**GAU CUCU uauau ugug ca cuaggc \**

**CUG GAGA GUGUG ACGU GU GAUUCG C**

**A U \ --^ U GU - UA**

**zma-miRgs160 ACUAAAAGGCUAUGUUGAUGA**

**- UUU-- aaa .-uauguug| UAGU A**

**CUUG GGUacu aggc auga UUAU A**

**GAAU CCGUGG UCCG UACU AGUG U**

**C UUAAC --- \ -------^ UU-- A**

**zma-miRgs161 UUUUGUAUCGUAGAUAACAC**

**G U UUUAU - U-- U AA .-AUACA| CA U**

**CU UAAAU GUG UGUCUAUG UAAAAUA UA UUUGUG AGU GAUGUUU \**

**GA AUUUG cac auagaugc guuuuGU AU AAACAU UCA CUACAAG A**

**A U U---- a uau C A- \ -----^ UC G**

**zma-miRgs162 AAUUCAUAAGUAGAGAUUGUUC**

**C C A U AA A UGUA UUCUU--- UU UU AGUCUUCU .-UAAUCACUCACC U**

**AUU AAAG AGA CAAUCU UACU UGUGA UUCC GUUUAAA GG UUG ACAU AUG**

**UAA UUUC Ucu guuaga auga auacu aAGG CGAAUUU UC AAC UGUA UAC**

**U C - u g- - ua-- UACUUUAU UC U- GUAAUCUU \ ------------ U**

**zma-miRgs163 UGUAAGACACAGGUCCGAAGAG**

**U CUAA A AC U AAU UGUCC .-UCAC .-ACUUU .-UUUUA| CU**

**UCAC GA UUCUUCG ACCUGUG UCUUG UC UAUUG UUCUC CUUGA AGCUCAAA \**

**AGUG CU gagaagc uggacac agaau AG AUAAC AAGAG GAACU UCGAGUUU C**

**- UAC- G c- - gu- UC--- \ ---- \ ----- \ -----^ UA**

**zma-miRgs164 CUGAGUUGGCUAGGUGGUUUG**

**G A UG A CGA GAU U .-C| CA**

**GAGUGCUACUCAGACCACCUA CCAACU GCUAGAG CCCUUUCGC UUAG CA UUUUUUG CUGG \**

**CUCACGAUGAguuugguggau gguuga cGAUCUC GGGAAAGCG AAUC GU GAAAGAU GACC A**

**- c gu C A-- --- C \ -^ AU**

**zma-miRgs165 AUGGGGAGGCUGCUGGAGUGA**

**GUA A .-CG - CCC UUU**

**UUCAUCACUCCA CGGCCUCUCCAUCC UUCC CCAU CA U**

**AAGUagugaggu gucggagggguaGG GAGG GGUG GU A**

**AC- c \ -- A C-- UUC**

**zma-miRgs166 UUGAUGCAGUGGCUGUAAAGGA**

**- G - uga- - ug u- C .-C GA | AU**

**GG UGGA UCUu ugc ag gcug aaagga AC GGCGC AGG--CCACUG A**

**CC GCUU AGAG AUG UC CGAC UUUCCU UG UUGUG UUC GGUGAU G**

**A G G CGAG U GU CU C \ - AG \ ^ GC**

**zma-miRgs167 GUGGAUAUAUGGCUCACUCGU**

**AC AU C -- CG A AUUUACCCUAUAC**

**CCC CGGGU AGCCAUA UCCAC GUGG UAUGA U**

**GGG gcuca ucgguau aggug CACC AUACU C**

**CU Cu c au -- C CAUAUCCCCCAUA**

**zma-miRgs168 UUGGUCCGAAGAAGAAGAUGCA**

**UU --- UAA CG - UA .-AUA| G A**

**GUGGUG GC CUUC UUUUCGGAUC AG CCC GG UU A**

**UACUAC cg gaag agaagccugg uC GGG CU AA A**

**UG AAa ua- a- u GC \ ---^ G C**

**zma-miRgs169a UCCAAAUCGUCGAAGCCGCAU**

**U AG- - UC U - - CU---| C C UU**

**GGU UGGA GUGGUUUUG CG UUUGGA GUGU GGC AUU UCUG UU C**

**UCA AUCu cgccgaagc gc aaaccu CACA CCG UAA AGAC AA C**

**U GGA a u- u C U CUCCC^ U - UA**

**zma-miRgs169b UCCAAAUCGUCGAAGCCGCAU**

**U AG- - UC U - - CU---| C C UU**

**GGU UGGA GUGGUUUUG CG UUUGGA GUGU GGC AUU UCUG UU C**

**UCA AUCu cgccgaagc gc aaaccu CACA CCG UAA AGAC AA C**

**U GGA a u- u C U CUCCC^ U - UA**

**zma-miRgs169c UCCAAAUCGUCGAAGCCGCAU**

**- .-CGC - c a .-CGACG| UUCC**

**CGUCAA Cucca aaucgu g agccgcau GCC \**

**GCAGUU GAGGU UUGGCA C UCGGUGUG CGG A**

**A \ --- G - - \ -----^ UAAU**

**zma-miRgs170 CUUGCUUGGGACAAAAGGCUU**

**- G -| uu U UUGUUU**

**CCUAC CCAAcuugcuugggac aaaaggc UGUUG UGUUG A**

**GGAUG GGUUGAACGAACCCUG UUUUCCG ACAAC ACAAC U**

**C G A^ UU U UCUGGU**

**zma-miRgs171 UGCACAUAAGGUCUGAUUGCA**

**ACAACU GU CA C G C GA- G .-UCCAACC| UA**

**AAUAUGCAAUUAG CUUAUGUGCAAG UG AAGC AGGUC CU UUCGU AGAUUAUGA AUG \**

**UUAUacguuaguc gaauacacguUC AC UUCG UUCAG GA AGGCA UCUAGUACU UAC U**

**CAUCAC ug CC A - A ACC A \ -------^ AC**

**zma-miRgs172 UGAAGAUGUGGUUCGUGAGAGG**

**A AG g a cgug .-AUAGCA A .-AA| ACA AGCG**

**CAA CACCu aag ugugguu agaggC AGGUC ACACUCA CU UUGC \**

**GUU GUGGA UUC GUACUAG UUUCCG UUCGG UGUGAGU GA AACG A**

**G G- G - AG-- \ ------ - \ --^ GG- AGAA**

**zma-miRgs173 CUUUGAGGGGAAUGUUGUCUGG**

**-| UCU u uu CU -- A ACGCA- A**

**AGCUU UAcuu gaggggaaug gucugg CGAGGU GCAG AAC GAUCUC \**

**UCGAG AUGAA CUCCCCUUAC CGGACC GCUCUA UGUU UUG CUGGAG U**

**C^ --- - UU AG GU G GUUCUG C**

**zma-miRgs174a UGCAAGAUGGUUGACUCAAGCU**

**AA .-UUu - u a a .-UAGCUGAUUUAA| CU**

**UUAUCC gc aagaugg ug cuc agcuU GGUCC U**

**AAUGGG CG UUCUGCU AC GAG UCGAG CUAGG G**

**UA \ --- U - C C \ ------------^ UU**

**zma-miRgs174b UGCAAGAUGGUUGACUCAAGCU**

**AA .-UUu - u a a .-UAGCUGAUUUAA| CU**

**UUAUCC gc aagaugg ug cuc agcuU GGUCC U**

**AAUGGG CG UUCUGCU AC GAG UCGAG CUAGG G**

**UA \ --- U - C C \ ------------^ UU**

**zma-miRgs175 AUCCUCGGUCACCAAAUCAGC**

**C AUGUU A UA .-UA| G**

**GAC AGGGCU AUUUGGUGAU GGGAUCCC GG G**

**UUG UCUcga uaaaccacug uccuaGGG CC A**

**- AGAU- c gc \ --^ U**

**zma-miRgs176 UACUAGUAGUGGAUUCUUAUGC**

**AGC AUG A - UG----| C AAUUAC A UAAAGAAAAG U**

**AACA GG AUAA AAUCC AGUA GC UC GCAAGACUGA--CCCGAC ACUC U**

**UUGU Cc uauu uuagg ucau CG AG CGUUUUGGCU GGGUUG UGGG A**

**AU- AAA g c ugauga^ U AAA--- A \ UUGGGUUGUA A**

**zma-miRgs177a UGGACUGAGCGCAUAAUAUCCA**

**CUUAAGUCA cu c u u- .-aGA| A G- U CC**

**Cugga gag gca aaua cc GAG UC GGGGC AAU \**

**GACCU UUC UGU UUAU GG CUC AG CUCCG UUA A**

**UUUCUCUGG CU - U UU \ ---^ - AG U AC**

**zma-miRgs177b UGGACUGAGCGCAUAAUAUCCA**

**CUUAAGUCA cu c u u- .-aGA| A G- U CC**

**Cugga gag gca aaua cc GAG UC GGGGC AAU \**

**GACCU UUC UGU UUAU GG CUC AG CUCCG UUA A**

**UUUCUCUGG CU - U UU \ ---^ - AG U AC**

**zma-miRgs178 UGGGUCAAGAAAGUAGAUGAAG**

**- C--| c g g agAAGUUGA U**

**UGCCA CCUUugggu aagaaa ua auga UGGGUA G**

**ACGGU GGGGACCUA UUCUUU AU UACU ACCCAU A**

**C CAA^ C - G G-------- U**

**zma-miRgs178 AGGAUGAGGAGUAGCAUGUGAA**

**- AGAAAC -- gua .-gaaGAUUGAUGAGGUGAUGAUGAAUGUACAAUAG| U**

**GGUC aggau gagga gcaugu GAAC A**

**CCAG UCCUA CUCCU UGUACG UUUG A**

**G CAGGU- GG GC- \ ----------------------------------^ A**

**zma-miRgs180 UCAAAAAUAGUGAUUACUGCAU**

**- GU A a- u a .-AAUCA| GU A**

**UAUAA G Uucaaa auag gauu cugcau AUGAAGU CA C**

**AUGUU U AAGUUU UAUC UUAA GACGUA UACUUUA GU C**

**C UG - CA U A \ -----^ AU A**

**zma-miRgs181a UGGCAAUACUGCAGUCAUGACA**

**UUGC ACAC CU C ACAA .-C CC .-UGUACGAC| AU**

**AGGCU CAUGGC CAGU UU GCCACC UAGCUC CA GGUC \**

**UCUGA guacug guca aa cgguGG AUCGAG GU CCAG A**

**GUUU Aaca ac u ---- \ - UA \ --------^ AC**

**zma-miRgs181b UGGCAAUACUGCAGUCAUGACA**

**- U AUG g ----| aAAG**

**CCA GU Gu gcaauacu gcagucaugac U**

**GGU CA CA CGUUGUGA UGUCGGUACUG C**

**U C AA- - GAAC^ CUUU**

**zma-miRgs181c UGGCAAUACUGCAGUCAUGACA**

**UUGC ACAC CC C ACAA .-UC CC ----- A-| AC AU**

**AGGCU CAUGGC CAGU UU GCCACC AGUUC CA UGU CG GGUC \**

**UCUGA guacug guca aa cgguGG UCGAG GU ACG GC CCAG A**

**GUUU Aaca ac u ---- \ -- UA UUUUA AA^ -- AC**

**zma-miRgs181d UGGCAAUACUGCAGUCAUGACA**

**UUGCA ACAC CC C ACAA .-CC CC .-UGUACGAC| AU**

**GGCU CAUGGC CAGU UU GCCACC AGCUC CA GGUC \**

**CUGA guacug guca aa cgguGG UCGAG GU CCAG U**

**GUUUC Aaca ac u ---- \ -- UA \ --------^ AC**

**zma-miRgs182 UGAUUCAGGUGCUGUUGGCGA**

**- - A C -- a A AC A .-G UG--- .-ACC .-CUAGAUCAAAAC| AAGA**

**UCC GG GG Guga uuc ggugcuguuggcgaUUGUCGUCGCCCC GCGC GUGC GACCAUCC GGCGCCA UGCGA GC AGGG \**

**AGG CC CC CACU AAG CCACGACAAUCGCUAGCGGCGGCGGGG CGCG CGCG CUGGUAGG CCGUGGU ACGCU CG UCCU C**

**G A C - CU A A CA C \ - UAGUA \ --- \ ------------^ CGCU**

**zma-miRgs183 UCACGUCGAUGCUUAGGAGGCU**

**- GAG - g a- - uCA-- -- - - | UGC U**

**CAU UAA Aucacgucgau cuu gga ggc GG AAG CU CCA--GGAGUAG UCGGUGAG \**

**GUA AUU UAGUGUAGUUA GGA CCU CCG CC UUC GA GGU CCUCAUU AGUUAUUC U**

**G AGG C G AC A UUCUG AA U C \ ^ UA- C**

**zma-miRgs184 CGCGUACCGAACUGCUAGGUUU**

**- GA - - guac aa -| uGCG**

**GAG AG CGC cgc cg cu gcuagguu \**

**CUC UC GCG GCG GC GA CGGUUCGG A**

**G A- G A GAGA GG G^ UAGC**

**zma-miRgs185 UCGUACCUGAGGCGACGAUGCA**

**G GC GG- A C U .-AU| A G**

**GGGGGC AUUG CGUCG CCUCG GU UGAG GA GGC A**

**CUCCUG UGac gcagc ggagu ca gcuC CU CCG G**

**- AC gua - c u \ --^ G C**

**zma-miRgs186a ACUCCUAAGGUUGUAGAAGGCU**

**- A Uac uaa --| a UUACUAGG A U AAC**

**AUCU AAUU ucc gg uugu gaaggcuUU AUAUG C CA \**

**UGGA UUGA AGG CC AACA CUUCUGAAA UAUAC G GU A**

**U G UU- UCA UA^ A -------- - - AAC**

**zma-miRgs186b ACUCCUAAGGUUGUAGAAGGCU**

**AUCUAAAU| c uaa -- a UUA---- - AUG**

**UUa ucc gg uugu gaaggcuUU CUUG GUU A**

**GAU AGG CC AACA UUUCCGAGA GAAC CAA C**

**UUGAAGUU^ C UCA UG A UAUACGG A ACU**

**zma-miRgs187a UCUGAACAGCGCGCUGCCGGA**

**U - U AUGC -------- G UG AA- G UCU**

**GUUUGGC GU UGCUGU GGGCU GU CG UGUUUGCUG UCUGU UG U**

**Caggccg cg gcgaca cuCGG CA GU GCGAGCGGC AGGCG AU G**

**- u c agu- GGCUUCAG G GG GCG G CCU**

**zma-miRgs187b UCUGAACAGCGCGCUGCCGGA**

**G| A UAACGUUGUUU - U AUG - -------- G UG AA- G UCU**

**GGCUC GG GUUUGGC GU UGCUGU CAG GCU GU CG UGUUUGCUG UCUGU UG U**

**CCGAG CC Caggccg cg gcgaca guc CGG CA GU GCGAGCGGC AGGCG AU G**

**A^ G ----------- u c a-- u GGCUUCAG G GG GCG G CCU**

**zma-miRgs188a AGGGUUUGCCUGAUGAGAUGG**

**AGA-- .-UCG CA ------------------------- CUA UA CAAAAC U CAA- .-ACC|UG U**

**UCC UUAUUUUUCU GGC UGAUAUA GU AGGGU UGA GUGCAAU GCUU G CAA \**

**AGG AAUAGAGAGA UCG ACUAUAU CA UCCCA GCU CAUGUUG UGAA C GUU U**

**AGCAC \ --- A- UAUCCUACUUAAUCUUGAAUUUUAA AAC -- UAUACU U AUAC \ ---^GU G**

**AAAAGAGAAUC G - A U .-AACC GCG**

**CUAU UCG CAG GUAAAC CC GGGUG G**

**ggua agu guc cguuug gg CCCAC A**

**CA--------- g a - - \ ---- GUA**

**zma-miRgs188b AGGGUUUGCCUGAUGAGAUGG**

**UAGCUCAA A - AGA C .-GUA| AG**

**AUCCA CU AUCGG GUAAACCCUAG UAG GC \**

**UAggu ga uaguc cguuugggaUC GUC CG A**

**AACACAAG a g --- C \ ---^ UA**

**zma-miRgs189 GCAAGUGAUGAUCAUACUCGGA**

**- UUA g aug u-- .-CC| U**

**AUUGAGC gcaa ug auca acucggaUU CC U**

**UAACUCG UGUU AC UAGU UGAGCUUAG GG C**

**G --- G CG- CUU \ --^ A**

**zma-miRgs190a UAACGUGAUCGUUUCUCGGCUG**

**UACAA c ga- --| UC U UUCUCUCUU CUUAAUU CC GGA UG U C C UUCUAUU U**

**UCUUGuaa gu uc guuucucggcug UU UUCUCU AGC GCGAAC ACGU ACGUC U UA UUA UACA GAAUGG G**

**AGGACGUU CA AG CGAGGAGCUGGC AG AAGAGA UCG CGUUUG UGUA UGUAG G AU AAU AUGU CUUGUC U**

**CGAAG - AGA CC^ UU C UACGC---- ------- UA --- GU U A U UUU---- A**

**zma-miRgs190b UAACGUGAUCGUUUCUCGGCUG**

**UAUAA G g c u --- g UUC--- --- U .-UUAAU CC-- GA-**

**UCAU uaac ugau guu cuc ggcu UCUUUU UCUUU CUC CUUAACC UGCGAGC GCGUG A**

**GGUG GUUG GCUG CGA GAG CCGA AGAAGA AGAAG GAG GAGUUGG ACGCUCG UGUAU U**

**ACGGA - - A C UCC - CACUCA CCC - \ ----- CGUU GUA**

**zma-miRgs191 UAGGAGGCUCAGCAAGCUCCGG**

**UUCAAGAACAA UCC A- A A - .-ACCC AC .-AACUC GA .-CCCCAA AU**

**CUG GC GCUGA GCC CC AGG GCCU UCC GGU AUGG GA \**

**ggc cg cgacu cgg gg uCC CGGG AGG CCG UGCC CU A**

**AG--------- cu- aa - a a \ ---- A- \ ----- AC \ ------ CC**

**zma-miRgs192a AUUUACUUAAUGUAGGGCGCC**

**A AU ---- u u-| a G**

**UACC GUC au uacu aaugu gggcgccC A**

**AUGG CAG UA AUGA UUGUA UCCGCGGG A**

**A -- AAAU U UC^ A G**

**zma-miRgs192b AUUUACUUAAUGUAGGGCGCC**

**AUA .-Ca u-| a G**

**CCAUGU uuuacu aaugu gggcgccC A**

**GGUAUA AGAUGA UUGUA CCCGCGGG A**

**CUA \ -- UC^ A G**

**zma-miRgs193 GGGGAUGUAGUUCAAAUGGUA**

**A| CC A - U A- U ACGUUUUC GG**

**CGA CGUUCUA CUAUU GAGCU AUAUUU UGA GUG AUUAUGUU C**

**GCU GCAAGau gguaa cuuga uguagg ACU CAU UAAUACGG U**

**C^ C- - a - gg C GA------ UA**

**zma-miRgs194 AGACACUCGGCAAAGACAAC**

**C .-UA caa GACAC CGAAC--- - -----| AGCCG**

**GGCACA Aagacacucggcaaaga cUUU UCGA GC CGGCGCUCGGCAAAG GGCCGUCAGC \**

**CCGUGU UUCUGUGAGCUGUUUCU GAAG GGCU CG GCCGUGAGCCGUUUC CCGGCAGUCG U**

**A \ -- UC- AAAC- CACAAUCU A UAUUG^ AUAUC**

**zma-miRgs195a UUUUUUGACACUCGGUAAA**

**- Cuu--| c AG**

**GCUGAGUG uuuuugacacu gguaaaG \**

**CGGCUCAC AAAAACUGUGA CCGUUUC C**

**A AAUAU^ A UU**

**zma-miRgs195b UUUUUUGACACUCGGUAAAG**

**- UCuu--| u G**

**GUCGAGU uuu ugacacucgguaaag G**

**CGGCUCA AAA ACUGUGAGCCGUUUC C**

**A CAACAU^ C U**

**zma-miRgs196a UCAGUUUGAAUUGAUAGUUGC**

**.-AGUAUGC| c u ag C UU**

**UGAu agu ugaauugau uugcAA GAU A**

**AUUG UUA GUUUAAUUA AAUGUU UUG U**

**\ -------^ U U -- U UU**

**zma-miRgs196b UCAGUUUGAAUUGAUAGUUGC**

**.-AGUAUGC| c u ag C UU**

**UGAu agu ugaauugau uugcAA GAU A**

**AUUG UUA GUUUAAUUA AAUGUU UUG U**

**\ -------^ U U -- U UU**

**U UUUUCA A UAAAAAG UAG--- - UU - .-AAAA A UU--- CA A U**

**AAUGUGUGG AAUU GAAAU AAUUA CUU UUAUGU UUGU AAAC AUAAGAG UAGUUA UAU AGAU UUUG A**

**UUGCACACU UUAA CUUUG UUAAU GAA GGUAUA AACA UUUG UAUUCUC AUCGAU AUA UCUA AAAC C**

**- UG---- C UGA---- UUUAAA U UU C \ ---- C CAUUU AA A A**

**zma-miRgs197 CCCAAGGUCGGGCGAGGCGGUA**

**UCG G a - .-ga aA U .-GA| C**

**GUCCGA ccca gg ucgggc ggcggu UUCC CC GGU G**

**CGGGCU GGGU CC AGCCUG CUGCCA GAGG GG UCG A**

**GAG G C G \ -- -- U \ --^ G**

**zma-miRgs198 ACUGGCUCACCAUCGUCUGGC**

**GACAGA a u u .-cCCCUUAACAAGCUC A CGU- UCC ------------| UG AA UCCAGCCCUCG AA**

**GUAC cuggc caccaucg cugg GC GA CCCCAG GAAG GUCGUUUGC CCACCA UGUU GUG \**

**CAUG GGCCG GUGGUGGU GGCC CG CU GGGGUU CUUC CAGUAAAUG GGUGGU ACAA CAC G**

**GAUCGA - - - \ --------------- A UCCU --- UCUAAUACCGGA^ GU GA UAAA------- CC**

**zma-miRgs199 AGGUUGUAGGAAGUUAGCGGCU**

**UUACUAGA ---- C U ---| C**

**GGagguuguaggaaguuagc ggcu G UUGAC CG A**

**UUUUCGAUAUCCUUCAAUCG UUGA C AACUG GC C**

**AAACAAUA AAAG - - AUU^ G**

**zma-miRgs200a UAACUAGCCGUCGGAGAUAAG**

**UUAUCUCCGACGGCUAGUUAC**

**C ACCG UU AAA -| A**

**GGG UG AA AG CuuaucuccgacggcuaguuacGAGGCCGUCGGAUAUA C**

**CCC GC UU UC gaauagaggcugccgaucaauGCUCUGGCAGCCUGUAU C**

**A GAGA CU AUA U^ U**

**zma-miRgs200b UAACUAGCCGUCGGAGAUAAG**

**GUUAAAAAA| U UAA**

**GCUUAUCUCCGACGGCUA UUACGAGGCCGUCGGAUA C**

**Ugaauagaggcugccgau aauGCUCCGGCAGCCUGU C**

**CUUUAUAUC^ c UUU**

**zma-miRgs200c UAACUAGCCGUCGGAGAUAAG**

**GUUAAAAAA --| A**

**GCUUA UCCGACGGCUAGUUACGAGGCUGUCAGAUAUA C**

**Ugaau aggcugccgaucaauGCUCCGGCAGUUUGUAU C**

**CUUUAUAUC ag^ U**

**zma-miRgs201 GAAGAUUACACUCAUGAUGU**

**UUGACCUA a c u .-AC .-UA| AUCU GG A**

**AAg agauuaca uca gauguGG CUGU CAUCA GCU UUGU U**

**UUC UCUAAUGU AGU CUAUACU GACA GUAGU CGA GACG A**

**UCCCGAAC A - - \ -- \ --^ CC-- UU U**

**zma-miRgs202a UUGAAGGGGAUUGGAGAGGAU**

**UGC GG g u-- .-AA| A CAA**

**GAAG Auugaag ggau ggagaggauUA UCCCUUC UAUU U**

**UUUC UAAUUUU CCUA CCUCUCUUAGU AGGGAAG AUAA A**

**AAA A- A UCU \ --^ G AUU**

**zma-miRgs202b UUGAAGGGGAUUGGAGAGGAU**

**UUA| A aa AA U AGG**

**GAGU GAuug ggggauuggagaggauUAAAUCCCCUCUUAUAC AUUG AU A**

**CUCA CUAAC CCCCUAACCUCUCCUAAUUUAGGGGAGGAUAUG UAAU UA G**

**AAC^ C -- C- U GGG**

**zma-miRgs203 ACUAAAACAAAACGGAGGGAGU**

**A ----| CU A U A**

**UAGU UACUCCCUCCGUUU UUUUAGUUGUCGCUGGAU GU UA U**

**AUCG Augagggaggcaaa aaaaucaAUAGCGACCUA CA GU U**

**A GUUG^ ac C C U**

**zma-miRgs204 CGGAGGGAAUUGGAGGGGCUA**

**-| G c ga AUCUUCUCAUCCAA**

**GC ACAAAAU ggagg auuggaggggcuaGAAUC \**

**CG UGUUUUG CCUUC UAACCUCCCCGAUCUUAG A**

**U^ G U UC AAAAAAAAUAAAUA**

**zma-miRgs205a CUAUUGGACUUUGUGUGCGGUG**

**GG C AGCG G G C CGA .-C| CA CG AG G- CAAC**

**CAGG GGC GC GCA GCAG AG CGGUAGG AGG GG GCGGC GCA GCAUG \**

**GUCC CCG ug cgu uguu uc guuaucC UCC UC CGUCG CGU CGUGC A**

**GG - Gg-- g g - ag- \ -^ AA AU G- GG AGGG**

**zma-miRgs205b CUAUUGGACUUUGUGUGCGGUG**

**GG C AGCG G G C CGA .-C| CA CG AG G- CAAC**

**CAGG GGC GC GCA GCAG AG CGGUAGG AGG GG GCGGC GCA GCAUG \**

**GUCC CCG ug cgu uguu uc guuaucC UCC UC CGUCG CGU CGUGC A**

**GG - Gg-- g g - ag- \ -^ AA AU G- GG AGGG**

**zma-miRgs206 GUCUUAGACACUCGGCACAG**

**UU u .-cagAACGCGAUUC| U**

**UGCCGAGUgucu agacacucggca CGGUAG C**

**ACGGCUCACAGA UCUGUGAGCCGU GCCGUU C**

**AC U \ -------------^ U**

**zma-miRgs207 UUUACAACGAUCGGACGGUCC**

**- - u .-aac acg -| CG -------- .-UUUUUCCAAAAAAAACUUCUC U AUC**

**GUUUUUUG GUuu ac gaucgg guc cG CAU AGGUGU CUG UCGAAAUA U**

**CAAAAAAC CAAA UG UUGGUC CGG GC GUA UCCACG GAC AGCUUUAU A**

**G A C \ --- AAA A^ AA GUAAAGAG \ --------------------- U GGC**

**zma-miRgs208 UGGACGAGAAAAAGUACGGUUU**

**G ggac u g ------ - A A A-- CC U .-UCUUCGC CCCU UU- CC-| UCA**

**GAAUGUUGGu gagaaaaag ac guuuU GG CUAGA GGCCGG UG CGAA GU GCC CUGG GA AGG UC C**

**CUUAUGACUA CUCUUUUUC UG CAAAA CC GAUUU CUGGCC AC GCUU UA CGG GACC CU UCC AG C**

**A AAC- U A UACUCU A A A GUC UC C \ ------- CUU- UAU CUA^ UCA**

**zma-miRgs209 UCUAGCGGACAAGACUGGUUG**

**G| GAGG C UA UA C UGCCUU CUCCG**

**CAGGU UUC AUCG UCUUGUU UGCU GA GAAGUGCU \**

**GUUCG GAg uggu agaacag gcga cu UUUCACGG A**

**-^ AUG- u c- -- u ------ CCUUA**

**zma-miRgs210 UUGGGCUAGACUUCGGUUUUU**

**CCC| CUG GA- A GU G**

**GCCGG AAGGAGCUGAAG GGC CUAA CA G**

**CGGCC uuuuuuggcuuc ucg gguu GU U**

**UUU^ A-- aga - -- A**

**zma-miRgs211 ACACAGACUUAGAAUACGGUUC**

**AG- AC CUUCAC UCU -| A GG C GC**

**CC GGG GCCGU UUCU AGUCU UG GC GAG \**

**GG CCC uggca aaga ucaga ac CG UUC U**

**GCA AA cu---- u-- u^ c a- - UA**

**zma-miRgs212 GCUUAAACUGAUAGGAGGAGGU**

**CAACU u ug- -- A -- .-UAUUA| UCCA GGU**

**CAAAAgc uaaac auag gaggagguGGGC AUCC ACUUA CAU ACAGA U**

**GUUUUCG GUUUG UGUC CUCCUUCACCCG UAGG UGGAU GUA UGUUU U**

**CUCGU - UCA UU G UA \ -----^ UG-- ACU**

**zma-miRgs213 AGAAGGUGGGACAUGAAUGG**

**- ggga --- GC AA .-AU G .-GAGA -| G**

**CUGGGCUC--GUagaaggu caug aaugg UG UGCA GC UCUGA CUG UCCGC U**

**GAUCUGGG CGUCUUUCA GUAC UUACC AC ACGU CG AGAUU GAC AGGCG A**

**U \ AAG- AGA A- -- \ -- - \ ---- A^ A**

**zma-miRgs214 AAGGAAUGAUGAUUUUAAGGCG**

**AAAAUAU AA -- A - .-C| A UAA**

**GCCCGCU AAAAU UCAU CC UAUGA GG UA A**

**UGGgcgg uuuua agua gg aUAUU CC GU A**

**AAAAUUU aa gu a a \ -^ C UUU**

**zma-miRgs215 ACGGACUGUCCGGCCUCAGG**

**GA--- AGCa u - a CA C G G- GA .-AAAAACUC ---| UU AAAAUGAA A G CAGA**

**GACCG cggac guccg gccuc gg CGGAC GUCCG UC GCGCA AACG GAAGGUG ACGGG CGGU UUAU GCG CCUCG \**

**UUGGC GCCUG CAGGC CGGGG CC GCCUG CAGGC GG CGUGU UUGC UUUCCAU UGCCU GCUA AGUA CGU GGGGC C**

**GACGG ---- C G A AG C G GA AG \ -------- CAG^ -- GACC---- - - CUGC**

**zma-miRgs216 AAAGGCAAUUUUACACUCGGCAA**

**CACA G aa -| aa u G**

**CUU GC ag gc uuu acacucggcaaA \**

**GAA CG UC CG AAA UGUGAGCCGUUU G**

**AAAA A GC A^ AA C C**

**zma-miRgs217 CAGAGCUGAACUCGACACAUGC**

**A| U G A GG AUAC UA**

**UAGCA UUAAG GUGUGUU GAG UCAGCU UUAG G**

**AUCGU AAUUc uacacag cuc agucga AGUU A**

**-^ U g - a- gac- UC**

**zma-miRgs218 UUCAAGGAAGGUGGCAAAUGCA**

**A CUCC C A A .-UGUA A U-- UU AA .-AGUA A**

**CC CA UUUGCCA UC UCUUU AAACAA UCCU UUG UUUCU AGC CUA G**

**GG gu aaacggu gg aggaa UUUGUU AGGA AAU AAGGA UUG GAU C**

**- CCac - - a \ ---- A CGU GG AC \ ---- A**

**zma-miRgs219 CGAGAUCAUGUCCCUUGAGCGG**

**AAU C CGA G CU C G G .-AAGGUU| UC**

**GCU CUG UCCG UCGG GG GA AUGAUCU GCC GCUUC \**

**CGA GAC Aggc aguu cc cu uacuaga cGG CGAAG C**

**UUU - --- g -- - g g \ ------^ CG**

**zma-miRgs220 UUAGUUAGAACAUAGGAUGCAU**

**C UC C--| GCCCCC UAC CU**

**AAG UUUGAU UCUUGUGUUU AACA UUGGCAA \**

**UUU AAACua aggauacaag uuGU GGCCGUU A**

**U GA cgu^ auuga- UUA CU**

**zma-miRgs221 UCAAUUAGUAUCAGAGCUUCGU**

**UUUAACAAUCUUU GUU AA UUC U UUCACC**

**CGAAGUUU GUG AAUU AGA UAGCUUA \**

**gcuucgag uau uuaa uUU AUCGGAU C**

**CAu---------- ac- ga c-- C CUCCCC**

**zma-miRgs222 UCCUGUCUUGCGGUCUUCCACC**

**U| AGAUCCAG - - UCU AAUC**

**UGCU CGAAUG UGGGAGGCU UGAG CAGGA \**

**GCGG GCUUAc accuucugg guuc guccu U**

**U^ -------- c c u-- ACCC**

**zma-miRgs223 AUGAGAUUUAUAUGAGUGAGCA**

**- AAG gau a .-caCAUUAAUA AA-| A**

**ACAUG UGauga uuau ugagugag CAG GGUG U**

**UGUAC GCUAUU GGUA ACUUACUU GUU UCAC C**

**A CA- --- A \ ---------- ACA^ C**

**zma-miRgs224 UGGAAACGAGUCGAGCCGAG**

**GG-| A G aa U**

**ACU GGG ugga cgagucgagccgagC \**

**UGG CUC GCCU GCUCGGCUCGGCUCG C**

**AUG^ - G G- G**

**zma-miRgs225 CCUGCUAGACGGCGUAGGCGAU**

**A G GACCG UG GC AGAGGA - GC UUAAG .-GGCUGU AGGA- .-A| AGAG U**

**GAG UA CGU ACGUUGUC AGU GGGUG GC GC GC GGAGCUCGUGG GCUGGCGC UCCA GCC C**

**UUC GU gcg ugcggcag ucg uccAC CG CG CG CCUCGAGCACC UGGCUGCG GGGU CGG U**

**G G AUAua ga a- ------ A AA UAUA- \ ------ AGAUG \ -^ CCGA U**

**zma-miRgs226 AUGUAAUAGAAGGUGACACGAC**

**A| UG guaa-- gaa c - A CA AUAAUAA GC**

**CGAU AUCau ua ggugaca gacA CC UUCG UAC CC \**

**GUUA UAGUA GU CCACUGU UUGU GG AGGC AUG GG A**

**A^ -- AUACAG AUA - U - CC ACA---- AC**

**zma-miRgs227 CUGCUGAGGAAAGACACCUGGC**

**CC -- gc a c c g UAGAACUCAUACAU UUGU .-AA GUG UG AAA .-G AG .-UUCAUGA| G**

**UCAU AAGGcu ug ggaaaga ac ug cACCAC UCUGAUCU GAGAUG UGGU U AC AG UGGUAAG AUAC CAUU A**

**AGUA UUCUGA AC CCUUUCU UG GC GUGGUG AGACUAGA UUCUGC ACCA A UG UC AUCGUUC UAUG GUAG U**

**UU GU A- - - A G CU------------ ---- \ -- AA- GU GAG \ - A- \ -------^ U**

**zma-miRgs228 GUAUUAGACACUCGGCAAAG**

**A GGAGUGC - UCG .-AU UCUUG---| CGA**

**AUUUGU UUUCUUUGCU AGUGUUU ACACUC CAAAG GUCGAGUGC A**

**UAAGCA GAAgaaacgg ucacaga ugUGAG GUUUC CAGCUCAUG A**

**C ------- c uua \ -- UCAAAAAA^ AAA**

**zma-miRgs229 AAAGAUACUUGGUGGUGGACUG**

**AAGUAAA a-- .-uggugga| AUG**

**CUUaa gauacuugg cug A**

**GAAUU UUAUGAGCC GAC U**

**AGAAGAA AUG \ -------^ AUG**

**zma-miRgs230 UCUAUAAGAUGGCUAGGAUUC**

**AG -- u aa gg G .-AUAGGAAGAC| G**

**AAAGA UGUuc au gauggcua auuc CG AAG G**

**UUUCU AUAGG UG UUAUCGGU UAAG GU UUC G**

**AA CA U AC A- G \ ----------^ C**

**zma-miRgs231 CCGCUGACUGGUGGGGCCGGG**

**AG G A c a g- g CAG- .-GUA| G**

**GG UGG Gccg ug cuggu gggccg gCUGU GC GCGCG G**

**CC ACC CGGC AU GGCCG UUUGGC CGGCG CG CGCGC U**

**AG G - - A GG G CGGG \ ---^ G**

**zma-miRgs232 AUGAGUGUAAUCUUCUUUGGGU**

**GGUUAUAAA|G UG UC CGGA U GAGA A - CU - - A ACUU UACC C G**

**GG U AAG GAUUACAU UAUGAC G GAUC GUU CGUC UCU CU GUU UGUGCGCG GCCCC GG UG A**

**Cu g uuc cuaaugug guaCUG C CUAG CAA GUAG AGA GA CAA ACAUGCGU CGGGG CC AC A**

**CUCUGAAAA^g gu uu a--- - AA-- A U UU C U C CAAU CUA- - U**

**zma-miRgs233 AUCGAUCCUUUGUAGAACCGGUA**

**AUCC A A C C GACUAUUU .-AC| UUGA**

**CUUUA UACCGGUUCU GCAAAGGA UGAUA UAAA CCC CCCG \**

**GAAAU auggccaaga uguuuccu gcuaU AUUU GGG GGGU A**

**UAUA A - a A -------- \ --^ UUUG**

**zma-miRgs234 AGCCCGCCGUUCGGGAUACCU**

**.-UA| UCC cc cc - CAGCUCCAAUAA**

**ACU GAag cg guucg ggauaccu \**

**UGA CUUU GU UAGGC CCUAUGGA A**

**\ --^ C-- UU AA A AUAAUAUAGCUC**

**zma-miRgs235 UUCGAAGACGCAUGUUCCUCUA**

**- UU ---- a--- a .-cuaUUUCUUCUAAU| G**

**GA GCA UCGuucg agacgc uguuccu CUAAAGAG U**

**CU UGU GGUAAGU UCUGUG GUAAGGG GAUUUCUU C**

**A CG UUUU AUCA - \ --------------^ A**

**zma-miRgs236 UGGGAGGAACAUGAGGUGGAUU**

**UCA AUGug g au - -- .-AU| UU**

**GCCA ggag aac gaggu ggauuG GGUGUAG UCCC C**

**CGGU UCUC UUG CUUCG CCUGAU CCAUAUC AGGG C**

**CGG ----- G CC U CA \ --^ CC**

**zma-miRgs237 UGAACUCGUAGAUUAGAUGCA**

**UGCAGU AU cu - - .-aUUUCUAAGC .-UCAGAAAACAAAAAAUA| G**

**GU ugaa cguag auuag augc AUUUUUUUUCUUUC GAGU U**

**CG ACUU GUAUC UAAUC UAUG UGAAGAAAAGAAAG CUUA C**

**UAGAC- GU U- U U \ ---------- \ -----------------^ A**

**zma-miRgs238 AAUACACAUGGGUUGAGGAGG**

**- a -| A**

**UAUUGACCCCaauacacauggguugagg ggAUUG GGUGUAAAUUA \**

**AUAACUGGGGUUAUGUGUACUCAACUCC CCUAAC CUACAUUUAAU A**

**C A U^ C**

**zma-miRgs239 UCCAAGCUGUCCGGACGAGUAC**

**.-UU A C aa u- - G .-CCCUCGCUGAAC| C**

**GG CU CGucc gc gucc ggacgaguac AGGUCC UCC U**

**CC GA GCGGG UG CGGG UUUGUUUAUG UUCGGG AGG C**

**\ -- A A A- UC A G \ ------------^ G**

**zma-miRgs240 UGACCGGAGAGAAAGACAUGUU**

**A A- G-- A - .-G| UG**

**AAGGAG GAA GUCU UUC CUCCGGU GGUCAAGU \**

**UUUCUC Cuu caga aag gaggcca UCAGUUCA G**

**G AC gua - a \ -^ UG**

**zma-miRgs241 UAAAGGACGUCGGCCCGAAGGC**

**G .-GA A - -| A CC**

**GCCGUC--CC CCG AGUCU GCC UGCG GGG \**

**CGGUAG GG GGU UCAGG CGG GUGC CCU U**

**- \ \ -- G U C^ A GC**

**A-- UC G C- GA U**

**GGU UUGGGC GAU CCU UGUAG \**

**Ccg agcccg cug gga auAUC G**

**UCA ga g ca a- G**

**zma-miRgs242 UCGAAUUUGGAAGACUUGGGCC**

**CAC C UU - -- U --- G-| C UG**

**UG CUAGG GGCCU AGUC CUA GUUU UGU GCU GG U**

**AC GAUCC ccggg ucag ggu uaag ACG CGA CU U**

**UAC - -- u aa u cuG GA^ A UU**

**zma-miRgs243a GACGGCUGUUAGACGGAUCG**

**- AC- U c g-| g UA U AUU**

**UCCU GU Cga ggcu uuagacg aucgU GACAUAAGGUUAU UUCGAUGGCU A**

**GGGA CA GCU CCGG GAUCUGC UGGCA CUGUAUUCCAAUA AGGCUGCCGA G**

**A AUA U A AG^ - GC C AUA**

**zma-miRgs243b GACGGCUGUUAGACGGAUCG**

**- AC- g-| a g UA U UC AUC**

**UCCU GUUCgacggcu uuag cg aucgU GACAUAAGGUUAU U GAUGGCU A**

**GGGA CAGGCUGCCGG GAUC GC UGGCA CUGUAUUCCAAUA A CUGCCGA A**

**A AUA AG^ C - GC C GA AUA**

**zma-miRgs244 UAGAAAAUAUUUAGGACAUCU**

**.-UA CUGAA U G .-ACCACUAA A**

**UUUGUUUAUUUU UGUG GGGUGU UCAA GUA \**

**AGAuaaauaaaa acgu uccACG AGUU CAU U**

**\ -- ----- u A \ -------- C**

**zma-miRgs245 UUGCGGAGUAUGUUGAUGUGUA**

**UA - G CAGAG .-UUACAGAUGAUGA .-AUAAU UCUAUU GGUUA .-UUCU A**

**GAG ACACAU GAU AUACUUUGU UGAGG GUUGGUU GUGAUUAU UGGU GGUAUUCUUUCC U**

**CUC ugugua uug uaugaggcg AUUCC CAACCAG CAUUAAUA AUCA UCAUGGGAAAGG U**

**CA a g ----- \ ------------- \ ----- UC---- AUG-- \ ---- U**

**zma-miRgs246 AGAGGGACCAGCAGCAGCUUGC**

**GA A A - a-| a gc gcU**

**GA GG UGa gaggg cc gca agcuu C**

**CU CC ACU CUUCC GG UGU UCGAG A**

**UG G A C GA^ A -- AGC**

**zma-miRgs247 AUCCAUGAUUCUAGGAACGG**

**- a-| u - a CG**

**GAGCCGUUCC ucca gauu cuagg acggAGC C**

**CUCGGCGAGG AGGU CUAA GGUUU UGUCUUG U**

**U CA^ - U G UC**

**zma-miRgs248 AACGAGGGACCUAUGGAUGGA**

**CA aa ac--| a - U UCUUU**

**GCGA--ACUG cgaggg cu uggaugg aC GC \**

**CGCU UGAU GCUUCC GA ACCUGCC UG UG G**

**AA \ AG GUUU^ - G U UGCCU**

**zma-miRgs249 CAAAAGACCAUCUAACAGAUC**

**G| G AA GCAA C-- UG**

**UCGCACCU AAAcaaaagaccaucuaacagaucA UAUUUUUCAGGUUUUCUAA AAGA AUAU \**

**AGCGUGGA UUUGUUUUCUGGUAGAUUGUCUAGU AUAAAAGGUCCAAAAGAUU UUCU UAUA A**

**-^ G CA ---- ACU UC**

**zma-miRgs250 GCUUGCAGGACGUUGCCCUCGU**

**A--------- A U A - A C GC**

**GC AG GG CGACG CC GCG GCUC \**

**ug uc cc guugc gg cgu cgGG G**

**GUAGAGUCCA c - - a a u AG**

**zma-miRgs251 UGCACUAAAACGGACGCCUAGG**

**A ACUA u cgc a .-AUC A .-GAUUA | UU**

**GG GUA gcacuaaaacgga cu ggAUGU UG ACUCC GGUUGG--UCU A**

**CC CAU CGUGGUUUUGUUU GA CUUACG AC UGAGG UCAACC AGA U**

**A ACAG - UA- - \ --- - \ ----- \ ^ UA**

**zma-miRgs252 UUUGGGAAGAUAGAUGGAUUCA**

**UC A ---- aa- - u a a .-CGAA | A CCUAGAUU UA**

**GAC CUCU uuuggg gaua ga gg uuc UGGA GUGUGA--UGGAU UUGCC GC \**

**CUG GGGA AGGCCC CUGU CU UC AGG ACCU CAUACU ACUUA AGCGG CG U**

**AC - UUUU ACA A C G A \ ---- \ ^ - AACCGUGU AU**

**zma-miRgs253 GGUUUUGUAAAGAUAUCGGCUA**

**GU UG A- C- C .-C .-C U C CA- .-UAGC G**

**GGAG G AGCC UGUUUU AC GAAGACU CAU UC CUUU AUCUACGACU AUA G**

**UCUU U ucgg auagaa ug cuuuugg GUA AG GAAA UAGAUGCUGA UAU A**

**UU GU Aa cu a \ - \ - U A AUA \ ---- A**

**zma-miRgs254 GAAGGGAAUUGAGGGGGCUAG**

**C| U - a a UC A**

**AAG GG AAUg aggg auugagggggcuagAAUCCC ACUAUUUA A**

**UUC CC UUAC UCCC UAACUUCUCUGAUCUUAGGG UGAUAAGU A**

**A^ - A C C GA U**

**zma-miRgs255 AAGCAAUUCCUGGCCACAACUG**

**UAUGGGUCAGAGGACUGAUUUUA**

**C C- -- -| a c a G AG**

**UAUCA UUua ug ggucag gga ug uuuua UG A**

**AUAGU AAgu ac ccgguc cuu ac gaaAU AC U**

**U UA ca a^ - a - G GG**

**zma-miRgs256 UCGGGGACGGCUGACGGAGCUG**

**C A - ---- | cuga cugUAC G A UUUA**

**UCAUCC C Guc gggg--acgg cggag GCGG GACCC UGC U**

**AGUAGG G CAG CCCC UGCC GCCUC CGUU CUGGG GCG U**

**U C U GUUG \ ^ UA-- ------ A - UUCU**

**zma-miRgs257 AGGAGACCGGUUGCUGACGUG**

**AAAAUAUA agac a .-GCGGAA GA-----| GGGG**

**GGagg cgguugcug cgug UAGUGG UAUA G**

**CCUCC GUCAACGAC GCAC GUUACC AUAU C**

**CUUCGUCG ACAA C \ ------ AAGGGAG^ AAAA**

**zma-miRgs258 AUAGUAGUAGUAGUAGUAGUAU**

**UU| U UCC A - G AG**

**AUUUA UU ACUGCUA UUACU CUACUGU CA G**

**UAGAU AG ugaugau gauga gaugaua GU G**

**UU^ U ua- - u - AU**

**zma-miRgs259 GUGGAUUGGAUGGUAUUGAGUC**

**UUACUAUAGG AU CC .-G U CC--- A .-CU| U C GCGG**

**GGCUCAG UCAUCU UCUACA UGGC CGACGA UUUGU CUC GGGCU CC CCUU C**

**cugaguu gguagg aggugU AUUG GUUGUU AAACA GAG CCUGA GG GGAA U**

**CGUAAAUUUG au uu \ - - CACUU C \ --^ - A AAUA**

**zma-miRgs260 UGAUGAUGGAUAGAUUAACUU**

**UCU AU G GUAC U GCUAU UG -- UG|A**

**UCCAA GGGU GAUU UCUGUU UUAU AU UCU AUU U C**

**AGGUU uuca uuag agguag aguG UA AGA UAA G G**

**UAU GU a au-- u UAUUC GU AG GU^U**

**zma-miRgs261 UAGAAUGGAAAUGGGAAGAAAU**

**GAAGCUCA GG- - .-AC .-GAA| AGAU CAGGA U**

**UGAUUU CUUA UUCUAUUC CCUG GUACCA GUAC CC G**

**ACuaaa gggu aagguaag GGAC UAUGGU UAUG GG A**

**UAGGUAUG gaa a \ -- \ ---^ GGU- AAAA- A**

**zma-miRgs262 ACCUAAUACGCUCGGCGAAG**

**- - c- au- g | AG**

**GCCGAGUG CCac ua acgcucggc aagGA--CCU \**

**CGGCUCAC GGUG AU UGUGAGCCG UUCUU GGA U**

**A A AC GAC A \ ^ AA**

**zma-miRgs263 GCUUAUUUUCGGCGGCGUCUG**

**U| A A C A A**

**UUUUGGC GCCAGACGCCGCCGAAAAUAA CU UUUGCC CCGAAAAUA \**

**AAAACCG CGgucugcggcggcuuuuauu gG AAACGG GGCUUUUAU G**

**-^ C c U C C**

**zma-miRgs264 GAGGACUUCAGAGACGAAGUAU**

**UU U -- u--| cg auA**

**GA AAGCU gaggac ucagaga aagu C**

**CU UUCGA UUCCUG AGUUUUU UUCG U**

**UC U AC UUU^ CG GGG**

**zma-miRgs265 GGGAUUGGAGAGGUUUAAAUC**

**-| C ag u CCG AAUCAUUU**

**AAU UAGAGggggauuggag g uuaaaucU UCU \**

**UUA GUCUCCCCCUAAUCUC C AAUUUAGG AGA U**

**G^ A CU U --- AAAUCAAU**

**zma-miRgs266 UUACUUGGGACUAAAAGGCUAU**

**CUACCCCAA c - aaaa - UG .-UGU| UUA**

**Cuua uuggg acu ggcu auG GA UGAUG \**

**GGAU AACCC UGG UCGA UAC CU ACUAC A**

**UUUG----- - G CAC- U GU \ ---^ CAG**

**zma-miRgs267 GAGCUGUUCGACGGGCUCAAC**

**---- UAUA A AUGU A U C .-GGCCC CG C- AA**

**CGGU AGU GAG CCCG CGAGC GC CGGCCC GGUC AACC GUUG \**

**GCCG Uca cuc gggc gcuug cg gCUGGG CCAG UUGG CAGC G**

**GCAA C--- a ---- a u a \ ----- A- CA AC**

**zma-miRgs268 UCUUUAGCUGGAUCGUAUGGC**

**- AG - u a a G A-| A CUUCAAG UC G GA**

**UCUCUU GUucuu uagc gg ucgu uggcU CUG CAUCUUCC UCU GCC CU CA U**

**GGAGAG CGGGAG GUCG UC AGCG GCCGG GAC GUGGGAGG GGG CGG GA GU U**

**G AG C U G G A GA^ - ------- GA - AG**

**zma-miRgs269 UUGGAUGUGGGAGGGGAGAGGA**

**U .-U C a gag- ga-| G**

**GGU AGAA uugg ugugg ggga ggaC A**

**CCG UUUU AAUC GCACC CCCU CUUG C**

**U \ - U - ACGA AAG^ G**

**zma-miRgs270 CAAAGUGCGAAGGUUGUCAGAA**

**- A UC ---------------| g**

**GAUUU GU caaagu--gcgaagguugu ca a**

**CUAAA UA GUUUUA UGCUUCCAACA GU a**

**G C GU \ AAAACUAGAGAACGU^ A**

**zma-miRgs271 UCCGAGUCAGUAACAGCUCCAG**

**- --- U -----| c aacagc CU AC**

**GC UGCC GC Guc gagucagu uccag UCU \**

**CG GCGG UG CAG CUCAGUCA AGGUC AGA C**

**U CUU C CCUCC^ U GAGCCU CU CG**

**zma-miRgs272 ACCCGUUGGGUAGACGAGUA**

**AAGAACCUC| u C ACG U**

**Uacccgu ggguagacgagua GGGU GGU G**

**AUGGGUA CCCAUCUGUUCAU CCCA UCA G**

**UCAUAUAAA^ - A --- U**

**zma-miRgs273 ACUCGCUAUAUGUUCGGACGCU**

**A acuc - ucggac .-AAAAGGUUUAA| UG**

**UCUUUCAGU gc uauaugu gcuCA UGCA C**

**GGGAGGUCG CG AUGUACA CGAGU ACGU A**

**C A--- U ------ \ -----------^ UG**

**zma-miRgs274 UGAAGAGUCAAGUAGAACGGA**

**AC A CUAUU G- CCAU C- U AUGU**

**UCGU CUACU CUCU GGC CGCUGCCU GU UAGCGU G**

**ggca gauga gaga uUG GUGAUGGA UA GUCGCA A**

**a- a acu-- ag AUUU UU C GGAG**

**zma-miRgs275 AACUCCUCGGAUUCAAACAG**

**CAUUAA A - C C - U .-A UU C UCC C--| U UACAUUU**

**GGGCCU UUUGGAUCU AGGAG UAAAG AAAA UG CUAA GU AG UCC UAAGAAG UAAAAGUG CUAA \**

**CUCGga aaacuuagg uccuc aUUUU UUUU AC GAUU CA UC AGG GUUUUUC AUUUUCAC GAUU U**

**CCGAAA c c a A C C \ - UU U UUU UUU^ U UUAUCAU**

**zma-miRgs276 AACCUUAUUUUCGGCGGUCAG**

**CC c -- cg g G A--| GA AGC**

**GCCGAAAUaa cu uauuuu gcgguca GAC CAGGCCAUCGAAAAU GCC GUU \**

**CGGUUUUAUU GA AUAAAA CGCCAGU CUG GUCCGGUGGCUUUUG UGG CGA U**

**UU C UA AU - G CUG^ G- CUU**

**zma-miRgs277 ACGACGACGAGGACGACGAGGA**

**- .-acgac ac a -- .-CAACGA .-A .-AU| A**

**CCUG gacg gacgagg cgac gagga GG CGACGAC GGCG \**

**GGAC CUGC CUGCUCC GCUG CUCCU CC GCUGCUG CCGC C**

**G \ ----- GU - GA \ ------ \ - \ --^ C**

**zma-miRgs278 AGAUGGGGGUUGGAGAGGAUU**

**- A u -| ga A CAU**

**ACGU AAUCCaga ggggg uug gaggauuAAAUUCCUU CUAGU U**

**UGCG UUAGGUCU CCCCU AAC CUCCUAAUUUAGGGGA GAUCA U**

**A G U A^ UC G UUU**

**zma-miRgs279 GCUCCUGCAGCGAGGUCGUGG**

**G A U .-uc a a --- .-GUCCA .-GUC CG .-GAGCACCAC A CA**

**G AGGAG Cgc cugc gcg gguc guggACC GCCC GU CAGA CAAACG UA C**

**C UCCUC GCG GACG CGC CUAG CACCUGG CGGG CG GUCU GUUUGU AU C**

**G - - \ -- A A CUC \ ----- \ --- AU \ --------- A AU**

**zma-miRgs280 UGGAUAAGAACCAGAACUUGGC**

**GAUCUAAAGA a - acu-| cCU**

**uggauaaga cca ga ugg A**

**ACCUAUUCU GGU CU ACC U**

**CAAAACAAAG C A ACAU^ UCG**

**zma-miRgs281 UCACGGAAAGUUAGACGAGCC**

**A AUUAU - C UUAGA .-U| GUG**

**GCAUC GGCU G CU UCUGUG UUUG U**

**UGUGG ccga c ga aggcac AGAC G**

**- AAAAU g a uugaa \ -^ AGG**

**zma-miRgs282 AAUUAUGGCAAUUGUGAUCUUA**

**- AAC auu a uc .-CAU| UUU U U**

**UUG UAUAa auggc auuguga uuaA GUG UCG CUAUG C**

**GAC AUAUU UAUCG UAACACU AAUU CAC AGU GAUAU U**

**U GU- CU- - CU \ ---^ C-- - A**

**zma-miRgs283 UUCCUGCAGAAACGAACAGGCCC**

**U A U C .-GUU| U AGUA**

**GGCC ACU AGGGCC GUUCG GUUA GGAAUGG C**

**UUGG UGA Ucccgg caagc CGAU CCUUGCC U**

**- G U a \ ---^ - AAGG**

**zma-miRgs284 CAAGGAGAUAGUACGAGGCCU**

**C CAA G C CG U A C - A**

**GAGGCACGG GCCUCGUAU AUCUUCUUGGCA UGAGC CUUUAGGGCCUUGAAGUGAAGGU CCC AAAUGCUCGUGCCACUG CACGC CUC UC**

**UUCCGUGCC cggagcaug uagaggaaCCGU ACUCG GAAGUCCCGGAGCUUCACUUCCA GGG UUUGCGAGUACGGUGAC GUGCG GAG AG**

**- Auc a A AU C C C A C**

**zma-miRgs285 AGAAUAACUGCGGGAUGAUUGA**

**UGAAUCAAAAa -- au- .-C AAA- - .-UCAACAC| U**

**gaauaacu gcggg gauuga GAUAG AC GUACCCA GGG \**

**UUUGUUGA CGUCC UUGACU CUAUC UG UAUGGGU CCU U**

**CCAUUAUACCG UC GUU \ - CAAA A \ -------^ C**

**zma-miRgs286 GAGCACACUAUAUUCCUGAUGG**

**- A cac a - - a gA -------| AUU AG**

**G AGCUUUCCgag acu uauu cc ug ug GACUU GCA AAAACU \**

**C UUGAAAGGCUC UGA GUAA GG AC AC CUGGG CGU UUUUGA A**

**A - UA- C U C - UC ACGAAAU^ AAU GG**

**zma-miRgs287 UGCCUAGCGGACCUGGUAUCUU**

**A U--------- - ua--| cug uCA**

**CCGUUA AG ugcc gcggac guaucu U**

**GGUAGU UC ACGG UGCCUG CAUGGA A**

**A UAUUCAAGCU G UAGG^ A-- UAC**

**zma-miRgs288 AUAGGGACCGACGAAACGUGG**

**UUCA UU AC AA -- GA .-U -----| U U**

**CUGGC CUCACGUU UG GGUCUU UCU AUC GCACU GUC CUUC \**

**GAUCG Gggugcaa gc ccaggg aGG UAG CGUGG CAG GGAG U**

**UUUC -- a- ag au G- \ - UCUUU^ U C**

**zma-miRgs289 GAGGGGAUUGGAGAGGCUAAA**

**C UCCA-| a GCA CG A**

**UUAAA gaggggauuggag ggcuaaaAU CUC UAUUCA A**

**AAUUU CUCCCCUAACUUC CCGAUUUUA GAG AUAAGU U**

**A UGAGC^ C G-- A- U**

**zma-miRgs290a AAUGUUGCAAUGGUUCGUCUGU**

**A| U uu a ucu UAUGA U A C AUAGG U-- G**

**CAUGUACG aaug gca ugguucg gu UCUU AC UG GC AGUUGACA GUCUU C**

**GUGCAUGU UUAU UGU AUCGGGC CA AGAA UG AC CG UUAACUGU CGGAG U**

**A^ - C- - U-- UG--- - A - GGUUA CGC A**

**zma-miRgs290b AAUGUUGCAAUGGUUCGUCUGU**

**AGAAAU U GG UA UG ACAA- AAAGG AA---- .-AGUA GA**

**GCAUGGACG GCUA GU AG UUA AUG GUUCC UUCAC UC G**

**UGugucugc uggu cg uu aaU UAC CAAGG GAGUG AG A**

**U----- u aa -- gu GUAUG AA--- GAAACA \ ---- AA**

**zma-miRgs291 UGAACCGGUUUUCUGUUUUC**

**--| UG C UU -- A**

**AGUU ACCAGAAA CGG GAACCGGUUU CUG \**

**UCGG UGGUcuuu guc uuuggccaag GAC A**

**AC^ -- u u- uU C**

**zma-miRgs292 AUCGAACCGUCGGAGAUAAG**

**UUAG C .-UAA U - GUCG**

**CUUA CUCCGACGGUUU GCCGUCG GG AUAACGC G**

**gaau gaggcugccaag CGGCAGC CU UAUUGCG A**

**CG-- a \ --- - A AUAA**

**A----- G**

**GCCGUCGGACAUA \**

**CGGCAGCCUGUAU C**

**cuaCCC G**

**zma-miRgs293 UUGGAUUGUGAUGUCAAGAUUA**

**CUCUCCA -- - caa- CU AA .-A AC- .-AG A CG UA A UUC -| UA CUA**

**AUGuu ggau ugugaugu gauuaU GU CU UGA CCA UGC UUC AAACU GGG GUG--UCUC UG GAGU GGG \**

**UGCAG UCUG AUACUACA CUGAUA CA GG ACU GGU ACG AAG UUUGG CCC CAC AGAG AC CUCG CUU A**

**AUAUACC AG C UUAG AU AG \ - ACA \ -- G AU -- A \ UAU A^ GA UCC**

**zma-miRgs294 AAAAAGAAACGGAGGGAGUAGCA**

**AAUUACUUCCAAA CAA**

**UUACUCCCUCCGUUUCUUUUUAUUUGUCGCUGGAUAGUG U**

**gaugagggaggcaaagaaaaaUAAACAGCGACCUAUCAC U**

**AUUUac------- AUU**

**zma-miRgs295 GAUUAUGUGCGGAGGAAGAUGAA**

**GGUUUCCUCUGGCUGUAAAGAC**

**CAC ACGAA g - - u aga .-ACC C U .-U| G G AAG**

**GGA CCUU guu uccucug gc guaa cCUAU AGCUC UC GA ACUUCUGAU AU UUU C**

**CCU GGaa uag aggaggc ug uauu GGGUA UCGAG AG CU UGAAGGCUA UG AAA A**

**AUA GG--- g a g - ag- \ --- C U \ -^ G G CUG**

**zma-miRgs296 UAGUAUAUUUGAGACACUUGAG**

**CACUA A ag u cuu- .-GCG| U UUU**

**CG UGu uaua uugagaca gagA UUAUA AAUUU \**

**GC ACG AUGU AAUUUUGU CUCU AAUAU UUAAA G**

**GUUUG C GA - AUAU \ ---^ U UUA**

**zma-miRgs297a AGGAUUACUCUAAUGAGCUGCA**

**A------- AGCC- U - .-AU GUG G- GCA CAUA-- A A GGCAG**

**GCAG UAGA GUA UCCUUCA GAGCG CCUU AC ACU UUUUGGA CU CU \**

**cguc aucu cau aggaGGU UUCGC GGAA UG UGG AAGACUU GA GA A**

**AAGAAGUa gagua - u \ -- AA- GA AGG UUAAGG C C AGAAA**

**zma-miRgs297b AGGAUUACUCUAAUGAGCUGCA**

**C GCAGGUGGA .-GCA - UAGAUGU UCAAUGA G UG .-CGCAACUCAUA| A A GGCAG**

**GAC GCAGCU GC AGAGCC AUCCU GCGGU CCU A UUUUGGA CU CU \**

**CUG cgucga cg ucuugg uagga UGCCG GGA U AAGACUU GA GA A**

**- AAGAAGUa- \ --- a uu----- UUUG--- G GU \ -----------^ C C AGAAA**

**zma-miRgs298 UACUCAAAUUCAAGGAAGGUAA**

**- AA AA u aauuc- g U .-A ----| U**

**UC GG GG acuca aa gaagguaa GC GGA AUUGU U**

**AG UC UC UGAGU UU CUUCCGUU CG CCU UAACG U**

**U GC GG - GGAUGC G - \ - CAAC^ A**

**zma-miRgs299 AAGCGCGGUAGAAGAUAAACUA**

**ACUAUCUC - G AA | U**

**GUUGGUUU UCUU UG GCGC--CCCGA C**

**CGaucaaa agaa au cgcg GGGUU C**

**ACCGAACC u g gg \ ^ U**

**AAA--- - .-A CC**

**--ACUC UGUGAUA CA UUACUAGUC A**

**UGAG ACAUUAU GU AAUGAUCGG G**

**\ ACCAUA U \ - AG**

**zma-miRgs300 UUUGGACCUGUGAUGACACCAAA**

**C| A C GUC**

**UAAACUUGUUUUUGGUGUCAUCA AGGUCCGAA AG U**

**GUUUGAACAAaaaccacaguagu uccagguuu UC C**

**-^ g A AAA**

**zma-miRgs301 UCUAGUCGACGAUCAAUGGCCA**

**UC U G ACGA UC A UAU C--- ACA-- - ------ .-AAUAGCCAUGAAC -----| U A A CU**

**GGG GU GUGU GCC UGGUCG UGAUU GGC AC GCCCC CA UUUUUGUCGAA GACCAUUU UCAA AAU CCG AGG A**

**CCC CA CACa cgg acuagc gcuga CCG UG UGGGG GU AGGAACGGCUU CUGGUAAA AGUU UUA GGC UCC A**

**C- - A c--- ua a ucu AGCA AGGAG U AUACAA \ ------------- GAACC^ U - A AC**

**zma-miRgs302 ACGAAUAUUCGAGAACGGAGG**

**GCA| AAUU C UUUA G- UC A**

**GGU ACU CCUCCGUUCUU AUUUGUCGC GUU GUUCA A**

**CCA UGA ggaggcaagag uaagcaGCG CGA CAAGU A**

**UUA^ ---- U cuua GG U- A**

**zma-miRgs303 UCGAGCGAGCCGAACCGAGCC**

**CUUGAGAUCGGAGCAGCUCGGCU**

**C UC -| aga agca -- C**

**UC GAGCC GGcuug ucgg gcucg gcu G**

**AG UUUGG ccgagc agcc cgagc CGA C**

**- UU A^ ca- gag- uU G**

**zma-miRgs304 CAAAGAAAGUAGCCACAUGCCU**

**A| C G ACA A- UAAGAU**

**UGA AUCACAAGGCAUGUGGC UGC CU GUUA \**

**ACU UAGUGUuccguacaccg aug ga cAAU A**

**-^ C - aaa aa UAUGGU**

**zma-miRgs305 UCGAAGGGGAUUGGAGAGGUU**

**-| C a a G G A**

**AGCCA AAAAucg aggggauuggag gguuA AAUCC CUUCUUAUUCA A**

**UUGGU UUUUGGU UCCCCUAACCUC CCGAU UUAGG GAAGAAUGAGU A**

**C^ A C C G G U**

**zma-miRgs306 AGGGGCAUGUUAUCGAUCUUCGG**

**G C Aa--- gg uu c--- - .-AAA .-AAAAGG| AA**

**GCA GAGA gg caug au ga ucuucgg AUGCUUUG GGUA \**

**CGU CUUU UC GUAU UA CU GGAAGCC UACGAAGC CCGU U**

**A A GUAAU AU GG AAAA C \ --- \ ------^ AC**

**zma-miRgs307 UUCAGAACAACGAAGAAGACCA**

**- CGU gaa| cga .-aAACUUUUGUUCAAU UC A**

**GCUUCUG uuca caa agaagacc GAC GU U**

**UGGAGAU AAGU GUU UCUUCUGG CUG CA C**

**G --- G--^ CCG \ --------------- CU C**

**zma-miRgs308 AAGUACGAAGGUUGUUUUUGAU**

**GA CU CU G UG .-CAA .-AAA| C**

**UGC GA GAU CAAAA GAUCUUCGUAC UUUGGUCAUAG UC \**

**ACG CU Cua guuuu uuggaagcaug aaAUCGGUGUU AG C**

**AC UU -- - ug \ --- \ ---^ U**

**zma-miRgs309 UUCGACAAGACGCUCAAGGAGC**

**U AA-- - ca g aa a | U AC**

**GC ACCUUu ucga agac cuc gg gc--CG GCCCCU C**

**CG UGGAAG GGCU UCUG GAG CC CG GC CGGGGA A**

**G GCGG U CG - GG G \ ^ C CU**

**zma-miRgs310 UCUAGAUCCAACGGACCAAAA**

**UUGGUCCGUUAGAUCUAGAUC**

**G C a A | U**

**CAAG AGCUCUUuugguccguu gaucuagaucUAA CGUAU--GCUA A**

**GUUU UCGAGaaaaccaggcaa cuagaucuAGGUU GCAUA CGAU U**

**- A c G \ ^ U**
